# Supplementary material for: Exploring the contribution of risk factors on major illness: a microsimulation study in England, 2023-2043
Source: Nat Commun. 2025 Nov 4;16:9402. doi: 10.1038/s41467-025-64820-1 (PMC12586531; doi:10.1038/s41467-025-64820-1)
Supplement: Supplementary file 1 — Supplementary Information [file 41467_2025_64820_MOESM1_ESM.pdf]

Supplementary information

Supplementary information for *Exploring the contribution of risk factors on major illness: a microsimulation study in England, 2023-2043.We provide two sets of supplementary materials:*

Supplementary Methods – Technical Appendix

Supplementary Results

Record Statement

Supplementary Methods - Technical Appendix

UNIVERSITY OF LIVERPOOL

The IMPACT$_{NCD}$ technical appendix

Anna Head, Toby Watt, Ann Raymond, Laurie Rachet-Jacquet, Max Birkett, Chris Kypridemos

Table of Contents

1. Introduction 1

2. Epidemiological engine 3

High-level description 3

Sociodemographic module 6

Exposure module 7

Clustering of risk factors 9

Disease module 9

Disease incidence 10

Estimating the observed incidence probability IObserved 14

Initial prevalence 14

Conditions with recovery and recurrence: asthma, anxiety and depression, constipation, pain, alcohol problems and cancers 14

Dependencies between conditions 14

Disease duration 15

Mortality 16

Mortality calibration 16

3. Model outputs 17

Uncertainty and probabilistic sensitivity analysis 17

4. Validation and calibration 18

5. Definition of the Cambridge Multimorbidity Score and defining conditions in primary care data 19

6. Strengths and limitations of this modelling approach 26

7. References 27

A) Appendix A: Disease module details 32

Model structure 32

Coronary Heart Disease (CHD) 34

Validation plots for CHD incidence, case fatality, and prevalence 40

Stroke (including transient ischaemic attack (TIA)) 41

Validation plots for stroke incidence, case fatality, and prevalence 46
Breast cancer 47
Validation plots for breast cancer incidence, case fatality, and prevalence 51
Colorectal cancer 52
Validation plots for colorectal cancer incidence, case fatality, and prevalence 55
Lung cancer 56
Validation plots for lung cancer incidence, case fatality, and prevalence 60
Prostate cancer 61
Validation plots for prostate cancer incidence, case fatality, and prevalence 64
Other cancers 65
Validation plots for other cancer incidence, case fatality, and prevalence 67
Chronic obstructive pulmonary disease (COPD) 68
Validation plots for COPD incidence, case fatality, and prevalence 71
Atrial fibrillation 72
Validation plots for atrial fibrillation incidence, case fatality, and prevalence 75
Heart Failure 76
Validation plots for heart failure incidence, case fatality, and prevalence 78
Type 2 diabetes mellitus 79
Validation plots for type 2 diabetes mellitus incidence, case fatality, and prevalence ... 83
Chronic kidney disease (CKD) 84
Validation plots for chronic kidney disease incidence and prevalence 87
Dementia 88
Validation plots for dementia incidence, case fatality, and prevalence 91
Hypertension 92
Validation plots for hypertension incidence and prevalence 95
Asthma 96
Validation plots for asthma incidence and prevalence 99
Alcohol problems 100
Validation plots for alcohol problems incidence and prevalence 102

Anxiety and depression 103
Validation plots for anxiety and depression incidence and prevalence 105
Constipation 106
Validation plots for constipation incidence and prevalence 108
Pain 109
Validation plots for pain incidence and prevalence 111
Psychosis 112
Validation plots for psychosis incidence and prevalence 114
Epilepsy 115
Validation plots for epilepsy incidence, case fatality, and prevalence 117
Rheumatoid arthritis 118
Validation plots for rheumatoid arthritis incidence, case fatality, and prevalence 119
Connective tissue disorders 120
Validation plots for connective tissue disorders incidence, case fatality, and prevalence 121
Type 1 Diabetes Mellitus 122
Validation plots for type 1 diabetes mellitus incidence, case fatality, and prevalence . 123
Hearing loss 124
Validation plots for hearing loss incidence and prevalence 125
Irritable bowel syndrome (IBS) 126
Validation plots for IBS incidence and prevalence 127
Non-modelled mortality 128
Cambridge Multimorbidity Score (CMS) Validation 132
B) Appendix B: exposure modelling details and validation 133
Exposure modelling details 133
Exposure validation plots 137
Active days 138
Alcohol intake 141
Body mass index 144

Systolic blood pressure 147

Total cholesterol 150

Fruit intake 153

Vegetable intake 155

Fruit and Vegetable intake combined 157

Statin prescriptions 158

Number of cigarettes smoked (ex-smokers) 160

Number of cigarettes smoked (current smokers) 162

Smoking status 164

Years of smoking (ex-smokers) 167

Years of smoking (current smokers) 169

Years since smoking cessation 171

Exposure to environmental smoking 173

1. Introduction

IMPACT$_{NCD}$ is an open-source microsimulation modelling framework for public health policy planning and decision making in non-communicable disease (NCD) prevention. The epidemiological engine of the modelling framework translates changes in the trends of disease risk factors into changes in disease incidence and case fatality and subsequent disease prevalence. The policy layer of the framework complements the epidemiological engine and translates policy changes (hypothetical or real) into changes in the trends of disease risk factors. In this study, hypothetical scenarios are modelled in the policy layer.

IMPACT$_{NCD}$ has been used extensively to model primary prevention policies nationally in England, Germany, Brazil, and the US, and locally in Liverpool. [1–11] A simplified diagram of the model structure is presented in Figure 1-1. At the core of the model is an epidemiological engine which includes age, sex, area deprivation (English Index of Multiple Deprivation (IMD)), smoking, environmental tobacco smoke exposure, alcohol consumption, fruit and vegetable consumption, physical activity, body mass index (BMI), systolic blood pressure (SBP), and total serum cholesterol, as risk factors. The diseases modelled from risk factor trends include coronary heart disease (CHD), stroke, hypertension, type 2 diabetes mellitus (T2DM), lung cancer, colorectal cancer, breast cancer, prostate cancer, chronic kidney disease (CKD), dementia, atrial fibrillation (AF), asthma, and chronic obstructive pulmonary disease (COPD), plus thirteen additional conditions that are modelled from past trends in disease incidence and mortality. Notably, diseases can be risk factors for other diseases (i.e. type 2 diabetes mellitus is a risk factor for coronary heart disease). The policy layer is dedicated to modelling either a specific public health policy formulation or a hypothetical scenario and consists of a mathematical/statistical model of the policy.

This iteration of IMPACT$_{NCD}$ uses data from the Health Survey for England (HSE) to inform trends in risk factors and linked primary care records (Clinical Practice Research Datalink (CPRD) Aurum linked to Hospital Episode Statistics (HES) inpatient, HES outpatient, and Office for National Statistics (ONS) mortality records; described in the Disease module section) to inform trends in disease incidence, prevalence, and disease-specific mortality. ONS population estimates and projections are used to inform estimates of the population size and structure and to calibrate mortality. We simulate hypothetical policy scenarios that explore the impact of reducing risk factor exposure levels within the population.

Figure 1-1 - Conceptual structure of IMPACT$_{NCD}$

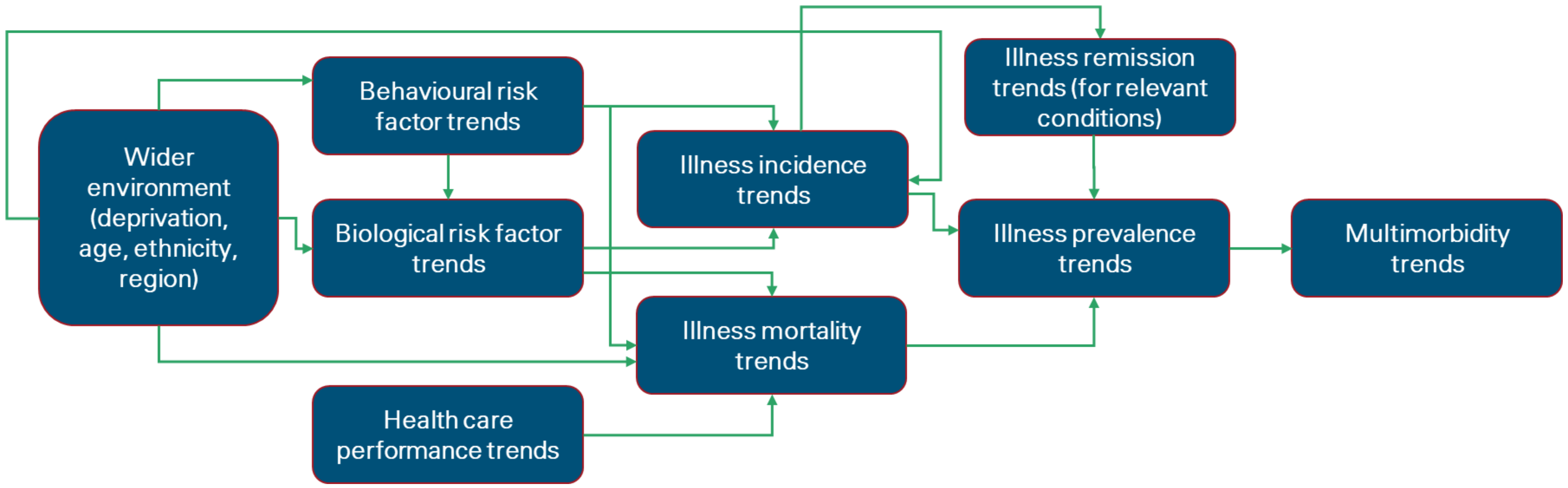

The IMPACT$_{NCD}$ framework is modular and can grow in several directions, with contributions from us and others. For example, it allows third parties to develop policy layers independently and hook them to the epidemiological engine. The open-source licence (GPLv3) ensures transparency and accountability while promoting collaborative work throughout development and deployment. The source code of the current implementation is available at https://github.com/ChristK/IMPACTncd_Engl/ .

2. Epidemiological engine

High-level description

The epidemiological engine of IMPACT$_{NCD}$ is a discrete-time, dynamic, stochastic microsimulation consisting of three modules: the **Sociodemographic** module, the **Exposure** module, and the **Disease** module.

Within the IMPACT$_{NCD}$ epidemiological engine, each unit is a synthetic individual (simulant) represented by a record containing a unique identifier and a set of associated attributes. The microsimulation then projects the life course of each synthetic individual.

The attributes of each synthetic individual include sociodemographic characteristics, exposures to risk factors, acquired diseases, and cause of death if relevant.

Specific attributes include:

1. Age, sex, ethnicity, region, and index of multiple deprivation deciles as sociodemographic exposures.
2. Alcohol intake, smoking status (current smoker/ ex-smoker/ never-smoker), smoking duration, smoking intensity, environmental tobacco exposure, fruit consumption, vegetable consumption, and physical activity as behavioural risk exposure variables.
3. Body mass index, systolic blood pressure, and total serum cholesterol as biological risk exposures.
4. The risk for thirteen diseases is modelled explicitly: asthma, atrial fibrillation, chronic kidney disease, chronic obstructive pulmonary disease, coronary heart disease, hypertension, dementia, stroke, type 2 diabetes mellitus, breast cancer, colorectal cancer, lung cancer, and prostate cancer. The risk for these conditions is modelled from relevant exposures to sociodemographic, behavioural, and biological risk factor attributes, as well as certain prevalent conditions. For instance, the risk of heart failure depends on age, sex, ethnicity, region, IMD, and prevalent CHD, COPD, T2DM and hypertension. Finally, the risk for a further 13 conditions – alcohol misuse, anxiety and depression, connective tissue disorders, constipation, epilepsy, hearing loss, heart

failure, irritable bowel syndrome (IBS), other cancers, pain, psychosis and bipolar disorders, rheumatoid arthritis, and type 1 diabetes – is directly driven by past trends in disease incidence and sociodemographic exposures. Some of these conditions are combined in post hoc analyses to derive the 20 conditions for the Cambridge Multimorbidity Score (CMS). [12] The Disease module section summarises the modelling approach, and Appendix A: Disease module details gives more detail on the modelling and data sources for each condition. Based on their risk of developing any of these conditions, simulants may develop some conditions during their life course.

5. Mortality from the diseases listed in 4 or any other cause is recorded if it occurs.

All these attributes are updated in discrete annual steps according to a set of stochastic rules. We structured these rules based on well-established epidemiological principles. Specifically, behavioural risk exposures are conditional on sociodemographic exposures; biological risk exposures are conditional on behavioural and sociodemographic exposures, and diseases are conditional on biological, behavioural, and sociodemographic exposures, as well as diagnosis of other conditions. Finally, mortality is conditional on sociodemographic, behavioural, biological and disease exposures.

The life course of synthetic individuals is simulated as many times as the number of scenarios to be modelled (sixteen for this study), using the same random numbers for all policy scenarios to reduce stochastic noise. One of the scenarios is always the 'baseline' or 'base-case' scenario with which all remaining policy scenarios are compared. In this paper, the base-case scenario is that of continuing trends in risk factors modelled from Health Survey for England 2003-2014. Comparing the disease outcomes from the life courses under the base-case scenario versus the policy scenarios generates the health impact of the policy scenarios. The output of the epidemiological engine is a dataset containing the adult life course of the simulated synthetic individuals with all the attributes mentioned above recorded annually for every scenario. From this dataset of life course trajectories, summary measures such as average CMS, mortality, disease incidence and prevalence can be calculated annually.

As we mentioned above, the epidemiological engine of IMPACT$_{NCD}$ consists of three modules: the **sociodemographic** module, the **exposure** module, and the **disease** module. In the following paragraphs, we will describe these three modules. Table 2-1 summarises the key assumptions and limitations of the IMPACT$_{NCD}$ microsimulation model.

Table 2-1 IMPACT$_{NCD}$ key assumptions and limitations

Model component	Key assumptions
**Sociodemographic module**	Migration is not modelled explicitly in the model. However, the model outputs are calibrated to ONS population projections, which include migration (we used the ONS principal migration assumptions). Nevertheless, we assume that migrants have similar characteristics to the local population.
	Social mobility is not considered.
	Decile groups of the index of multiple deprivation (DIMD) is a relative marker of (area) deprivation with several versions since 2003. We have used the 2015 version and assume it is constant throughout the simulation.
**Exposure module**	We assume that the surveys used are truly representative of the population. For example, the adjustments for selection bias in the Health Survey for England are adequate.
	On average, simulants remain in the same exposure quantile throughout their life (see Exposure module section on p7).
	The linear correlations in exposure quantiles remain constant over time (i.e. the clustering of exposures in some subpopulations) (see Exposure module section on p7).
	We assume that trends in risk factor exposures continue and follow log-linear trends.
**Disease module Continuing risk factor trends scenario (base-case)**	We assume multiplicative risk effects (see Disease incidence section on p10).
	We assume log-linear exposure-response for the continuous risk factors.
	We assume that the effects of the risk factors on incidence and case fatality are equal (see Mortality on p16).
	We assume a mean lag time between exposure and outcome of about 4-5 years for most exposure/outcome pairs, except for cancers, for which we assume a mean lag time of 9 years (see Table 2-3 and Disease incidence section on p10).
	We assume 100% risk reversibility for all exposures except smoking. We allow smoking to have a cumulative effect on the risk for COPD and lung, breast, and colorectal cancers.
	We assume that trends in disease incidence are attributable only to trends of the relevant modelled risk factors or other diseases modelled.
	We assume that the linked primary care data used to model disease trends over time represents England's adult population.
	We assume that trends in disease incidence continue to follow log-linear trends (other than pain, see below).
	For cancers, we assume that survival ten years after diagnosis equals remission.
	For all conditions other than cancer (see point above), pain, constipation, asthma, alcohol problems, and anxiety and depression, we assume conditions are chronic.

	For pain, we modelled the incidence of pain based on the incidence in 2013 due to data quality issues over time with the prescription data.
	For anxiety and depression and constipation, we did not calibrate to the observed trends in incidence rates because their projections led to implausible rates.

Sociodemographic module

The first year of every simulation in $IMPACT_{NCD}$ is 2013, so we could use the overlapping period 2013-19 to calibrate and validate the model.

For each simulation, the algorithm in the module:

1. Identifies the Lower Super Output Areas (LSOAs) that constitute the user area selection; for this study, this was set to the whole of England.
2. Draws 200,000 synthetic individuals, aged 30 to 99, from the joint age-sex distribution of the identified LSOAs. This is a default value that can be modified by the user. The joint age-sex distribution for each LSOA for 2013 is informed by the ONS population estimates. [13]
3. Assigns to each synthetic individual an IMD score based on their LSOA.
4. Probabilistically assigns to each synthetic individual an ethnicity based on their age group, sex, and LSOA. The ethnicity mixture of each LSOA is informed by the 2011 Census. [14] We include nine ethnicities in the model (white, Indian, Pakistani, Bangladeshi, other Asian, Black Caribbean, Black African, Chinese, and others).
5. Assigns to each synthetic individual a Strategic Health Authority (SHA) based on their LSOA. SHA is the smallest geographical area accessible in the Health Survey for England series and is mostly aligned with English regions. We use this in the exposure module, as we describe in the next section.

So far, the algorithm has created a synthetic population that is a snapshot of the population of England in 2013. The following steps of the algorithm create backward and forward projections of the synthetic population that are essential to model exposure time trends and time lags between exposures and diseases.

The backward projection of the synthetic population goes back to 2003; therefore, the maximum time lag we allow in the model is ten years. As everyone alive and older than 30 years old in 2013 was alive in 2003, the algorithm simply creates the back projections by

appropriately reducing the age of the synthetic individuals while keeping constant all other variables.

Similarly, for the forward projections, we project until 2043, and the algorithm increases the age of the synthetic individuals while keeping all other variables constant. For forward projections, mortality needs to be considered. We describe mortality with the disease module as disease-specific mortality is closely related to disease prevalence. $IMPACT_{NCD}$ follows an open cohort approach. Every simulated year from 2013 onwards, a new cohort of 30-year-old synthetic individuals enters the model. The same sources inform the cohort size and the joint age-sex-ethnicity distribution we described above. For example, in 2014, the new 30-year-old cohort will be informed by the population size and the joint age-sex-ethnicity distribution of those who were 29 years old in 2013. The approach may be crude; however, the final model outputs are directly standardised to ONS national or local authority population projection estimates as applicable. [15,16]

Exposure module

This module simulates the adult life course exposures of synthetic individuals based on the HSE series between 2003 and 2014. [17–28] We followed the same general principles for all simulated exposures. First, we fit an appropriate statistical model to the HSE data with the exposure of interest as the dependent variable and some functions of the year, age, sex, quintile groups of IMD (QIMD; deciles are not available in HSE), ethnicity, and SHA as independent variables. Then, we use the statistical model to predict the exposure level of every synthetic individual in the simulation based on their sociodemographic characteristics estimated from the sociodemographic module.

Including year as an independent variable in our exposure model allows us to extract the trends from the HSE series and project them into the future. Furthermore, it allows us to make backward projections of exposures when we simulate time lags. For example, for a synthetic female aged 30 in 2013, we can estimate her BMI in 2003, when she was 20 and in 2033 when she would be 50. To avoid excessively fast changes in exposure trends and to reflect our belief that decays and growths in natural phenomena are rarely linear, we included the natural logarithm of years in the statistical models assuming logarithmic trends.

We used logit ordinal regression to model exposures that were recorded as ordinal categorical variables in HSE. We used generalised additive models for location, scale, and shape (GAMLSS) for all other exposures. [29,30] These are flexible statistical models that can make all parameters of an assumed distribution for the dependent variable conditional to some function of the independent variables. For example, GAMLSS can model the mean and the standard deviation of a normally distributed dependent variable conditional on the predictors, while a linear regression only models the mean. Table B-1 summarises our modelling approach for all the exposures in the model.

Figure 2-1 – Example of risk factor assignment for the synthetic population: Plot of the systolic blood pressure quantiles of a male synthetic individual living in QIMD 3 area for ages 40 and 60

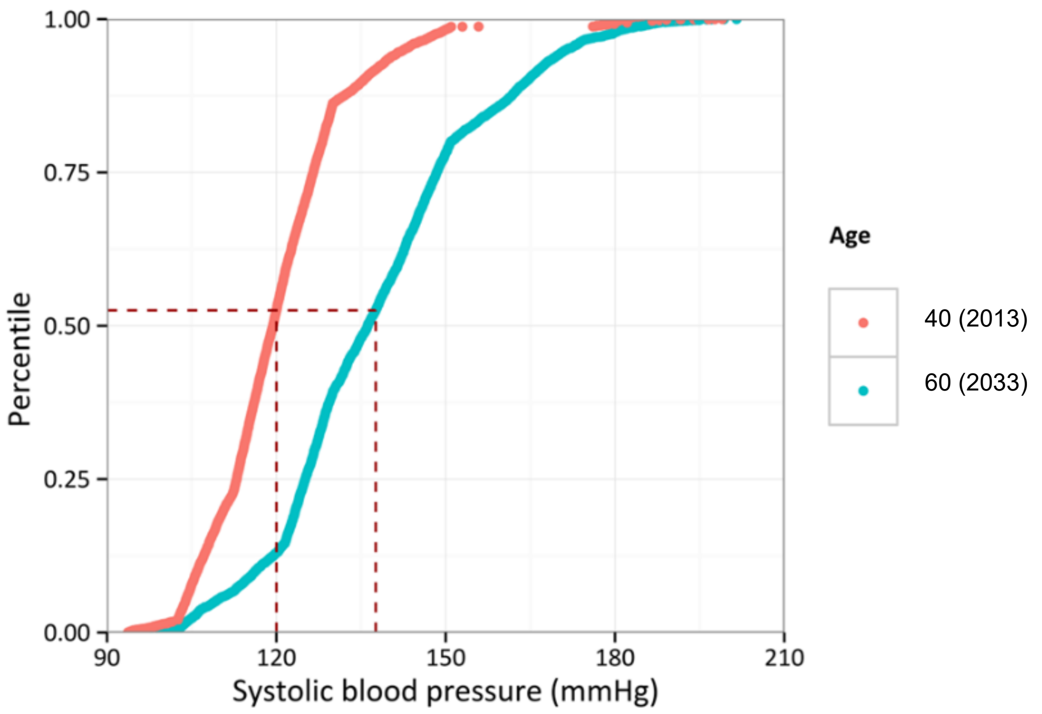

QIMD = Quintile group of the Index of Multiple Deprivation

The approach described above provides us with equations to estimate the distribution of the exposure to a risk factor for a given time and the sociodemographic characteristics of a synthetic individual. When the synthetic individual enters the simulation, a vector of random numbers between 0 and 1 and of size equal to the number of the modelled exposures is allocated to them. Each one of the numbers represents the quantile of the relevant exposure distribution. The principle is that synthetic individuals retain their quantiles throughout their life

course (this is known as the rank stability assumption). [31] For example, in 2013, a 40-year-old male synthetic individual living in a QIMD 3 area with an SBP of 120 mmHg has a SBP quantile of 0.52. Twenty years later, the same synthetic individual retained their quantile score for SBP. However, their SBP is now estimated to be 137.6 mmHg because the SBP distribution has changed to reflect the SBP of 60-year-old men living in a QIMD 3 area in 2033 (Figure 2-1). In $\text{IMPACT}_{\text{NCD}}$, we allow the quantile of the synthetic individuals to fluctuate every year using random walks to relax the rank stability assumption. But on average the large majority of synthetic individuals will have minimal changes to their exposure quantile throughout their lives.

Clustering of risk factors

Finally, exposures in individuals are correlated. For example, people with a high BMI may also have high total cholesterol and hypertension. Some of these correlations reflect strong and well-established causal mechanisms, but the cross-sectional design of HSE may bias these correlations. The method we described above captures some of these correlations by including exposures as independent variables in the statistical models for estimating exposures. For example, we included BMI as a predictor for T2DM. Going a step further, we model the full correlation structure in HSE using the following approach:

1. We used the exposure models to impute missing variables in HSE.
2. We used the quantile function of the distribution estimated by the exposure models to convert exposures in HSE to quantiles. Because the distributions were conditional on the independent variables used in each model, the quantiles are adjusted for these variables (i.e. age, sex, quintile group of IMD, etc.).
3. We estimated the linear correlation matrix of the quantiles of the exposures of interest in HSE using Pearson's correlation.
4. We used the linear correlation matrix from #3 to generate streams of uniform random numbers between 0 and 1 with a correlation structure similar to the one observed in HSE. [32]
5. We used the correlated streams of random numbers from #4 as the exposure quantiles for the synthetic individuals.

For simplicity, we assumed that the correlation structure of the exposure quantiles remains constant over time.

Disease module

The previous two modules for demographics and exposure generate a dynamic close-to-reality synthetic population composed of the adult life course exposures of each synthetic individual. The disease module then translates these exposures to disease incidence using a

population-attributable risk fraction approach (PARF). [33] We will first describe how the disease incidence is simulated in the model and then how the model simulates mortality.

We have modelled conditions in several different ways depending on the disease and the currently accepted causal associations between risk factors and diseases. The included risk factors and the modelled relationships between risk factors and disease incidence are those where sufficient, good quality data on relative risks were available and where there is sufficient evidence of a causal relationship between a risk factor and incidence of disease. Relative risks were obtained from published systematic reviews and meta-analyses supplemented with systematic literature searches. Appendix A: Disease module details includes a summary of all data sources. Furthermore, we have modelled conditions at a more granular level for some of the conditions within the Cambridge Multimorbidity Score and then aggregated them. Table 2-2 summarises the modelling approach for each condition, and further detail is given in Appendix A: Disease module details.

Disease incidence

To estimate the individualised annual probability of a synthetic individual developing a specific disease conditional on their cumulative risk exposures, we follow a 3-step approach:

Step 1. The incidence proportion attributable to each modelled risk factor by age, sex, and DIMD is estimated, assuming a specific time lag between exposure and disease. The relationships between exposures and disease incidence included in the model are outlined in Table 2-2. The time lags in the model vary stochastically between 1 and 10 years following a shifted binomial distribution. We set the mean lag time for each pair of risk exposure and disease combination according to the best possible empirical data based on the observation period of cohort studies and time to risk reversal in randomised clinical trials (see Table 2-3 and Appendix A: Disease module details). For example, the mean lag time between alcohol intake and an increase in breast cancer risk is 9 years. That means that a change in alcohol intake will take 9 years on average to be translated to a change in breast cancer risk.

Step 2. The portion of the disease incidence attributable to all the modelled risk factors is estimated and subtracted from the total incidence for 2013, assuming multiplicative risks. By multiplicative risk we mean, for example, that if the relative risk (RR) of obesity on CHD is 2 and the RR of smoking on CHD is 3, then the RR of an obese smoker is assumed to be $2 * 3 = 6$.[1]

[1] An alternative approach would be to assume additive risks. Under this assumption and using the previous example, the RR of an obese smoker would be $2 + 3 - 1 = 4$. This multiplicative risk assumption is commonly used for epidemiological modelling. [34–37]

Table 2-2: Overview of how individual conditions are modelled

CMS condition	Modelled condition	Recovery and recurrence	Causal relationships	
			Risk factors	Conditions
**Dementia**	Dementia	No recovery	Y	Y
**Cancer**	Breast cancer	Recovery after 10 years; no recurrence	Y	Y
	Colorectal cancer	Recovery after 10 years; no recurrence	Y	Y
	Lung cancer	Recovery after 10 years; no recurrence	Y	Y
	Prostate cancer	Recovery after 10 years; no recurrence	Y	N
	Other cancers	Recovery after 10 years; can recur	N	Y
**COPD**	COPD	No recovery	Y	N
**Atrial fibrillation**	Atrial fibrillation	No recovery	Y	Y
**Heart failure**	Heart failure	No recovery	N	Y
**Constipation**	Constipation	Recovery is stochastic; can recur	N	Y + past constipation
**Epilepsy**	Epilepsy	No recovery	N	Y
**Chronic pain**	Chronic pain	Recovery is stochastic; can recur	N	Y + past chronic pain
**Stroke/transient ischaemic attack**	Stroke/transient ischaemic attack	No recovery	Y	Y
**Diabetes (type I or II)**	Diabetes Type 1	No recovery	N	N
	Diabetes Type 2	No recovery	Y	N
**Alcohol problems**	Alcohol problems	Recovery is stochastic; can recur	N	Past alcohol problems
**Psychosis/ bipolar disorder**	Psychosis/bipolar disorder	No recovery	N	Y
**Chronic kidney disease**	Chronic kidney disease	No recovery	Y	N
**Anxiety and depression**	Anxiety and depression	Recovery is stochastic; can recur	N	Y + past anxiety and depression
**Coronary heart disease**	Coronary heart disease	No recovery	Y	Y
**Connective tissue disorders**	Rheumatoid arthritis	No recovery	N	N
	Other connective tissue disorders	No recovery	N	N
**Irritable bowel syndrome**	Irritable bowel syndrome	No recovery	N	N
**Asthma**	Asthma	Recovery is stochastic; can recur	Y	Past asthma
**Hearing loss**	Hearing loss	No recovery	N	N
**Hypertension**	Hypertension	No recovery	Y	N

CMS = Cambridge multimorbidity score; COPD = Chronic Obstructive Pulmonary Disease

Step 3. The probability of developing the disease is estimated for each individual in the synthetic population and is used in an independent Bernoulli trial to select those who finally develop the disease.

Table 2-3 Causal relationships included in the model between risk factors and disease incidence; the number corresponds to the time lag (in years) between exposure and outcome.

	Breast cancer	CHD	Colorectal cancer	Stroke	AF	T2DM	Asthma	CKD	Dementia	COPD	Lung cancer	Prostate cancer	Mortality from non-modelled diseases
Physical activity	9	4	9	4	-	4	-	-	-	-	-	-	5
Alcohol	9	4	9	4	4	5	-	-	-	-	-	-	5
BMI	9	4	9	4	4	5	5	5	9	-	-	-	
ETS	1	1	-	1	-	1	-	-	-	1	1	-	
Fruit	-	4	-	4	-	4	-	-	-	-	9	-	
SBP	-	4	-	4	4	-	-	4	-	-	-	-	5
Smoking	9	4	9	4	4	5	5	-	9	5	9	9	5
Cholesterol	-	4	-	4	-	-	-	-	-	-	-	-	
Vegetable	-	4	-	4	-	-	-	-	-	-	-	-	

BMI = body mass index; ETS = environmental tobacco smoke; SBP = systolic blood pressure; AF = atrial fibrillation; CHD = coronary heart disease; T2DM = type 2 diabetes mellitus; CKD = chronic kidney disease; COPD = chronic obstructive pulmonary disease

The implementation of the above method is described in more detail using CHD as an example. The same process is used for all modelled diseases except type 1 diabetes mellitus, rheumatoid arthritis, other connective tissue disorders, irritable bowel syndrome, and hearing loss, which will be described separately.

Step 1

The population-attributable risk fraction (PARF) is an epidemiological measure that estimates the proportion of the disease attributable to an associated risk factor. It depends on the relative risk associated with the risk factor and the prevalence of the risk factor in the population. In a

microsimulation context where exposures to risk factors are known at the individual level and assuming multiplicative risk factors, PARF can be estimated using the formula:

$$PARF = 1 - \frac{n}{\sum_{i=1}^{n}(RR_{i1} * RR_{i2} * \dots * RR_{ik})}$$

where n is the number of synthetic individuals in the population, and $RR_{i1 \dots ik}$ are the relative risks of the risk factors associated with CHD for each individual i. We calculated PARF based on the above formula stratified by age, sex, DIMD, ethnicity and SHA. Consistent with findings from the respective meta-analyses used for $\text{IMPACT}_{\text{NCD}}$ (Apeendix A Table A-2), on average SBP below 115 mmHg, total cholesterol below 3.8 mmol/l and BMI below 22 Kg/m^2 were considered to have a relative risk of 1.[38] Similarly, consumption of eight or more portions of fruit and vegetables and five or more active days (more than 30 minutes of moderate to vigorous activity) per week were also considered to have a relative risk of 1. All the relative risks were taken from published meta-analyses and empirical studies (for references, see Appendix A: Disease module details where we describe each disease).

Step 2

The incidence of CHD not attributable to the modelled risk factors can be estimated by the formula:

$$I_{Theoretical\, minimum} = I_{Observed} * (1 - PARF)$$

Where $I_{Observed}$ is the CHD incidence and $PARF$ is from Step 1. $I_{Theoretical\, minimum}$ represents CHD incidence if all the modelled risk factors were at optimal levels. The theoretical minimum incidence is calculated by age, sex, and QIMD only in the initial year of the simulation, and it is assumed to be stable after that.

Step 3

Assuming that $I_{Theoretical\, minimum}$ is the annual baseline probability of a synthetic individual to develop CHD for a given age, sex, DIMD, ethnicity and SHA due to risk factors not included in the model (i.e. genetics, air pollution, dietary exposures beyond fruit and vegetables etc.), the individualised annual probability of developing CHD, $\mathbb{P}(\text{CHD} \mid \text{age, sex, DIMD, ethnicity SHA, exposures})$, given their risk factors were estimated by the formula:

$$\mathbb{P}(CHD \mid age, sex, DIMD, ethnicity\, SHA\, , exposures) =$$
$$= I_{Theoretical\, minimum} * RR_{i1} * RR_{i2} * RR_{i3} * \dots * RR_{ik}$$

Where $RR_{i1 \dots ik}$ are the relative risks that are related to the specific risk exposures of the synthetic individual, the same as in step 1.

Estimating the observed incidence probability $I_{Observed}$

To estimate the observed incidence probability $I_{Observed}$ we fitted a binomial GAMLSS model to the linked primary care data (CPRD, HES, ONS) with disease incidence probability as the dependent variable and year, age, sex, DIMD, ethnicity and SHA as the independent variables. For type 1 diabetes mellitus, rheumatoid arthritis, other connective tissue disorders, irritable bowel syndrome, and hearing loss that do not depend on any risk factors, we used these models to project their future incidence, assuming log-linear trends. To account for biases in the data, we injected uncertainty of ± 5% (relative) to the estimated disease incidence probability during the simulation.

Initial prevalence

For the initial simulation year, some synthetic individuals must be allocated as prevalent cases for each modelled disease. We fitted a binomial GAMLSS model to the linked primary care data (CPRD, HES, ONS) with disease prevalence probability as the dependent variable and year, age, sex, DIMD, ethnicity and SHA as the independent variables. We used these models to allocate the initial year disease prevalence in the simulation.

Conditions with recovery and recurrence: asthma, anxiety and depression, constipation, pain, alcohol problems and cancers

Asthma, anxiety and depression, constipation, pain, and alcohol problems were treated as short-term conditions that can go into remission and reoccur. Spell duration was stochastically derived from observed durations in the CPRD data. In addition to the risk factor relationships described in Table 2-3, incident cases of these five conditions were associated with a previous history of the condition. Cancers were assumed to go into remission after 10 years, but recurrence was not modelled conditional on cancer history. All other conditions were treated as life-long.

Dependencies between conditions

As the Cambridge Multimorbidity Score (CMS) is a composite measure, it is derived from the combination of conditions an individual has. We, therefore, modelled dependencies between conditions with strong correlations based on our epidemiological understanding and the correlation structure in our linked CPRD data using GAMLSS models to derive relative risks for the effect of prevalent condition A on the incidence of condition B. Figure 2-2 displays the dependencies modelled between a prevalent condition (on the y-axis) and the subsequent incidence of another condition (on the x-axis). Disease dependencies modelled for each incident condition are summarised in Appendix A: Disease module details.

Figure 2-2 - Modelled relationships between conditions[2]

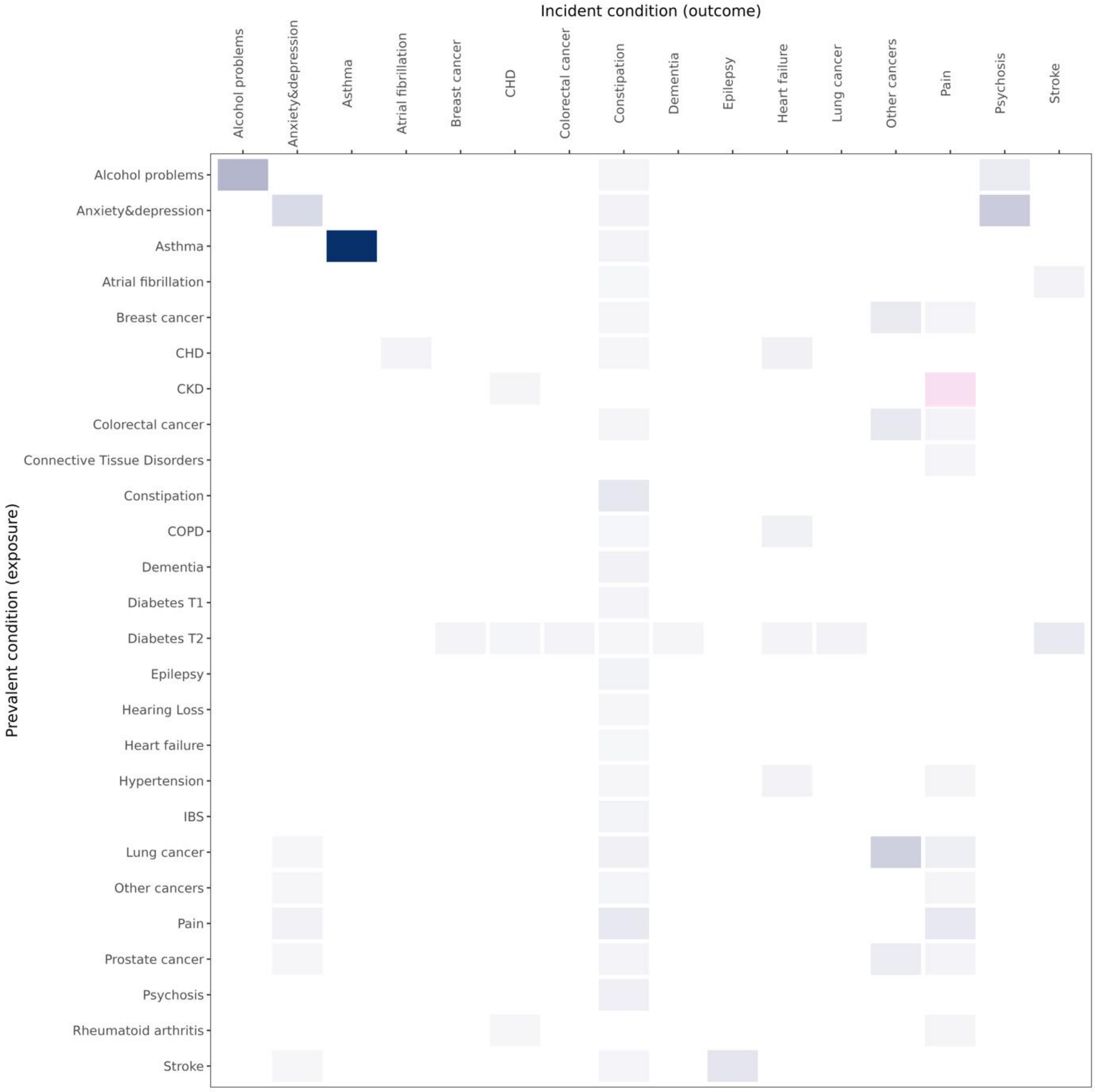

CHD = coronary heart disease; CKD = chronic kidney disease; COPD = chronic obstructive pulmonary disease; Diabetes T1 = diabetes type 1; Diabetes T2 = diabetes type 2; IBS = irritable bowel syndrome

Disease duration

For existing prevalent conditions among simulants at the start of the simulation (2013), the number of years lived with each condition is assigned based on sociodemographic characteristics and duration of the condition derived from administrative data, as derived from

[2] The darker the blue, the stronger the association. Prevalent CKD was associated with a reduced risk of pain. This is likely to be because we measured pain using prescription data in CPRD, and many pain medications are contra-indicated for CKD.

GAMLSS models fitted in the linked CPRD data. For the five conditions with remission and recurrence, these models also inform the duration of each disease occurrence.

Mortality

All synthetic individuals are exposed to the risk of dying from any of their acquired modelled conditions or any other non-modelled cause. We treat the latter as a condition that everyone is a prevalent case of. That allows us to treat it like any other condition in the model. Hearing loss, IBS, asthma, anxiety and depression, constipation, pain, hypertension, and alcohol problems have very low case fatality; hence, we assume that mortality from these conditions is 0. Furthermore, our dataset had very few CKD deaths, and we could not estimate CKD case fatality accurately. Hence, we assume that CKD mortality is 0 as well. For all other conditions, we fitted GAMLSS models to estimate their case fatality (the probability of a prevalent case of a condition dying of this condition), conditional on year, age, sex, and DIMD. For conditions with high mortality (i.e. CHD), we additionally decomposed case fatality to the first-year post-diagnosis case fatality and the second-year onwards post-diagnosis case fatality. For all potentially fatal conditions, we applied the same PARF approach described above for incidence to the case fatality. For instance, if the RR of smoking on CHD is 3, we assume that smokers have 3 times the risk of never-smokers to develop CHD and when they do, they have 3 times the risk of never-smokers to die from CHD. Therefore, we allow exposures to risk factors to influence the mortality probability of the simulants. The exception is conditions with explicit first-year case fatality, for which we assumed it is independent of exposure to risk factors and is only conditional on age, sex, and DIMD.

Additionally, using a similar approach to the one we used to model disease incidence, we allowed prevalent cases of T2DM, synthetic individuals with SBP higher than 140 mmHg, active smokers, those with one or less active day per week, and those with excessive alcohol intake to experience higher non-modelled cause mortality rates. [39]

Mortality calibration

All-cause mortality in our linked CPRD dataset was lower than the official mortality estimates that the ONS reports. This appears to be a known issue in CPRD. [40] We, therefore, calibrated the CPRD-driven mortality in the model to the ONS estimates. We first fitted functional demographic models by sex and DIMD to the ONS mortality rate estimates by single year of age from 2001 to 2019. [41,42] Using the R package 'demography', we projected all-cause mortality rates to the simulation horizon (2043). [43] Functional demographic models are generalisations of the Lee-Carter demographic model, influenced by ideas from functional data analysis and non-parametric smoothing. [44] We then inflated the case fatality rates of

potentially fatal diseases in the simulation by an age/sex/DIMD-specific calibration factor to track the projections of the functional demographic model.

3. Model outputs

The population in the model for this study was set for England. The model has a time horizon of 30 years, from 2013 to 2043. The simulation begins in 2013 to allow for validation and calibration with the linked primary care data used to inform the model. The model outputs produce life-course trajectories for each simulant, including CMS scores. From these, annual summary measures such as incidence, prevalence and mortality rates are calculated. These measures can then be compared across time and between scenarios to estimate the effects and equity of different scenarios. All outcome measures can be standardised to the 2013 European Standard Population or not, and stratification is possible by year, sex, DIMD and age group, allowing examination of how effects differ across sub-populations.

Crucially, the model is a dynamic, open-cohort microsimulation model. That means the model is trying to estimate the actual impact of the scenarios within a dynamic population where people are born, people age, people's risk factors change, and people die. The detailed modelling of the population dynamics in our model is thus different from many economic models, which are often closed-cohort, meaning they follow the same population cohort over time and often have a lifetime horizon.

Uncertainty and probabilistic sensitivity analysis

$IMPACT_{NCD}$ implements a 2nd order Monte Carlo approach to estimate uncertainty intervals (UI) for each scenario. [45,46] For each iteration, a different set of input parameters is used by sampling from the respective distributions of input parameters. We assumed log-normal distributions for relative risks and hazard ratios, normal distributions for coefficients of linear regression equations, and uniform distributions for estimates of incidence, prevalence, and case fatality rates. Specifically, for relative risks and hazard ratios, the distributions were bounded above 1 when the mean was above 1 and vice versa.

$IMPACT_{NCD}$ allows stochastic uncertainty, parameter uncertainty, individual heterogeneity, and to some extent, structural uncertainty to be propagated in the reported UI. In $IMPACT_{NCD}$, we minimise stochastic uncertainty by using the same random numbers for all scenarios when appropriate. The following example illustrates the different types of uncertainty considered in the model. Let us assume that the annual risk for CHD is 5%. Suppose we apply this risk to all individuals and randomly draw from a Bernoulli distribution with p = 5% to select those who

will manifest CHD. In that case, we only consider stochastic uncertainty. Our model minimises stochastic uncertainty when we compare different policy scenarios by using the same random numbers for all scenarios, where appropriate. If we allow the annual risk for CHD to be conditional on individual characteristics (i.e. age, sex, exposure to risk factors), then individual heterogeneity is considered. Finally, when the uncertainty of the relative risks due to sampling errors is considered in estimating the annual risk for CHD, the parameter uncertainty is considered. From these three types of uncertainty, only the parameter uncertainty could be reduced by better studies in the future.

The structure of the model is grounded in fundamental epidemiological ideas and well-established causal pathways on which exposures are causally related to the specific NCDs which are explicitly modelled. For example, hypertension is causally related to CVD but not lung cancer. Hence, structural sensitivity analysis is not necessary to explore the possibility of hypertension being a risk factor for lung cancer. Therefore, we considered this type of uncertainty relatively small and did not study it in detail, with one exception: the discrete-time bias that arises from the fact that time in $IMPACT_{NCD}$ is not continuous. A synthetic individual within the model may die of multiple causes within one year; however, the discrete-time nature of the simulation does not allow the identification of the cause that 'killed' the simulant first. Every time this happens to a simulant, we randomly select a cause of death from the list of all the terminal events that occurred for the simulant that year. Hence, we propagate discrete-time uncertainty to the output.

4. Validation and calibration

We validated the $IMPACT_{NCD}$ epidemiological engine using internal validation plotting the modelled exposures' prevalence and disease incidence against the observed exposures' prevalence and disease incidence in HSE and linked primary care data, respectively. Mortality in the model is calibrated to mortality projections as described above (Mortality). We present the relevant validation plots for modelled exposures in Appendix B: exposure modelling details and validation stratified by year and age group and by quintiles of IMD and age group. Figure 4-1 shows the modelled prevalence of CMS > 1.5 (defined as 'major illness' in this paper) against the observed CPRD Aurum prevalence. The decline from 2017 in the observed data is an artefact caused by the case definition and consequent prevalence of pain. Validation plots (age-sex-dimd standardised) for incidence, case fatality, and prevalence of individual conditions are shown in Appendix A: Disease module details. Additionally, we have produced and inspected plots for multiple combinations of stratification levels that are available on

request. Overall, the plots suggest that $IMPACT_{NCD}$ captures exposure trends and translates them to disease incidence and mortality reasonably well for the purpose of this project.

Figure 4-1 - Validation plot for the prevalence of major illness* (age-sex-dimd standardised)

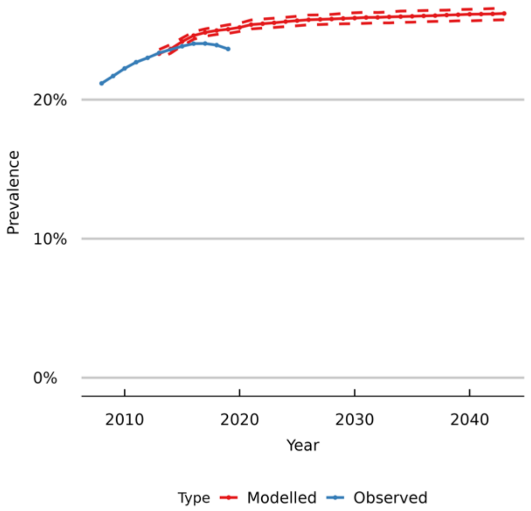

*Major illness is defined as a Cambridge Multimorbidity Score > 1.5

5. Definition of the Cambridge Multimorbidity Score and defining conditions in primary care data

The Cambridge Multimorbidity Score (CMS) is used to define levels of ill health for an individual and for the population. [12] The CMS assigns a weight or 'score' to 20 conditions on the basis of how the illness affects their use of primary care, emergency health services and the patient's likelihood of death (see

Table *5-1* below for the exact weight of each condition).

Our projections draw on detailed patient-level administrative data linked to primary care (CPRD Aurum) and secondary care (Hospital Episode Statistics) from a sample of 1.7 million patients. The data include chronic conditions diagnosed in primary care and the Index of Multiple Deprivation (IMD) score for each patient's local area, age, sex, ethnicity and region. Since our analysis relies on diagnosis data from patient records, all our projections refer to *diagnosed* prevalence, incidence and illness. Due to our focus on the long-term trends in ageing and health and due to our data sample being available up to 2019/20, we do not include COVID-19 and its impacts in our analysis.

The use of diagnosed conditions in administrative primary care data to measure levels of illness has several advantages over using survey data. Administrative data tend to have larger and more representative patient samples and are less subjective than self-reported measures of health in surveys. [47] It is, however, important to note that rates of diagnosis can be impacted by patients' access to care and changes in diagnosis policy and practices over time. Several studies have found underdiagnosis in COPD, [48] hypertension, [49] type 2 diabetes, [50] and dementia, [51] so it is likely that the true burden of illness in the baseline scenario will be higher than what we estimate in this study.

The CMS is used to define levels of ill health for an individual and for the population. [12] The score provides a metric which allows comparisons of levels of illness across conditions and over time. The CMS assigns a weight or 'score' to 20 conditions based on how the illness affects their use of primary care, emergency health services and the patient's likelihood of death (

Table *5-1*). For instance, cancer and heart failure are given higher scores than hypertension (high blood pressure) or hearing loss because they are more likely to lead to death, unplanned hospital admissions or greater primary care needs. For those with multimorbidity, scores are added together, meaning individuals with the same score can have a different number and combination of conditions.

We use the CMS rather than other multimorbidity indices such as Charlson [52] and Elixhauser, [53] as it is more representative of conditions that are highly prevalent today and because it outperforms the Charlson index in predicting primary care consultations and hospital admissions and, to a lesser extent, mortality. [12]

Of the 20 conditions in our analysis, most are considered "permanent" because there is no cure: once someone is diagnosed with a condition, like atrial fibrillation, they will forever have that condition. For six of the conditions observed to be more transient in the data, we allow patients to go into remission: cancer, asthma, anxiety and depression, alcohol problems, constipation and chronic pain.

The code lists used for the CMS, available online, [54] have been developed using CPRD GOLD based on read codes, medcodes and prodcodes. In this study, we use data from CPRD Aurum.

Table 5-1 Cambridge Multimorbidity Score conditions and their weights

Condition	Primary care consultations[1]	Unplanned admissions[2]	Mortality[2]	General outcome[3]
**Dementia**	1.81	156.9	124.42	**2.50**
**Cancer**	2.58	104.8	62	**1.53**
**COPD**	3.43	134.51	42.5	**1.46**
**Atrial fibrillation**	5.94	105.21	22.14	**1.34**
**Heart failure**	2.9	73.2	43.47	**1.18**
**Constipation**	3.42	72.73	35.42	**1.12**
**Chronic pain**	3.43	84.93	16.46	**0.92**
**Epilepsy**	2.13	113.42	18.26	**0.92**
**Stroke/transient ischaemic attack (TIA)**	1.54	90.84	20.63	**0.80**
**Diabetes (type 1 or 2)**	3.77	55.33	10.23	**0.75**
**Alcohol problems**	0.97	93.59	12.72	**0.65**
**Psychosis/bipolar disorder**	2.24	77.28	7.2	**0.64**
**Chronic kidney disease**	0.98	52.13	16.61	**0.53**
**Anxiety/depression**	2.12	46.61	7.04	**0.50**
**Coronary heart disease**	1.49	70.87	4.22	**0.49**
**Connective tissue disorders**	3.1	28.87	−0.39	**0.43**
**Irritable bowel syndrome**	1.82	8.55	−1.33	**0.21**
**Asthma**	1.32	22.78	−2.73	**0.19**
**Hearing loss**	1.04	8.93	−3.94	**0.09**
**Hypertension**	0.66	10.76	−2.09	**0.08**

Note: 1. Per person-year. 2. Per 1000 person-years. 3. Unit change associated with a change of 1 standard deviation in each of the 3 outcomes. Negative weights can be interpreted as reflecting a negative association with the outcome of interest after controlling for other conditions. Constipation, when diagnosed in primary care data, is likely linked to the side effects of taking opioid analgesics (strong pain killers such as codeine phosphate) and other medication. Chronic pain that leads to opioid prescribing tends to be worse, and therefore constipation could be linked with higher care needs.
COPD = Chronic obstructive pulmonary disease

Table 5-2 summarises the definitions we have used for each condition and in the original CMS paper. We use code lists for CPRD developed by Anna Head, available on GitHub [55] and adapted from code lists from the CALIBER algorithms[3]. [56]

[3] Available here https://www.caliberresearch.org/portal/phenotypes/chronological-map and in machine-readable format here https://github.com/spiros/chronological-map-phenotypes.

Table 5-3 and Figure 5-1 summarise the sample inclusion and exclusion criteria along with the cleaning rules applied to the initial 2 million random sample of patients from CPRD Aurum.

Table 5-2 Definition of the conditions in the Cambridge Multimorbidity Score

Condition	In this microsimulation model
**Alcohol problems**	At least one code recorded (in CPRD Aurum or HES) in the last 12 months. Remission after 12 months without new diagnosis. The code list for alcohol misuse excludes 'alcoholic liver disease'.
**Asthma**	At least one code recorded (in CPRD Aurum or HES) in the last 12 months. Remission after 12 months without new diagnosis.
**Anxiety/depression**	At least one code recorded for anxiety OR depression in the last 12 months (in CPRD Aurum or HES). Remission after 12 months without new diagnosis.
**Atrial fibrillation**	At least one code ever recorded (in CPRD Aurum or HES).
**All cancers**	At least one code recorded (in CPRD Aurum or HES) for either bowel, lung, prostate or any other cancer. Remission after 10 years since the first diagnosis of the last cancer, if more than one.
**Chronic kidney disease**	Based on at least two records (in CPRD Aurum) of test values (eGFR) < 60ml/min, corresponding to a diagnosis of CKD of stage 3 or above. The two records must be at least within a 90-day interval.<br>OR<br>At least one CKD observation code ever recorded (in CPRD Aurum or HES)
**Chronic pain**	At least four prescriptions (in CPRD Aurum) for painkiller drugs in the span of a year (analgesics or epilepsy drugs if not diagnosed with epilepsy). Remission after 12 months without new prescriptions.
**Connective tissue disorder/rheumatoid arthritis**	At least one code ever recorded for connective tissue disorder OR rheumatoid arthritis (in CPRD Aurum or HES).
**Constipation**	Four or more laxative prescriptions in last 12 months (in CPRD Aurum). Remission after 12 months without new prescriptions.
**Chronic obstructive pulmonary disease**	At least one code ever recorded (in CPRD Aurum or HES).
**Coronary heart disease**	At least one code ever recorded (in CPRD Aurum or HES).
**Dementia**	At least one code ever recorded (in CPRD Aurum or HES).
**Diabetes (any type)**	At least one code ever recorded for diabetes type 2 OR other type of diabetes (in CPRD Aurum or HES).
**Epilepsy**	At least one code ever recorded (in CPRD Aurum or HES).
**Hearing loss**	At least one code ever recorded (in CPRD Aurum or HES).
**Heart failure**	At least one code ever recorded (in CPRD Aurum or HES).
**Hypertension**	At least one code ever recorded (in CPRD Aurum or HES).
**Irritable bowel syndrome (IBS)**	At least one code ever recorded (in CPRD Aurum or HES).
**Psychosis**	At least one code ever recorded (in CPRD Aurum or HES).
**Stroke/Transient ischaemic attack (TIA)**	At least one code ever recorded for stroke OR TIA (in CPRD Aurum or HES).

Table 5-3 Sample exclusions and cleaning rules

Type	Rule
**Sample inclusion/ exclusion**	Exclude duplicate CPRD patient IDs, keep the most recent one*
	Exclude if not eligible (linkable) for all 5 datasets (CPRD, ONS, HES, cancer registry, mental health) (linkage eligibility file)**
	Exclude if entered the study after 31 March 2020 (as the data contain no HES records after 31st October 2020)
	Exclude implausible linkage**, ie. different number of linkages to HES and ONS registry data
	Exclude implausible linkage, ie. end of registration or death before patient's start of registration with the practice (*regstartdate)*
	Exclude patients who are active in the sample for less than one year**, unless they are <1 year old (to be used to calculate infant mortality rates)
	Exclude if missing IMD (deprivation) and missing ethnicity
	Use ONS' date of death (*dod*) if recorded; otherwise, use date of death recorded in CPRD (*emis_ddate)* or estimated by CPRD (*cprd_ddate)* if ONS date of death is missing and if the CPRD date of death is between 1 Nov 2019 - 31 Oct 2020 (i.e. one year before end of sample eligibility) to account for ONS delays in transmitting death information.
**Cleaning of patient variables**	Use earliest of: CPRD registration end date (*regenddate* in patient file), date of death (ONS file's date of death (*dod*) if available, or *cprd_ddate* (CPRD patient file) if between 1 Nov 2019 and 31 Oct 2020), practice last collection date (*lcd* in practice file), 31 October 2020 (HES data not available after this)
	Use most common ethnicity recorded across all sources (CPRD, HES inpatient, outpatient, A&E); unless it is 'Unknown', use non- unknown record of ethnicity. If tie in most common ethnicity, use the most recently recorded.
	Where date of observation (*obsdate*) is recorded, use *obsdate*; use the date the event was entered into the practice system (*enterdate*) otherwise.
	If date of observation (*obsdate*) is before 1900 or after 2021, and the date the event was recorded (*enterdate*) is between 1900-2021, use *enterdate*; if *enterdate* is outside of 1900-2021, we drop the observation[4].
	If date of observation (*obsdate*) is before patient's year of birth, but the date the event was recorded (*enterdate*) is after, we use the latter. If both dates are before year of birth, we drop the observation.
	Remove observations of type 'family history'
	If missing date of the start of the hospital episode (*epistart* in HES files), we replace it with date of the end of the hospital episode (*epiend*).
	If missing date of the procedure (*evdate*), we replace it with date of the start of the hospital episode (*epistart*).
	Recode as prevalence if a new diagnosis takes place within a year of registration with a new practice.
**Prevalence modelling**	For resolving conditions, if the date of a new diagnosis is within 365 days of the remission date of the previous disease spell, we recode it to be part of the same disease episode.

Note: * this is identified by the highest CPRD patient ID number; ** these are cleaning rules commonly implemented when using CPRD (e.g. see (1)).

[4] This is because a diagnosis will likely be picked up in other primary or secondary care activity while inferring diagnosis dates wrongly will introduce errors in our age-specific prevalence.

Figure 5-1 Flowchart for the sample exclusions

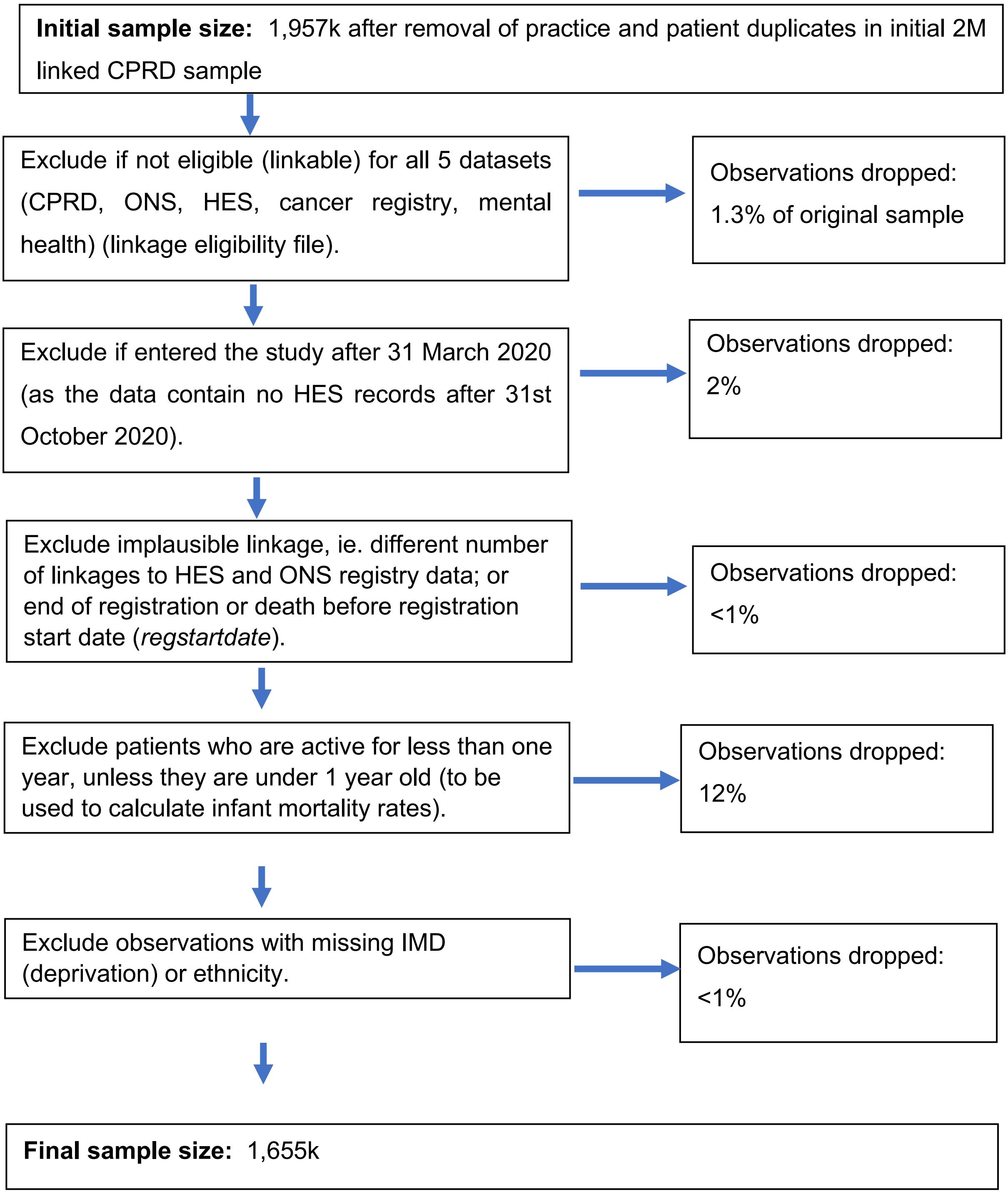

6. Strengths and limitations of this modelling approach

The IMPACT$_{NCD}$ model is an advanced, validated, flexible microsimulation of the dynamics of NCDs in a population, including important NCDs that are amenable to prevention and provide support to a range of capabilities to conduct state-of-the-art effectiveness and equity analysis.

The complex dynamic generated by reductions in risk factors and disease trends needs to be modelled for a more realistic estimate of the future burden of illness from a societal perspective, which includes competing causes of illness and death. For instance, the model factors in that if CHD incidence is delayed, people may live longer and be more likely to get any of the other modelled diseases. This was evident in our projections, where, for example, the number of COPD cases increased because of people living longer. The modelling of individual conditions allows in-depth exploration of how trends in risk factors may impact the future burden of multimorbidity.

All such modelling analyses have limitations. This iteration of the model uses exposure data from HSE waves up to 2014; however, we know that there have been changes in more recent years to both overall trends and socioeconomic inequalities. Furthermore, the data that informs our model is from before the coronavirus pandemic and therefore does not capture any of the impacts of the coronavirus or pandemic-related restrictions. The use of HSE data from 2003-2014 was based on several reasons. Most importantly, during this time period there is consistent recording of risk factors based on age, decile of IMD, ethnicity and geographic region over time. This allows us to separately model the projected incidence of long-term illness for different population subgroups. Although later versions of HSE are available, differences in survey methodology meant it was not possible to include them in this model. As we have used data from HSE for the period between 2003-2014, some trends in risk factors differ from those reported by OHID and PHE Fingertips for the period between 2014-present day.

We derived trends in disease incidence, prevalence, and mortality, from CPRD data (GP practices) linked to HES (secondary care data) and ONS (cause of death) records. CPRD and HES data only provide information from where individuals have accessed healthcare and diagnostic information has been recorded. Whilst CPRD data is collated directly from GP records, HES data is an administrative summary of each episode of care. As such, our analyses measure only diagnosed cases of conditions and do not capture unmet needs. Whilst this linked primary care data largely represents the population of England registered with primary care providers, it is slightly less representative compared to the overall population of England: marginalised groups and those from the most deprived areas are underrepresented.

We have modelled dependencies between certain diseases based on the strength of the correlations and clinical understanding. In future iterations of the model, we plan to improve the modelling of disease clusters by including additional disease-disease interactions.

7. References

1. Collins B, Kypridemos C, Cookson R, Parvulescu P, McHale P, Guzman-Castillo M, et al. Universal or targeted cardiovascular screening? Modelling study using a sector-specific distributional cost effectiveness analysis. Prev. Med. 2020;130:105879.

2. Collins B, Kypridemos C, Pearson-Stuttard J, Huang Y, Bandosz P, Wilde P, et al. FDA Sodium Reduction Targets and the Food Industry: Are There Incentives to Reformulate? Microsimulation Cost-Effectiveness Analysis. Milbank Q. 2019;97:858–80.

3. Huang Y, Kypridemos C, Liu J, Lee Y, Pearson-Stuttard J, Collins B, et al. Cost-Effectiveness of the US Food and Drug Administration Added Sugar Labeling Policy for Improving Diet and Health. Circulation 2019;139:2613–24.

4. Kypridemos C, Allen K, Hickey GL, Guzman-Castillo M, Bandosz P, Buchan I, et al. Cardiovascular screening to reduce the burden from cardiovascular disease: microsimulation study to quantify policy options. BMJ 2016;353:i2793.

5. Kypridemos C, Collins B, McHale P, Bromley H, Parvulescu P, Capewell S, et al. Future cost-effectiveness and equity of the NHS Health Check cardiovascular disease prevention programme: Microsimulation modelling using data from Liverpool, UK. PLoS Med. 2018;15:e1002573.

6. Kypridemos C, Guzman-Castillo M, Hyseni L, Hickey GL, Bandosz P, Buchan I, et al. Estimated reductions in cardiovascular and gastric cancer disease burden through salt policies in England: an IMPACTNCD microsimulation study. BMJ Open 2017;7:e013791.

7. Laverty AA, Kypridemos C, Seferidi P, Vamos EP, Pearson-Stuttard J, Collins B, et al. Quantifying the impact of the Public Health Responsibility Deal on salt intake, cardiovascular disease and gastric cancer burdens: interrupted time series and microsimulation study. J. Epidemiol. Community Health 2019;73:881–7.

8. Nilson EAF, Pearson-Stuttard J, Collins B, Guzman-Castillo M, Capewell S, O'Flaherty M, et al. Estimating the health and economic effects of the voluntary sodium reduction targets in Brazil: microsimulation analysis. BMC Med. 2021;19:225.

9. O'Flaherty M, Lloyd-Williams F, Capewell S, Boland A, Maden M, Collins B, et al. Modelling tool to support decision-making in the NHS Health Check programme: workshops, systematic review and co-production with users. Health Technol. Assess. Winch. Engl. 2021;25:1–234.

10. Pearson-Stuttard J, Kypridemos C, Collins B, Mozaffarian D, Huang Y, Bandosz P, et al. Estimating the health and economic effects of the proposed US Food and Drug Administration voluntary sodium reformulation: Microsimulation cost-effectiveness analysis. PLoS Med. 2018;15:e1002551.

11. Emmert-Fees KMF, Amies-Cull B, Wawro N, Linseisen J, Staudigel M, Peters A, et al. Projected health and economic impacts of sugar-sweetened beverage taxation in Germany: A cross-validation modelling study. PLOS Med. 2023;20:e1004311.

12. Payne RA, Mendonca SC, Elliott MN, Saunders CL, Edwards DA, Marshall M, et al. Development and validation of the Cambridge Multimorbidity Score. CMAJ Can. Med. Assoc. J. [Internet] 2020 [cited 2022 Jan 5];192:E107–14. Available from: https://www.ncbi.nlm.nih.gov/pmc/articles/PMC7004217/

13. Office for National Statistics. Lower layer Super Output Area population estimates (supporting information) - Office for National Statistics [Internet]. [cited 2018 Dec 14];Available from: https://www.ons.gov.uk/peoplepopulationandcommunity/populationandmigration/populationestimates/datasets/lowersuperoutputareamidyearpopulationestimates

14. Office for National Statistics. LC2101EW (Ethnic group by sex by age) - Nomis - Official Labour Market Statistics [Internet]. 2013 [cited 2020 Feb 11];Available from: https://www.nomisweb.co.uk/census/2011/lc2101ew

15. Office for National Statistics. Population projections – local authorities: SNPP Z1 - Office for National Statistics [Internet]. 2018 [cited 2018 Dec 13];Available from: https://www.ons.gov.uk/peoplepopulationandcommunity/populationandmigration/populationprojections/datasets/localauthoritiesinenglandz1

16. Office for National Statistics. National population projections [Internet]. 2014 [cited 2014 Mar 13];Available from: http://www.ons.gov.uk/ons/publications/all-releases.html?definition=tcm%3A77-21600

17. National Centre for Social Research, University College London. Department of Epidemiology and Public Health. Health Survey for England, 2003 [computer file]. 2nd Edition. Colchester, Essex: UK Data Archive [distributor] [Internet]. 2010 [cited 2014 May 1];Available from: http://dx.doi.org/10.5255/UKDA-SN-5098-1

18. National Centre for Social Research, University College London. Department of Epidemiology and Public Health. Health Survey for England, 2004 [computer file]. 2nd Edition. Colchester, Essex: UK Data Archive [distributor] [Internet]. 2010 [cited 2014 May 1];Available from: http://dx.doi.org/10.5255/UKDA-SN-5439-1

19. National Centre for Social Research, University College London. Department of Epidemiology and Public Health. Health Survey for England, 2005 [computer file]. 3rd Edition. Colchester, Essex: UK Data Archive [distributor] [Internet]. 2011 [cited 2014 May 1];Available from: http://dx.doi.org/10.5255/UKDA-SN-5675-1

20. National Centre for Social Research, University College London. Department of Epidemiology and Public Health. Health Survey for England, 2006 [computer file]. 4th Edition. Colchester, Essex: UK Data Archive [distributor] [Internet]. 2011 [cited 2014 May 1];Available from: http://dx.doi.org/10.5255/UKDA-SN-5809-1

21. National Centre for Social Research, University College London. Department of Epidemiology and Public Health. Health Survey for England, 2007 [computer file]. 2nd Edition. Colchester, Essex: UK Data Archive [distributor] [Internet]. 2010 [cited 2014 May 1];Available from: http://dx.doi.org/10.5255/UKDA-SN-6112-1

22. National Centre for Social Research, University College London. Department of Epidemiology and Public Health. Health Survey for England, 2008 [computer file]. 4th Edition. Colchester, Essex: UK Data Archive [distributor] [Internet]. 2013 [cited 2014 May 1];Available from: http://dx.doi.org/10.5255/UKDA-SN-6397-2

23. National Centre for Social Research, University College London. Department of Epidemiology and Public Health. Health Survey for England, 2009 [computer file]. 2nd Edition. Colchester, Essex: UK Data Archive [distributor] [Internet]. 2011 [cited 2014 May 1];Available from: http://dx.doi.org/10.5255/UKDA-SN-6732-1

24. NatCen Social Research, Royal Free, University College London. Department of Epidemiology and Public Health. Health Survey for England, 2010 [computer file]. 2nd Edition. Colchester, Essex: UK Data Archive [distributor] [Internet]. 2012 [cited 2014 May 1];Available from: http://dx.doi.org/10.5255/UKDA-SN-6986-2

25. NatCen Social Research, University College London. Department of Epidemiology and Public Health. Health Survey for England, 2011 [computer file]. Colchester, Essex: UK Data Archive [distributor] [Internet]. 2013 [cited 2014 May 1];Available from: http://dx.doi.org/10.5255/UKDA-SN-7260-1

26. NatCen Social Research, University College London. Department of Epidemiology and Public Health. Health Survey for England, 2012 [computer file]. Colchester, Essex: UK Data Archive [distributor] [Internet]. 2014 [cited 2014 May 1];Available from: http://dx.doi.org/10.5255/UKDA-SN-7480-1

27. NatCen Social Research, University College London. Department of Epidemiology and Public Health. Health Survey for England, 2013 [computer file]. Colchester, Essex: UK Data Archive [distributor] [Internet]. 2015 [cited 2016 May 1];Available from: http://dx.doi.org/10.5255/UKDA-SN-7649-1

28. NatCen Social Research, University College London. Department of Epidemiology and Public Health. Health Survey for England, 2014 [computer file]. Colchester, Essex: UK Data Archive [distributor] [Internet]. 2016 [cited 2016 May 1];Available from: http://dx.doi.org/10.5255/UKDA-SN-7919-1

29. Stasinopoulos MD, Rigby RA, Heller GZ, Voudouris V, De Bastiani F. Flexible regression and smoothing: using GAMLSS in R. Boca Raton: CRC Press/Taylor & Francis Group; 2017.

30. Rigby RA, Stasinopoulos MD, Heller GZ, De Bastiani F. Distributions for Modelling Location, Scale, and Shape Using GAMLSS in R. [Internet]. New York: CRC Press LLC; 2019 [cited 2019 Nov 7]. Available from: http://public.ebookcentral.proquest.com/choice/publicfullrecord.aspx?p=5909942

31. Suen SC, Goldhaber-Fiebert JD, Basu S. Matching Microsimulation Risk Factor Correlations to Cross-sectional Data: The Shortest Distance Method. Med. Decis. Mak. Int. J. Soc. Med. Decis. Mak. 2018;38:452–64.

32. Embrechts P, Lindskog F, Mcneil A. Modelling Dependence with Copulas and Applications to Risk Management [Internet]. In: Handbook of Heavy Tailed Distributions in Finance. Elsevier; 2003 [cited 2020 Feb 13]. page 329–84.Available from: https://linkinghub.elsevier.com/retrieve/pii/B9780444508966500108

33. Levin ML. The occurrence of lung cancer in man. Acta - Unio Int. Contra Cancrum 1953;9:531–41.

34. Andersson T, Alfredsson L, Källberg H, Zdravkovic S, Ahlbom A. Calculating measures of biological interaction. Eur. J. Epidemiol. [Internet] 2005 [cited 2023 May 25];20:575–9. Available from: https://doi.org/10.1007/s10654-005-7835-x

35. Mehta N, Preston S. Are Major Behavioral and Sociodemographic Risk Factors for Mortality Additive or Multiplicative in Their Effects? Soc. Sci. Med. 1982 [Internet] 2016 [cited 2023 May 25];154:93–9. Available from: https://www.ncbi.nlm.nih.gov/pmc/articles/PMC5599176/

36. Comparative quantification of health risks [Internet]. Geneva: World Health Organisation; 2004 [cited 2014 Jan 30]. Available from: https://www.who.int/publications/i/item/9241580313

37. Murray CJL, Aravkin AY, Zheng P, Abbafati C, Abbas KM, Abbasi-Kangevari M, et al. Global burden of 87 risk factors in 204 countries and territories, 1990–2019: a systematic analysis for the Global Burden of Disease Study 2019. The Lancet [Internet] 2020 [cited 2020 Oct 19];396:1223–49. Available from: https://www.thelancet.com/journals/lancet/article/PIIS0140-6736(20)30752-2/abstract

38. Singh GM, Danaei G, Farzadfar F, Stevens GA, Woodward M, Wormser D, et al. The age-specific quantitative effects of metabolic risk factors on cardiovascular diseases and diabetes: a pooled analysis. PLOS ONE [Internet] 2013 [cited 2016 Dec 13];8:e65174. Available from: http://journals.plos.org/plosone/article?id=10.1371/journal.pone.0065174

39. Stringhini S, Carmeli C, Jokela M, Avendaño M, Muennig P, Guida F, et al. Socioeconomic status and the 25 × 25 risk factors as determinants of premature mortality: a multicohort study and meta-analysis of 1·7 million men and women. Lancet [Internet] 2017 [cited 2017 Feb 3];389:1229–37. Available from: http://www.thelancet.com/journals/lancet/article/PIIS0140-6736(16)32380-7/abstract

40. Schmidt JCF, Lambert PC, Gillies CL, Sweeting MJ. Patterns of rates of mortality in the Clinical Practice Research Datalink. PLOS ONE [Internet] 2022 [cited 2023 Mar 7];17:e0265709. Available from: https://dx.plos.org/10.1371/journal.pone.0265709

41. Number of deaths and populations in deprivation decile areas by sex and single year of age, England and Wales, registered years 2019 - Office for National Statistics [Internet]. [cited 2022 Feb 18];Available from: https://www.ons.gov.uk/peoplepopulationandcommunity/birthsdeathsandmarriages/deaths/adhocs/12413deathregistrationsandpopulationsbyindexofmultipledeprivationimddecileenglandandwales2019

42. Number of deaths and populations in deprivation decile areas by sex and single year of age, England and Wales, registered years 2001 to 2018 - Office for National Statistics [Internet]. [cited 2022 Feb 18];Available from: https://www.ons.gov.uk/peoplepopulationandcommunity/birthsdeathsandmarriages/deaths/adhocs/11169deathregistrationsandpopulationsbyindexofmultipledeprivationengland2001to2018

43. Hyndman RJ. demography: Forecasting mortality, fertility, migration and population data [Internet]. 2017;Available from: http://CRAN.R-project.org/package=demography

44. Hyndman RJ, Shahid Ullah Md. Robust forecasting of mortality and fertility rates: A functional data approach. Comput. Stat. Data Anal. [Internet] 2007 [cited 2014 Oct 28];51:4942–56. Available from: http://www.sciencedirect.com/science/article/pii/S0167947306002453

45. Koerkamp BG, Stijnen T, Weinstein MC, Hunink MGM. The combined analysis of uncertainty and patient heterogeneity in medical decision models. Med. Decis. Making

[Internet] 2011 [cited 2015 Mar 18];31:650–61. Available from: http://mdm.sagepub.com/content/31/4/650

46. Briggs AH, Weinstein MC, Fenwick EAL, Karnon J, Sculpher MJ, Paltiel AD. Model parameter estimation and uncertainty: a report of the ISPOR-SMDM modeling good research practices Task Force-6. Value Health [Internet] 2012 [cited 2013 Oct 7];15:835–42. Available from: http://www.sciencedirect.com/science/article/pii/S1098301512016592

47. How accurate are self-reported diagnoses? Comparing self-reported health events in the English Longitudinal Study of Ageing with administrative hospital records [Internet]. Inst. Fisc. Stud. [cited 2023 May 9];Available from: https://ifs.org.uk/publications/how-accurate-are-self-reported-diagnoses-comparing-self-reported-health-events-english

48. Nacul L, Soljak M, Samarasundera E, Hopkinson NS, Lacerda E, Indulkar T, et al. COPD in England: a comparison of expected, model-based prevalence and observed prevalence from general practice data. J. Public Health 2011;33:108–16.

49. Petersen J, Benzeval M. Untreated hypertension in the UK household population — Who are missed by the general health checks? Prev. Med. Rep. 2016;4:81–6.

50. Whicher CA, O'Neill S, Holt RIG. Diabetes in the UK: 2019. Diabet. Med. 2020;37:242–7.

51. Ford E, Rooney P, Oliver S, Hoile R, Hurley P, Banerjee S, et al. Identifying undetected dementia in UK primary care patients: a retrospective case-control study comparing machine-learning and standard epidemiological approaches. BMC Med. Inform. Decis. Mak. 2019;19:248.

52. Charlson ME, Pompei P, Ales KL, MacKenzie CR. A new method of classifying prognostic comorbidity in longitudinal studies: Development and validation. J. Chronic Dis. 1987;40:373–83.

53. Elixhauser A, Steiner C, Harris DR, Coffey RM. Comorbidity Measures for Use with Administrative Data. Med. Care 1998;36:8.

54. CPRD @ Cambridge. Code Lists - Primary Care Unit [Internet]. 2018 [cited 2022 Mar 16];Available from: https://www.phpc.cam.ac.uk/pcu/research/research-groups/crmh/cprd_cam/codelists/

55. Head A. CPRD_multimorbidity_codelists [Internet]. 2022 [cited 2023 May 9];Available from: https://github.com/annalhead/CPRD_multimorbidity_codelists

56. Kuan V, Denaxas S, Gonzalez-Izquierdo A, Direk K, Bhatti O, Husain S, et al. A chronological map of 308 physical and mental health conditions from 4 million individuals in the English National Health Service. Lancet Digit. Health 2019;1:e63–77.

A) Appendix A: Disease module details

The following subsections present the assumptions, data sources, structural diagram, and validation plots for each condition.

Incidence, prevalence, case-fatality and mortality trends are modelled using GAMLSS models fitted to the CPRD data by sex, age group, deprivation decile, and year.

Disease dependencies are where the incidence of the condition of interest is associated with the prevalence of other condition(s). These were included based on the strength of correlations and known plausible directional associations and modelled as relative risks by sex, age group, and deprivation decile, using logistic regression.

For some conditions, calibration factors were applied based on visual inspection of the simulated data against the observed data. Two types of calibration factors were used: intercept and trend. The intercept calibration factor is multiplied by the simulated incidence to shift it up (calibration factor > 1) or down (calibration factor < 1). The trend calibration factor is applied as an exponential based on the number of years since the start of the simulation. For all case fatality rate plots, the model output is post-calibrated to the ONS mortality; therefore, it is expected to be higher than the CPRD observed mortality.

Model structure

Figure A-1 is a 'birds-eye' diagrammatic presentation of all the relationships modelled between risk factors and conditions and between pairs of conditions, as described in the above sections. As this is difficult to read and interpret, the structural diagram for the relationships affecting each modelled condition is presented alongside the individual condition summaries in the following sub-sections.

In all the diagrams, conditions are in blue and modelled exposures are in red. Modelled associations between risk factors and conditions are shown by the red arrows; modelled associations between prevalent condition x and incident condition y are shown by the blue arrows.

In the disease-specific sub-sections, the incident condition of interest is in black text in the centre, and all direct relationships modelled between exposures and other conditions are shown by the arrows pointing towards the middle.

Figure A-1 Relationships modelled between exposures and conditions.

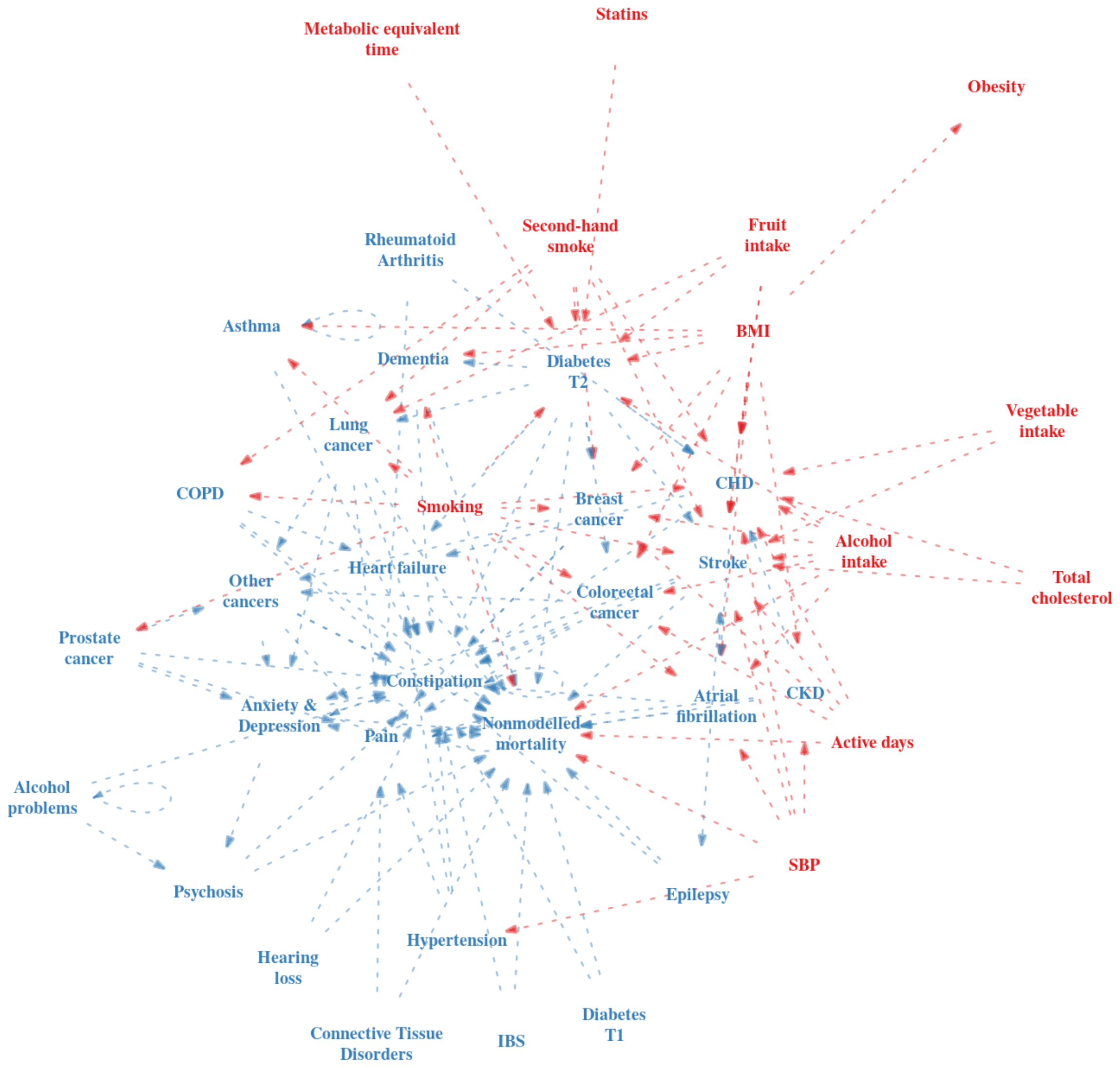

Coronary Heart Disease (CHD)

Table A-1 Modelling assumptions for incident CHD

**Component**	**Assumptions / Details**
**Disease type**	Chronic – no recovery
**Risk factor associations**	Smoking, environmental tobacco smoke, systolic blood pressure, total cholesterol, body mass index, physical activity, alcohol intake, fruit and vegetable intake
**Disease dependencies**	Chronic kidney disease, diabetes mellitus type 2, rheumatoid arthritis
**Calibration factor**	Intercept: 0.97

Figure A-2 - Causal structure of risk factor associations and disease dependencies for incident CHD

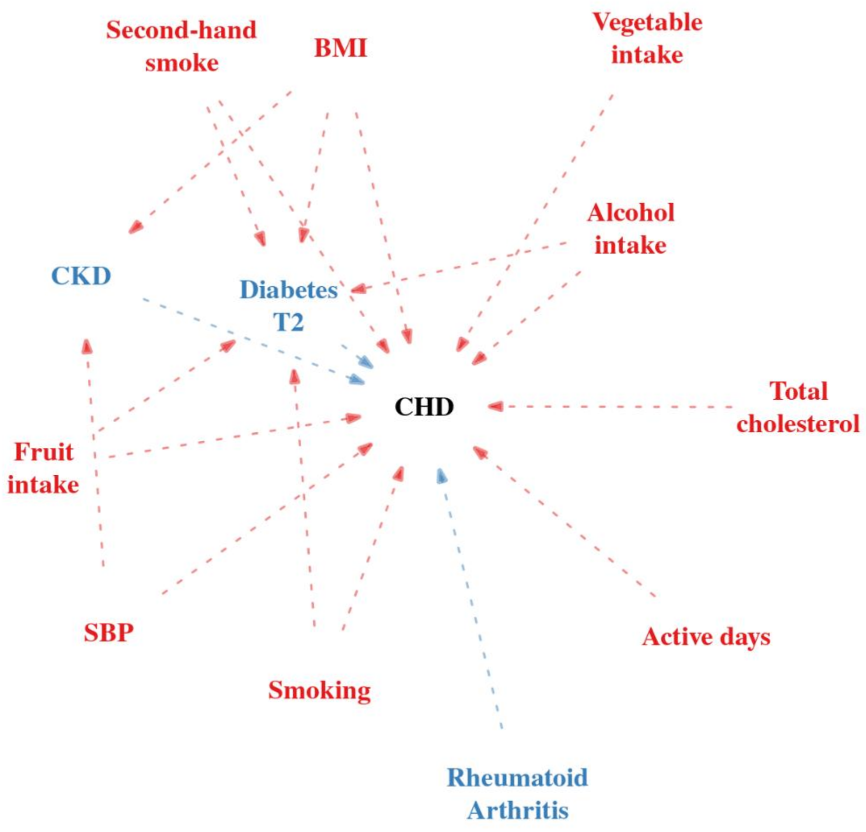

Table A-2 - Data sources for causal associations between risk factors and CHD incidence

Parameter	Details	Comments	Source
Relative risk for active smoking	GBD meta-analysis	RR from the GBD 2019 study	Global Burden of Disease Study 2019 (GBD 2019) Data Resources | GHDx [Internet]. [cited 2022 Mar 16];Available from: http://ghdx.healthdata.org/record/ihme-data/gbd-2019-relative-risks
Relative risk for ex-smoking	GBD meta-analysis	RR from the GBD 2019 study	Global Burden of Disease Study 2019 (GBD 2019) Data Resources | GHDx [Internet]. [cited 2022 Mar 16];Available from: http://ghdx.healthdata.org/record/ihme-data/gbd-2019-relative-risks
Relative risk for environmental tobacco smoking	Meta-analysis of 10 cohort and case-control studies	Adjusted for important CHD risk factors. The effect was applied to never regularly smokers.	He J, Vupputuri S, Allen K, Prerost MR, Hughes J, Whelton PK. Passive Smoking and the Risk of Coronary Heart Disease — A Meta-Analysis of Epidemiologic Studies. N Engl J Med 1999;340:920–6. (Table 3. Adjusted RR)

Parameter	Details	Comments	Source
Relative risk for systolic blood pressure	Meta-analysis of individual data from 61 prospective studies	Stratified by age and sex. Adjusted for regression dilution and total blood cholesterol and, where available, lipid fractions (HDL and non-HDL cholesterol), diabetes, weight, alcohol consumption, and smoking at baseline.	Age-specific relevance of usual blood pressure to vascular mortality: a meta-analysis of individual data for one million adults in 61 prospective studies. The Lancet 2002;360:1903–13. (Figures 3 and 5)
Relative risk for total cholesterol	Meta-analysis of individual data from 61 prospective studies	Stratified by age and sex. Adjusted for regression dilution and age, sex, study, systolic blood pressure and smoking.	Prospective Studies Collaboration. Blood cholesterol and vascular mortality by age, sex, and blood pressure: a meta-analysis of individual data from 61 prospective studies with 55 000 vascular deaths. The Lancet 2007;370:1829–39. (Web-table 6 fully adjusted and Figure 3)
Relative risk for body mass index	Meta-analysis of 58 prospective studies	Stratified by age. Adjusted for age, sex, smoking status, systolic blood pressure, history of diabetes, and total and HDL cholesterol.	The Emerging Risk Factors Collaboration. Separate and combined associations of body-mass index and abdominal adiposity with cardiovascular disease: collaborative analysis of 58 prospective studies. The Lancet 2011;377:1085–95. (Table 1 and Figure 2)

Parameter	Details	Comments	Source
Relative risk for diabetes mellitus type 2	Meta-analysis of 102 prospective studies	Stratified by age. Adjusted for age, smoking status, body-mass index, and systolic blood pressure.	The Emerging Risk Factors Collaboration. Diabetes mellitus, fasting blood glucose concentration, and risk of vascular disease: a collaborative meta-analysis of 102 prospective studies. The Lancet 2010;375:2215–22. (Figure 2)
Relative risk for physical activity	Meta-analysis of 18 cohort studies for CHD and 8 cohort studies for ischaemic stroke	Stratified by age and sex. Adjusted for measurement error, age, sex, smoking, blood pressure and cholesterol.	Bull FC, Armstrong TP, Dixon T, Ham S, Neiman A, Pratt M. Comparative quantification of health risks. Chapter 10: physical inactivity. Geneva: World Health Organisation; 2004. (Tables 10.19 and 10.20)
Relative risk for fruit and vegetable consumption	Meta-analysis of 9 cohort studies	RR per portion of F&V. Multiply-adjusted.	Dauchet L, Amouyel P, Hercberg S, Dallongeville J. Fruit and Vegetable Consumption and Risk of Coronary Heart Disease: A Meta-Analysis of Cohort Studies. J Nutr 2006;136:2588–93.
Relative risk for alcohol intake	GBD meta-analysis	RR from the GBD 2019 study	Global Burden of Disease Study 2019 (GBD 2019) Data Resources | GHDx [Internet]. [cited 2022 Mar 16];Available from: http://ghdx.healthdata.org/record/ihme-data/gbd-2019-relative-risks

Parameter	Details	Comments	Source
Exposure levels below which no excess risk for cardiometabolic disease is assumed for SBP, BMI, and total cholesterol	Pooling of 123 cohorts	Data on 1.4 million individuals and 52,000 CVD events. We assumed the levels are similar for other diseases beyond cardiometabolic.	Singh GM, Danaei G, Farzadfar F, Stevens GA, Woodward M, Wormser D, et al. The age-specific quantitative effects of metabolic risk factors on cardiovascular diseases and diabetes: a pooled analysis. PLOS ONE 2013;8:e65174.

Validation plots for CHD incidence, case fatality, and prevalence

Figure A-3 - Validation plot for modelled CHD incidence (age-sex-dimd standardised)

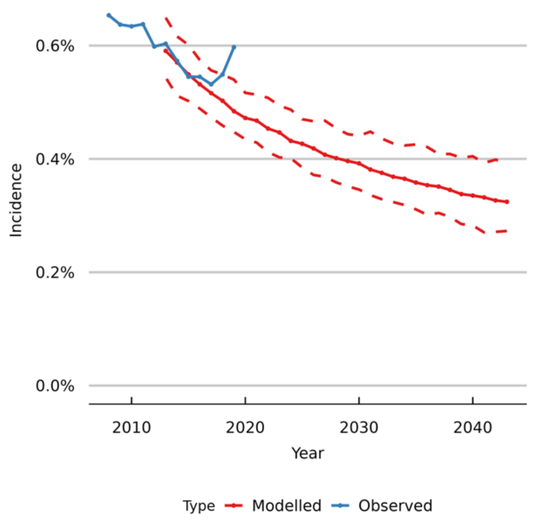

Figure A-4 - Validation plot for modelled CHD case fatality (age-sex-dimd standardised)

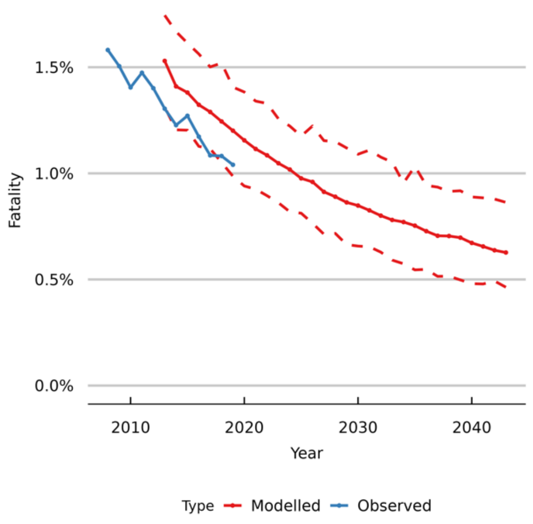

Figure A-5 - Validation plot for modelled CHD prevalence (age-sex-dimd standardised)

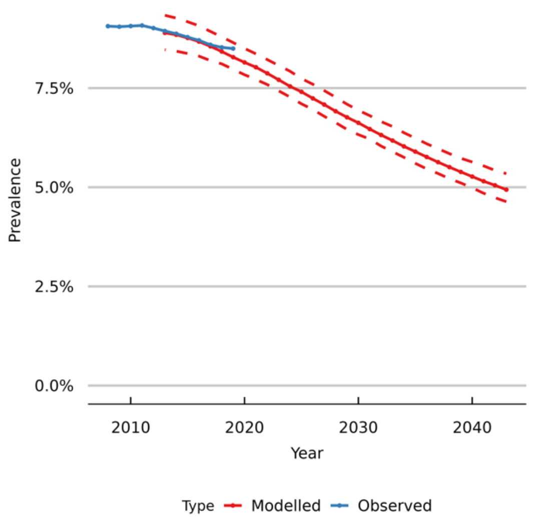

Stroke (including transient ischaemic attack (TIA))

Table A-3 - Modelling assumptions for incident stroke

Component	Assumptions / Details
**Disease type**	Chronic – no recovery
**Risk factor associations**	Smoking, environmental tobacco smoke, systolic blood pressure, total cholesterol, body mass index, physical activity, alcohol intake, fruit and vegetable intake
**Disease dependencies**	Atrial fibrillation, diabetes mellitus type 2
**Calibration factor**	Intercept: 0.99<br>Trend: 1.005

Figure A-6 - Causal structure of risk factor associations and disease dependencies for incident stroke

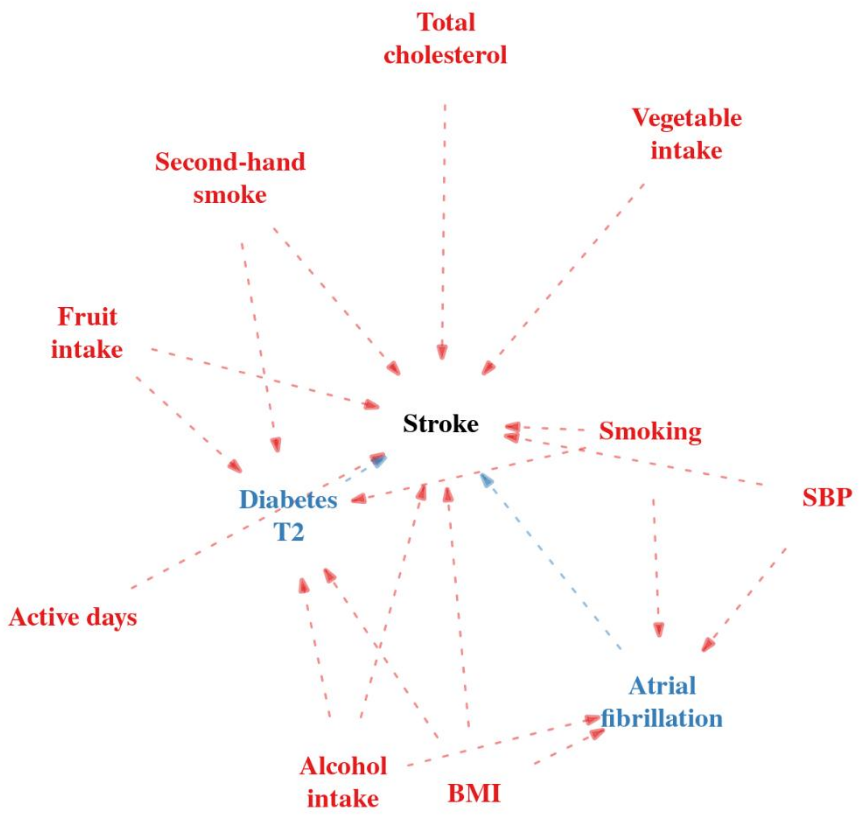

Table A-4 - Data sources for causal associations between risk factors and stroke incidence

Parameter	Details	Comments	Source
Relative risk for active smoking	GBD meta-analysis	RR from the GBD 2019 study	Global Burden of Disease Study 2019 (GBD 2019) Data Resources | GHDx [Internet]. [cited 2022 Mar 16];Available from: http://ghdx.healthdata.org/record/ihme-data/gbd-2019-relative-risks
Relative risk for ex-smoking	GBD meta-analysis	RR from the GBD 2019 study	Global Burden of Disease Study 2019 (GBD 2019) Data Resources | GHDx [Internet]. [cited 2022 Mar 16];Available from: http://ghdx.healthdata.org/record/ihme-data/gbd-2019-relative-risks
Relative risk for environmental tobacco smoking	Meta-analysis of 20 prospective, case-control and cross-sectional studies	13 studies adjusted for important CHD risk factors. The overall effect from all 20 studies was used. The effect was applied to never regularly smokers.	Oono IP, Mackay DF, Pell JP. Meta-analysis of the association between secondhand smoke exposure and stroke. J Public Health 2011;33:496–502. (Figure 1)

Relative risk for systolic blood pressure	Meta-analysis of individual data from 61 prospective studies	Stratified by age and sex. Adjusted for regression dilution and total blood cholesterol and, where available, lipid fractions (HDL and non-HDL cholesterol), diabetes, weight, alcohol consumption, and smoking at baseline.	Age-specific relevance of usual blood pressure to vascular mortality: a meta-analysis of individual data for one million adults in 61 prospective studies. The Lancet 2002;360:1903–13. (Figures 3 and 5)
Relative risk for total cholesterol	Meta-analysis of individual data from 61 prospective studies	Stratified by age and sex. Adjusted for regression dilution and age, sex, study, systolic blood pressure and smoking.	Prospective Studies Collaboration. Blood cholesterol and vascular mortality by age, sex, and blood pressure: a meta-analysis of individual data from 61 prospective studies with 55 000 vascular deaths. The Lancet 2007;370:1829–39. (Web-table 6 fully adjusted and Figure 3)
Relative risk for body mass index	Meta-analysis of 58 prospective studies	Stratified by age. Adjusted for age, sex, smoking status, systolic blood pressure, history of diabetes, and total and HDL cholesterol.	The Emerging Risk Factors Collaboration. Separate and combined associations of body-mass index and abdominal adiposity with cardiovascular disease: collaborative analysis of 58 prospective studies. The Lancet 2011;377:1085–95. (Table 1 and Figure 2)
Relative risk for diabetes mellitus type 2	Meta-analysis of 102 prospective studies	Stratified by age. Adjusted for age, smoking status, body-mass index, and systolic blood pressure.	The Emerging Risk Factors Collaboration. Diabetes mellitus, fasting blood glucose concentration, and risk of vascular disease: a collaborative meta-analysis of 102 prospective studies. The Lancet 2010;375:2215–22. (Figure 2)

Relative risk for physical activity	Meta-analysis of 18 cohort studies for CHD and 8 cohort studies for ischaemic stroke	Stratified by age and sex. Adjusted for measurement error, age, sex, smoking, blood pressure and cholesterol.	Bull FC, Armstrong TP, Dixon T, Ham S, Neiman A, Pratt M. Comparative quantification of health risks. Chapter 10: physical inactivity. Geneva: World Health Organisation; 2004. (Tables 10.19 and 10.20)
Relative risk for fruit and vegetable consumption	Meta-analysis of 7 cohort studies	RR per portion of F&V. Multiply-adjusted.	Dauchet L, Amouyel P, Dallongeville J. Fruit and vegetable consumption and risk of stroke A meta-analysis of cohort studies. Neurology 2005;65:1193–7.
Relative risk for alcohol intake	GBD meta-analysis	RR from the GBD 2019 study	Global Burden of Disease Study 2019 (GBD 2019) Data Resources | GHDx [Internet]. [cited 2022 Mar 16];Available from: http://ghdx.healthdata.org/record/ihme-data/gbd-2019-relative-risks
Relative risk for atrial fibrillation	Cohort study	Ages < 65 are an extrapolation	Yuan Z, Bowlin S, Einstadter D, Cebul RD, Conners AR Jr, Rimm AA. Atrial fibrillation as a risk factor for stroke: a retrospective cohort study of hospitalized Medicare beneficiaries. Am J Public Health 1998 Mar;88(3):395-400

Validation plots for stroke incidence, case fatality, and prevalence

Figure A-7 - Validation plot for modelled stroke incidence (age-sex-dimd standardised)

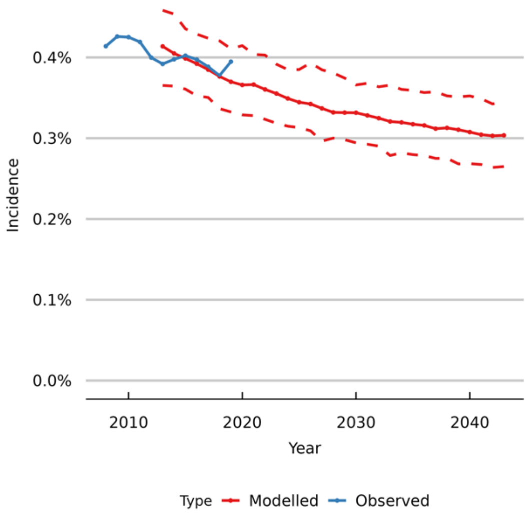

Figure A-8 - Validation plot for modelled stroke case fatality (age-sex-dimd standardised)

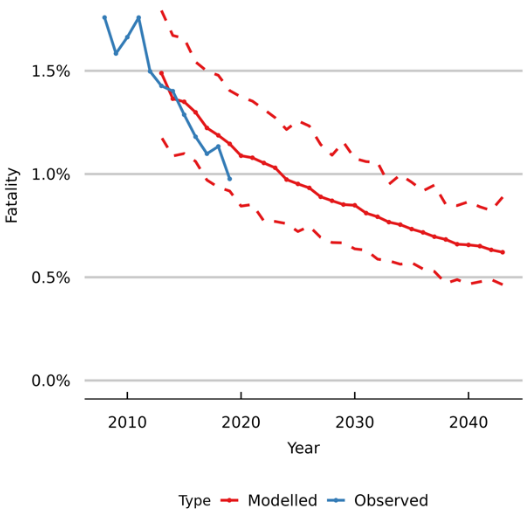

Figure A-9 - Validation plot for modelled stroke prevalence (age-sex-dimd standardised)

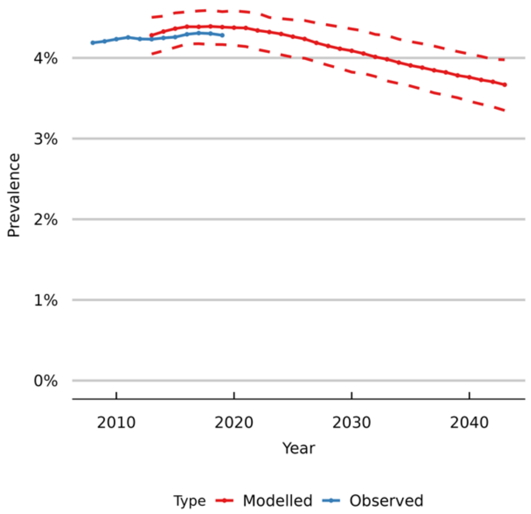

Breast cancer

Table A-5 - Modelling assumptions for incident breast cancer

Component	Assumptions / Details
**Disease type**	Recovery after 10 years; no recurrence; women only
**Risk factor associations**	Smoking, environmental tobacco smoke, body mass index, physical activity, alcohol intake
**Disease dependencies**	Diabetes mellitus type 2
**Calibration factor**	None

Figure A-10 - Causal structure of risk factor associations and disease dependencies for incident breast cancer

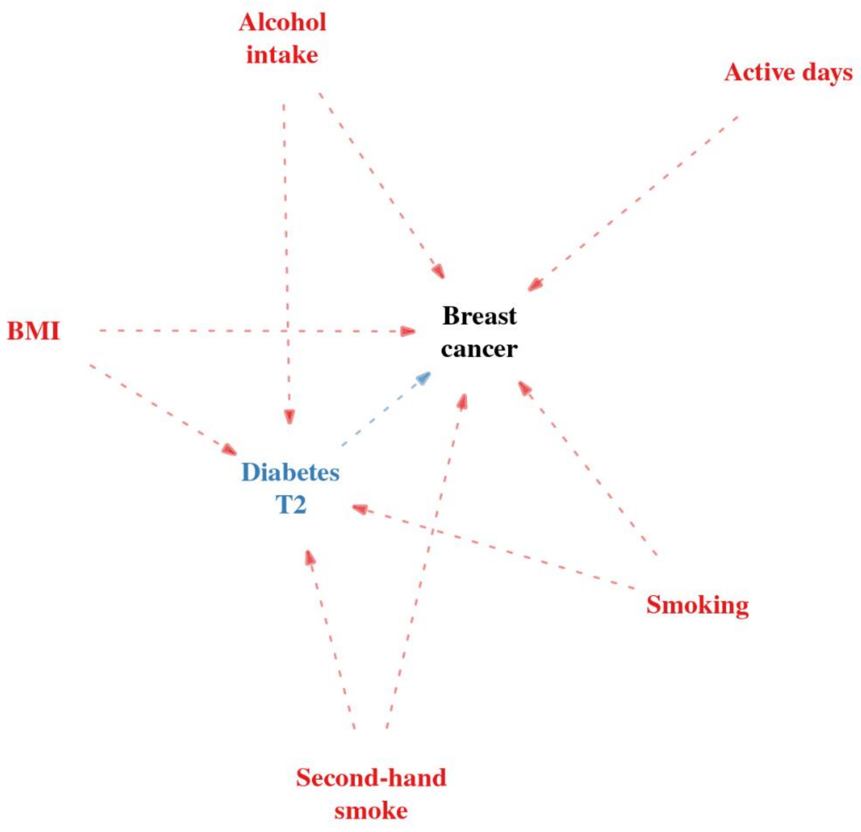

Table A-6 - Data sources for causal associations between risk factors and breast cancer incidence

Parameter	Details	Comments	Source
Relative risk for active smoking	Random effect meta-analysis of 27 prospective and 44 retrospective studies	The results were stable across different subgroup analyses, notably pre/post-menopause, alcohol consumption adjustments, including/excluding passive smokers from the referent group.	Macacu A, Autier P, Boniol M, Boyle P. Active and passive smoking and risk of breast cancer: a meta-analysis. Breast Cancer Res Treat 2015;154:213–24. (Table 1)
Relative risk for ex-smoking	GBD meta-analysis	RR from the GBD 2019 study	Global Burden of Disease Study 2019 (GBD 2019) Data Resources | GHDx [Internet]. [cited 2022 Mar 16];Available from: http://ghdx.healthdata.org/record/ihme-data/gbd-2019-relative-risks
Relative risk for environmental tobacco smoking	GBD meta-analysis	RR from the GBD 2019 study	Global Burden of Disease Study 2019 (GBD 2019) Data Resources | GHDx [Internet]. [cited 2022 Mar 16];Available from: http://ghdx.healthdata.org/record/ihme-data/gbd-2019-relative-risks

Relative risk for body mass index	GBD meta-analysis	RR from the GBD 2019 study	Global Burden of Disease Study 2019 (GBD 2019) Data Resources | GHDx [Internet]. [cited 2022 Mar 16];Available from: http://ghdx.healthdata.org/record/ihme-data/gbd-2019-relative-risks
Relative risk for diabetes mellitus type 2	GBD meta-analysis	RR from the GBD 2019 study	Global Burden of Disease Study 2019 (GBD 2019) Data Resources | GHDx [Internet]. [cited 2022 Mar 16];Available from: http://ghdx.healthdata.org/record/ihme-data/gbd-2019-relative-risks
Relative risk for physical activity	Meta-analysis		Bull FC, Armstrong TP, Dixon T, Ham S, Neiman A, Pratt M. Comparative quantification of health risks. Chapter 10: physical inactivity. Geneva: World Health Organisation; 2004. (Tables 10.19 and 10.20)
Relative risk for alcohol intake	GBD meta-analysis	RR from the GBD 2019 study	Global Burden of Disease Study 2019 (GBD 2019) Data Resources | GHDx [Internet]. [cited 2022 Mar 16];Available from: http://ghdx.healthdata.org/record/ihme-data/gbd-2019-relative-risks

Validation plots for breast cancer incidence, case fatality, and prevalence

Figure A-11 - Validation plot for modelled breast cancer incidence (age-sex-dimd standardised)

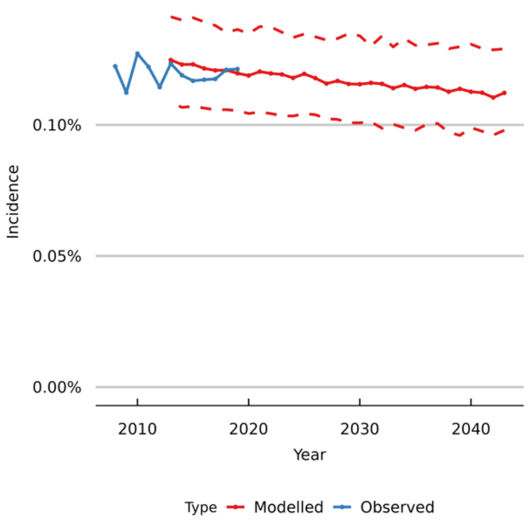

Figure A-12 - Validation plot for modelled breast cancer case fatality (age-sex-dimd standardised)

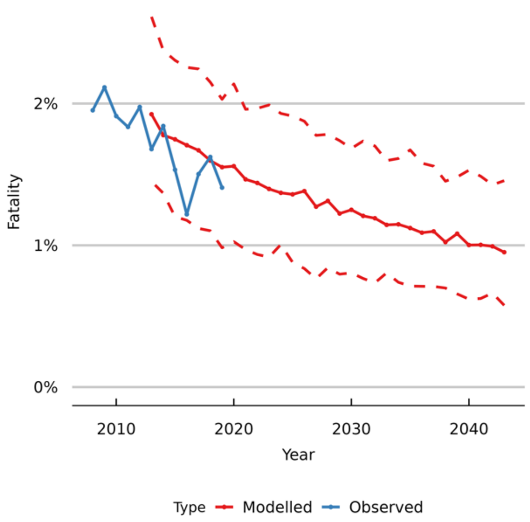

Figure A-13 - Validation plot for modelled breast cancer prevalence (age-sex-dimd standardised)

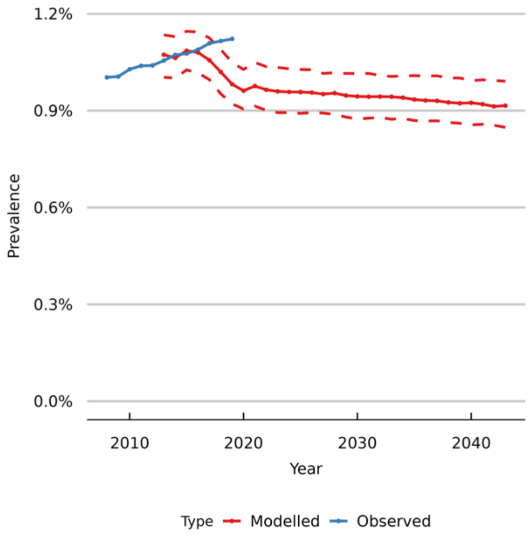

Colorectal cancer

Table A-7 - Modelling assumptions for incident colorectal cancer

Component	Assumptions / Details
**Disease type**	Recovery after 10 years; no recurrence
**Risk factor associations**	Smoking, body mass index, physical activity, alcohol intake
**Disease dependencies**	Diabetes mellitus type 2
**Calibration factor**	None

Figure A-14 - Causal structure of risk factor associations and disease dependencies for incident colorectal cancer

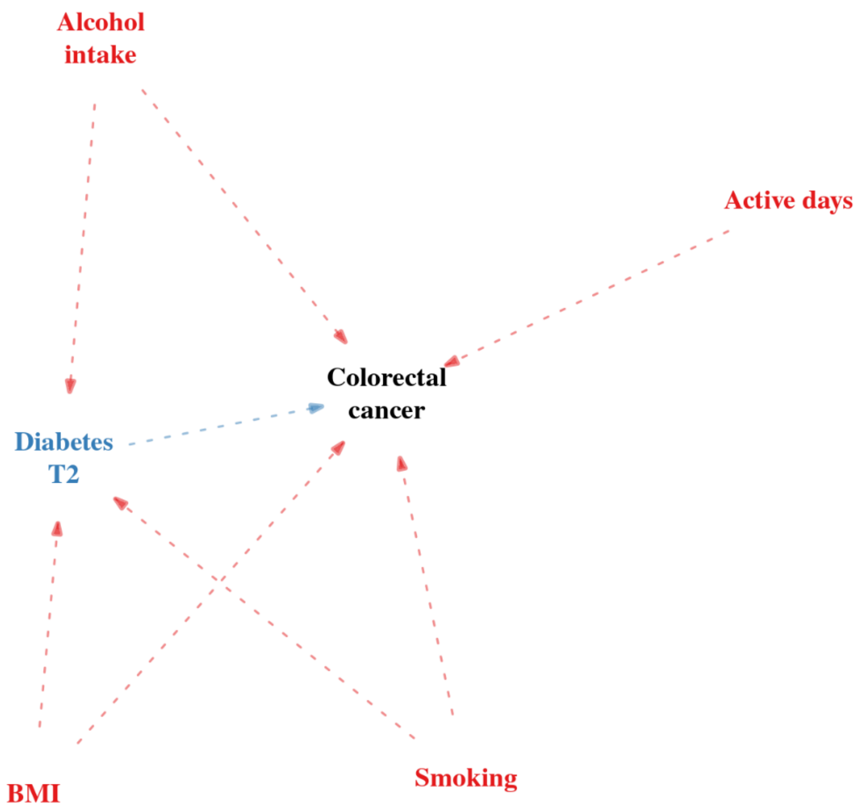

Table A-8 - Data sources for causal associations between risk factors and colorectal cancer incidence

Parameter	Details	Comments	Source
Relative risk for pack years	GBD meta-analysis	RR from the GBD 2019 study	Global Burden of Disease Study 2019 (GBD 2019) Data Resources | GHDx [Internet]. [cited 2022 Mar 16];Available from: http://ghdx.healthdata.org/record/ihme-data/gbd-2019-relative-risks
Relative risk for ex-smoking	GBD meta-analysis	RR from the GBD 2019 study	Global Burden of Disease Study 2019 (GBD 2019) Data Resources | GHDx [Internet]. [cited 2022 Mar 16];Available from: http://ghdx.healthdata.org/record/ihme-data/gbd-2019-relative-risks
Relative risk for body mass index	GBD meta-analysis	RR from the GBD 2019 study	Global Burden of Disease Study 2019 (GBD 2019) Data Resources | GHDx [Internet]. [cited 2022 Mar 16];Available from: http://ghdx.healthdata.org/record/ihme-data/gbd-2019-relative-risks
Relative risk for diabetes mellitus type 2	GBD meta-analysis	RR from the GBD 2019 study	Global Burden of Disease Study 2019 (GBD 2019) Data Resources | GHDx [Internet]. [cited 2022 Mar 16];Available from: http://ghdx.healthdata.org/record/ihme-data/gbd-2019-relative-risks
Relative risk for physical activity	GBD meta-analysis	RR from the GBD 2019 study	Global Burden of Disease Study 2019 (GBD 2019) Data Resources | GHDx [Internet]. [cited 2022 Mar 16];Available from: http://ghdx.healthdata.org/record/ihme-data/gbd-2019-relative-risks
Relative risk for alcohol intake	GBD meta-analysis	RR from the GBD 2019 study	Global Burden of Disease Study 2019 (GBD 2019) Data Resources | GHDx [Internet]. [cited 2022 Mar 16];Available from: http://ghdx.healthdata.org/record/ihme-data/gbd-2019-relative-risks

Validation plots for colorectal cancer incidence, case fatality, and prevalence

Figure A-15 - Validation plot for modelled colorectal cancer incidence (age-sex-dimd standardised)

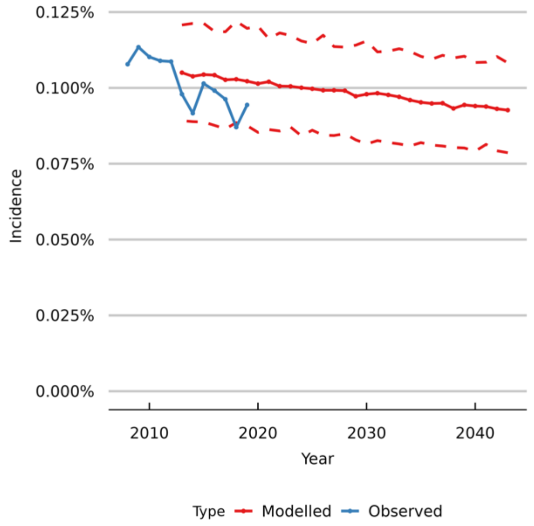

Figure A-16 - Validation plot for modelled colorectal cancer case fatality (age-sex-dimd standardised)

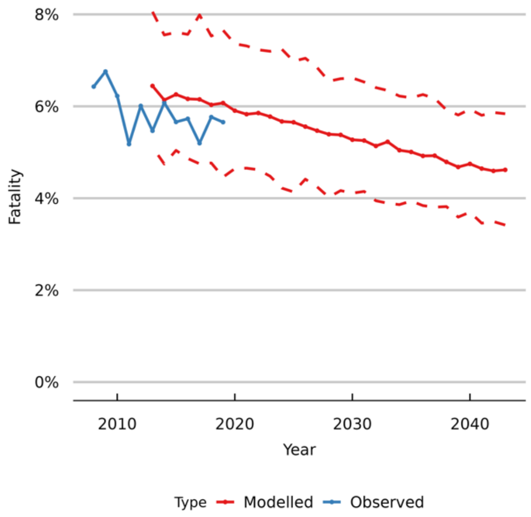

Figure A-17 - Validation plot for modelled colorectal cancer prevalence (age-sex-dimd standardised)

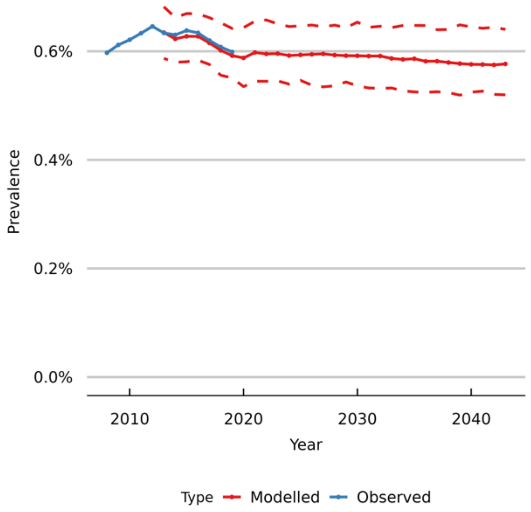

Lung cancer

Table A-9 - Modelling assumptions for incident lung cancer

Component	Assumptions / Details
**Disease type**	Recovery after 10 years; no recurrence
**Risk factor associations**	Smoking, environmental tobacco smoke, fruit intake
**Disease dependencies**	Diabetes mellitus type 2
**Calibration factor**	Intercept: 1.005<br><br>Trend:1.1

Figure A-18 - Causal structure of risk factor associations and disease dependencies for incident lung cancer

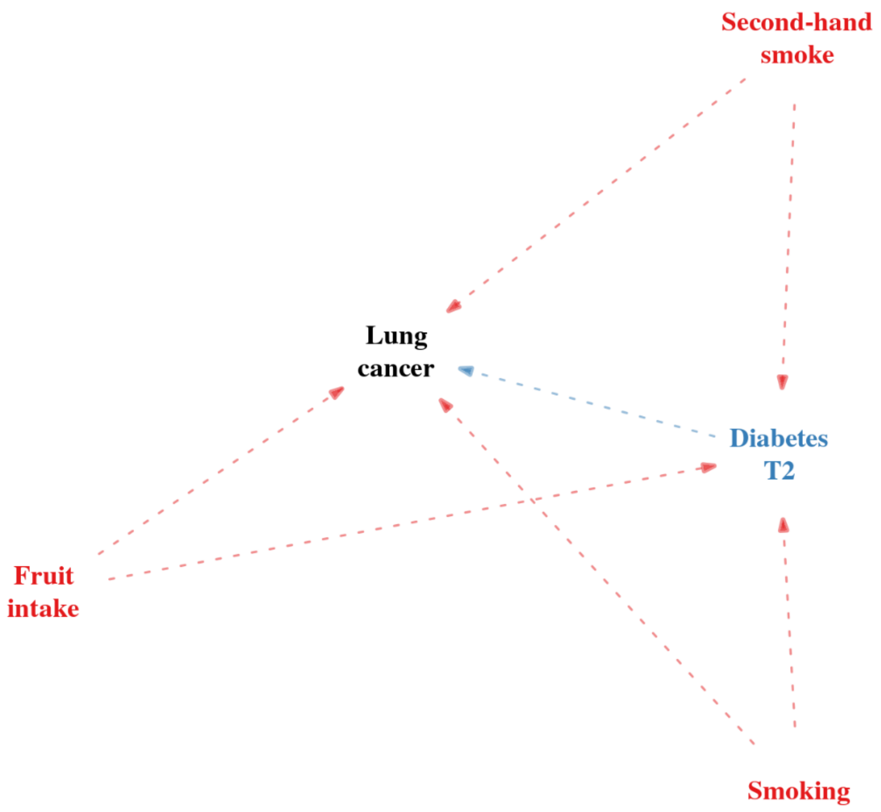

Table A-10 - Data sources for causal associations between risk factors and lung cancer incidence

Parameter	Details	Comments	Source
Relative risk for pack years	RCT of 208,371 individuals	We used the PLCO2014 model	Tammemägi MC, Church TR, Hocking WG, Silvestri GA, Kvale PA, Riley TL, et al. Evaluation of the lung cancer risks at which to screen ever- and never-smokers: screening rules applied to the PLCO and NLST cohorts. PLoS Med 2014;11:e1001764. (Table S1)
Relative risk for ex-smoking	GBD meta-analysis	RR from the GBD 2019 study	Global Burden of Disease Study 2019 (GBD 2019) Data Resources | GHDx [Internet]. [cited 2022 Mar 16];Available from: http://ghdx.healthdata.org/record/ihme-data/gbd-2019-relative-risks
Relative risk for environmental tobacco smoking	Meta-analysis of 18 case-control studies	The effect was applied to never regularly smokers.	Kim CH, Lee Y-CA, Hung RJ, McNallan SR, Cote ML, Lim W-Y, et al. Exposure to secondhand tobacco smoke and lung cancer by histological type: A pooled analysis of the International Lung Cancer Consortium (ILCCO). Int. J. Cancer 2014;135:1918–30.

Relative risk for fruit consumption	Dose response meta-analysis	The effect was like that estimated by Wang Y, et al. Fruit and vegetable consumption and risk of lung cancer: A dose-response meta-analysis of prospective cohort studies. Lung Cancer 2015;88:124-30.	Vieira AR, Abar L, Vingeliene S, Chan DSM, Aune D, Navarro-Rosenblatt D, et al. Fruits, vegetables and lung cancer risk: a systematic review and meta-analysis. Ann Oncol 2016;27:81–96
Relative risk for diabetes mellitus type 2	GBD meta-analysis	RR from the GBD 2019 study	Global Burden of Disease Study 2019 (GBD 2019) Data Resources | GHDx [Internet]. [cited 2022 Mar 16];Available from: http://ghdx.healthdata.org/record/ihme-data/gbd-2019-relative-risks

Validation plots for lung cancer incidence, case fatality, and prevalence

Figure A-19 - Validation plot for modelled lung cancer incidence (age-sex-dimd standardised)

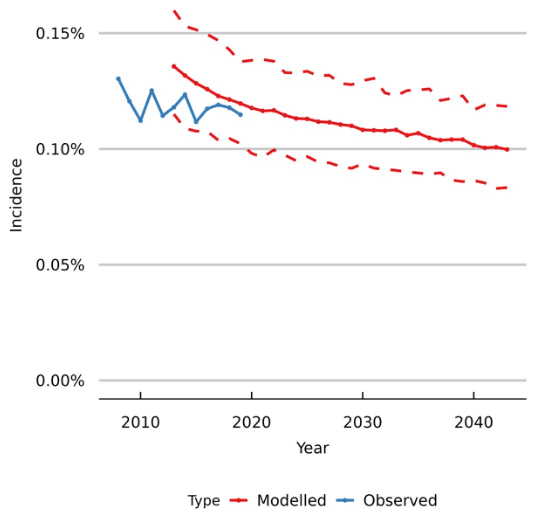

Figure A-20 - Validation plot for modelled lung cancer case fatality (age-sex-dimd standardised)

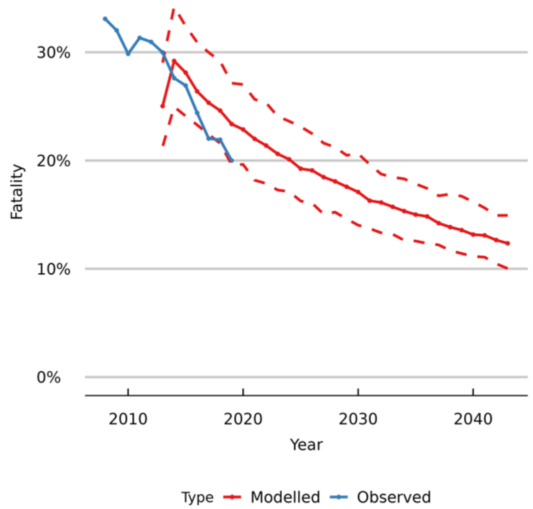

Figure A-21 - Validation plot for modelled lung cancer prevalence (age-sex-dimd standardised)

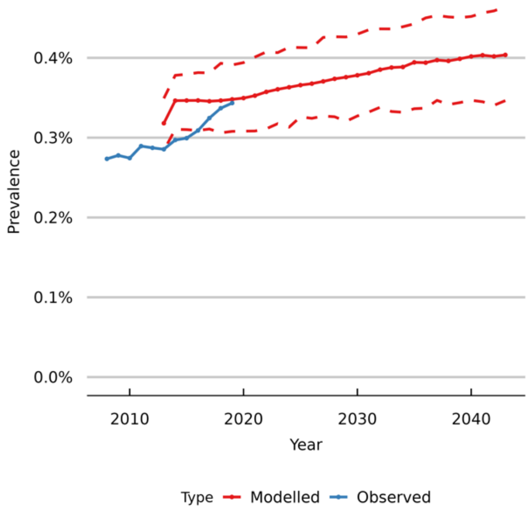

Prostate cancer

Table A-11 - Modelling assumptions for incident prostate cancer

Component	Assumptions / Details
**Disease type**	Recovery after 10 years; no recurrence; men only
**Risk factor associations**	Smoking
**Disease dependencies**	None
**Calibration factor**	Trend: 1.002

Figure A-22 - Causal structure of risk factor associations and disease dependencies for incident prostate cancer

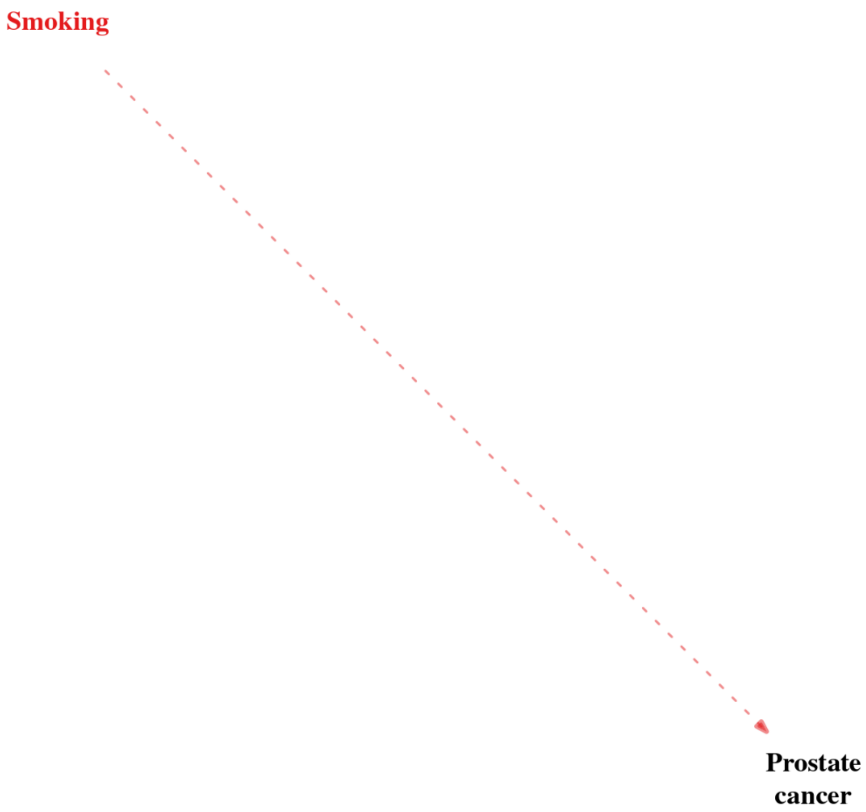

Table A-12 - Data sources for causal associations between risk factors and prostate cancer incidence

Parameter	Details	Comments	Source
Relative risk for active smoking	GBD meta-analysis	RR from the GBD 2019 study	Global Burden of Disease Study 2019 (GBD 2019) Data Resources | GHDx [Internet]. [cited 2022 Mar 16];Available from: http://ghdx.healthdata.org/record/ihme-data/gbd-2019-relative-risks
Relative risk for ex-smoking	GBD meta-analysis	RR from the GBD 2019 study	Global Burden of Disease Study 2019 (GBD 2019) Data Resources | GHDx [Internet]. [cited 2022 Mar 16];Available from: http://ghdx.healthdata.org/record/ihme-data/gbd-2019-relative-risks

Validation plots for prostate cancer incidence, case fatality, and prevalence

Figure A-23 - Validation plot for modelled prostate cancer incidence (age-sex-dimd standardised)

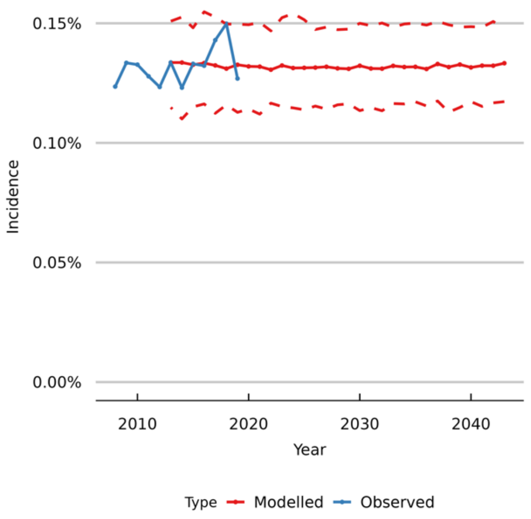

Figure A-24 - Validation plot for modelled prostate cancer case fatality (age-sex-dimd standardised)

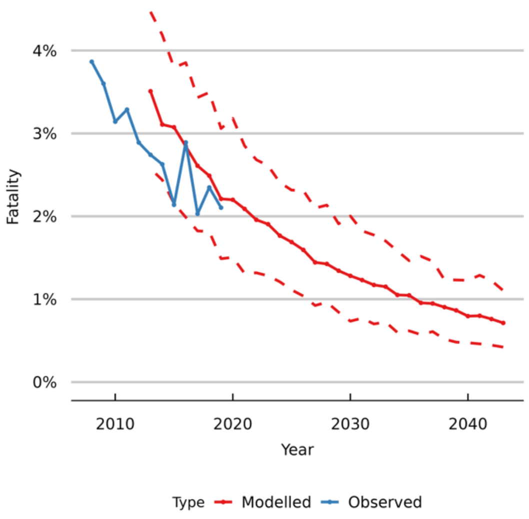

Figure A-25 - Validation plot for modelled prostate cancer prevalence (age-sex-dimd standardised)

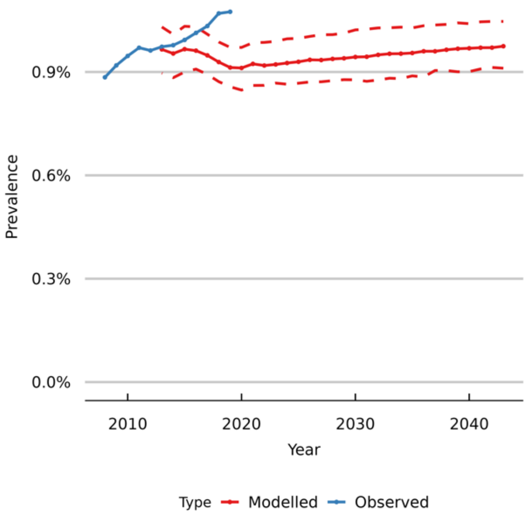

Other cancers

Table A-13 - Modelling assumptions for incident other cancers

Component	Assumptions / Details
**Disease type**	Recovery after 10 years; no recurrence; all primary malignancies aside from breast, colorectal, lung and prostate
**Risk factor associations**	None
**Disease dependencies**	Breast cancer, colorectal cancer, lung cancer, prostate cancer
**Calibration factor**	Intercept: 0.98<br>Trend: 0.999

Figure A-26 - Causal structure of risk factor associations and disease dependencies for incident other cancer

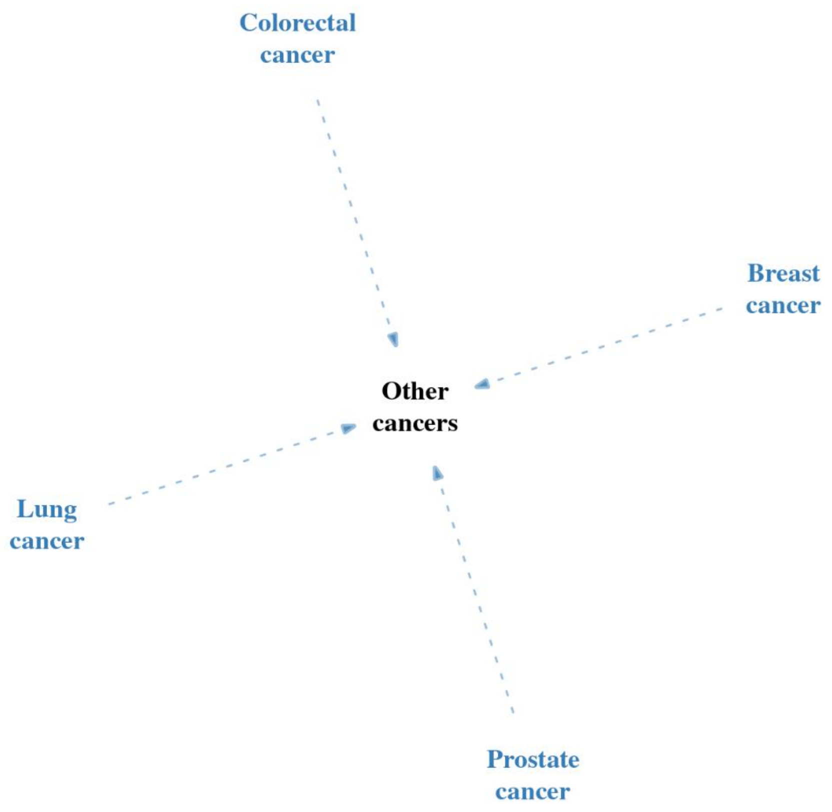

Validation plots for other cancer incidence, case fatality, and prevalence

Figure A-27 - Validation plot for modelled other cancer incidence (age-sex-dimd standardised)

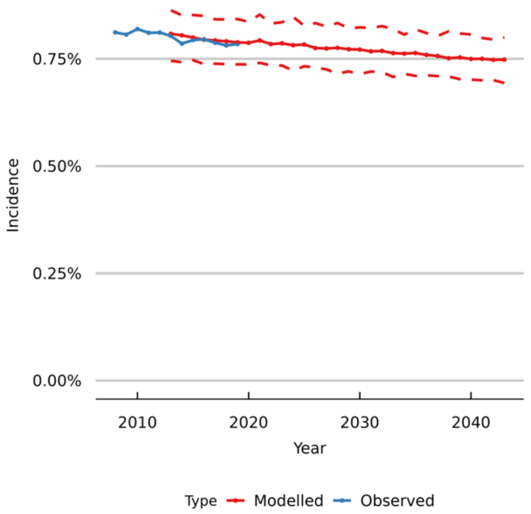

Figure A-28 - Validation plot for modelled other cancer case fatality (age-sex-dimd standardised)

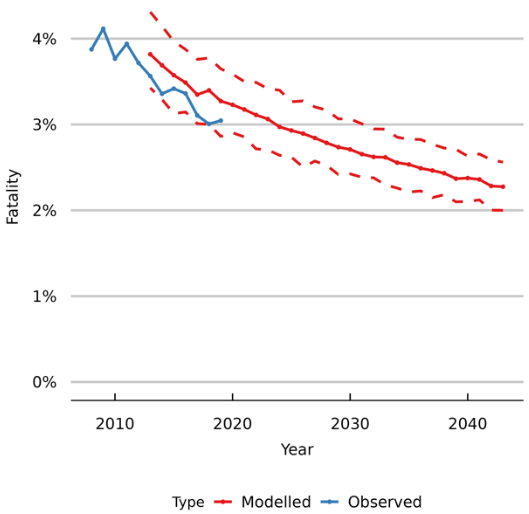

Figure A-29 - Validation plot for modelled other cancer prevalence (age-sex-dimd standardised)

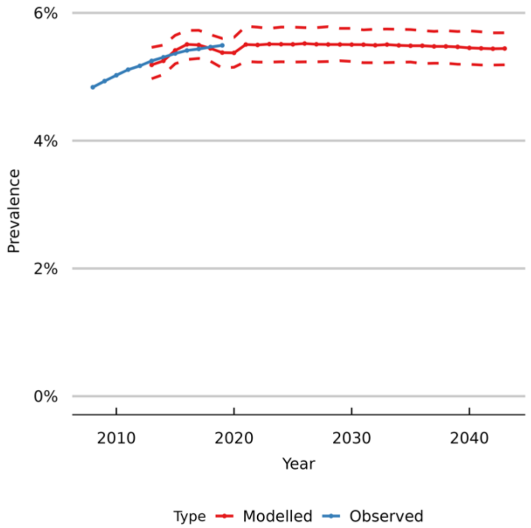

Chronic obstructive pulmonary disease (COPD)

Table A-14 - Modelling assumptions for incident COPD

Component	Assumptions / Details
**Disease type**	Chronic – no recovery
**Risk factor associations**	Smoking, environmental tobacco smoke
**Disease dependencies**	None
**Calibration factor**	None

Figure A-30 - Causal structure of risk factor associations and disease dependencies for incident COPD

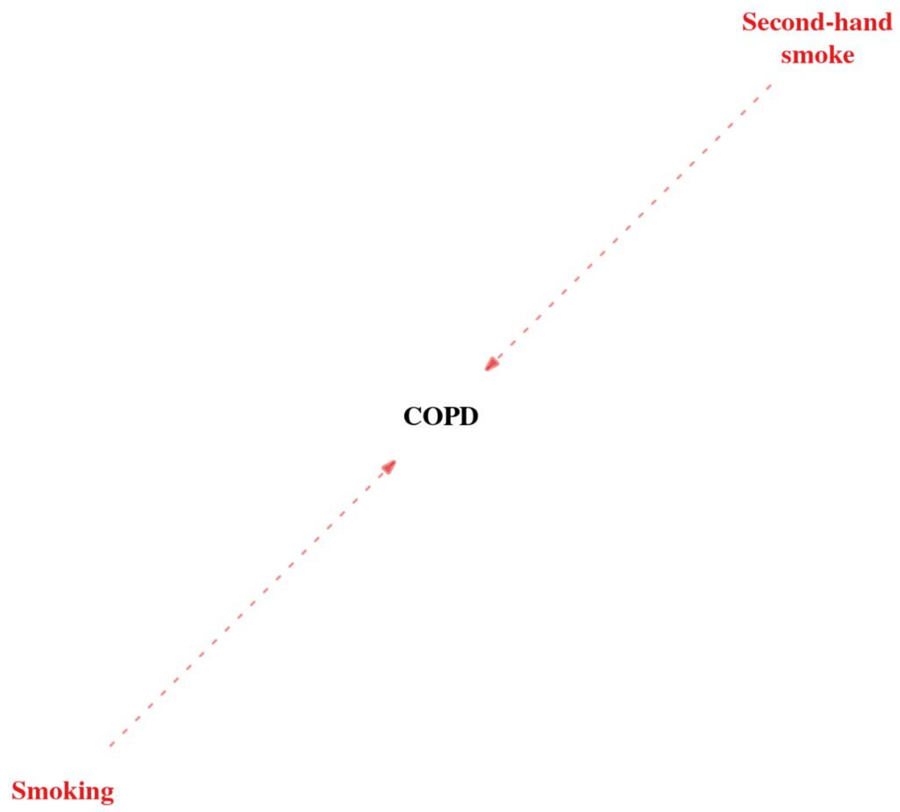

Table A-15 - Data sources for causal associations between risk factors and COPD incidence

Parameter	Details	Comments	Source
Relative risk for pack years	Very detailed random effect meta-analysis	Smoking duration was not significant but intensity and pack years were. We used pack years because they indirectly capture age effect. Most studies for pack years were about incidence rather than mortality. There was no differentiation between current and ex-smokers. This may dilute the effect	Forey BA, Thornton AJ, Lee PN. Systematic review with meta-analysis of the epidemiological evidence relating smoking to COPD, chronic bronchitis and emphysema. BMC Pulmonary Medicine. 2011 Jun 14;11(1):36.
Relative risk for ex-smoking	GBD meta-analysis	RR from the GBD 2019 study	Global Burden of Disease Study 2019 (GBD 2019) Data Resources | GHDx [Internet]. [cited 2022 Mar 16];Available from: http://ghdx.healthdata.org/record/ihme-data/gbd-2019-relative-risks
Relative risk for environmental tobacco smoking	Random effect meta-analysis of 24 studies	The effect was applied to never regularly smokers.	Fischer F, Kraemer A. Meta-analysis of the association between second-hand smoke exposure and ischaemic heart diseases, COPD and stroke. BMC Public Health. 2015 Dec;15(1):1202.

Validation plots for COPD incidence, case fatality, and prevalence

Figure A-31 - Validation plot for modelled COPD incidence (age-sex-dimd standardised)

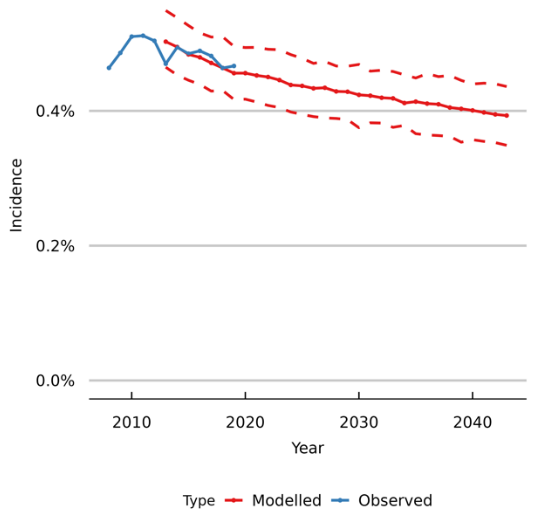

Figure A-32 - Validation plot for modelled COPD case fatality (age-sex-dimd standardised)

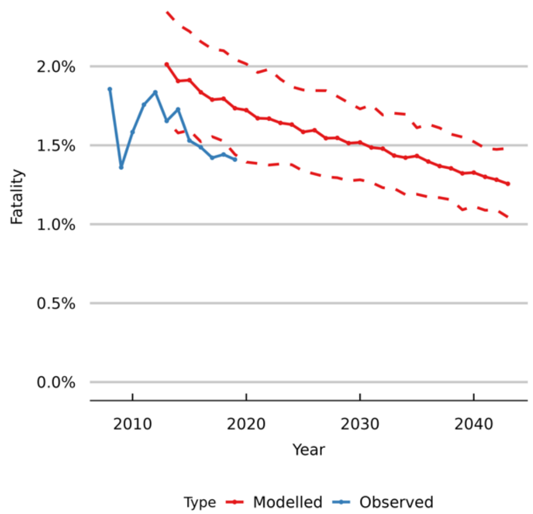

Figure A-33 - Validation plot for modelled COPD prevalence (age-sex-dimd standardised)

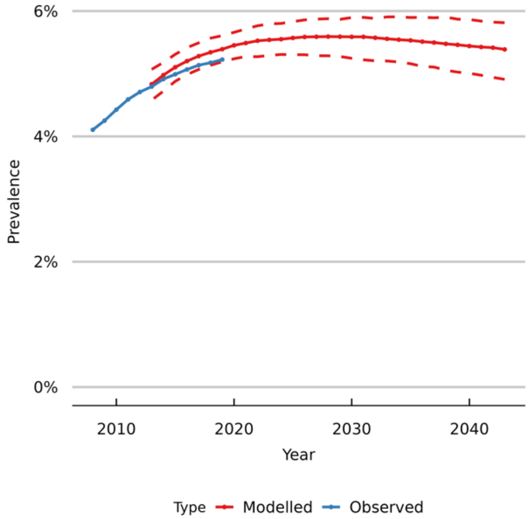

Atrial fibrillation

Table A-16 - Modelling assumptions for incident atrial fibrillation

Component	Assumptions / Details
**Disease type**	Chronic – no recovery
**Risk factor associations**	Smoking, alcohol intake, systolic blood pressure, body mass index
**Disease dependencies**	Coronary heart disease
**Calibration factor**	Trend: 1.002

Figure A-34 - Causal structure of risk factor associations and disease dependencies for incident atrial fibrillation

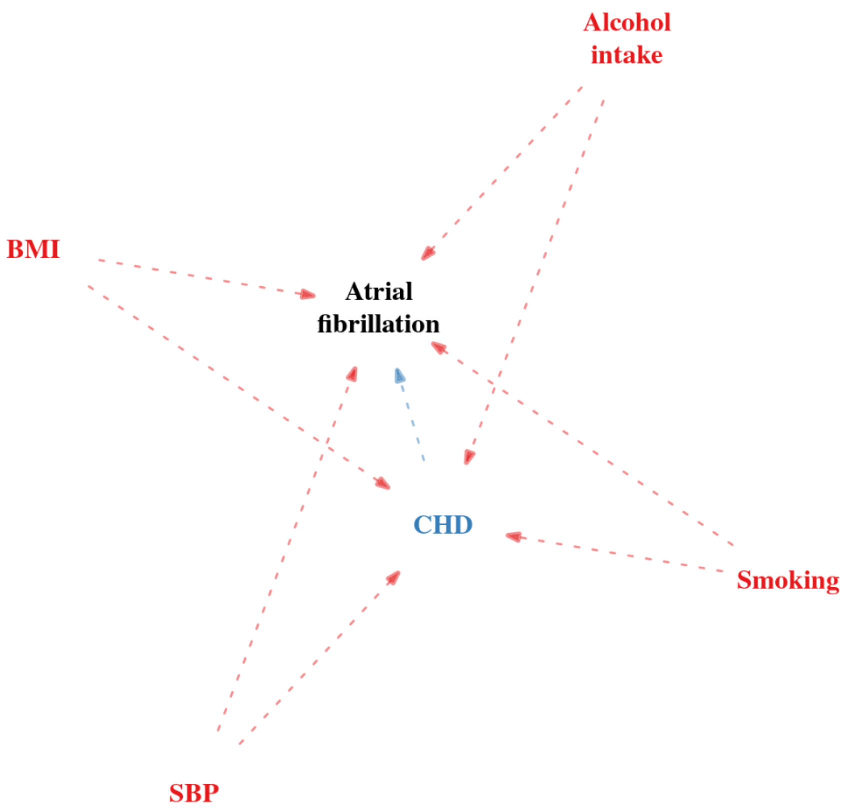

Table A-17 - Data sources for causal associations between risk factors and atrial fibrillation incidence

Parameter	Details	Comments	Source
Relative risk for active smoking	GBD meta-analysis	RR from the GBD 2019 study	Global Burden of Disease Study 2019 (GBD 2019) Data Resources | GHDx [Internet]. [cited 2022 Mar 16];Available from: http://ghdx.healthdata.org/record/ihme-data/gbd-2019-relative-risks
Relative risk for ex-smoking	GBD meta-analysis	RR from the GBD 2019 study	Global Burden of Disease Study 2019 (GBD 2019) Data Resources | GHDx [Internet]. [cited 2022 Mar 16];Available from: http://ghdx.healthdata.org/record/ihme-data/gbd-2019-relative-risks
Relative risk for systolic blood pressure	GBD meta-analysis	RR from the GBD 2019 study	Global Burden of Disease Study 2019 (GBD 2019) Data Resources | GHDx [Internet]. [cited 2022 Mar 16];Available from: http://ghdx.healthdata.org/record/ihme-data/gbd-2019-relative-risks
Relative risk for body mass index	GBD meta-analysis	RR from the GBD 2019 study	Global Burden of Disease Study 2019 (GBD 2019) Data Resources | GHDx [Internet]. [cited 2022 Mar 16];Available from: http://ghdx.healthdata.org/record/ihme-data/gbd-2019-relative-risks
Relative risk for alcohol intake	GBD meta-analysis	RR from the GBD 2019 study	Global Burden of Disease Study 2019 (GBD 2019) Data Resources | GHDx [Internet]. [cited 2022 Mar 16];Available from: http://ghdx.healthdata.org/record/ihme-data/gbd-2019-relative-risks

Validation plots for atrial fibrillation incidence, case fatality, and prevalence

Figure A-35 - Validation plot for modelled atrial fibrillation incidence (age-sex-dimd standardised)

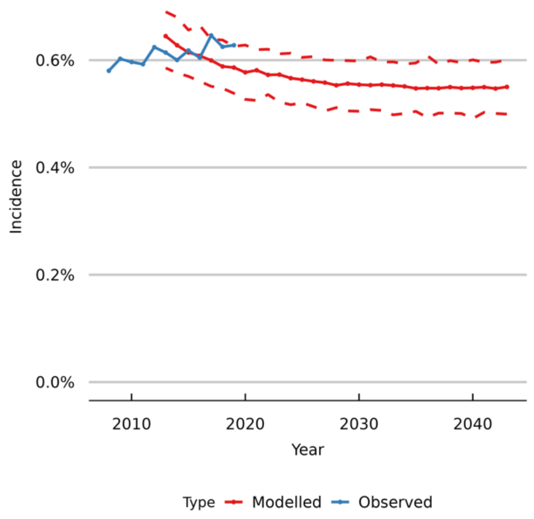

Figure A-36 - Validation plot for modelled atrial fibrillation case fatality (age-sex-dimd standardised)

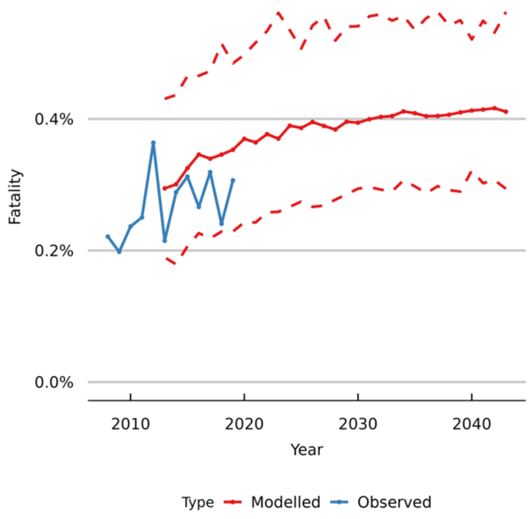

Figure A-37 - Validation plot for modelled atrial fibrillation prevalence (age-sex-dimd standardised)

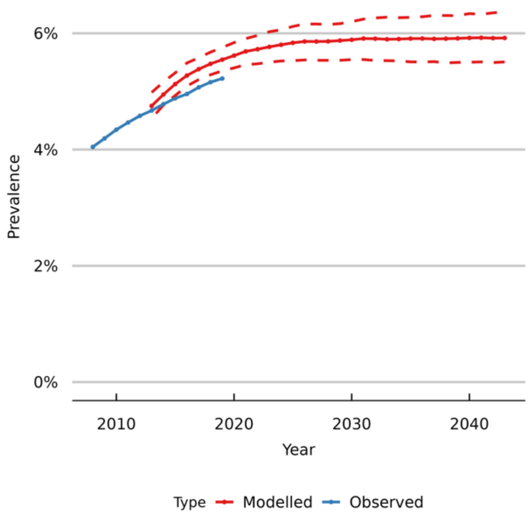

Heart Failure

Table A-18 - Modelling assumptions for incident heart failure

Component	Assumptions / Details
**Disease type**	Chronic – no recovery
**Risk factor associations**	None
**Disease dependencies**	Diabetes mellitus type 2, CHD, COPD, hypertension
**Calibration factor**	Trend: 1.01

Figure A-38 - Causal structure of risk factor associations and disease dependencies for incident heart failure

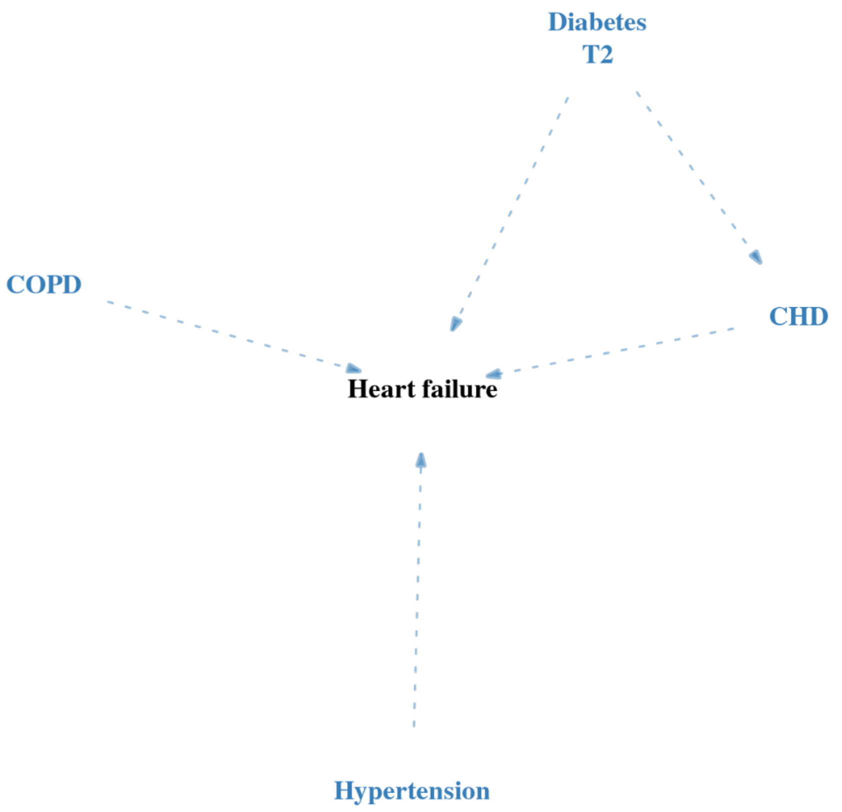

Validation plots for heart failure incidence, case fatality, and prevalence

Figure A-39 - Validation plot for modelled heart failure incidence (age-sex-dimd standardised)

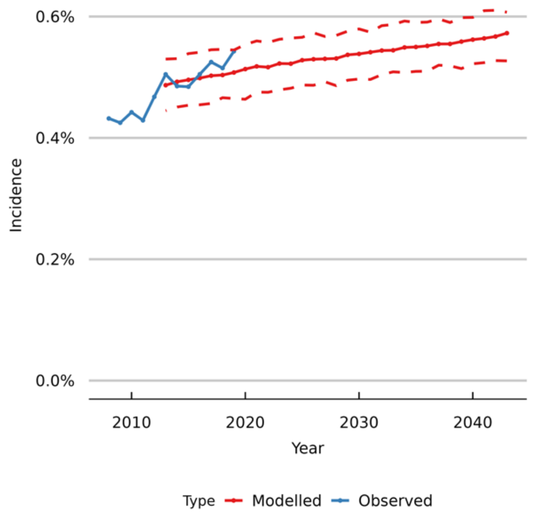

Figure A-40 - Validation plot for modelled heart failure case fatality (age-sex-dimd standardised)

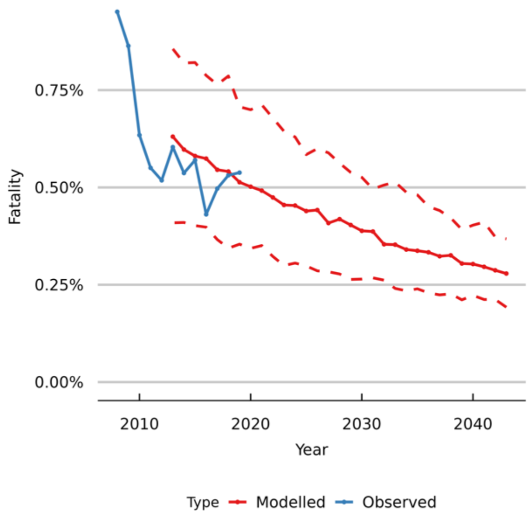

Figure A-41 - Validation plot for modelled heart failure prevalence (age-sex-dimd standardised)

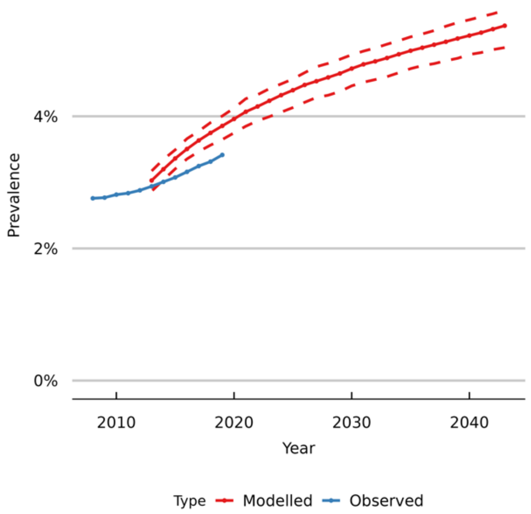

Type 2 diabetes mellitus

Table A-19 - Modelling assumptions for incident type 2 diabetes mellitus

Component	Assumptions / Details
**Disease type**	Chronic – no recovery
**Risk factor associations**	Smoking, environmental tobacco smoke, alcohol intake, body mass index, fruit intake, metabolic equivalent task, statins prescription
**Disease dependencies**	None
**Calibration factor**	Intercept: 1.05

Figure A-42 - Causal structure of risk factor associations and disease dependencies for incident type 2 diabetes mellitus

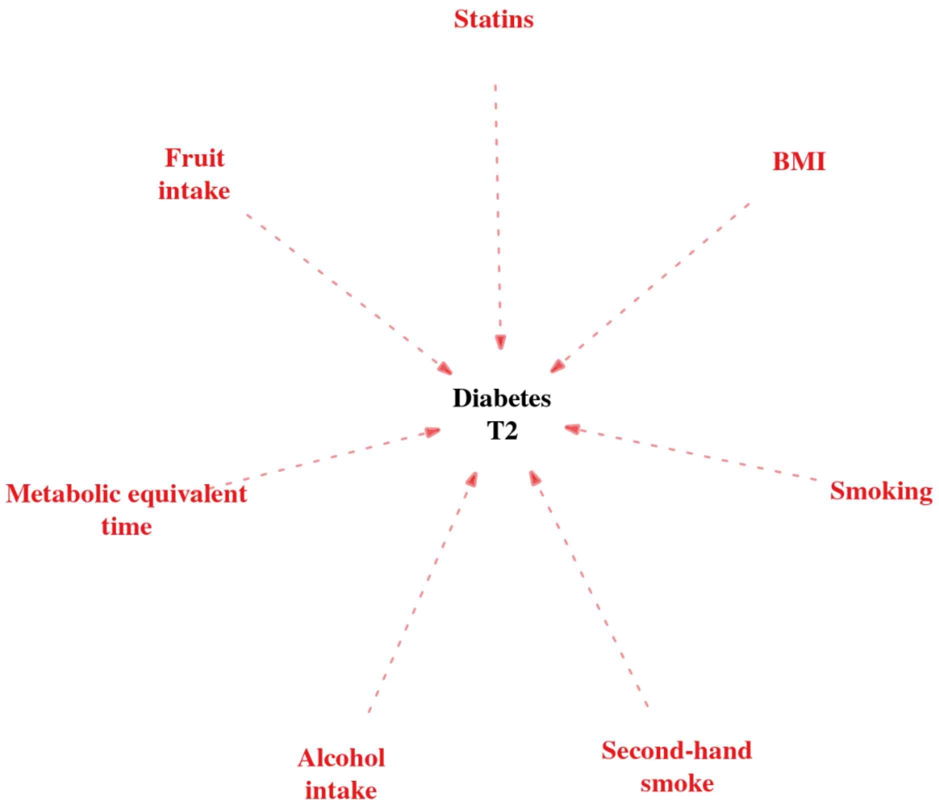

Table A-20 - Data sources for causal associations between risk factors and type 2 diabetes mellitus incidence

Parameter	Details	Comments	Source
Relative risk for active smoking	GBD meta-analysis	RR from the GBD 2019 study	Global Burden of Disease Study 2019 (GBD 2019) Data Resources | GHDx [Internet]. [cited 2022 Mar 16];Available from: http://ghdx.healthdata.org/record/ihme-data/gbd-2019-relative-risks
Relative risk for ex-smoking	GBD meta-analysis	RR from the GBD 2019 study	Global Burden of Disease Study 2019 (GBD 2019) Data Resources | GHDx [Internet]. [cited 2022 Mar 16];Available from: http://ghdx.healthdata.org/record/ihme-data/gbd-2019-relative-risks
Relative risk for environmental tobacco smoking	GBD meta-analysis	RR from the GBD 2019 study	Global Burden of Disease Study 2019 (GBD 2019) Data Resources | GHDx [Internet]. [cited 2022 Mar 16];Available from: http://ghdx.healthdata.org/record/ihme-data/gbd-2019-relative-risks
Relative risk for body mass index	GBD meta-analysis	RR from the GBD 2019 study	Global Burden of Disease Study 2019 (GBD 2019) Data Resources | GHDx [Internet]. [cited 2022 Mar 16];Available from: http://ghdx.healthdata.org/record/ihme-data/gbd-2019-relative-risks
Relative risk for alcohol intake	GBD meta-analysis	RR from the GBD 2019 study	Global Burden of Disease Study 2019 (GBD 2019) Data Resources | GHDx [Internet]. [cited 2022 Mar 16];Available from: http://ghdx.healthdata.org/record/ihme-data/gbd-2019-relative-risks

Relative risk for fruit consumption	GBD meta-analysis	RR from the GBD 2019 study	Global Burden of Disease Study 2019 (GBD 2019) Data Resources | GHDx [Internet]. [cited 2022 Mar 16];Available from: http://ghdx.healthdata.org/record/ihme-data/gbd-2019-relative-risks
Relative risk for Metabolic Equivalent of Task (MET)	GBD meta-analysis	RR from the GBD 2019 study. Metabolic Equivalent of Task (MET) minutes per week	Global Burden of Disease Study 2019 (GBD 2019) Data Resources | GHDx [Internet]. [cited 2022 Mar 16];Available from: http://ghdx.healthdata.org/record/ihme-data/gbd-2019-relative-risks
Relative risk for statins prescription	Random-effect meta-analysis	Assuming RR is approximately equal to Odds Ratio	Westendorp RG, Shepherd J, Davis BR, Pressel SL, Marchioli R, Marfisi RM, et al. Statins and risk of incident diabetes: a collaborative meta-analysis of randomised statin trials. Lancet. 2010 Feb 27;375(9716):735-42

Validation plots for type 2 diabetes mellitus incidence, case fatality, and prevalence

Figure A-43 - Validation plot for modelled type 2 diabetes mellitus incidence (age-sex-dimd standardised)

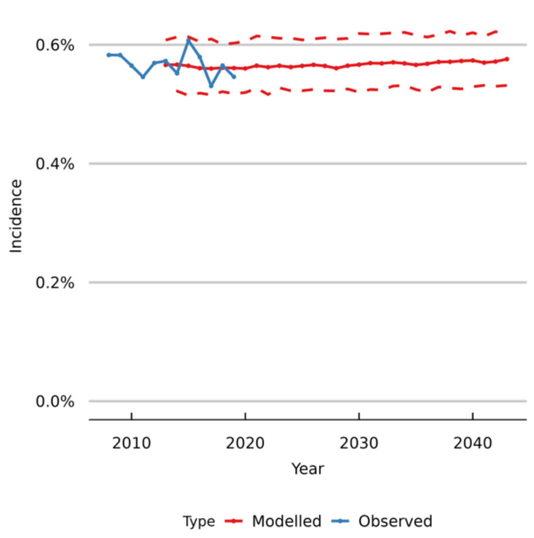

Figure A-44 - Validation plot for modelled type 2 diabetes mellitus case fatality (age-sex-dimd standardised)

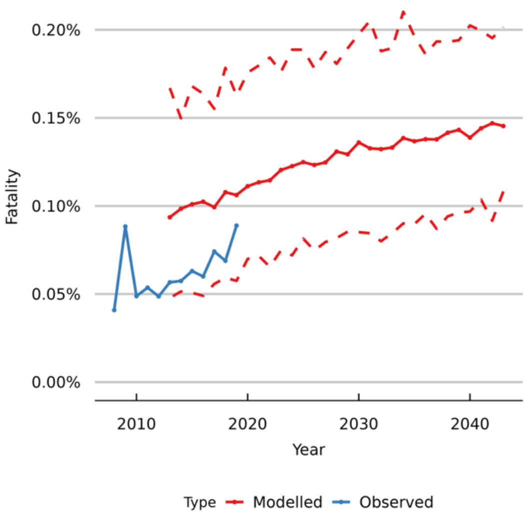

Figure A-45 - Validation plot for modelled type 2 diabetes mellitus prevalence (age-sex-dimd standardised)

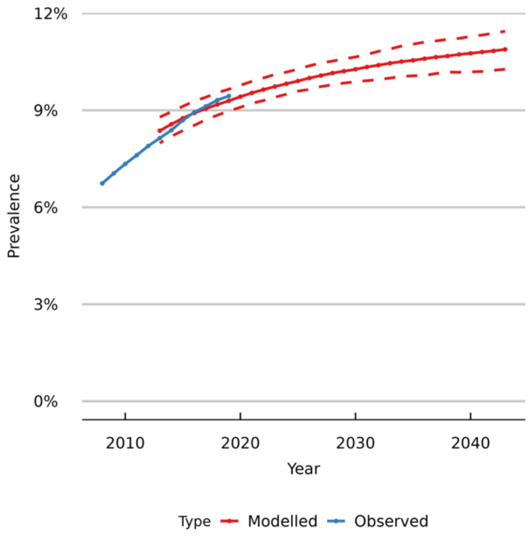

Chronic kidney disease (CKD)

Table A-21 - Modelling assumptions for incident chronic kidney disease

Component	Assumptions / Details
**Disease type**	Chronic – no recovery
**Risk factor associations**	Body mass index, systolic blood pressure
**Disease dependencies**	None
**Calibration factor**	Trend: 1.002

Figure A-46 - Causal structure of risk factor associations and disease dependencies for incident chronic kidney disease

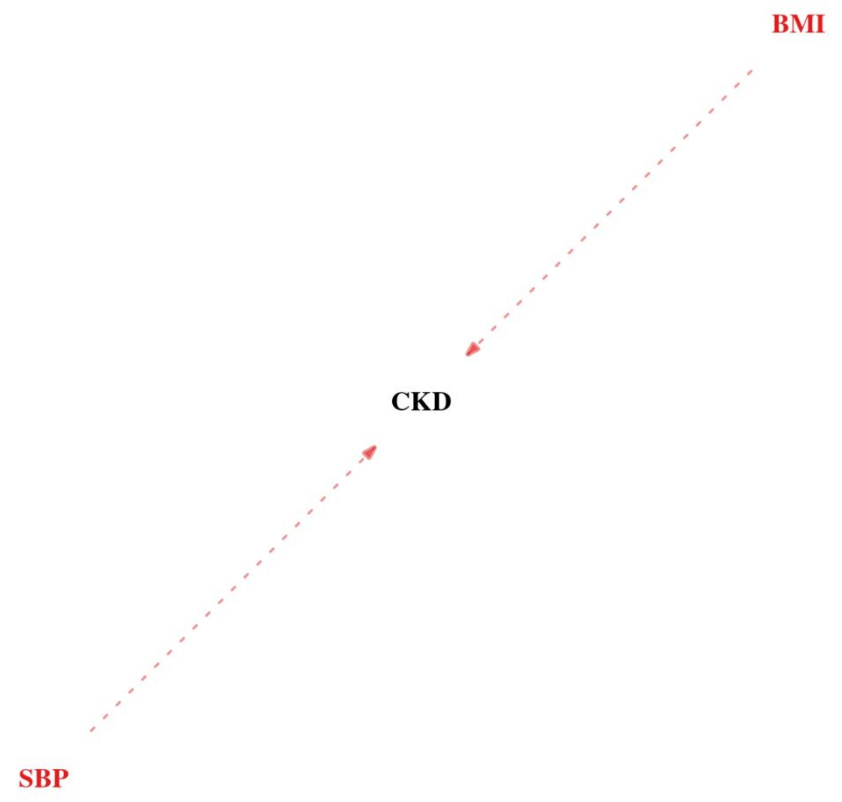

Table A-22 - Data sources for causal associations between risk factors and chronic kidney disease incidence

Parameter	Details	Comments	Source
Relative risk for body mass index	GBD meta-analysis	RR from the GBD 2019 study	Global Burden of Disease Study 2019 (GBD 2019) Data Resources | GHDx [Internet]. [cited 2022 Mar 16];Available from: http://ghdx.healthdata.org/record/ihme-data/gbd-2019-relative-risks
Relative risk for systolic blood pressure	GBD meta-analysis	RR from the GBD 2019 study	Global Burden of Disease Study 2019 (GBD 2019) Data Resources | GHDx [Internet]. [cited 2022 Mar 16];Available from: http://ghdx.healthdata.org/record/ihme-data/gbd-2019-relative-risks

Validation plots for chronic kidney disease incidence and prevalence

Figure A-47 - Validation plot for modelled chronic kidney disease incidence (age-sex-dimd standardised)

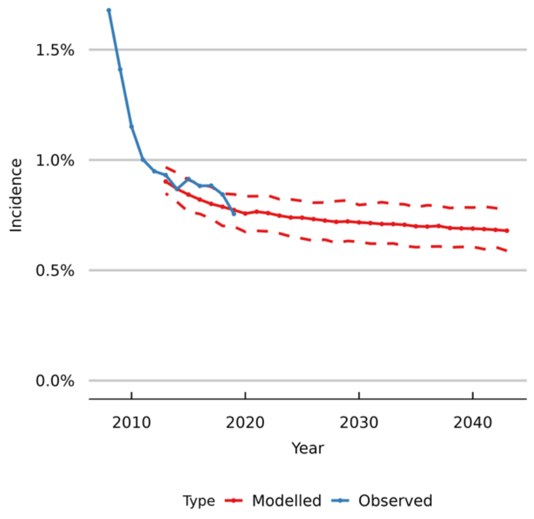

Figure A-48 - Validation plot for modelled chronic kidney disease prevalence (age-sex-dimd standardised)

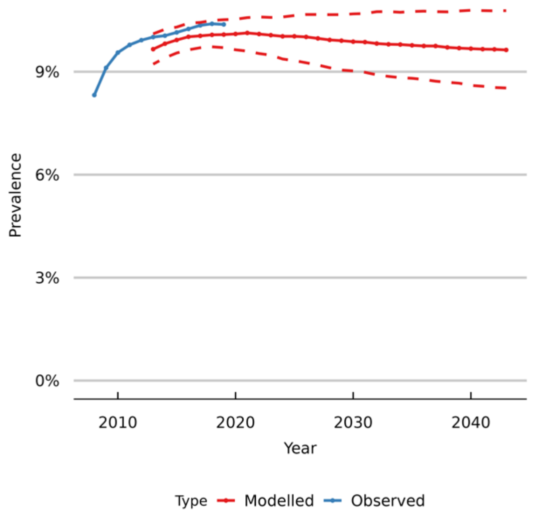

Dementia

Table A-23 - Modelling assumptions for incident dementia

Component	Assumptions / Details
**Disease type**	Chronic – no recovery
**Risk factor associations**	Smoking, body mass index
**Disease dependencies**	Diabetes mellitus type 2
**Calibration factor**	None

Figure A-49 - Causal structure of risk factor associations and disease dependencies for incident dementia

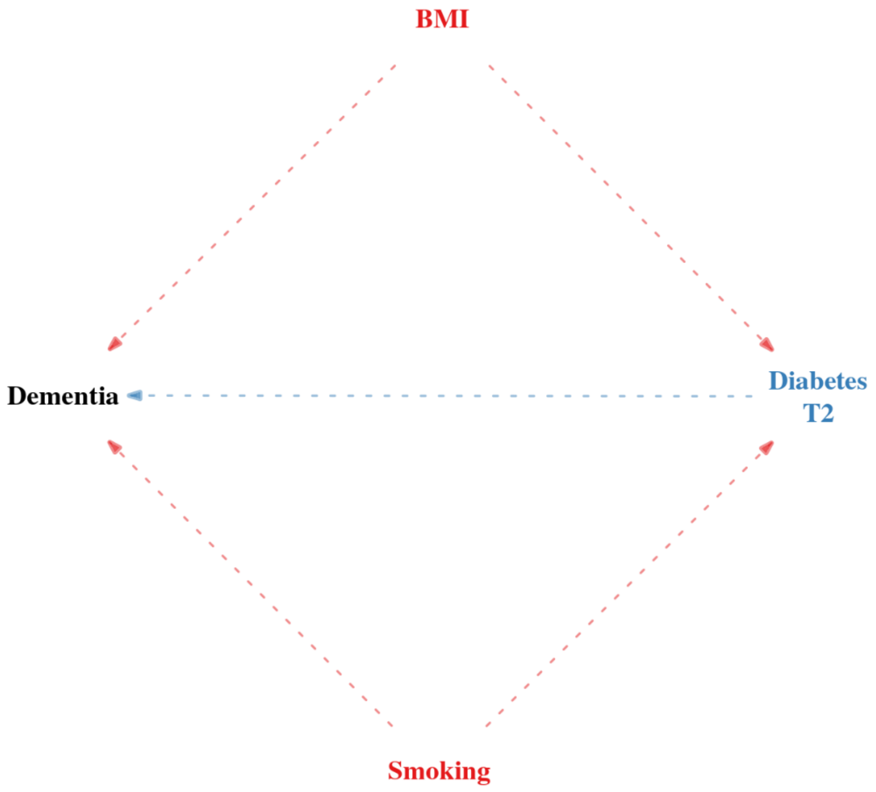

Table A-24 - Data sources for causal associations between risk factors and dementia incidence

Parameter	Details	Comments	Source
Relative risk for active smoking	GBD meta-analysis	RR from the GBD 2019 study	Global Burden of Disease Study 2019 (GBD 2019) Data Resources | GHDx [Internet]. [cited 2022 Mar 16];Available from: http://ghdx.healthdata.org/record/ihme-data/gbd-2019-relative-risks
Relative risk for ex-smoking	GBD meta-analysis	RR from the GBD 2019 study	Global Burden of Disease Study 2019 (GBD 2019) Data Resources | GHDx [Internet]. [cited 2022 Mar 16];Available from: http://ghdx.healthdata.org/record/ihme-data/gbd-2019-relative-risks
Relative risk for body mass index	GBD meta-analysis	RR from the GBD 2019 study	Global Burden of Disease Study 2019 (GBD 2019) Data Resources | GHDx [Internet]. [cited 2022 Mar 16];Available from: http://ghdx.healthdata.org/record/ihme-data/gbd-2019-relative-risks
Relative risk for diabetes mellitus type 2	GBD meta-analysis	RR from the GBD 2019 study	Global Burden of Disease Study 2019 (GBD 2019) Data Resources | GHDx [Internet]. [cited 2022 Mar 16];Available from: http://ghdx.healthdata.org/record/ihme-data/gbd-2019-relative-risks

Validation plots for dementia incidence, case fatality, and prevalence

Figure A-50 - Validation plot for modelled dementia incidence (age-sex-dimd standardised)

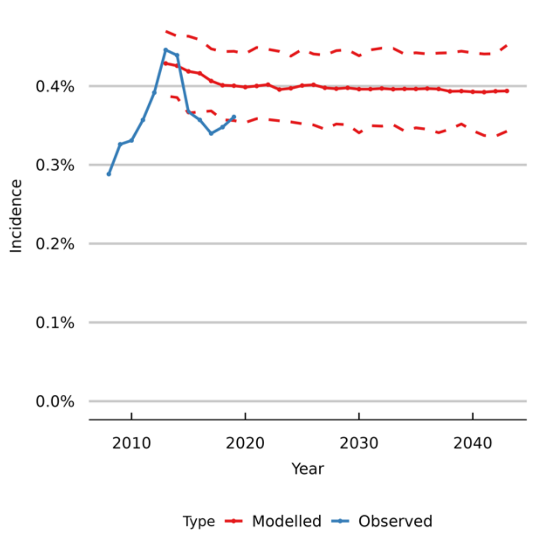

Figure A-51 - Validation plot for modelled dementia case fatality (age-sex-dimd standardised)

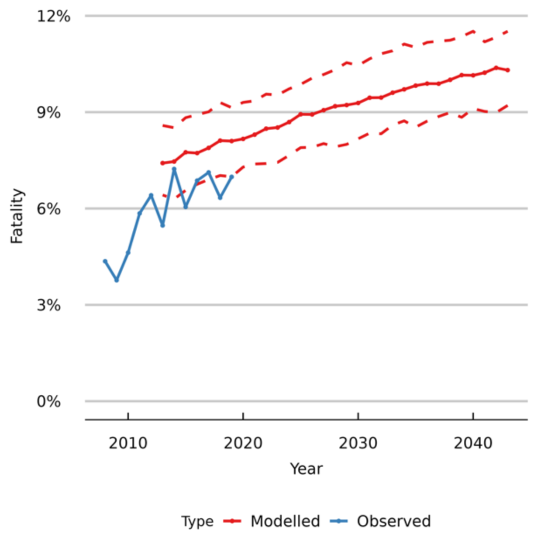

Figure A-52 - Validation plot for modelled dementia prevalence (age-sex-dimd standardised)

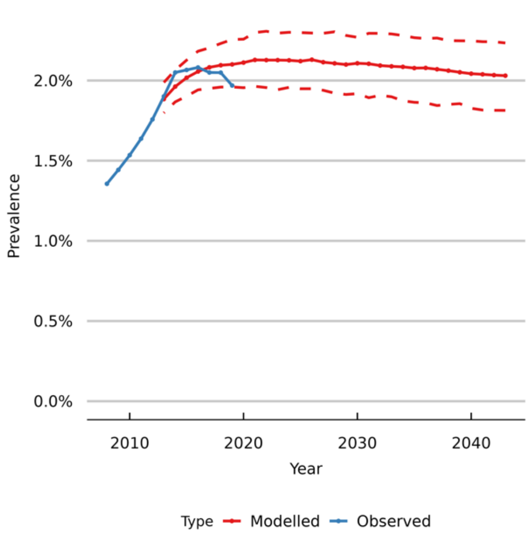

Hypertension

Table A-25 - Modelling assumptions for incident hypertension

Component	Assumptions / Details
**Disease type**	Chronic – no recovery
**Risk factor associations**	Systolic blood pressure
**Disease dependencies**	None
**Calibration factor**	None

Figure A-53 - Causal structure of risk factor associations and disease dependencies for incident hypertension

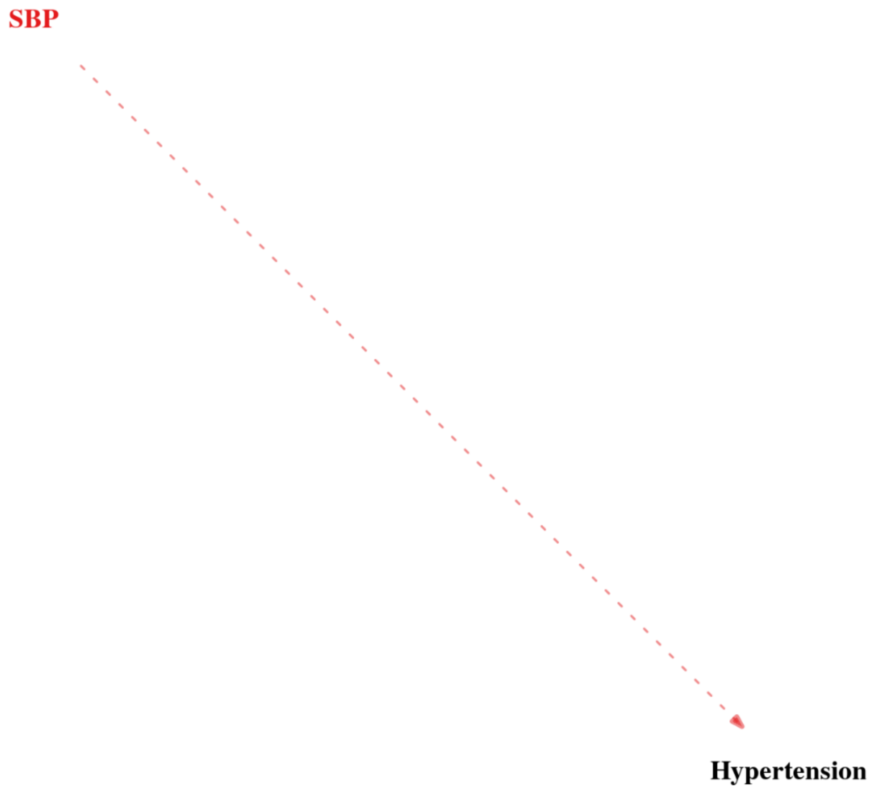

Table A-26 - Data sources for causal associations between risk factors and hypertension incidence

Parameter	Details	Comments	Source
Relative risk for systolic blood pressure	Own calculation of RRs from HSE data		Directly estimated from Health Survey for England 2003-2014

Validation plots for hypertension incidence and prevalence

Figure A-54 - Validation plot for modelled hypertension incidence (age-sex-dimd standardised)

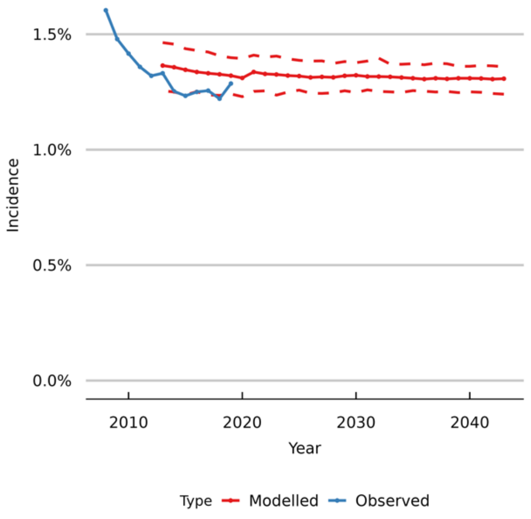

Figure A-55 - Validation plot for modelled hypertension prevalence (age-sex-dimd standardised)

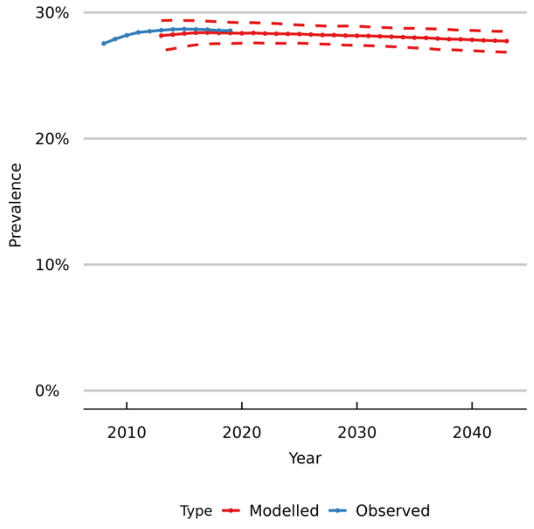

Asthma

Table A-27 - Modelling assumptions for incident asthma

Component	Assumptions / Details
**Disease type**	Spell – duration is stochastic; can recur
**Risk factor associations**	Smoking, body mass index
**Disease dependencies**	Past asthma
**Calibration factor**	Trend: 1.01

Figure A-56 - Causal structure of risk factor associations and disease dependencies for incident asthma

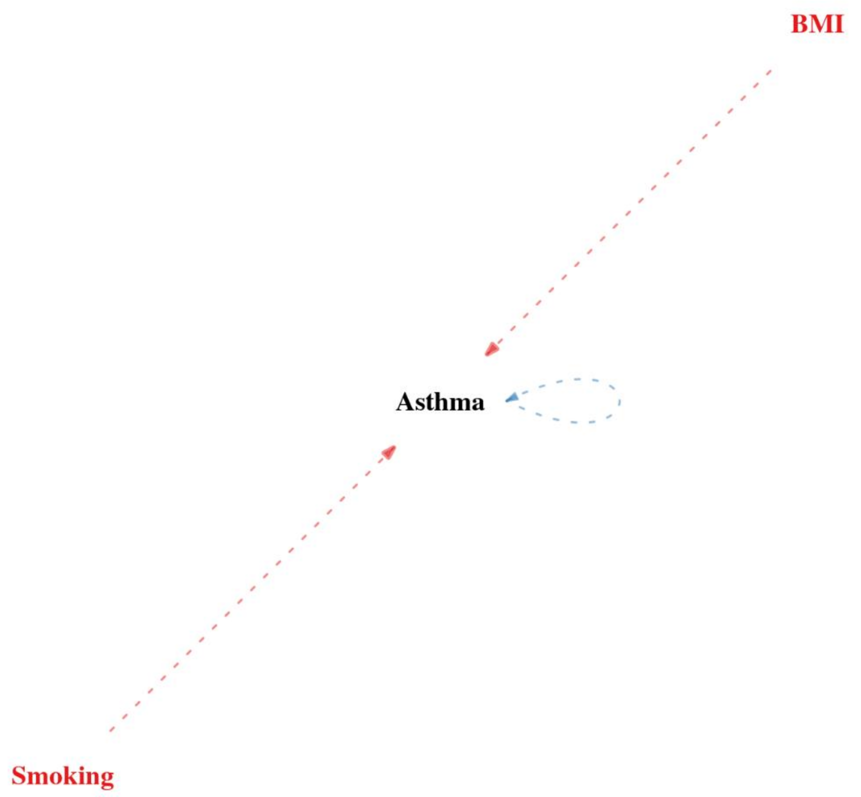

Table A-28 - Data sources for causal associations between risk factors and asthma incidence

Parameter	Details	Comments	Source
Relative risk for active smoking	GBD meta-analysis	RR from the GBD 2019 study	Global Burden of Disease Study 2019 (GBD 2019) Data Resources | GHDx [Internet]. [cited 2022 Mar 16];Available from: http://ghdx.healthdata.org/record/ihme-data/gbd-2019-relative-risks
Relative risk for ex-smoking	GBD meta-analysis	RR from the GBD 2019 study	Global Burden of Disease Study 2019 (GBD 2019) Data Resources | GHDx [Internet]. [cited 2022 Mar 16];Available from: http://ghdx.healthdata.org/record/ihme-data/gbd-2019-relative-risks
Relative risk for body mass index	GBD meta-analysis	RR from the GBD 2019 study	Global Burden of Disease Study 2019 (GBD 2019) Data Resources | GHDx [Internet]. [cited 2022 Mar 16];Available from: http://ghdx.healthdata.org/record/ihme-data/gbd-2019-relative-risks

Validation plots for asthma incidence and prevalence

Figure A-57 - Validation plot for modelled asthma incidence (age-sex-dimd standardised)

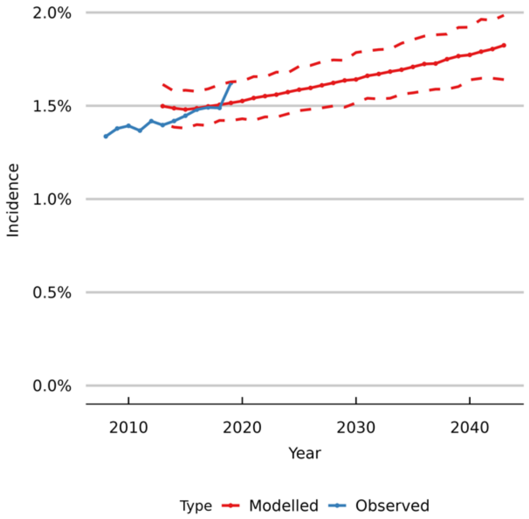

Figure A-58 - Validation plot for modelled asthma prevalence (age-sex-dimd standardised)

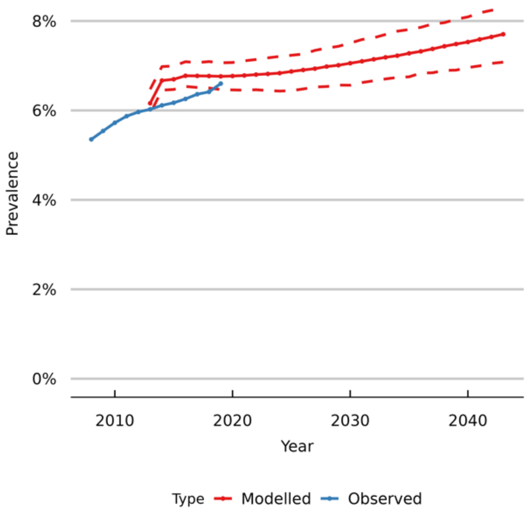

Alcohol problems

Table A-29 - Modelling assumptions for incident alcohol problems

Component	Assumptions / Details
**Disease type**	Spell – duration is stochastic; can recur
**Risk factor associations**	None
**Disease dependencies**	Past alcohol problems
**Calibration factor**	None

Figure A-59 - Causal structure of risk factor associations and disease dependencies for incident alcohol problems

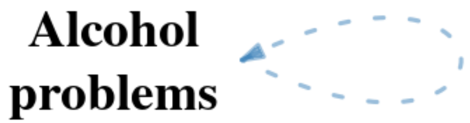

Validation plots for alcohol problems incidence and prevalence

Figure A-60 - Validation plot for modelled alcohol problems incidence (age-sex-dimd standardised)

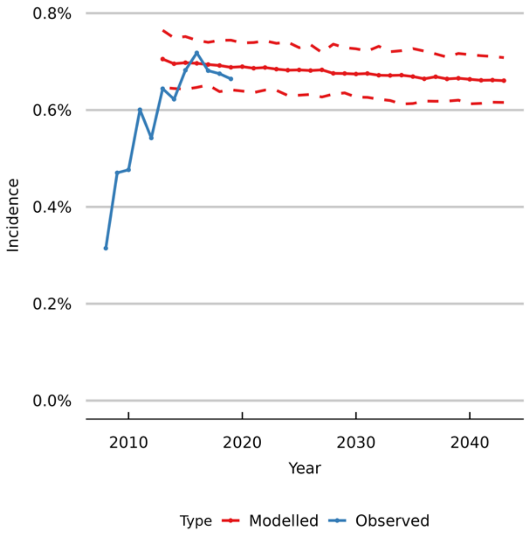

Figure A-61 - Validation plot for modelled alcohol problems prevalence (age-sex-dimd standardised)

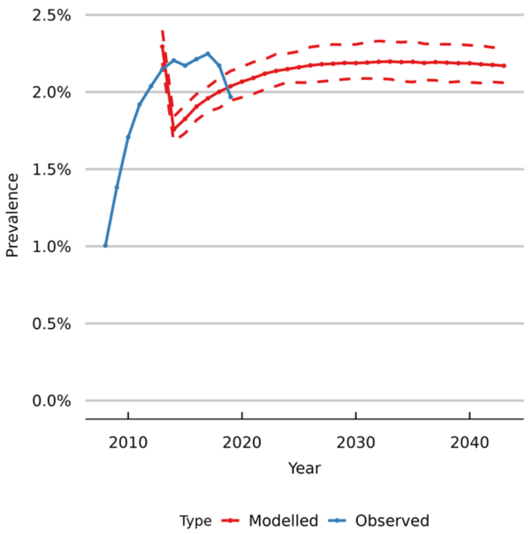

Anxiety and depression

Table A-30 - Modelling assumptions for incident anxiety and depression

Component	Assumptions / Details
**Disease type**	Spell – duration is stochastic; can recur
**Risk factor associations**	None
**Disease dependencies**	Past anxiety and depression, lung cancer, prostate cancer, other cancers, stroke, pain
**Calibration factor**	Intercept: 1.2<br><br>We did not calibrate to the observed incidence rate trends because their projections led to implausible rates.

Figure A-62 - Causal structure of risk factor associations and disease dependencies for incident anxiety and depression

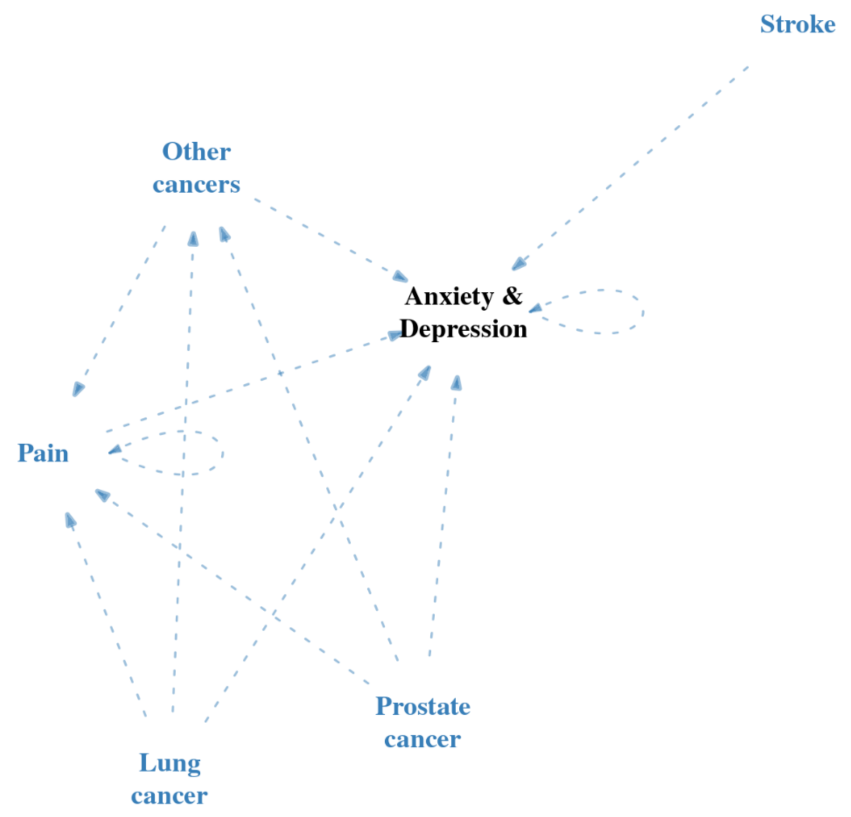

Validation plots for anxiety and depression incidence and prevalence

Figure A-63 - Validation plot for modelled anxiety and depression incidence (age-sex-dimd standardised)

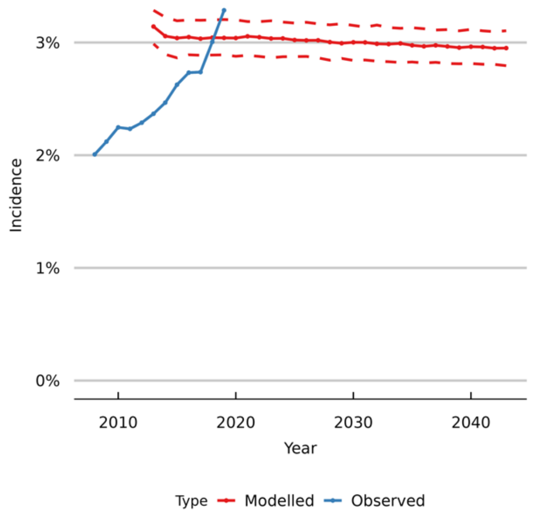

Figure A-64 - Validation plot for modelled anxiety and depression prevalence (age-sex-dimd standardised)

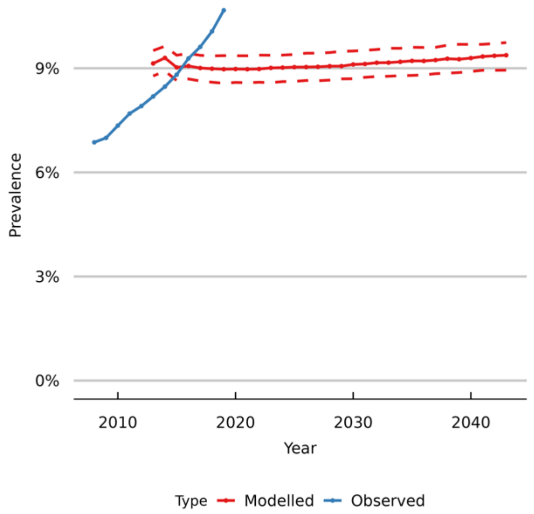

Constipation

Table A-31 - Modelling assumptions for incident constipation

Component	Assumptions / Details
**Disease type**	Spell – duration is stochastic; can recur
**Risk factor associations**	None
**Disease dependencies**	Past constipation, anxiety and depression, breast cancer, colorectal cancer, lung cancer, prostate cancer, other cancers, stroke, pain, alcohol problems, IBS, epilepsy, dementia, type 1 diabetes mellitus, type 2 diabetes mellitus, hypertension, psychosis, heart failure, CHD, COPD, atrial fibrillation, asthma, hearing loss
**Calibration factor**	None.<br><br>We did not calibrate to the observed incidence rate trends because their projections led to implausible rates.

Figure A-65 - Causal structure of risk factor associations and disease dependencies for incident constipation

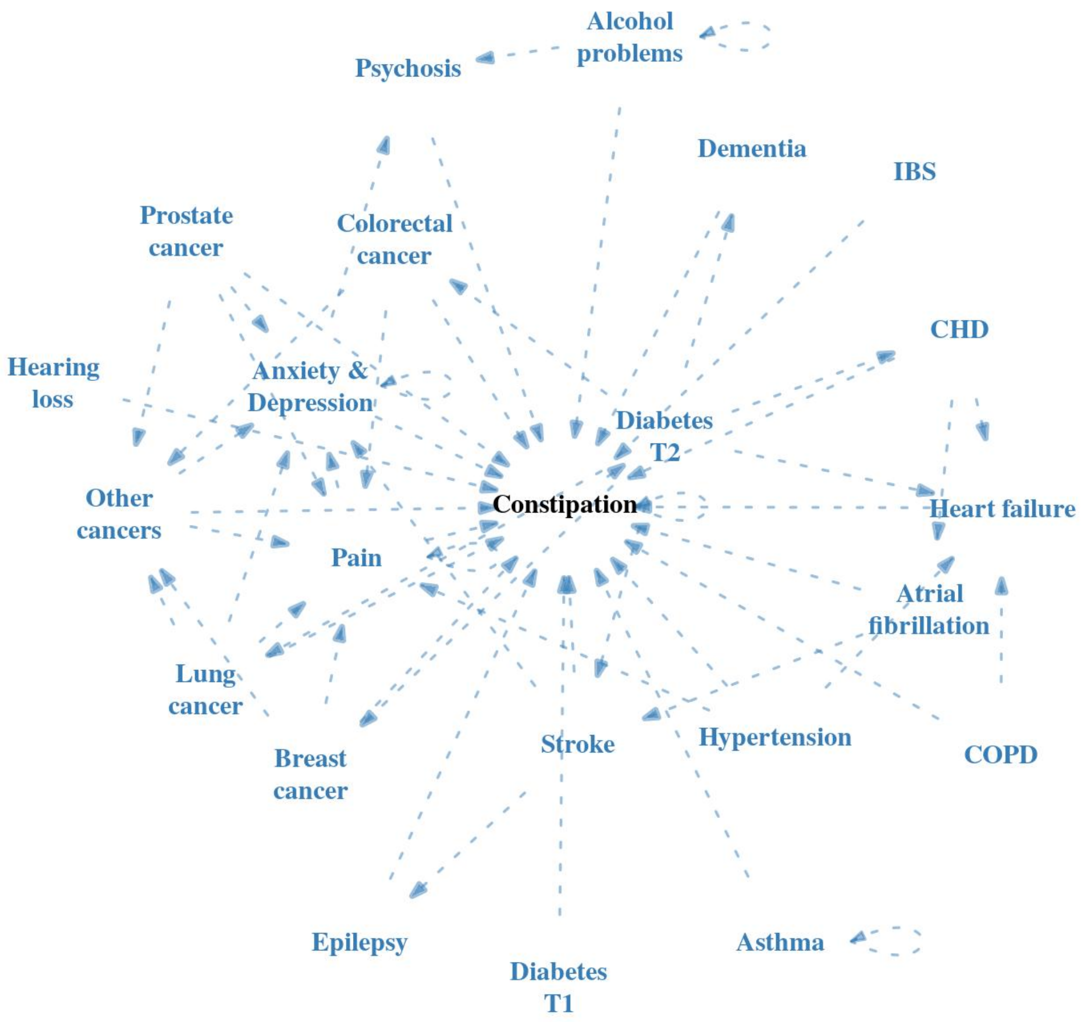

Validation plots for constipation incidence and prevalence

Figure A-66 - Validation plot for modelled constipation incidence (age-sex-dimd standardised)

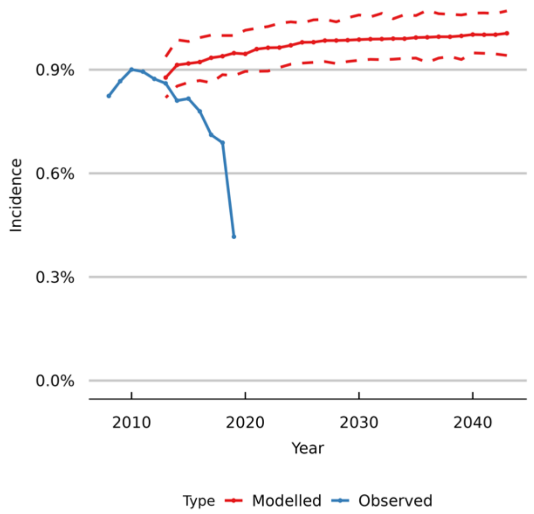

Figure A-67 - Validation plot for modelled constipation prevalence (age-sex-dimd standardised)

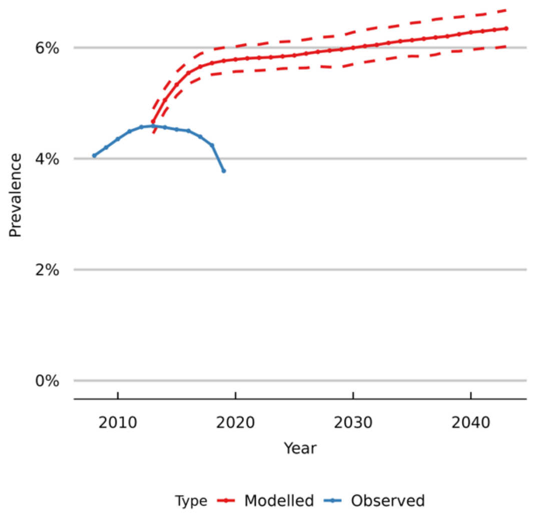

Pain

Table A-32 - Modelling assumptions for incident pain

Component	Assumptions / Details
**Disease type**	Spell – duration is stochastic; can recur
**Risk factor associations**	None
**Disease dependencies**	Past pain, connective tissue disorders, rheumatoid arthritis, breast cancer, colorectal cancer, prostate cancer, other cancers, CKD, hypertension, lung cancer
**Calibration factor**	Intercept: 0.9
**Other details**	Due to data quality issues over time with the prescription data, we modelled the incidence of pain based on the incidence in 2013

Figure A-68 - Causal structure of risk factor associations and disease dependencies for incident pain

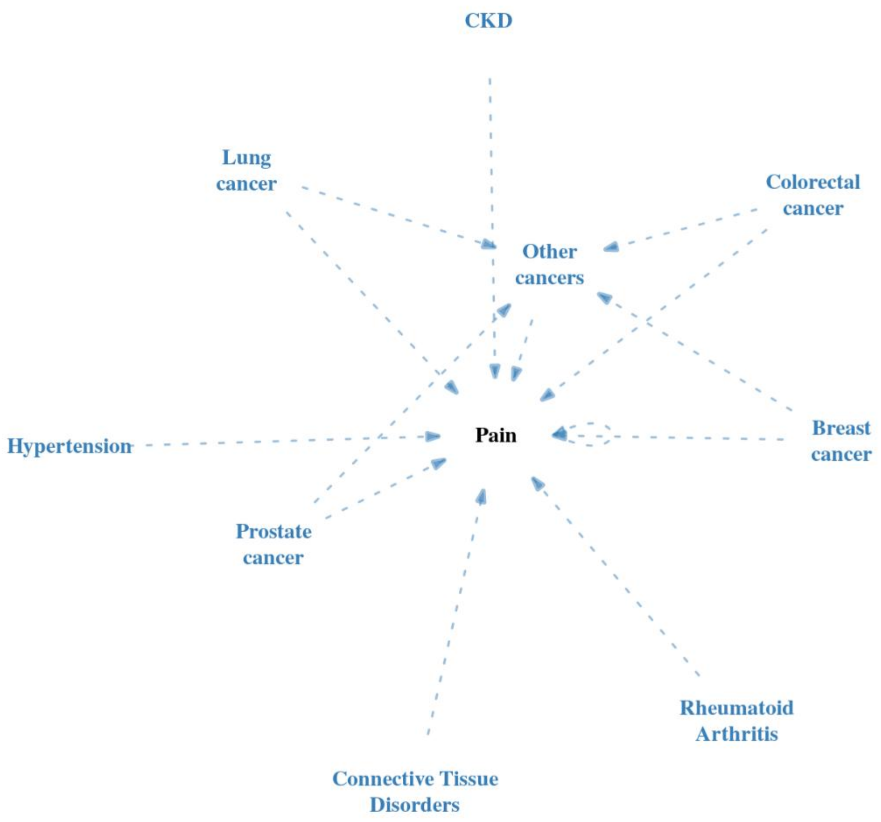

Validation plots for pain incidence and prevalence

Figure A-69 - Validation plot for modelled pain incidence (age-sex-dimd standardised)

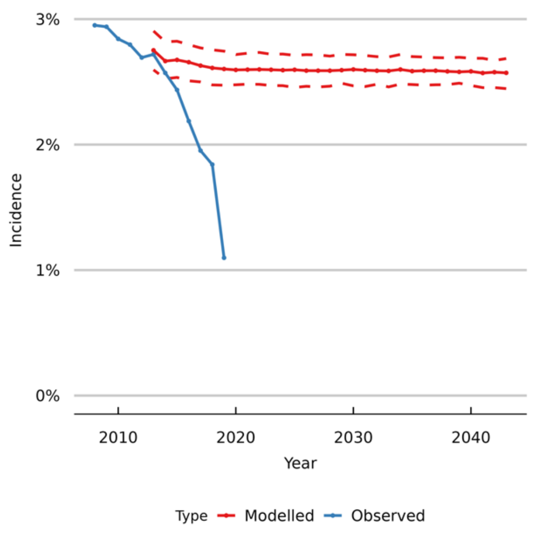

Figure A-70 - Validation plot for modelled pain prevalence (age-sex-dimd standardised)

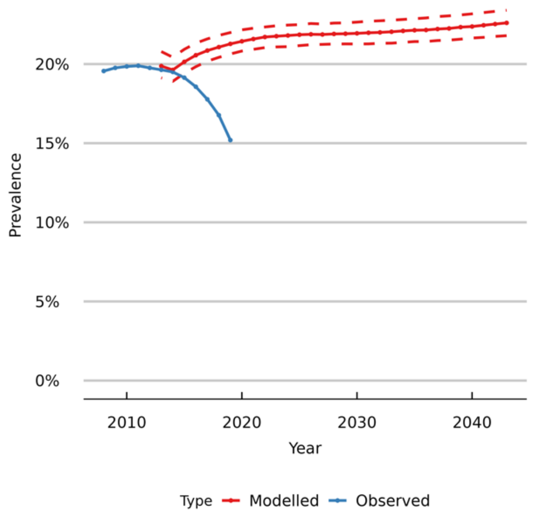

Psychosis

Table A-33 - Modelling assumptions for incident psychosiss

Component	Assumptions / Details
**Disease type**	Chronic – no recovery
**Risk factor associations**	None
**Disease dependencies**	Alcohol problems, anxiety and depression
**Calibration factor**	Intercept = 0.92

Figure A-71 - Causal structure of risk factor associations and disease dependencies for incident psychosis

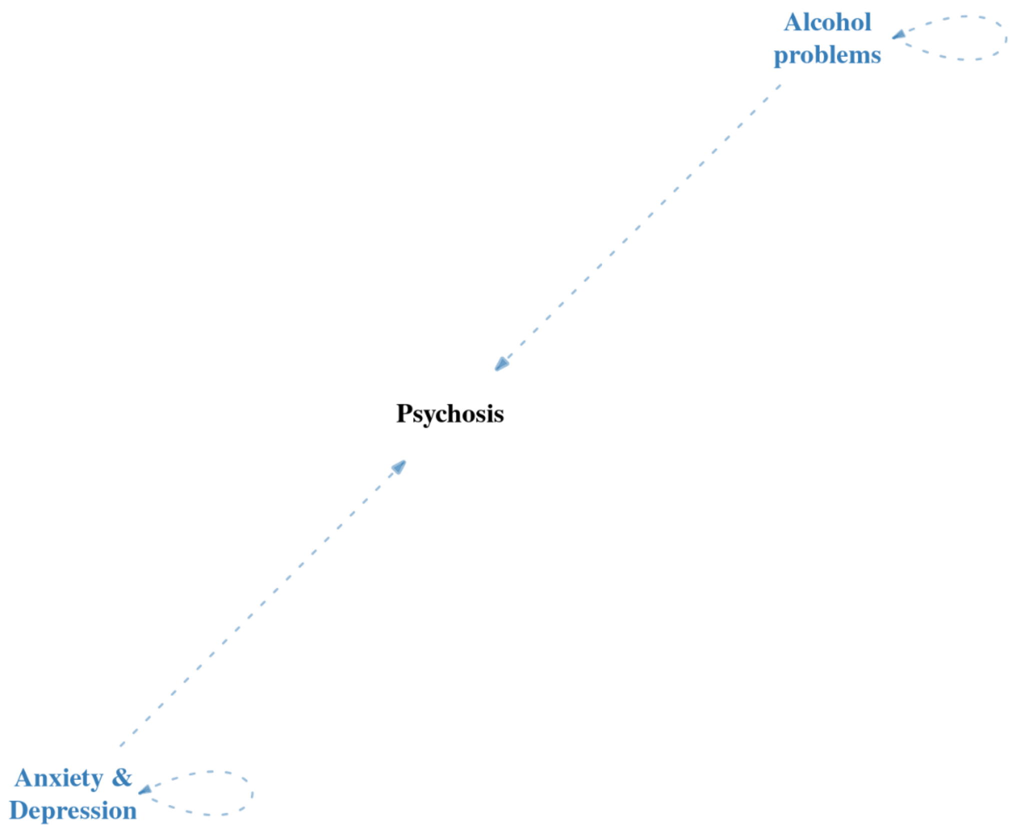

Validation plots for psychosis incidence and prevalence

Figure A-72 - Validation plot for modelled psychosis incidence (age-sex-dimd standardised)

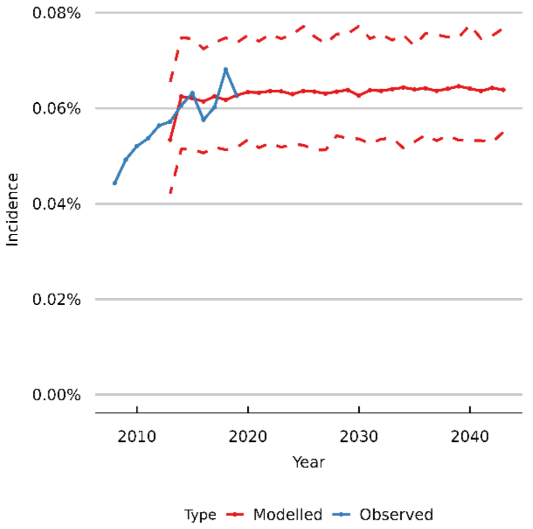

Figure A-73 - Validation plot for modelled psychosis prevalence (age-sex-dimd standardised)

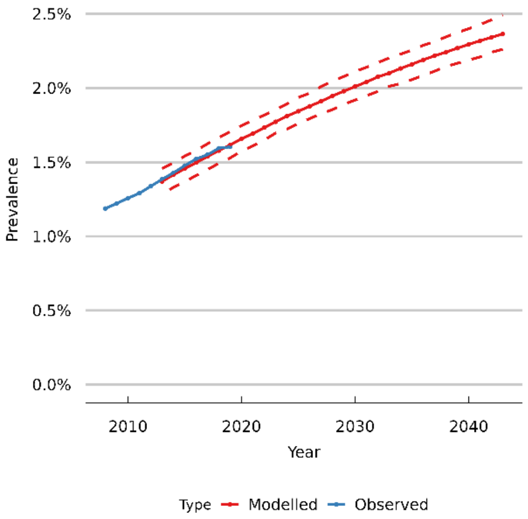

Epilepsy

Table A-34 - Modelling assumptions for incident epilepsy

Component	Assumptions / Details
**Disease type**	Chronic – no recovery
**Risk factor associations**	None
**Disease dependencies**	Stroke
**Calibration factor**	None

Figure A-74 - Causal structure of risk factor associations and disease dependencies for incident epilepsy

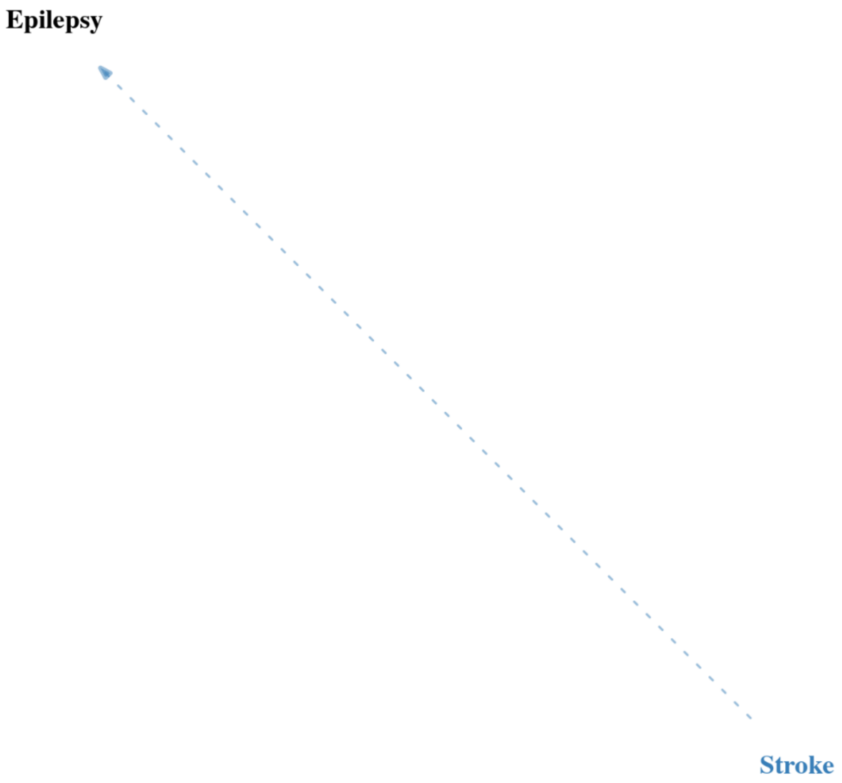

Validation plots for epilepsy incidence, case fatality, and prevalence

Figure A-75 - Validation plot for modelled epilepsy incidence (age-sex-dimd standardised)

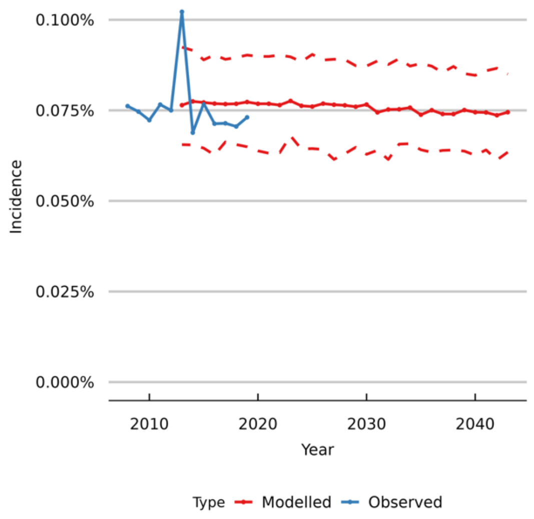

Figure A-76 - Validation plot for modelled epilepsy case fatality (age-sex-dimd standardised)

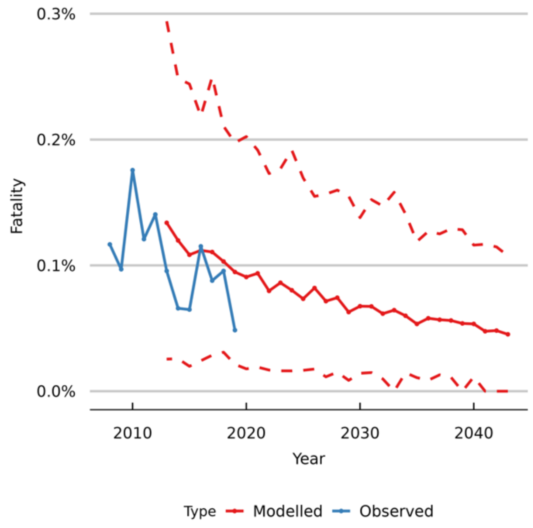

Figure A-77 - Validation plot for modelled epilepsy prevalence (age-sex-dimd standardised)

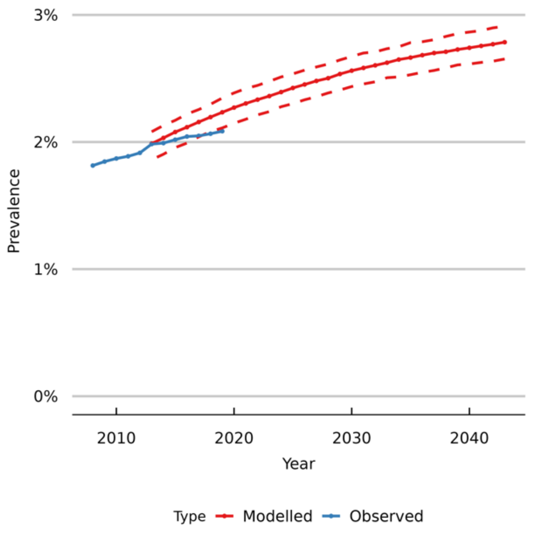

Rheumatoid arthritis

Table A-35 - Modelling assumptions for incident rheumatoid arthritis

Component	Assumptions / Details
**Disease type**	Chronic – no recovery
**Risk factor associations**	None
**Disease dependencies**	None
**Calibration factor**	Intercept: 0.95

Validation plots for rheumatoid arthritis incidence, case fatality, and prevalence

Figure A-78 - Validation plot for modelled rheumatoid arthritis incidence (age-sex-dimd standardised)

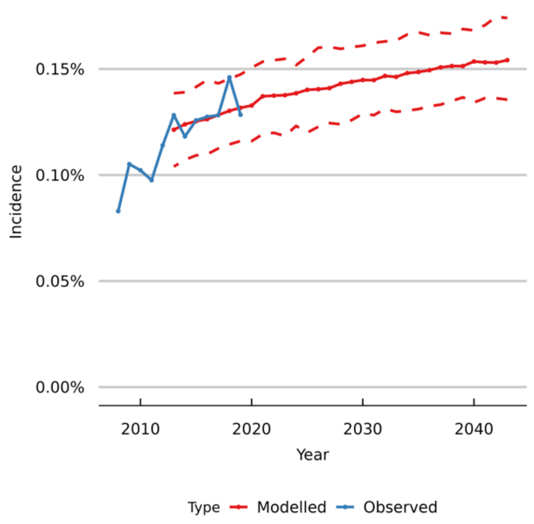

Figure A-79 - Validation plot for modelled rheumatoid arthritis case fatality (age-sex-dimd standardised)

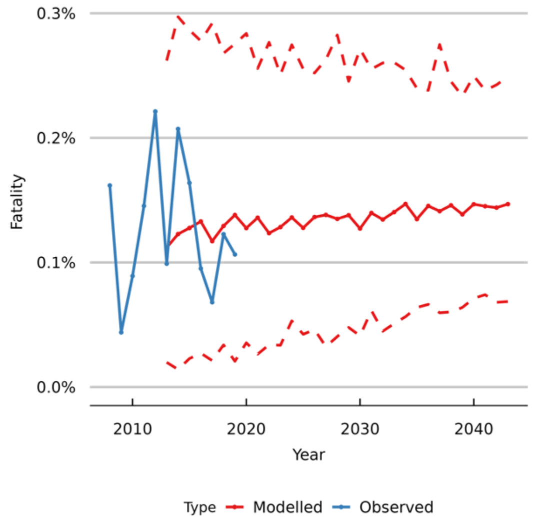

Figure A-80 - Validation plot for modelled rheumatoid arthritis prevalence (age-sex-dimd standardised)

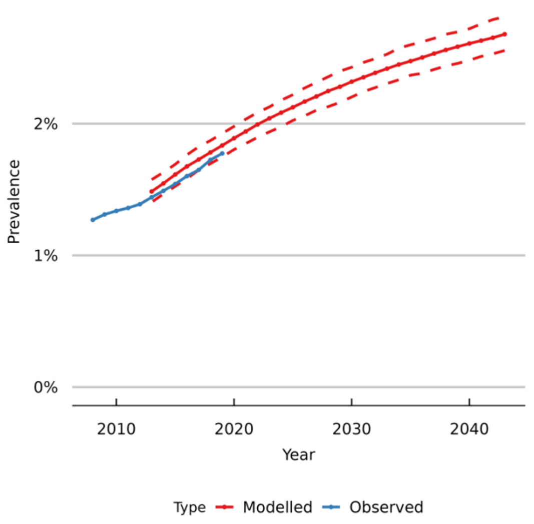

Connective tissue disorders

Table A-36 - Modelling assumptions for incident connective tissue disorders

Component	Assumptions / Details
**Disease type**	Chronic – no recovery; all connective tissue disorders, excluding rheumatoid arthritis
**Risk factor associations**	None
**Disease dependencies**	None
**Calibration factor**	Trend: 0.999

Validation plots for connective tissue disorders incidence, case fatality, and prevalence

Figure A-81 - Validation plot for modelled connective tissue disorders incidence (age-sex-dimd standardised)

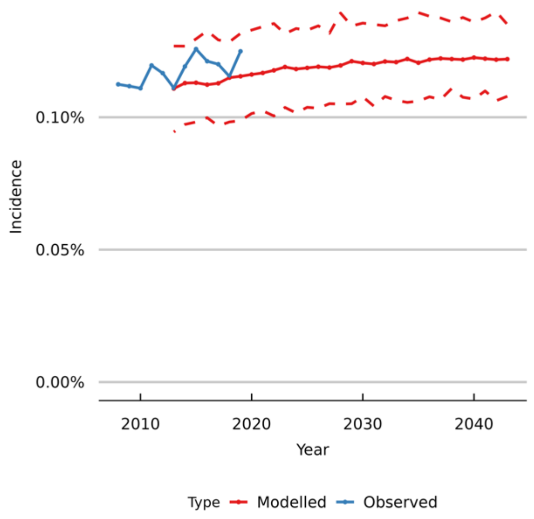

Figure A-82 - Validation plot for modelled connective tissue disorders case fatality (age-sex-dimd standardised)

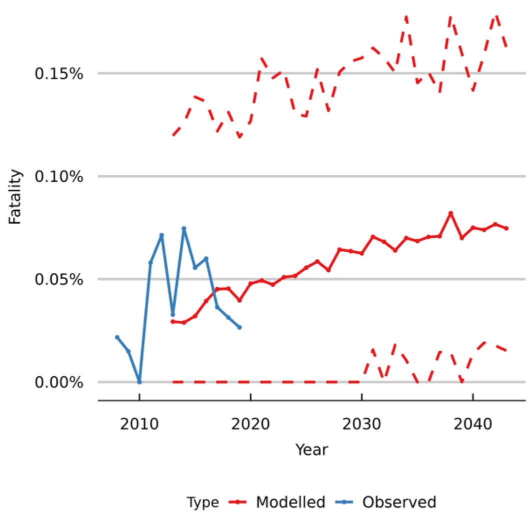

Figure A-83 - Validation plot for modelled connective tissue disorders prevalence (age-sex-dimd standardised)

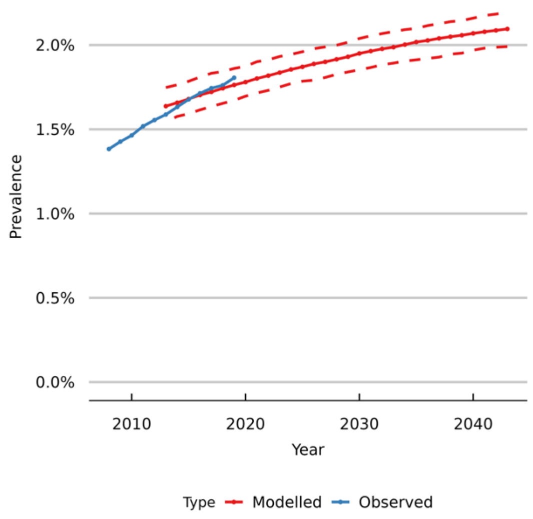

Type 1 Diabetes Mellitus

Table A-37 - Modelling assumptions for incident type 1 diabetes mellitus

Component	Assumptions / Details
**Disease type**	Chronic – no recovery
**Risk factor associations**	None
**Disease dependencies**	None
**Calibration factor**	None

Validation plots for type 1 diabetes mellitus incidence, case fatality, and prevalence

Figure A-84 - Validation plot for modelled type 1 diabetes mellitus incidence (age-sex-dimd standardised)

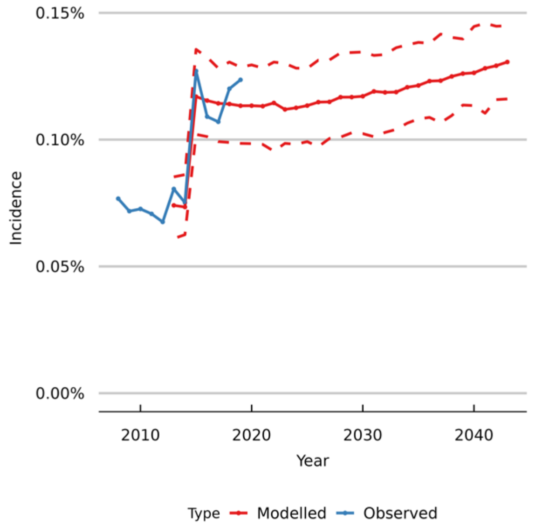

Figure A-85 - Validation plot for modelled type 1 diabetes mellitus case fatality (age-sex-dimd standardised)

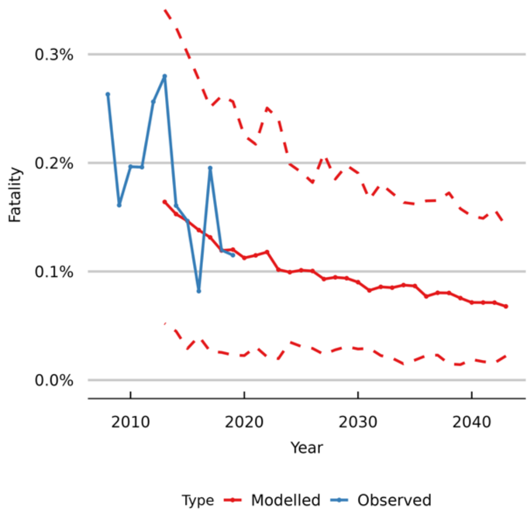

Figure A-86 - Validation plot for modelled type 1 diabetes mellitus prevalence (age-sex-dimd standardised)

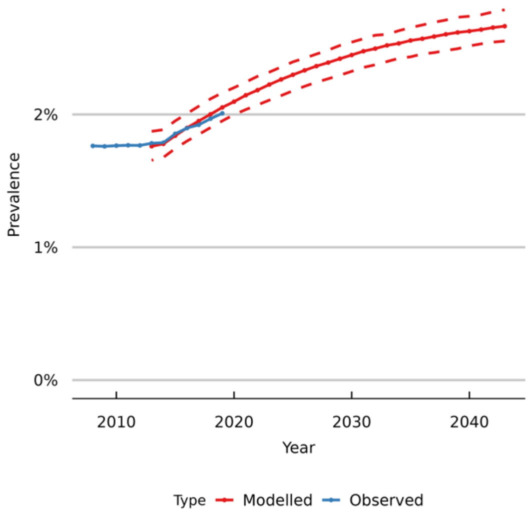

Hearing loss

Table A-38 - Modelling assumptions for incident hearing loss

Component	Assumptions / Details
**Disease type**	Chronic – no recovery
**Risk factor associations**	None
**Disease dependencies**	None
**Calibration factor**	Intercept: 0.95

Validation plots for hearing loss incidence and prevalence

Figure A-87 - Validation plot for modelled hearing loss incidence (age-sex-dimd standardised)

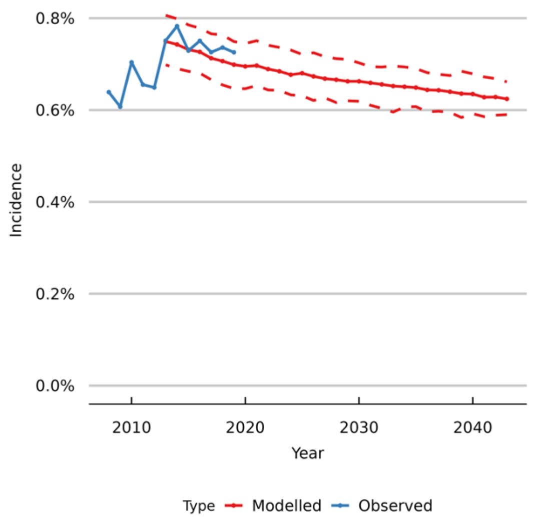

Figure A-88 - Validation plot for modelled hearing loss prevalence (age-sex-dimd standardised)

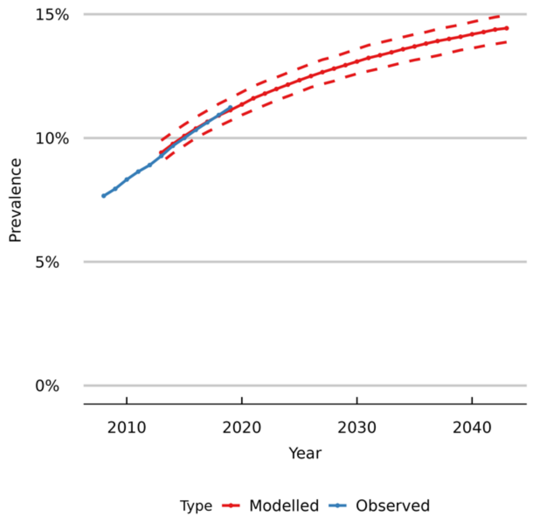

Irritable bowel syndrome (IBS)

Table A-39 - Modelling assumptions for incident IBS

Component	Assumptions / Details
**Disease type**	Chronic – no recovery
**Risk factor associations**	None
**Disease dependencies**	None
**Calibration factor**	Trend: 0.99

Validation plots for IBS incidence and prevalence

Figure A-89 - Validation plot for modelled IBS incidence (age-sex-dimd standardised)

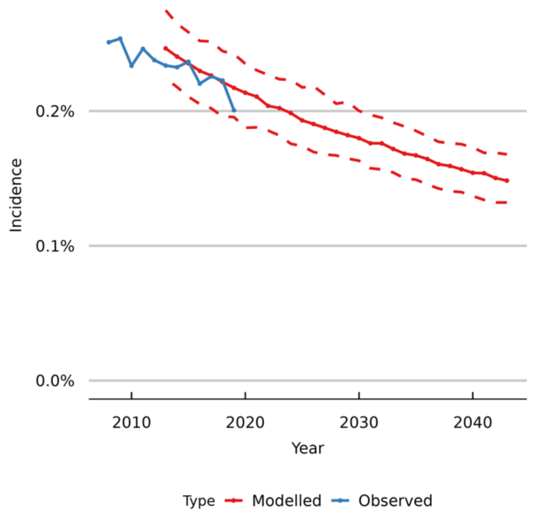

Figure A-90 - Validation plot for modelled IBS prevalence (age-sex-dimd standardised)

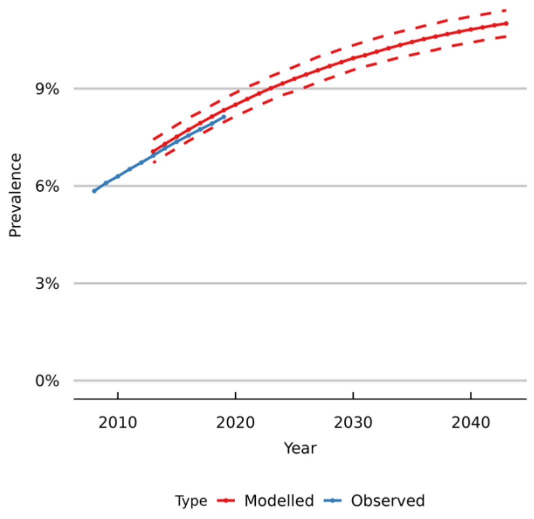

Non-modelled mortality

Table A-40 - Modelling assumptions for mortality for causes other than the case fatalities described above

Component	Assumptions / Details
**Outcome type**	Mortality from any cause other than the specifically modelled case fatality relationships described above
**Risk factor associations**	Smoking, systolic blood pressure, alcohol intake, physical activity
**Disease dependencies**	All 20 CMS conditions
**Calibration factor**	None

Figure A-91 - Causal structure of risk factor associations and disease dependencies for incident non-modelled mortality

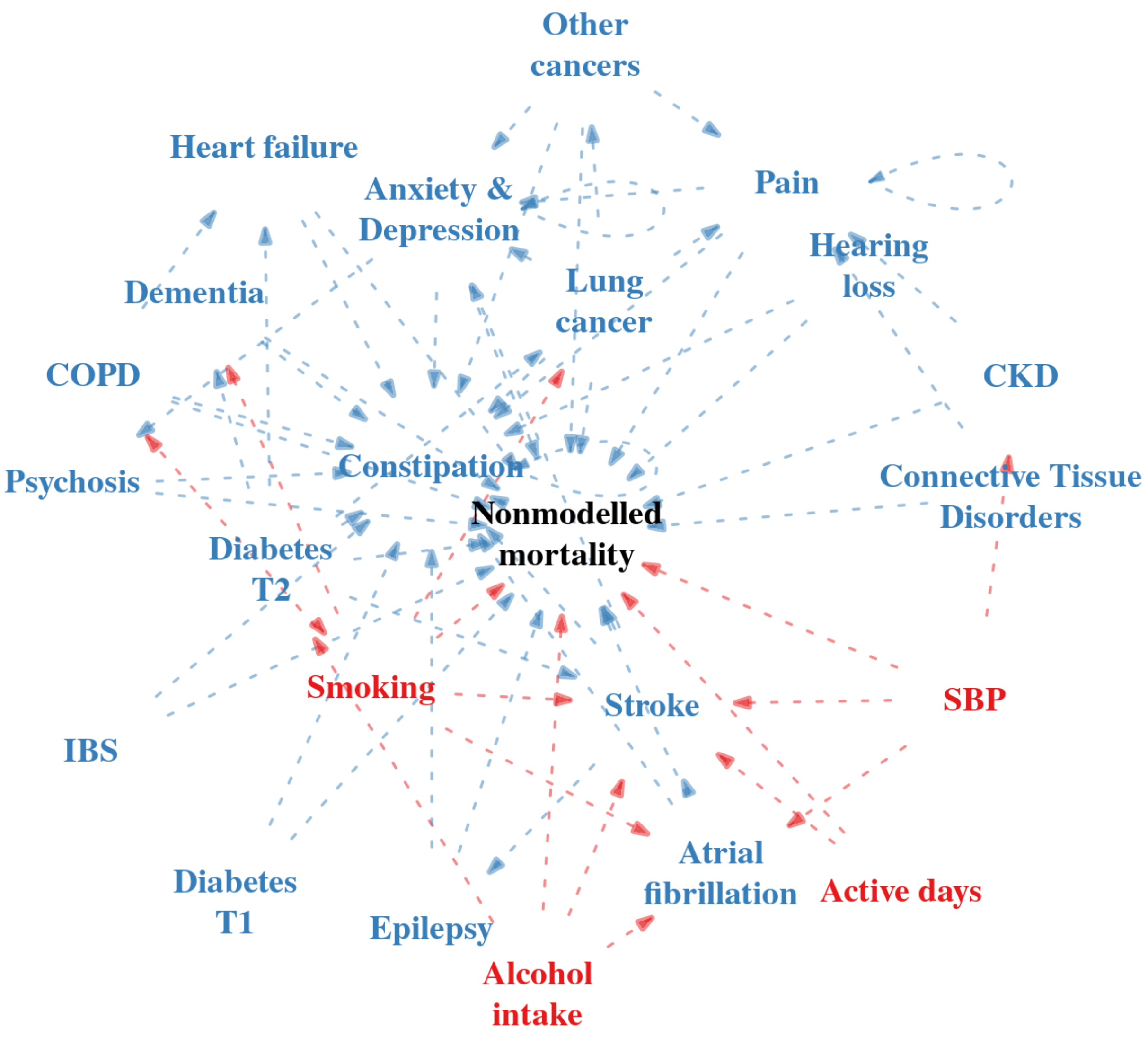

Table A-41 - Data sources for causal associations between risk factors and non-modelled mortality

Parameter	Details	Comments	Source
Relative risk for smoking status	Meta-analysis of 1.7 million men and women	Multiply adjusted. We used the Non-CVD, non-cancer mortality effects.	Stringhini S, Carmeli C, Jokela M, Avendaño M, Muennig P, Guida F, et al. Socioeconomic status and the 25 × 25 risk factors as determinants of premature mortality: a multicohort study and meta-analysis of 1·7 million men and women. The Lancet 2017;389:1229–37. (Figure 4)
Relative risk for systolic blood pressure	Meta-analysis of 1.7 million men and women	Multiply adjusted. We used the Non-CVD, non-cancer mortality effects. We applied the effect to those with SBP > 140 mmHg	Stringhini S, Carmeli C, Jokela M, Avendaño M, Muennig P, Guida F, et al. Socioeconomic status and the 25 × 25 risk factors as determinants of premature mortality: a multicohort study and meta-analysis of 1·7 million men and women. The Lancet 2017;389:1229–37. (Figure 4)
Relative risk for physical activity	Meta-analysis of 1.7 million men and women	Multiply adjusted. We used the Non-CVD, non-cancer mortality effects. We applied the effect only to those with one or less active days per week.	Stringhini S, Carmeli C, Jokela M, Avendaño M, Muennig P, Guida F, et al. Socioeconomic status and the 25 × 25 risk factors as determinants of premature mortality: a multicohort study and meta-analysis of 1·7 million men and women. The Lancet 2017;389:1229–37. (Figure 4)
Relative risk for alcohol intake	Meta-analysis of 1.7 million men and women	Multiply adjusted. We used the Non-CVD, non-cancer mortality effects.	Stringhini S, Carmeli C, Jokela M, Avendaño M, Muennig P, Guida F, et al. Socioeconomic status and the 25 × 25 risk factors as determinants of premature mortality: a multicohort study and meta-

	analysis of 1·7 million men and women. The Lancet 2017;389:1229–37. (Figure 4)

Figure A-92- Validation plot for non-modelled mortality (age-sex-dimd standardised)

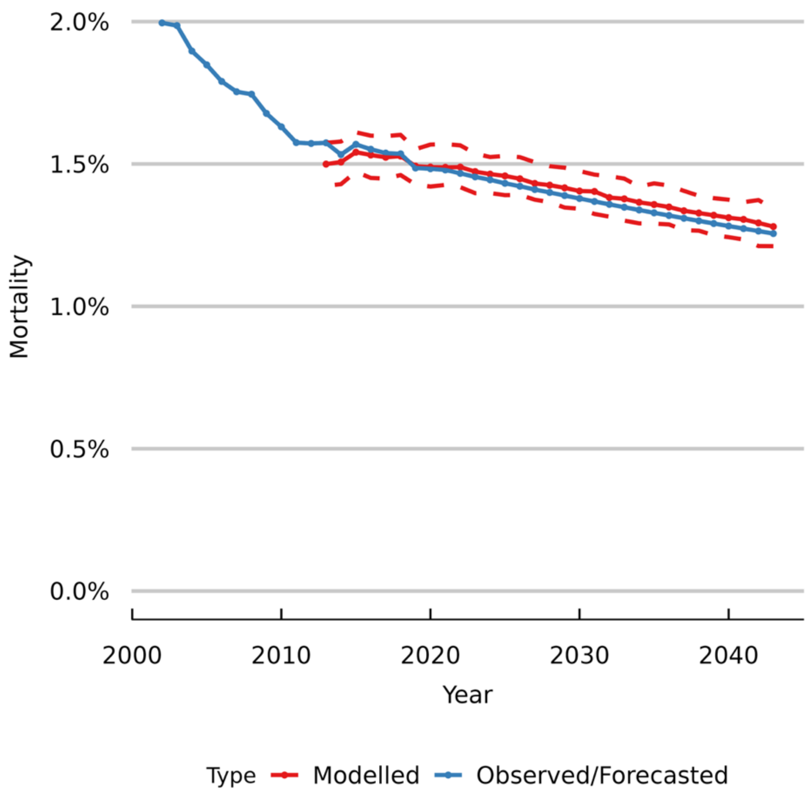

Cambridge Multimorbidity Score (CMS) Validation

Figure A-93 -Validation plot for prevalence of CMS > 0 (age-sex-dimd standardised)

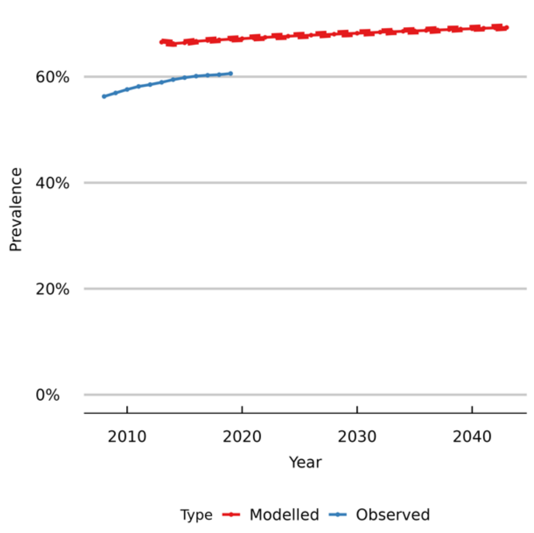

Figure A-94 - Validation plot for prevalence of CMS >= 2 (age-sex-dimd standardised)

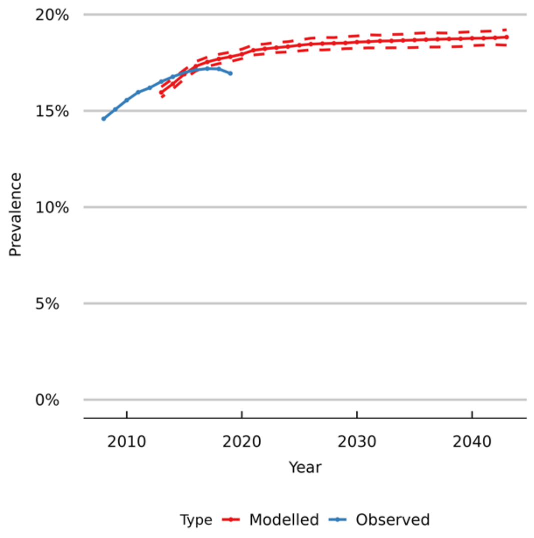

B) Appendix B: exposure modelling details and validation

Exposure modelling details

Table B-1 - Exposure modelling in IMPACT$_{NCD}$

Exposure	Statistical Modelling (distribution)	Independent Variables	Comment
Active days per week	Logit ordinal regression	Year, age, sex, QIMD, SHA, ethnicity	
Daily fruit consumption in grams	GAMLSS (Zero Inflated SICHEL)	Year, age, sex, QIMD, SHA, ethnicity	1 portion = 80g
Daily vegetable consumption in grams	GAMLSS (Delaporte)	Year, age, sex, QIMD, SHA, ethnicity	1 portion = 80g
Smoking status (never/ ex occasionally/ ex regularly /current)	GAMLSS (Multinomial with four categories)	Year, age, sex, QIMD, SHA, ethnicity	All the smoking-related variables are used in a smoking microsimulation subroutine that simulates smoking histories
Years of abstinence for ex-smokers	GAMLSS (double Poisson)	Year, age, sex, QIMD, SHA, ethnicity	Applies only to the first year that a synthetic individual enters the simulation. Then is estimated from the smoking subroutine

Exposure	Statistical Modelling (distribution)	Independent Variables	Comment
Smoking duration for ex-smokers	GAMLSS (double Poisson)	Year, age, sex, QIMD, SHA, ethnicity	Applies only to the first year that a synthetic individual enters the simulation. Then is estimated from the smoking subroutine
Smoking duration for current smokers	GAMLSS (negative binomial)	Year, age, sex, QIMD, SHA, ethnicity	Applies only to the first year that a synthetic individual enters the simulation. Then is estimated from the smoking subroutine
Smoking initiation probability	GAMLSS (binomial)	Year, age, sex, QIMD, SHA, ethnicity	
Smoking cessation probability	GAMLSS (binomial)	Year, age, sex, QIMD, SHA, ethnicity	
Smoking relapse probability	Exponential decay	Sex, QIMD, years since cessation	
Cigarettes per day for ex-smokers	GAMLSS (negative binomial)	Year, age, sex, QIMD, SHA, ethnicity	
Cigarettes per day for current smokers	GAMLSS (negative binomial)	Year, age, sex, QIMD, SHA, ethnicity	

Exposure	Statistical Modelling (distribution)	Independent Variables	Comment
Environmental tobacco smoking	GAMLSS (binomial)	Year, age, sex, QIMD, SHA, ethnicity	Currently, this is independent of smoking prevalence in an area
Ethanol consumption per day, based on average weekly consumption	GAMLSS (negative binomial)	Year, age, sex, QIMD, SHA, ethnicity, smoking status	Since HSE 2011
BMI	GAMLSS (Box-Cox Power Exponential)	Year, age, sex, QIMD, SHA, ethnicity, smoking status	
SBP	GAMLSS (Box-Cox Power Exponential)	Year, age, sex, QIMD, SHA, ethnicity, smoking status	
BP medication	GAMLSS (binomial)	Year, age, sex, QIMD, SHA, ethnicity, SBP	Since 2012
Total cholesterol	GAMLSS (Box-Cox t)	Year, age, sex, QIMD, SHA, ethnicity	
HDL to total cholesterol ratio	GAMLSS (generalized beta type 1)	Year, age, sex, QIMD, SHA, ethnicity	

Exposure	Statistical Modelling (distribution)	Independent Variables	Comment
Statins	GAMLSS (binomial)	Year, age, sex, QIMD, SHA, ethnicity, total cholesterol	Since 2012

Exposure validation plots

The following figures are the cumulative probability validation plots for the modelled exposures in the simulation compared to the observed Health Survey for England data. Plots are presented by 1) year and 10-year age-group, 2) year and quintiles of IMD.

For the main exposures modelled (alcohol, active days, BMI, fruit and vegetable intake, smoking prevalence, second-hand smoke exposure, SBP, total cholesterol), we also present trends in exposures by quintiles of IMD (unstandardised).

Active days

Figure B-1 - Validation: Active days – HSE and projected IMPACT$_{NCD}$ trends by quintile of IMD (unstandardised)

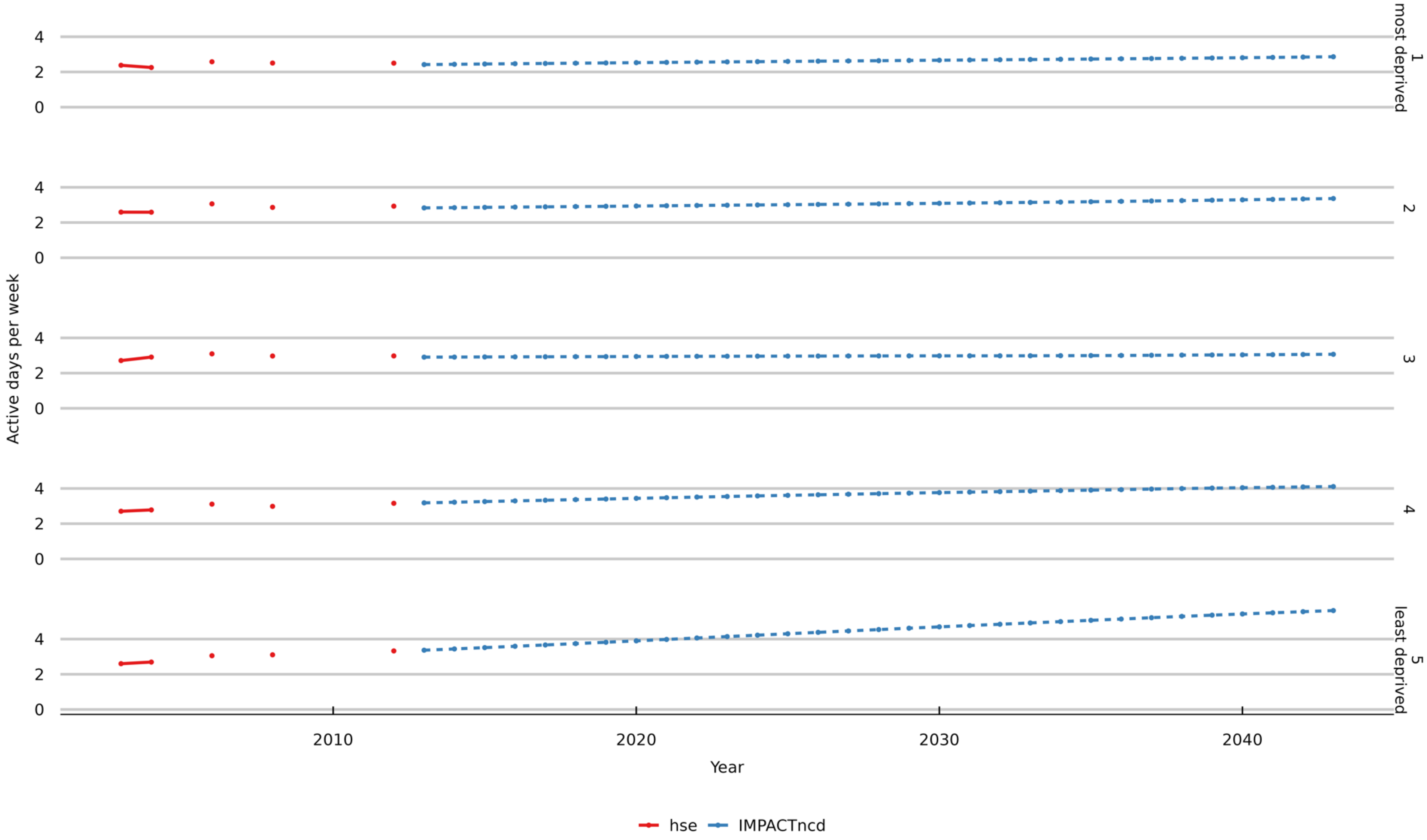

Figure B-2. Validation: Active days by year and age

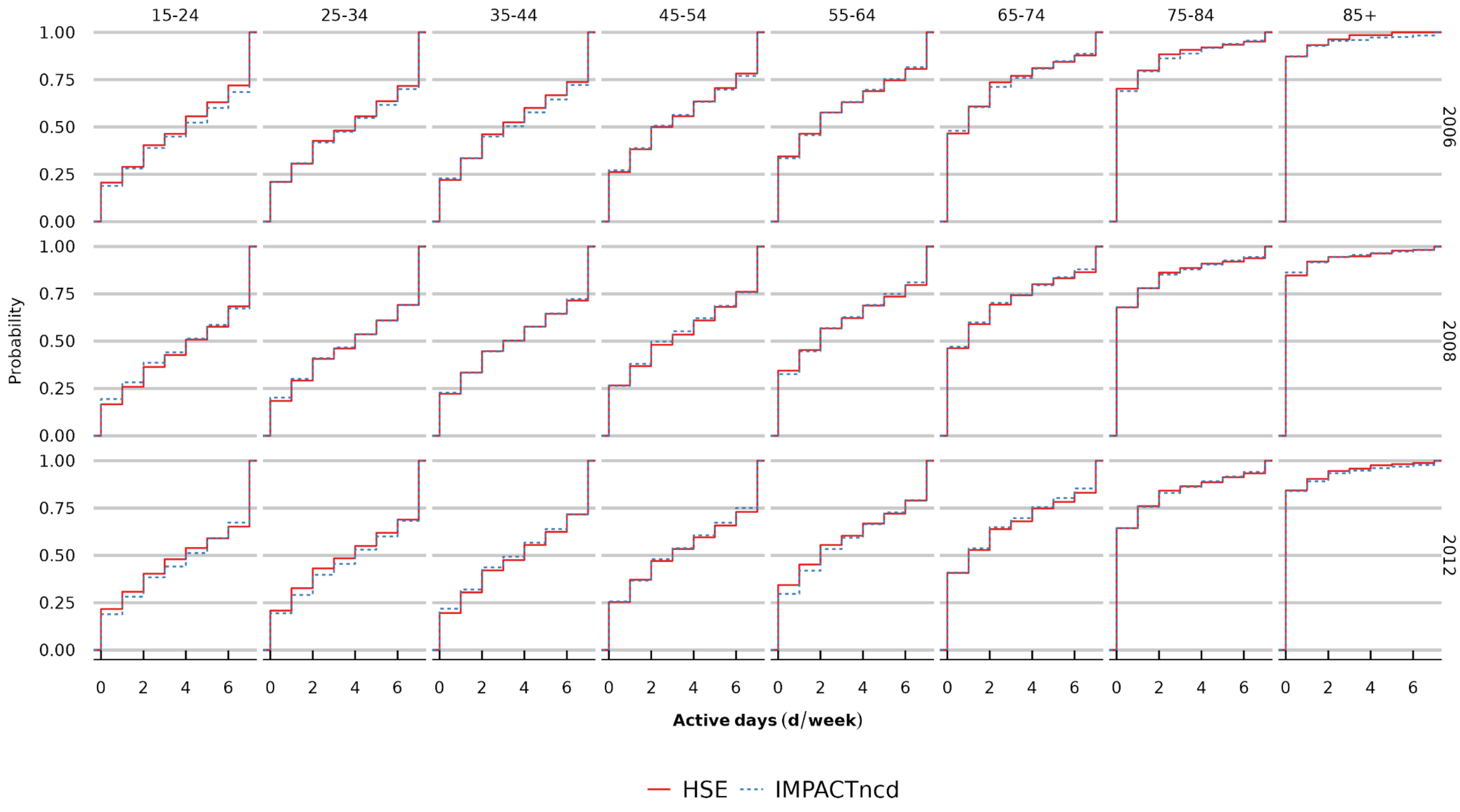

Figure B-3. Validation: Active days by quinitiles of IMD and age

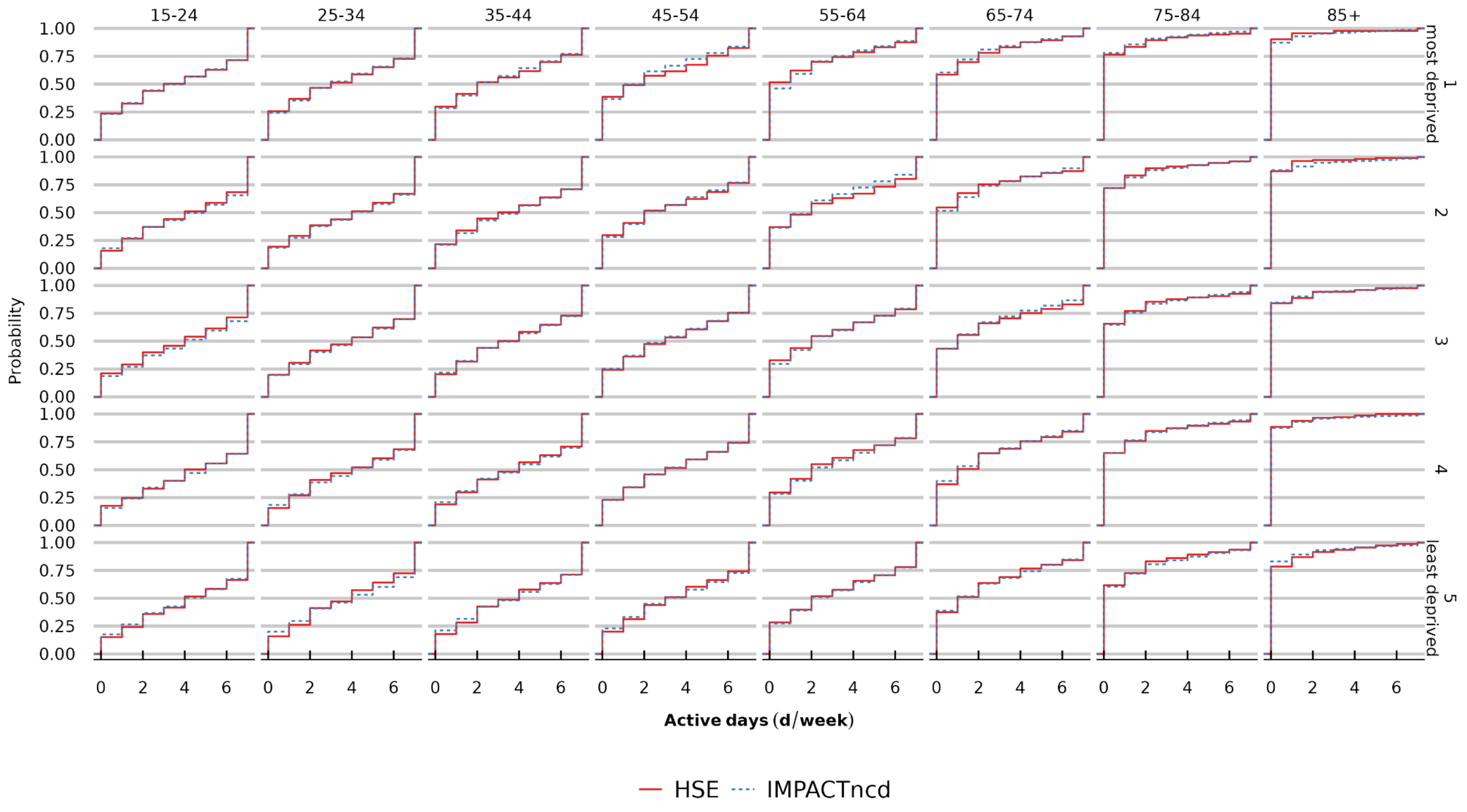

Alcohol intake

Figure B-4 - Validation: Alcohol intake – HSE and projected IMPACT$_{NCD}$ trends by quintile of IMD (unstandardised)

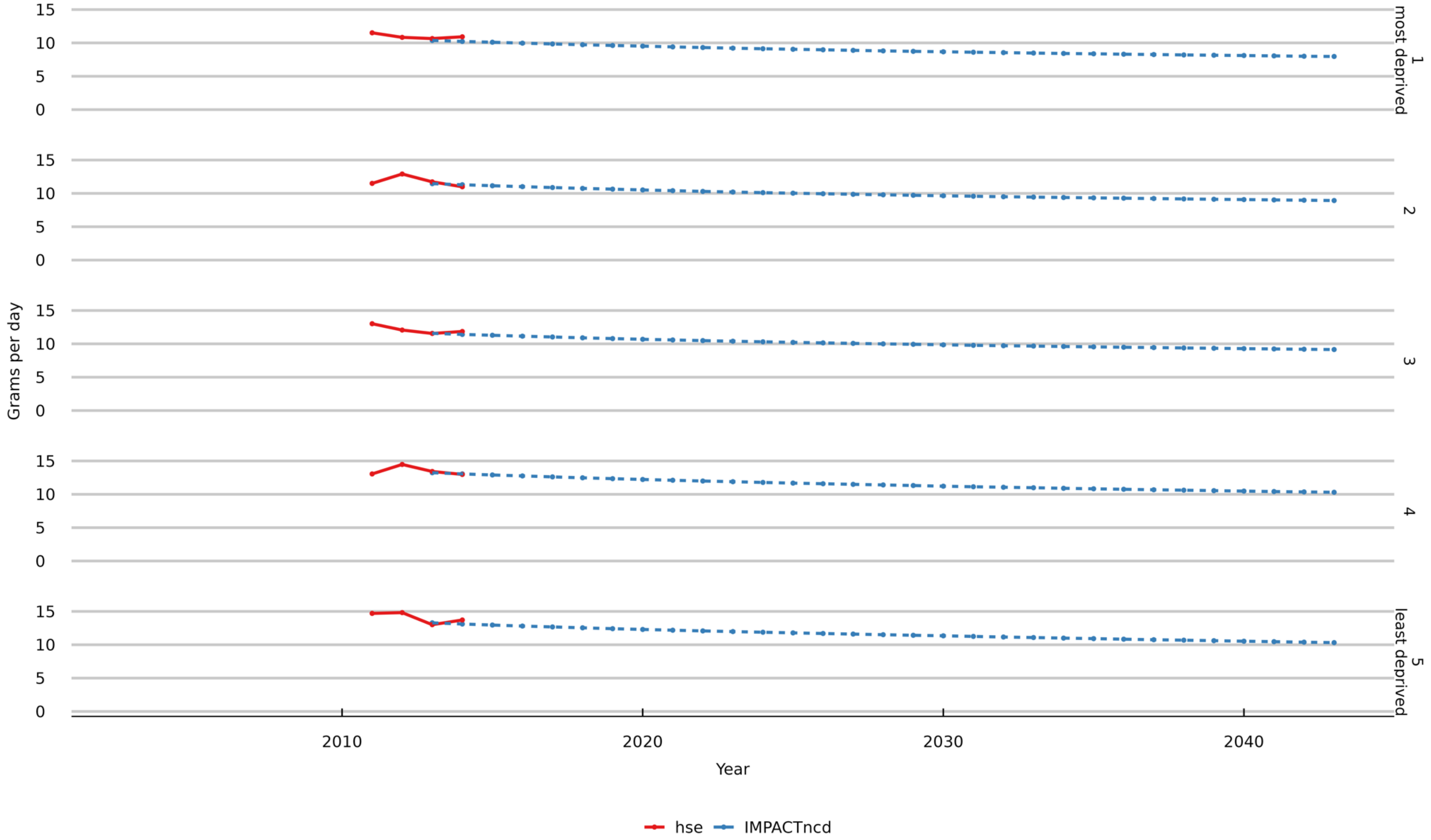

Figure B-5 - Validation: Alcohol intake (grams per day) by year and age

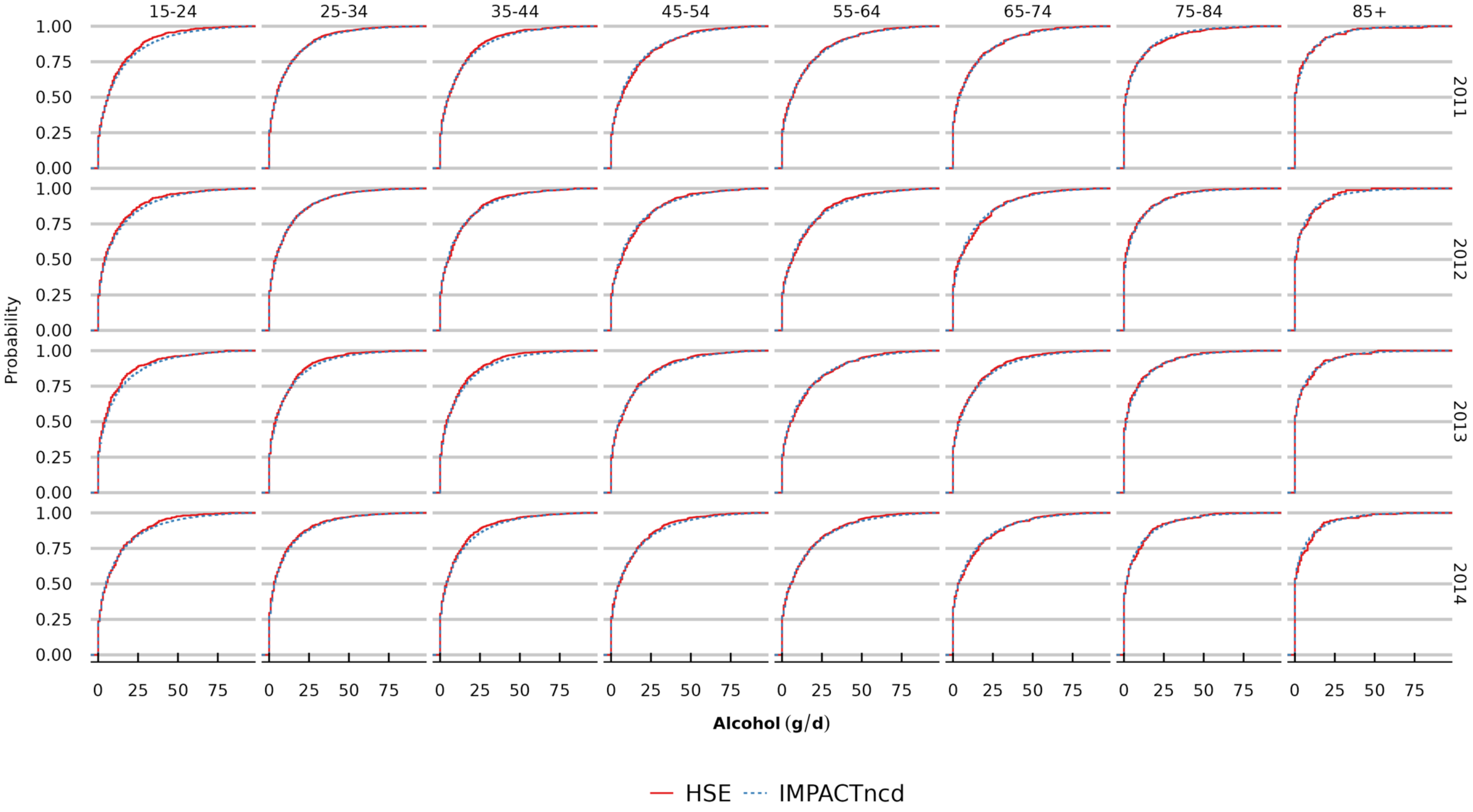

Figure B-6 - Validation: Alcohol intake (grams per day) by quintiles of IMD and age

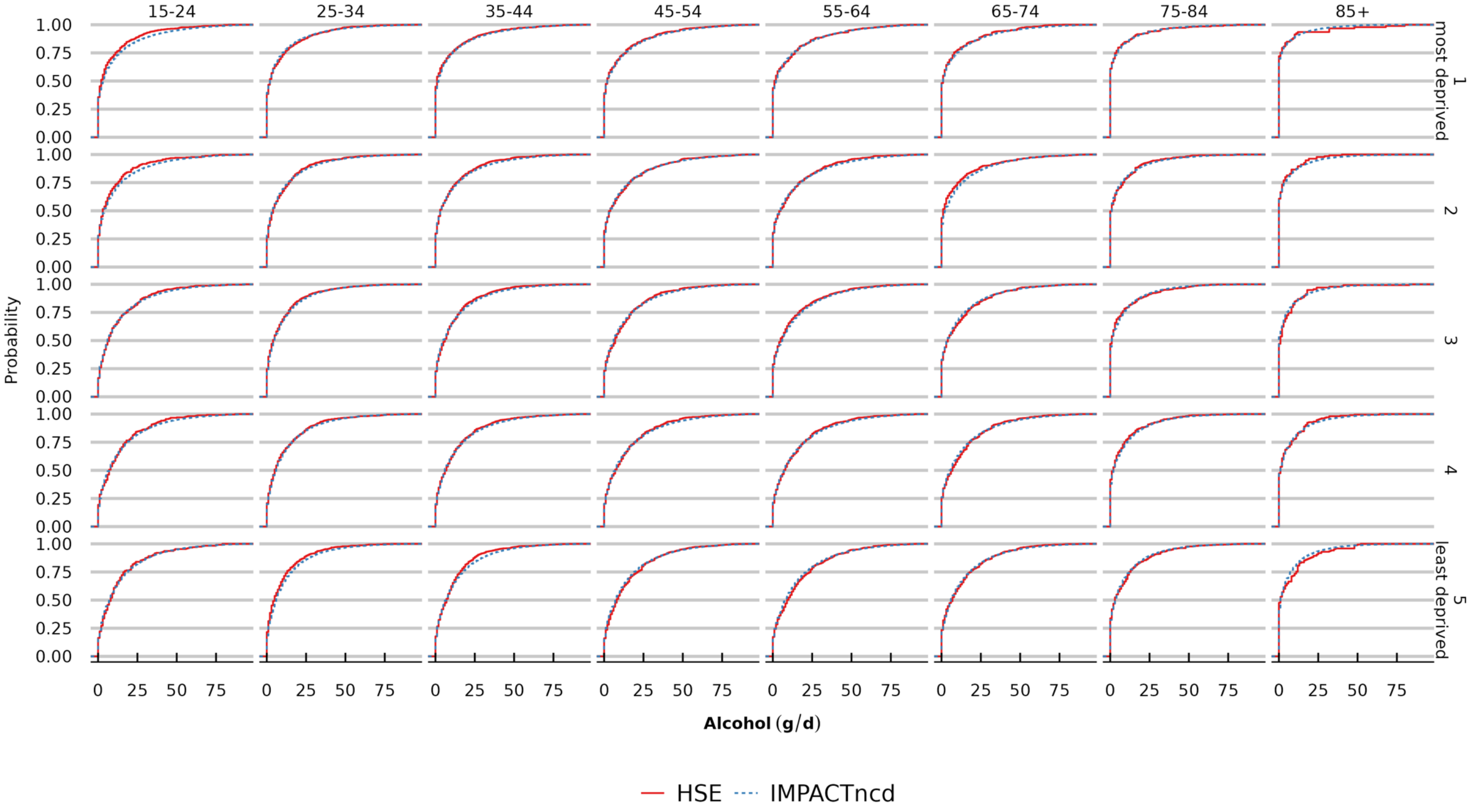

Body mass index

Figure B-7 - Validation: BMI – HSE and projected IMPACT$_{NCD}$ trends by quintile of IMD (unstandardised)

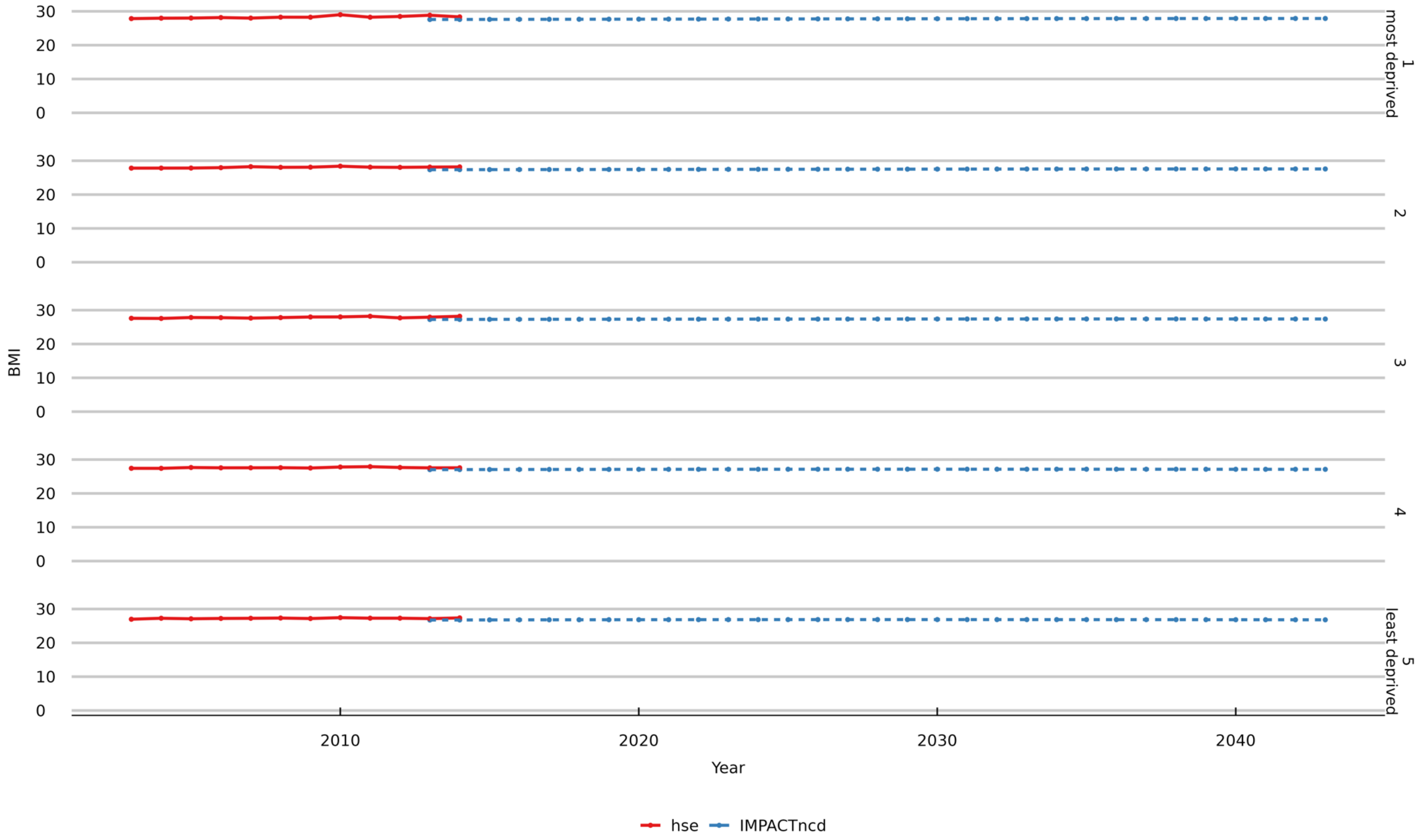

Figure B-8 Validation: Body mass index by year and age

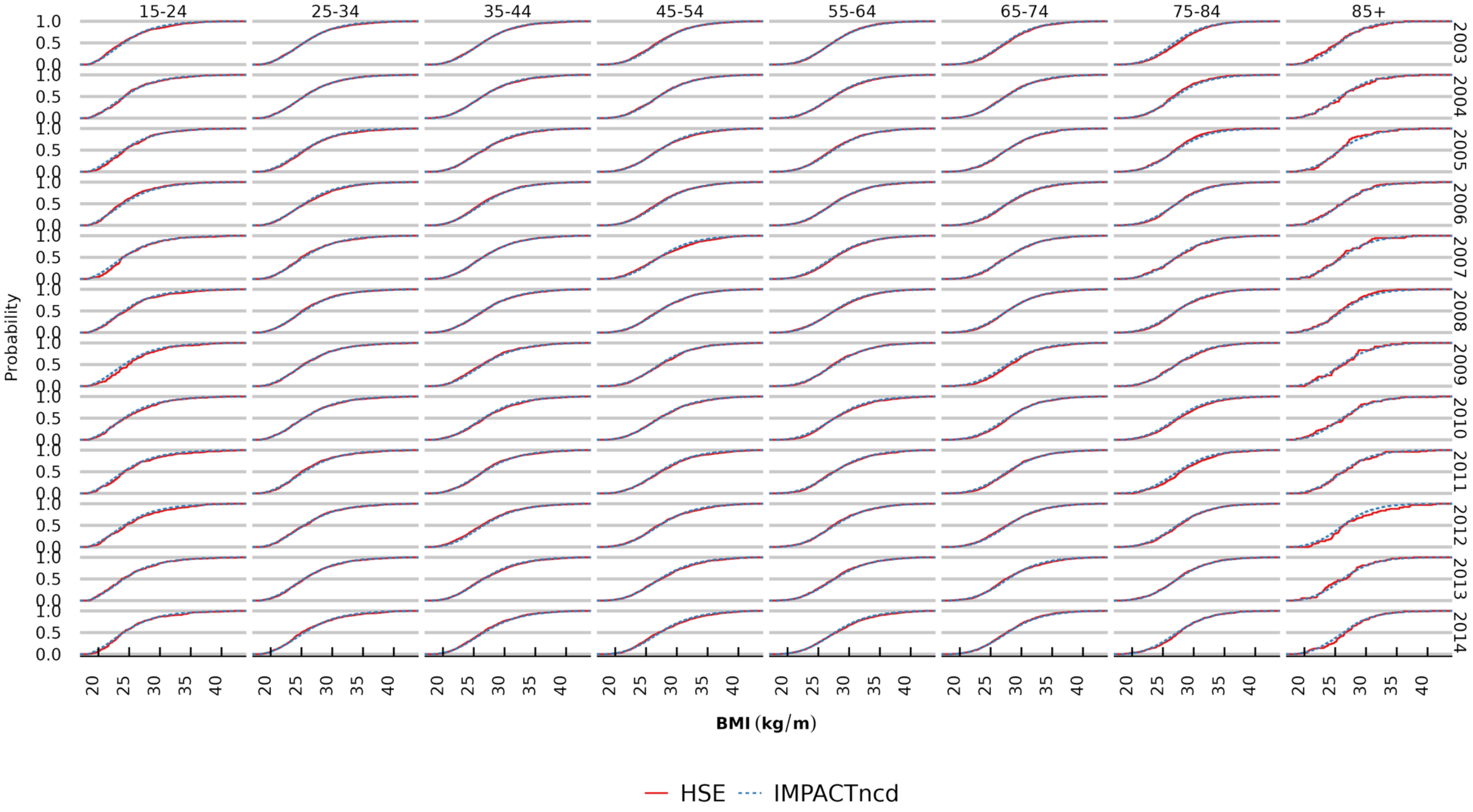

Figure B-9 - Validation: Body mass index by quintiles of IMD and age

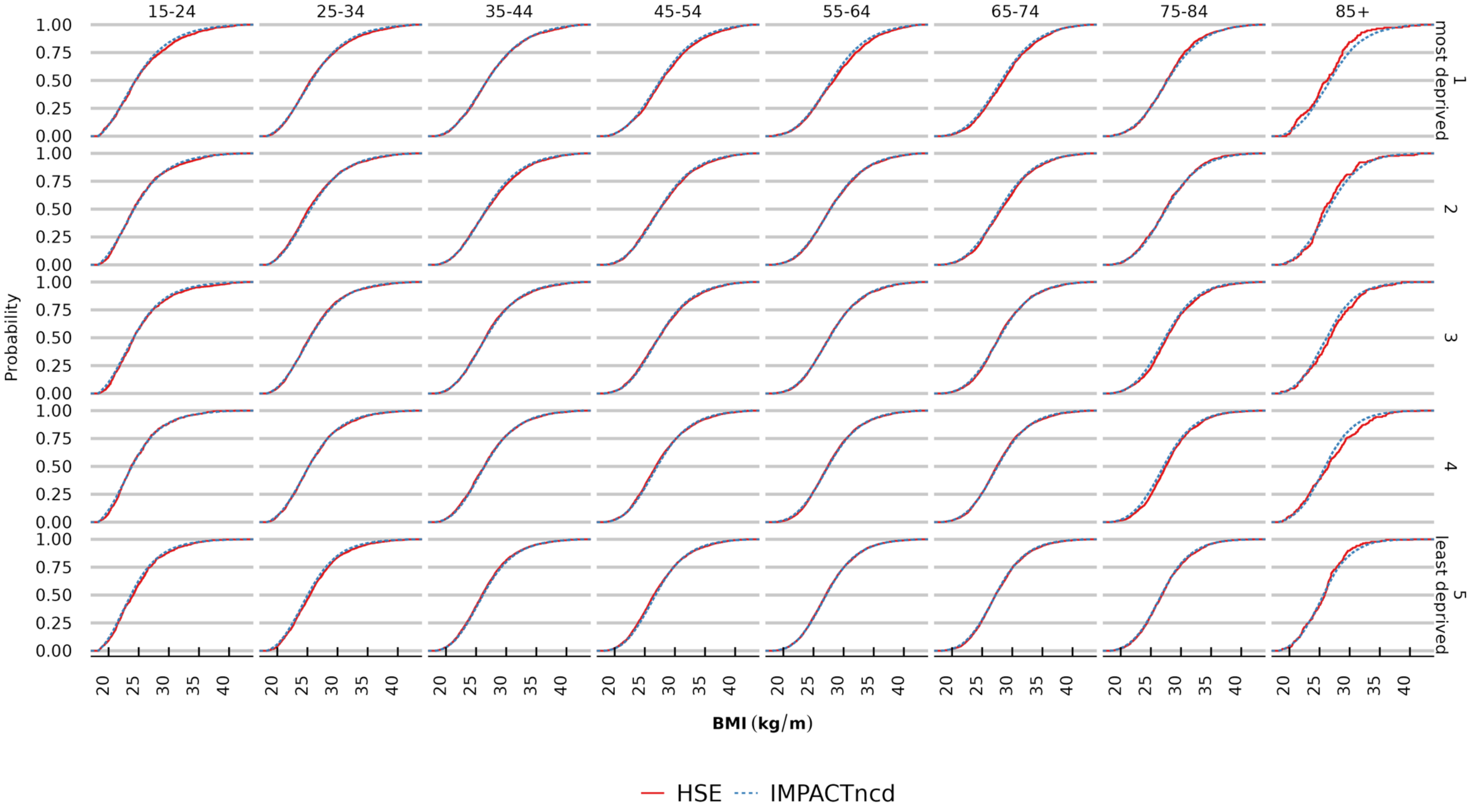

Systolic blood pressure

Figure B-10 - Validation: Systolic blood pressure – HSE and projected IMPACT$_{NCD}$ trends by quintile of IMD (unstandardised)

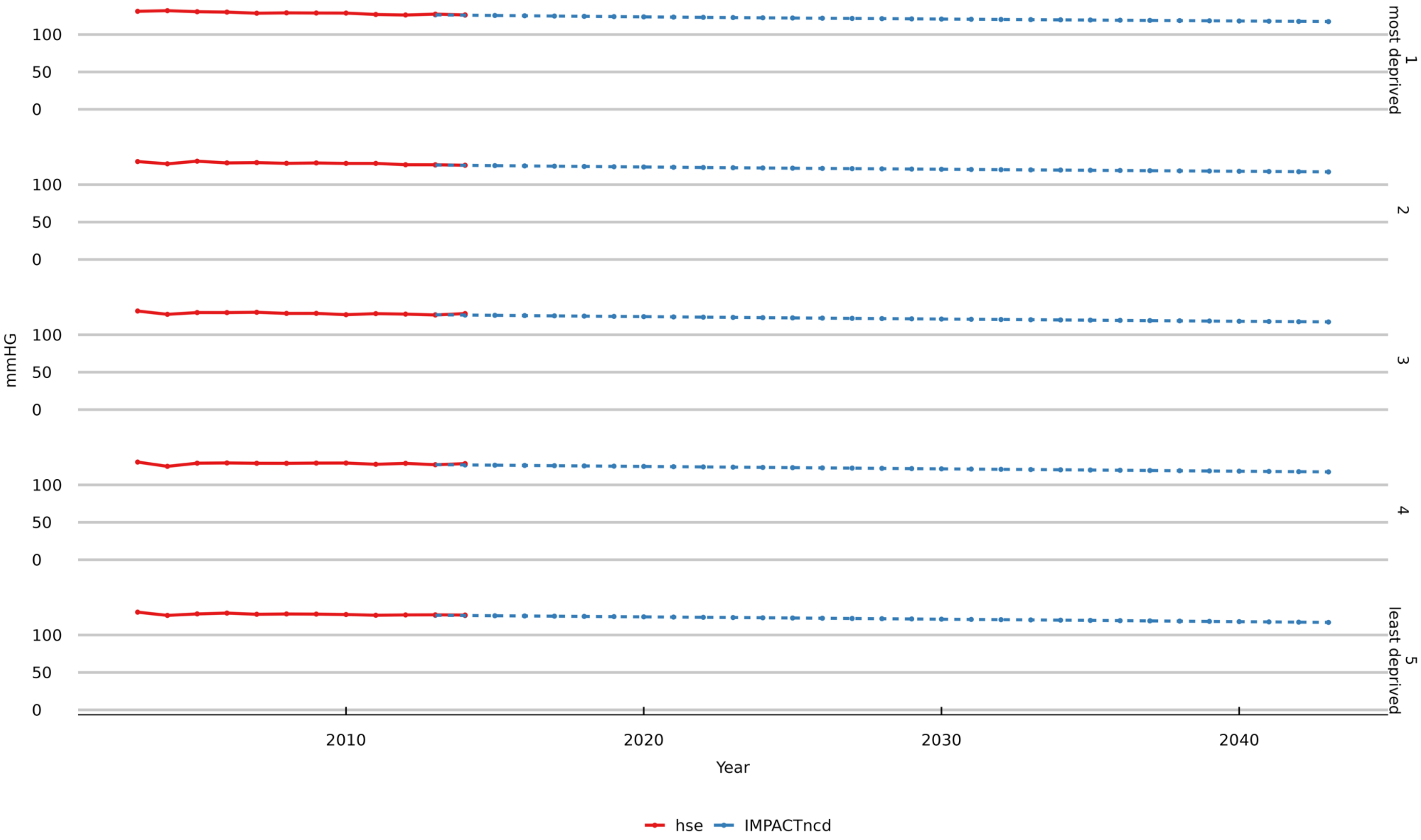

Figure B-11 - Validation: Systolic blood pressure by year and age group

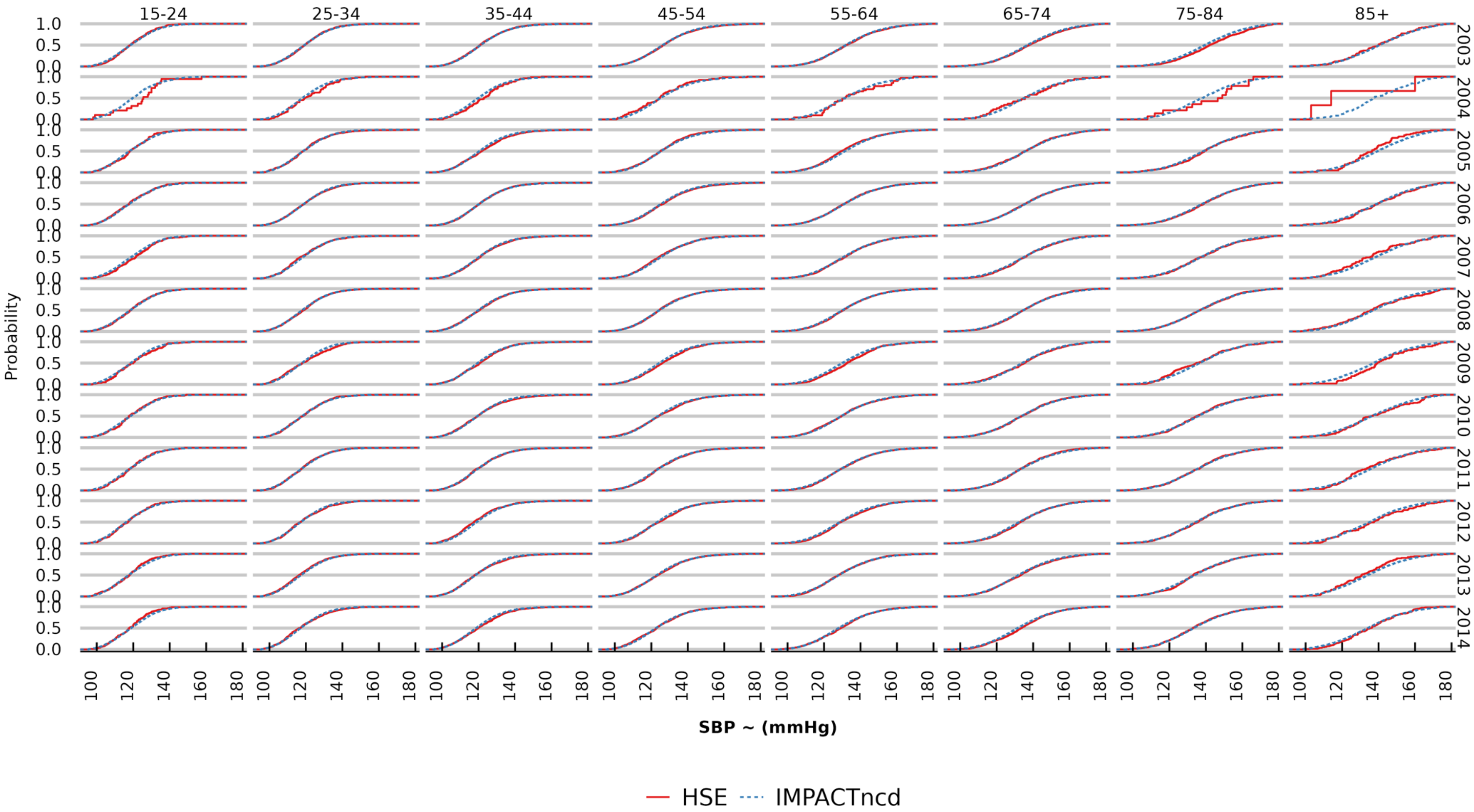

Figure B-12 - Validation: Systolic blood pressure by quintiles of IMD and age group

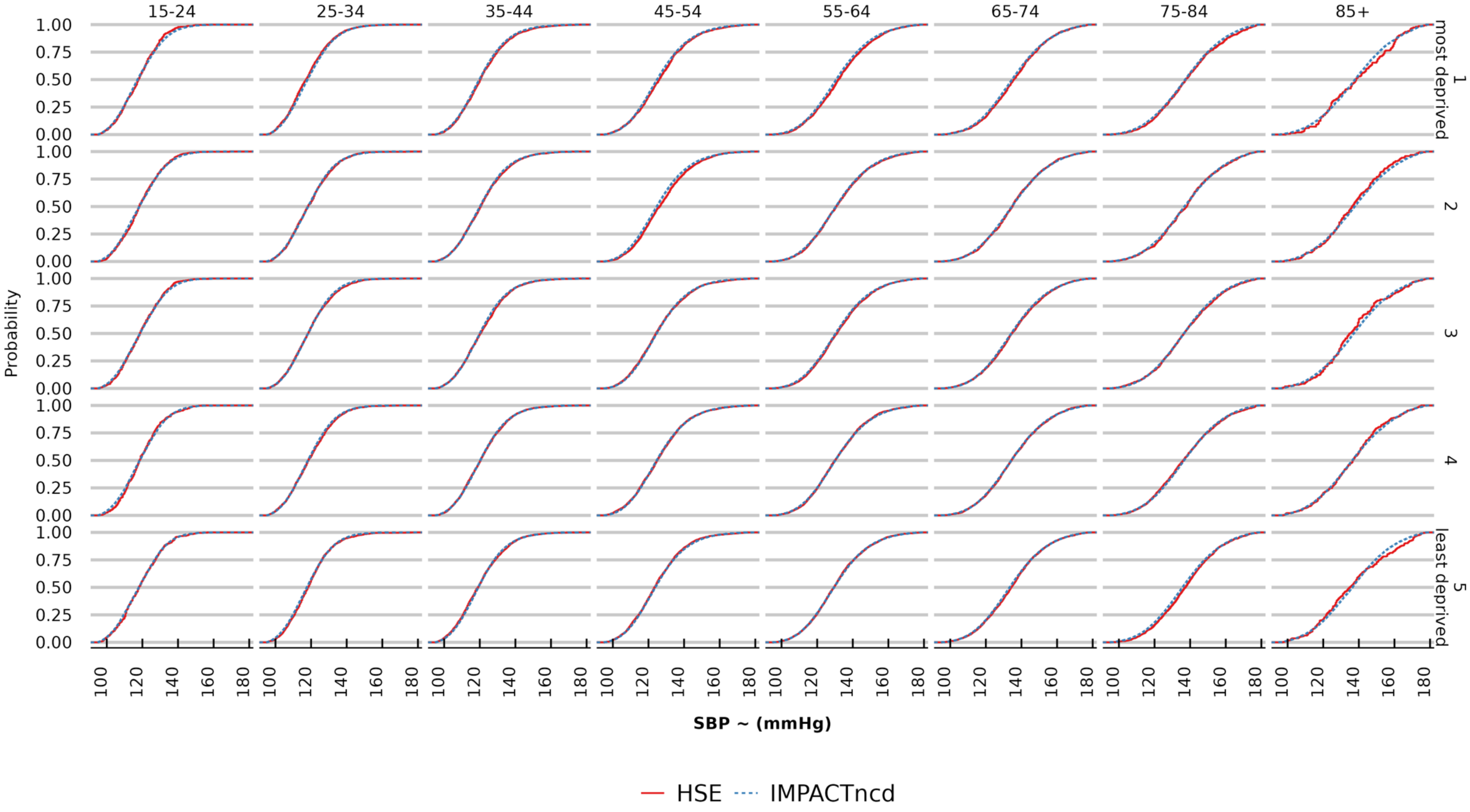

Total cholesterol

Figure B-13 - Validation: Total cholesterol – HSE and projected IMPACT$_{NCD}$ trends by quintile of IMD (unstandardised)

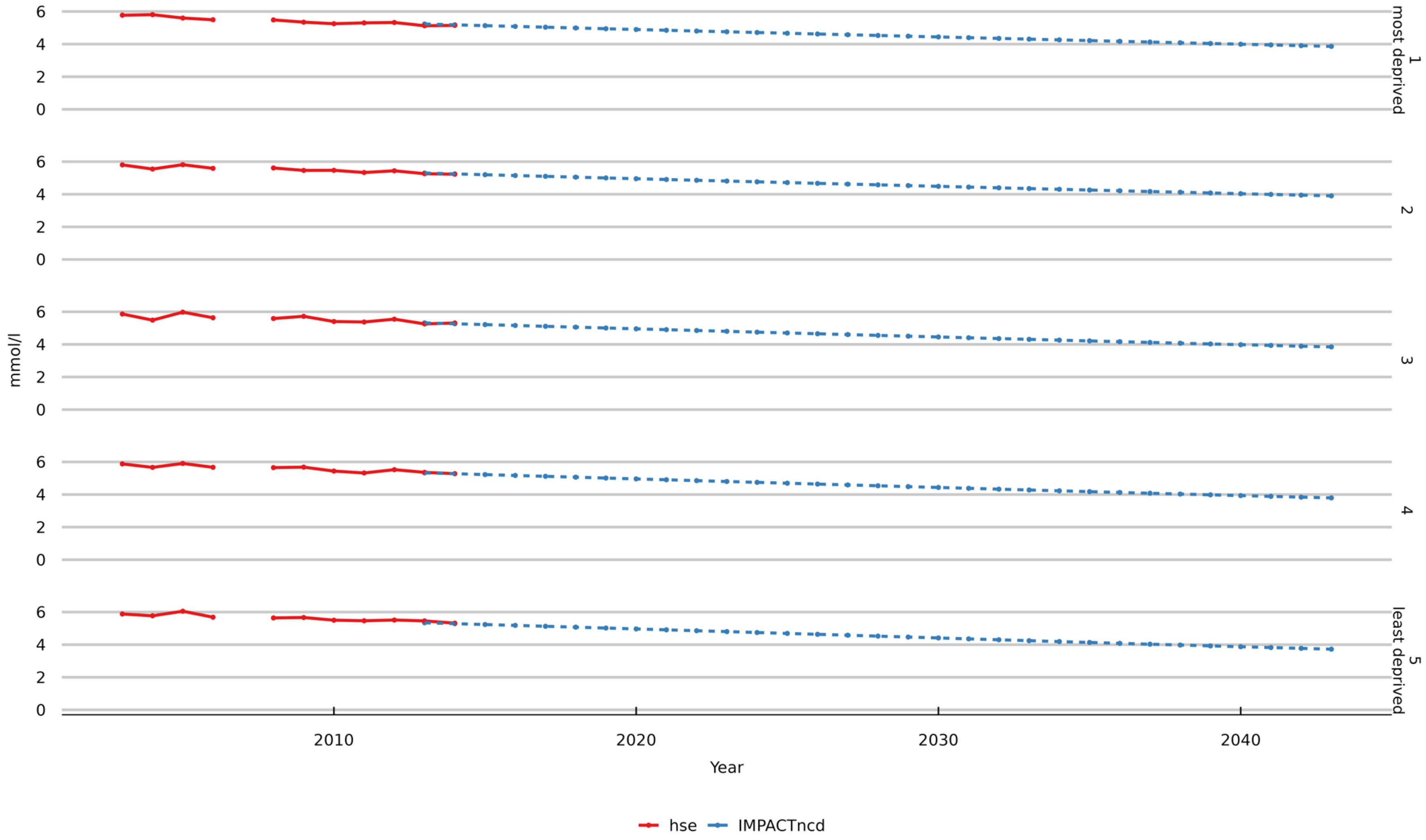

Figure B-14 - Validation: total cholesterol by year and age

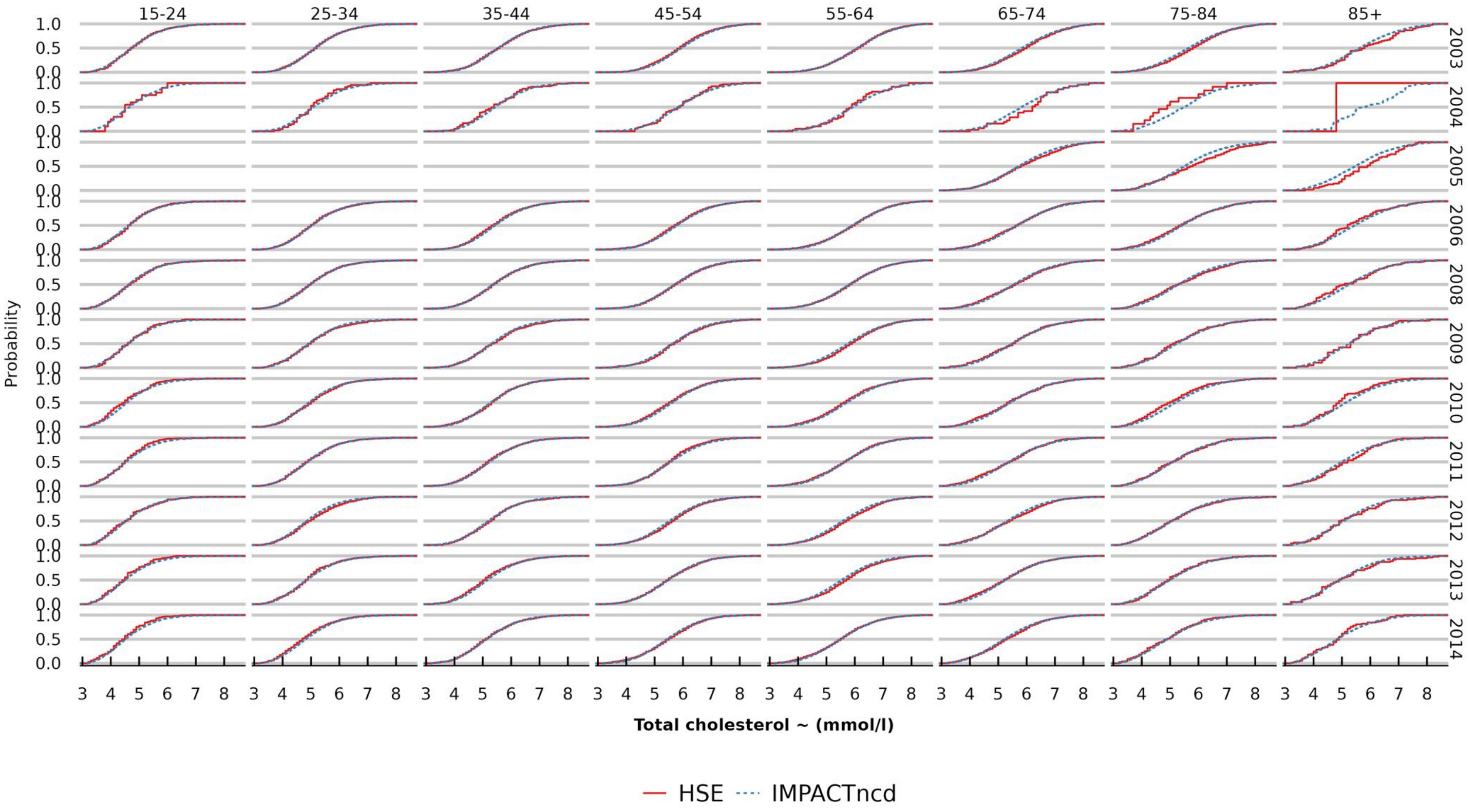

Figure B-15 - Validation: total cholesterol by quintiles of IMD and age

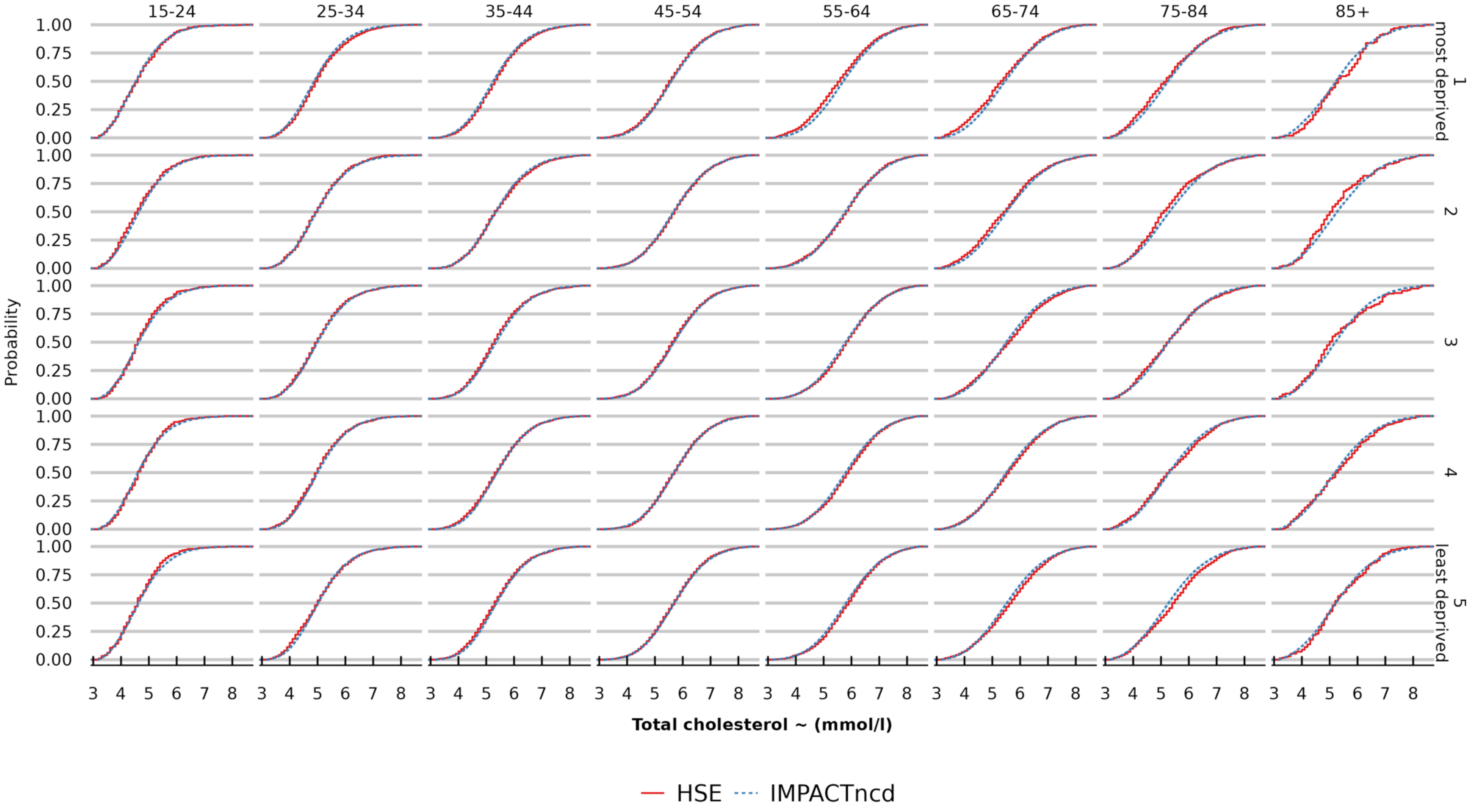

Fruit intake

Figure B-16 - Validation: fruit intake (portions per day) by year and age

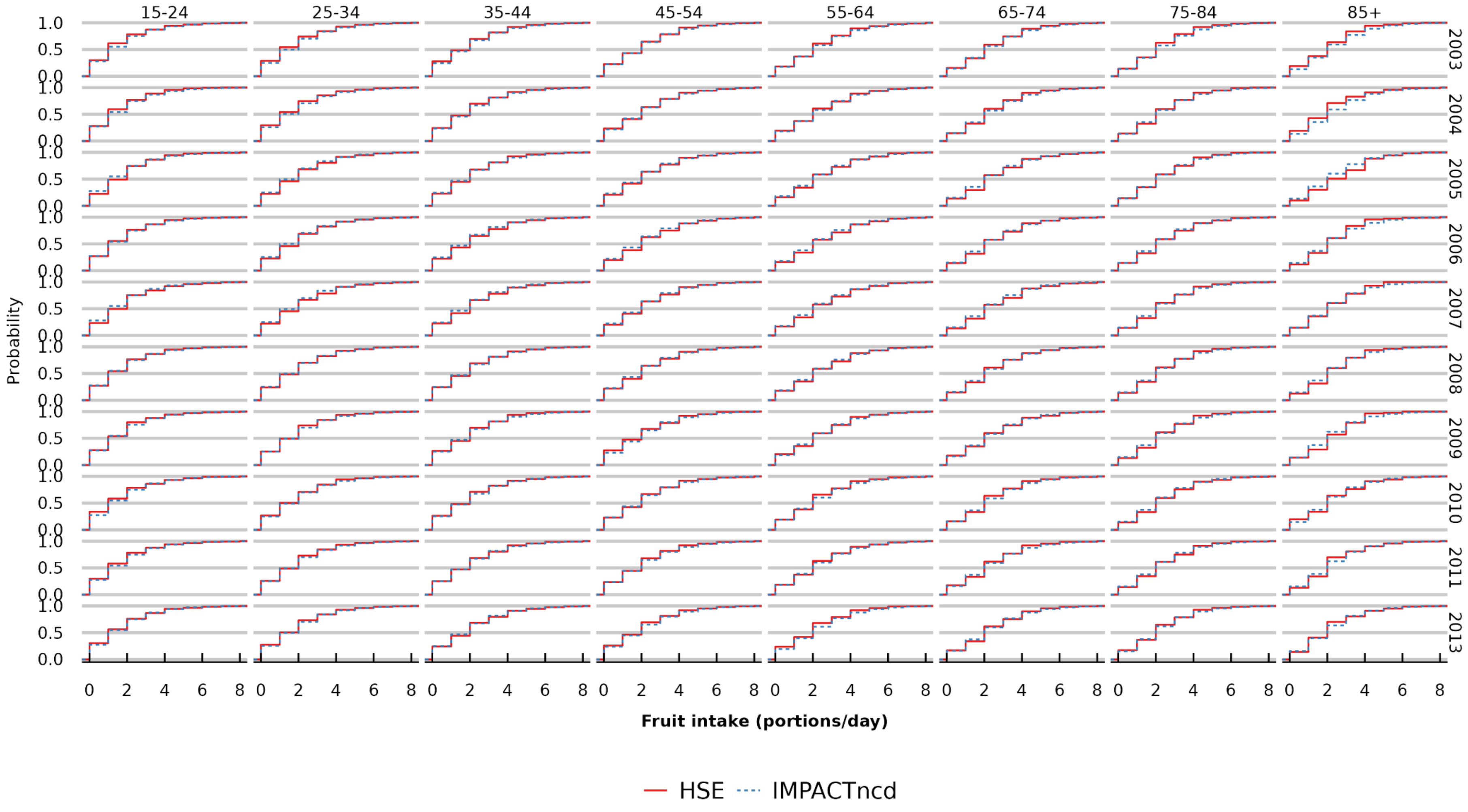

Figure B-17 - Validation: fruit intake (portions per day) by quintiles of IMD and age

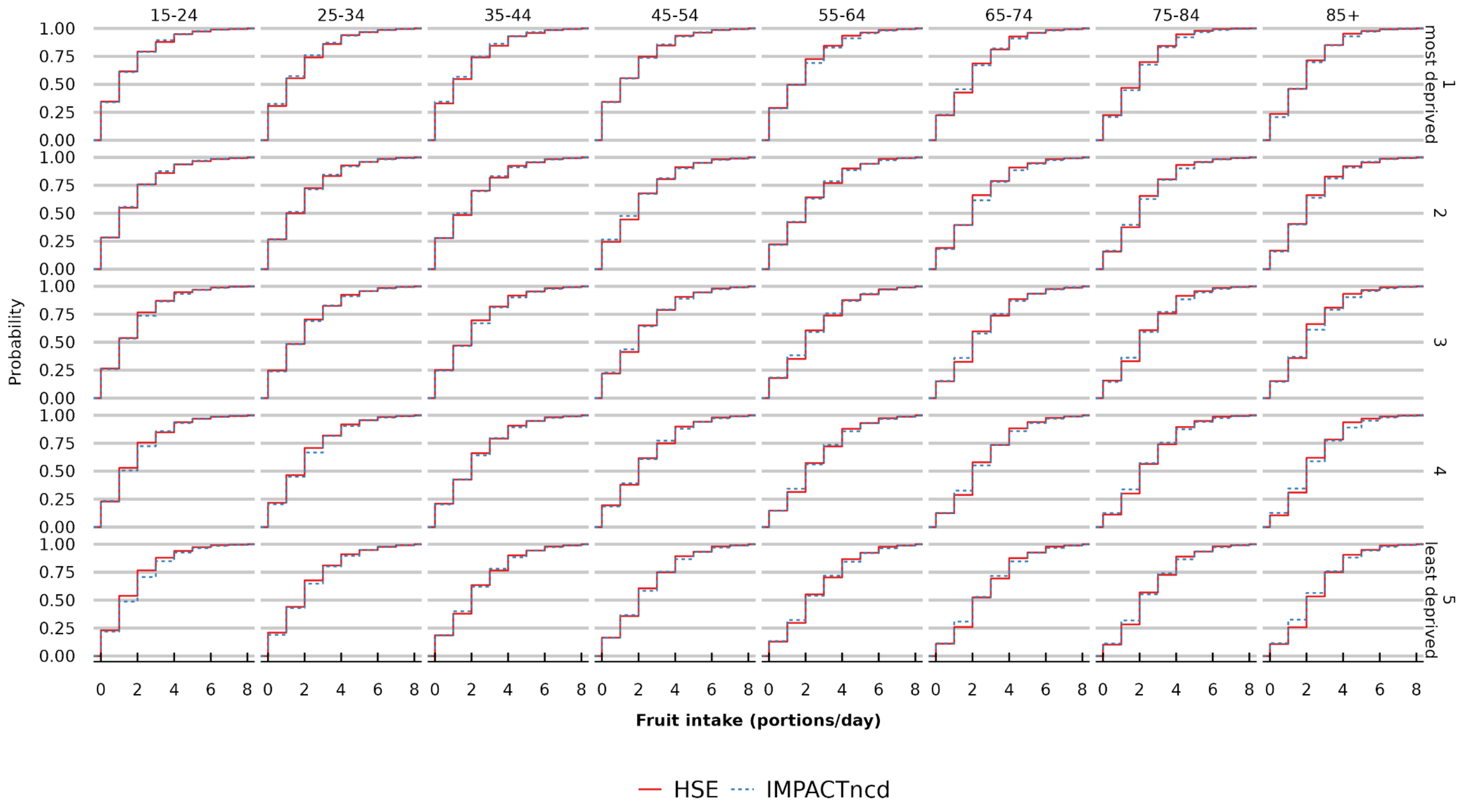

Vegetable intake

Figure B-18 - Validation: vegetable intake (portions per day) by year and age

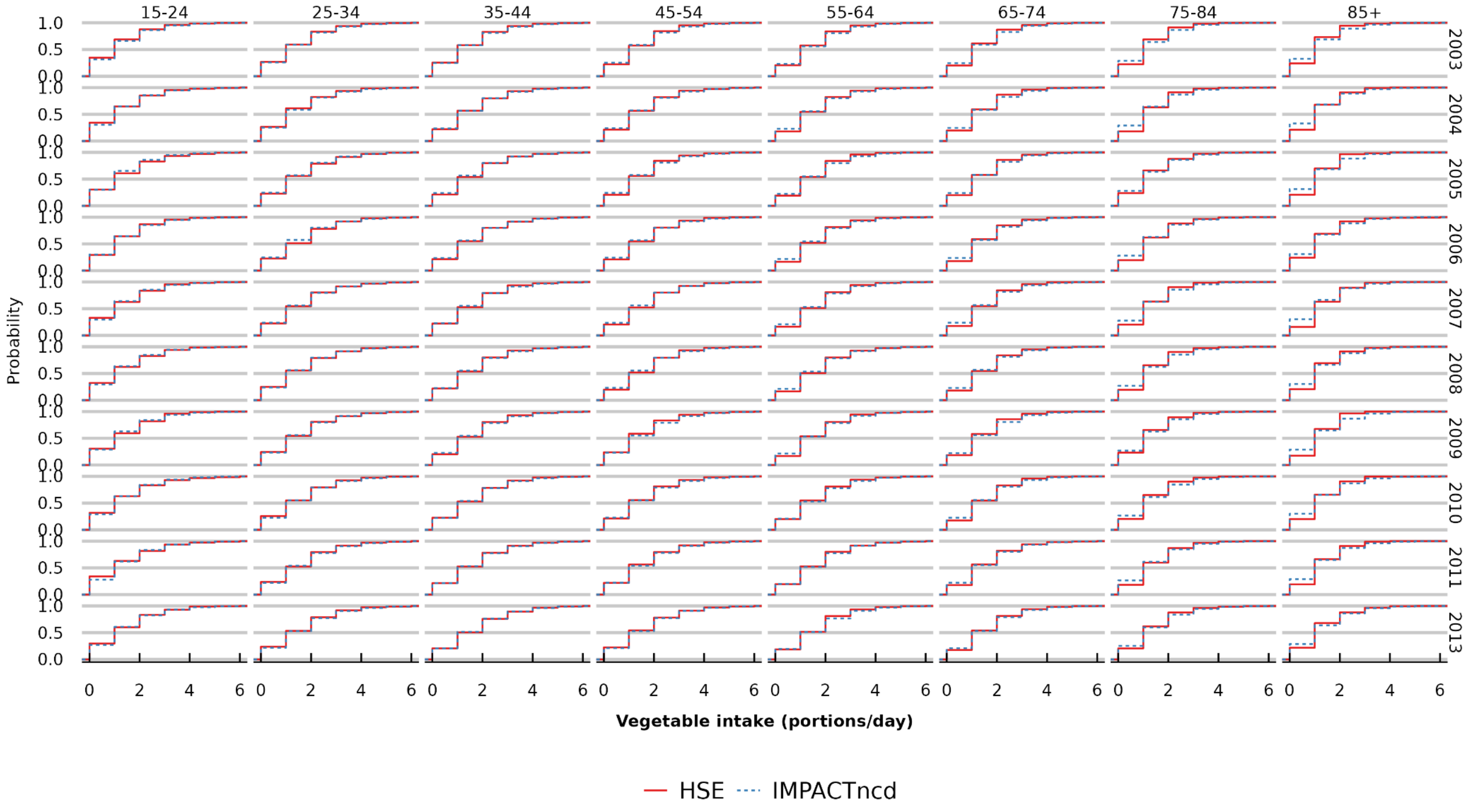

Figure B-19 - Validation: vegetable intake (portions per day) by quintiles of IMD and age

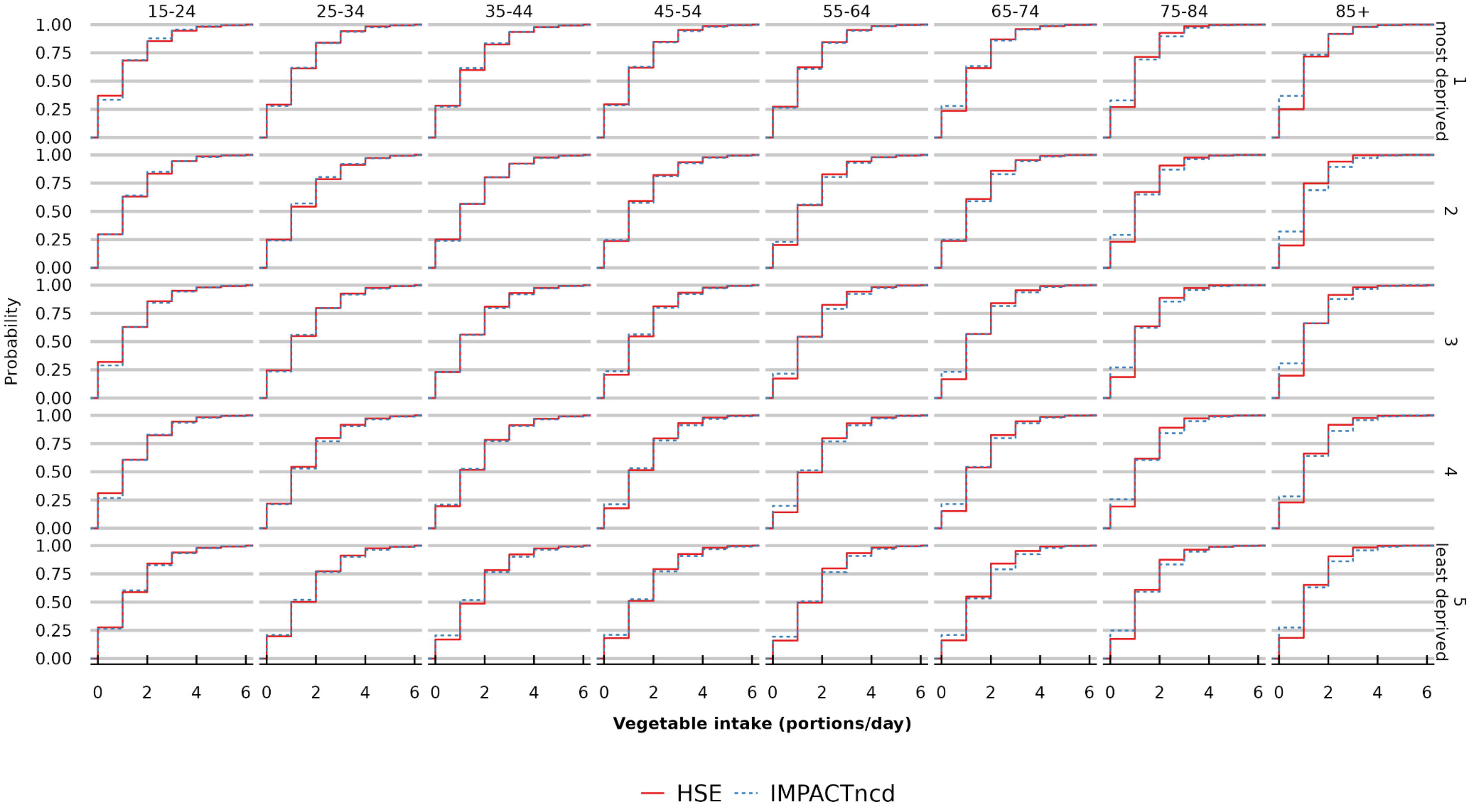

Fruit and Vegetable intake combined

Figure B-20 - Validation: Fruit and vegetable intake – HSE and projected IMPACT$_{NCD}$ trends by quintile of IMD (unstandardised)

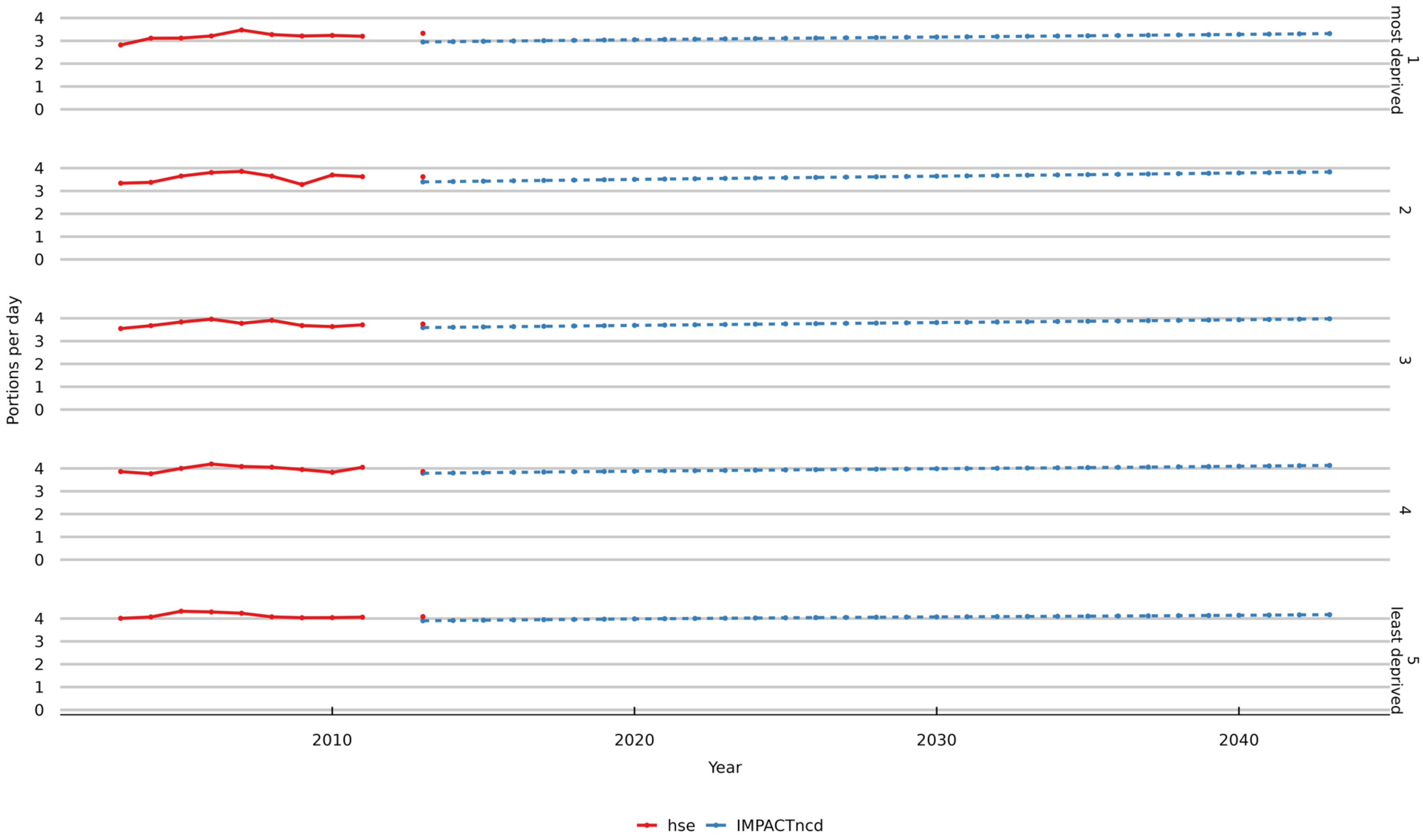

Statin prescriptions

Figure B-21 - Validation: statins prescriptions by year and age

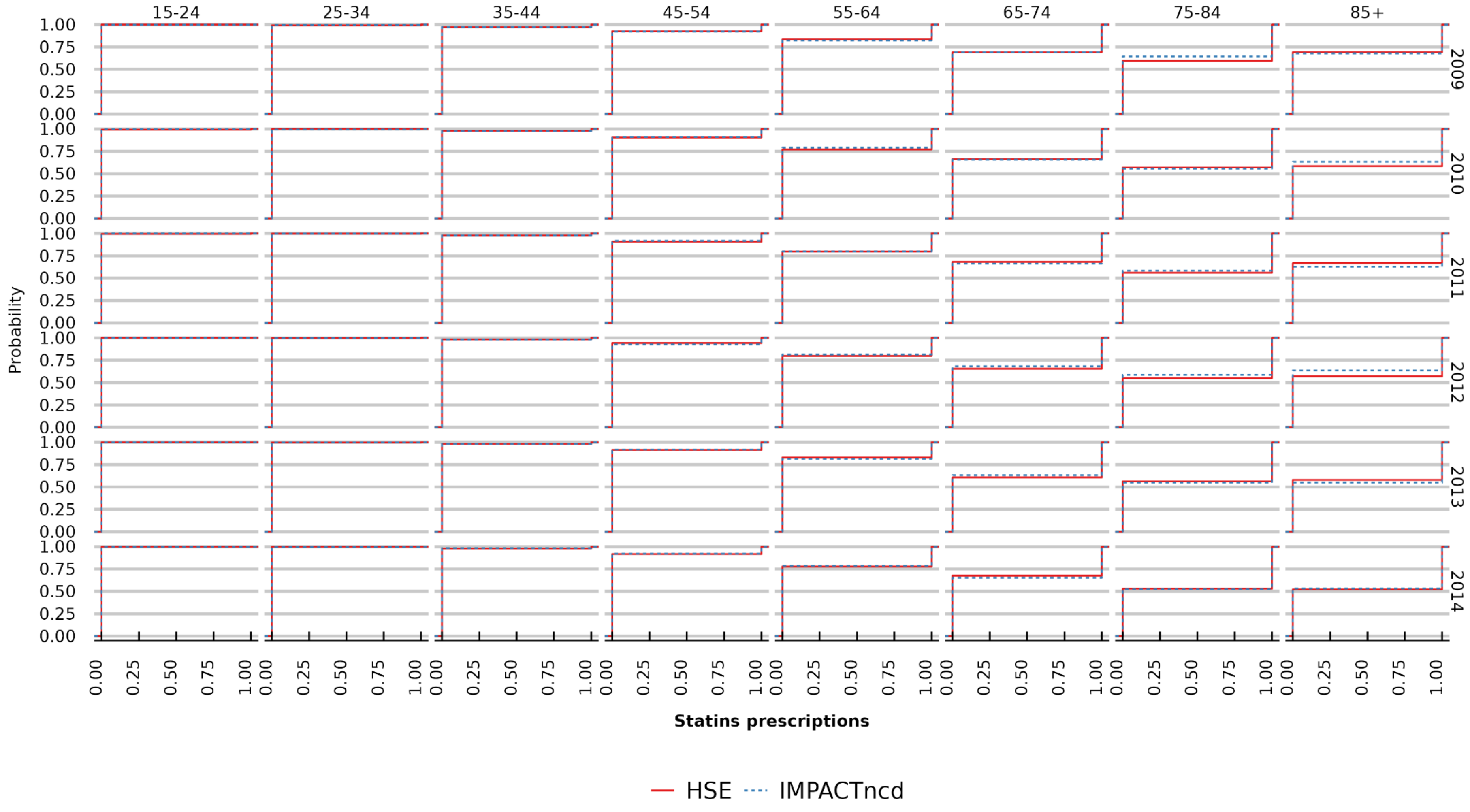

Figure B-22 - Validation: statins prescriptions by quintiles of IMD and age

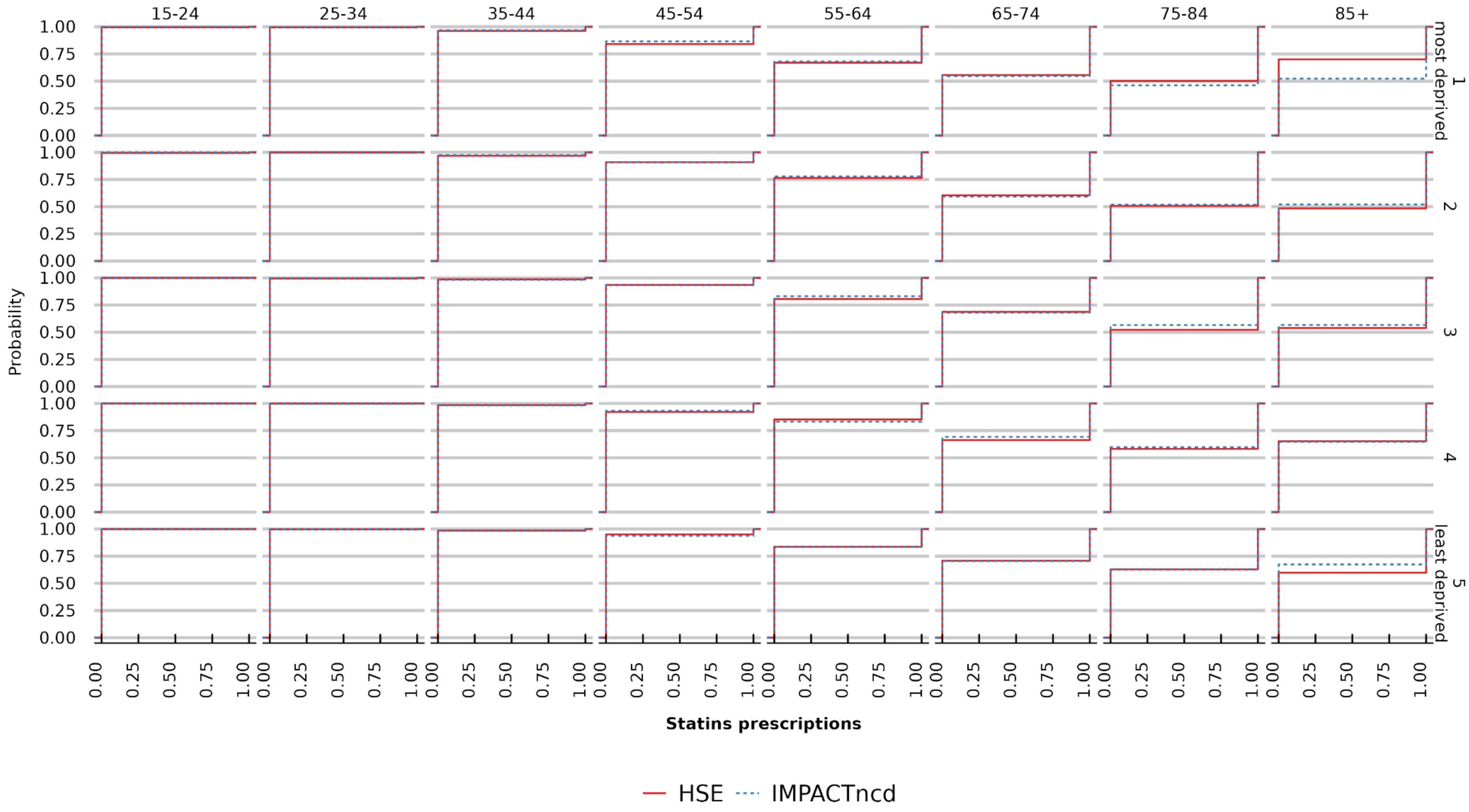

Number of cigarettes smoked (ex-smokers)

Figure B-23 - Validation: Number of cigarettes smoked by year and age group in ex-smokers

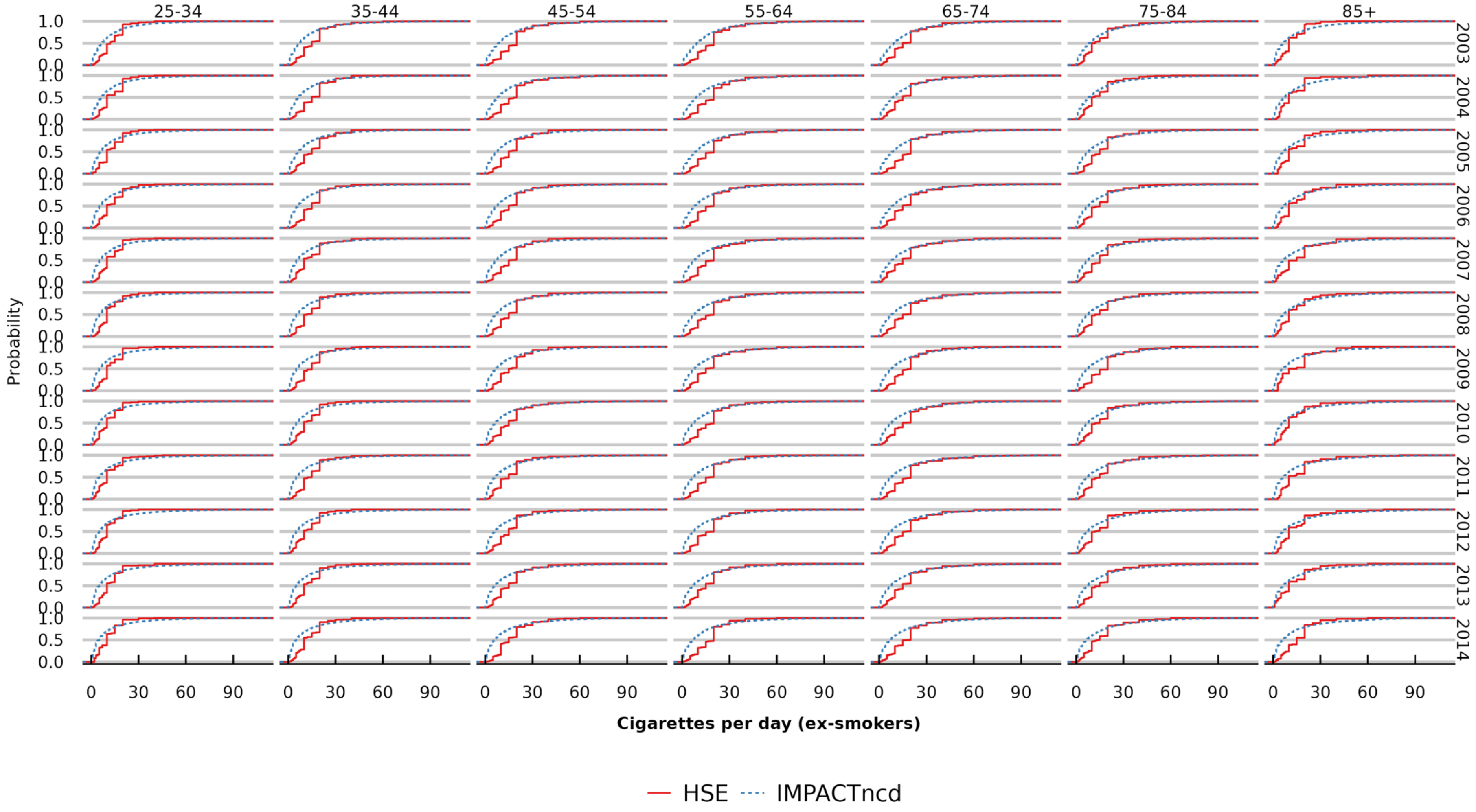

Figure B-24 - Validation: Number of cigarettes smoked by quintiles of IMD and age group in ex-smokers

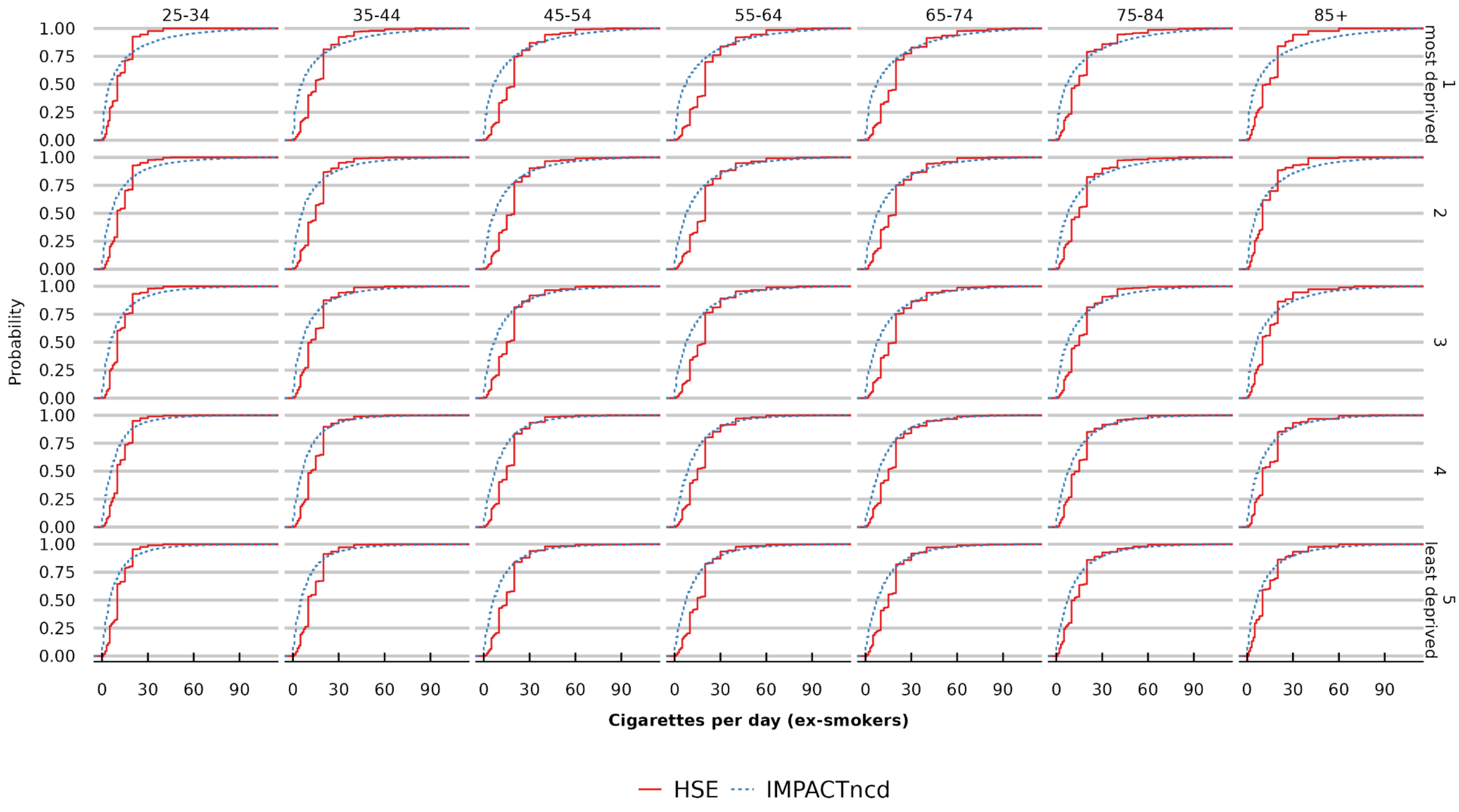

Number of cigarettes smoked (current smokers)

Figure B-25 - Validation: Number of cigarettes smoked by year and age group in current smokers

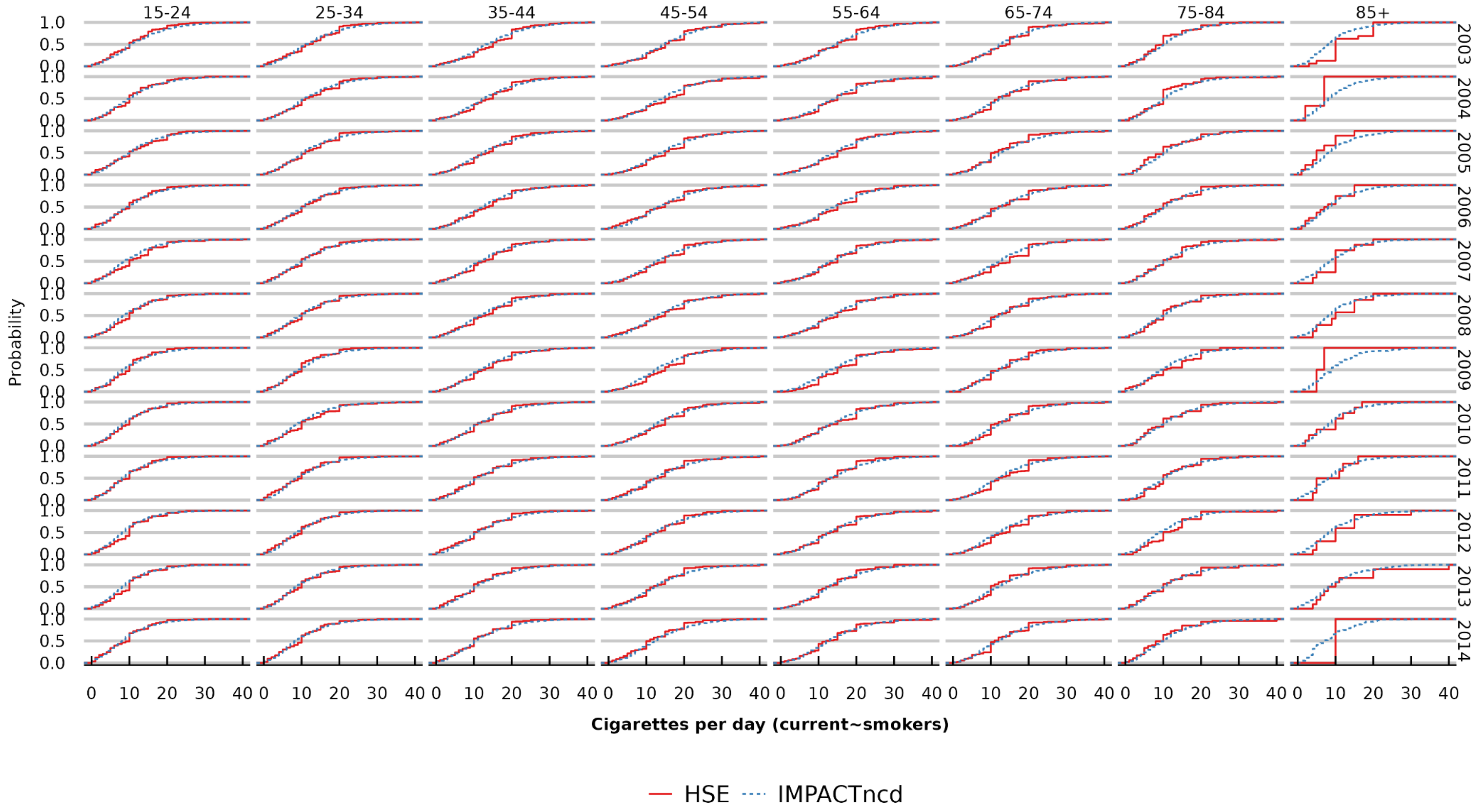

Figure B-26 - Validation: Number of cigarettes smoked by quintiles of IMD and age group in current smokers

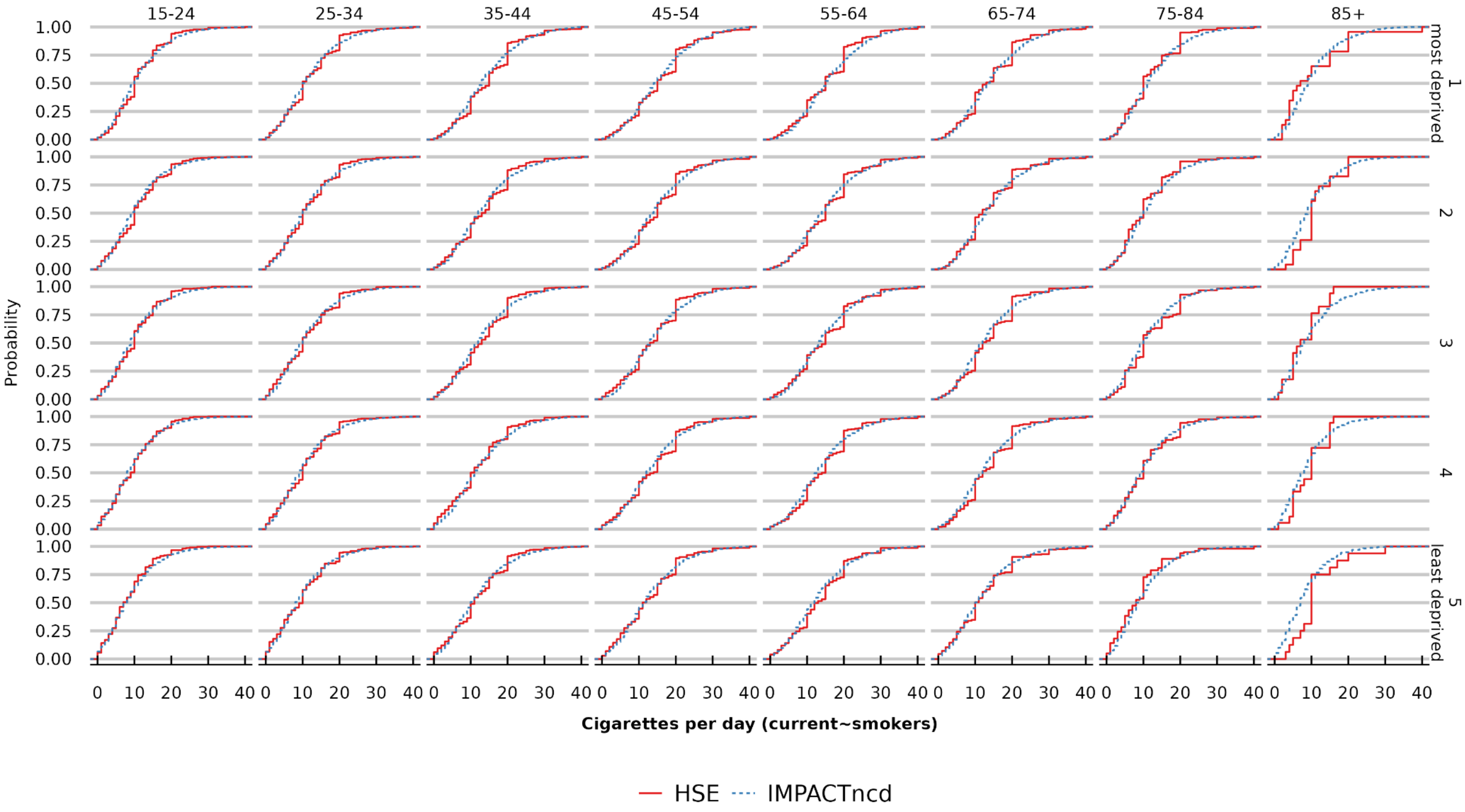

Smoking status

Figure B-27 - Validation: Proportion of active smokers – HSE and projected IMPACT$_{NCD}$ trends by quintile of IMD (unstandardised)

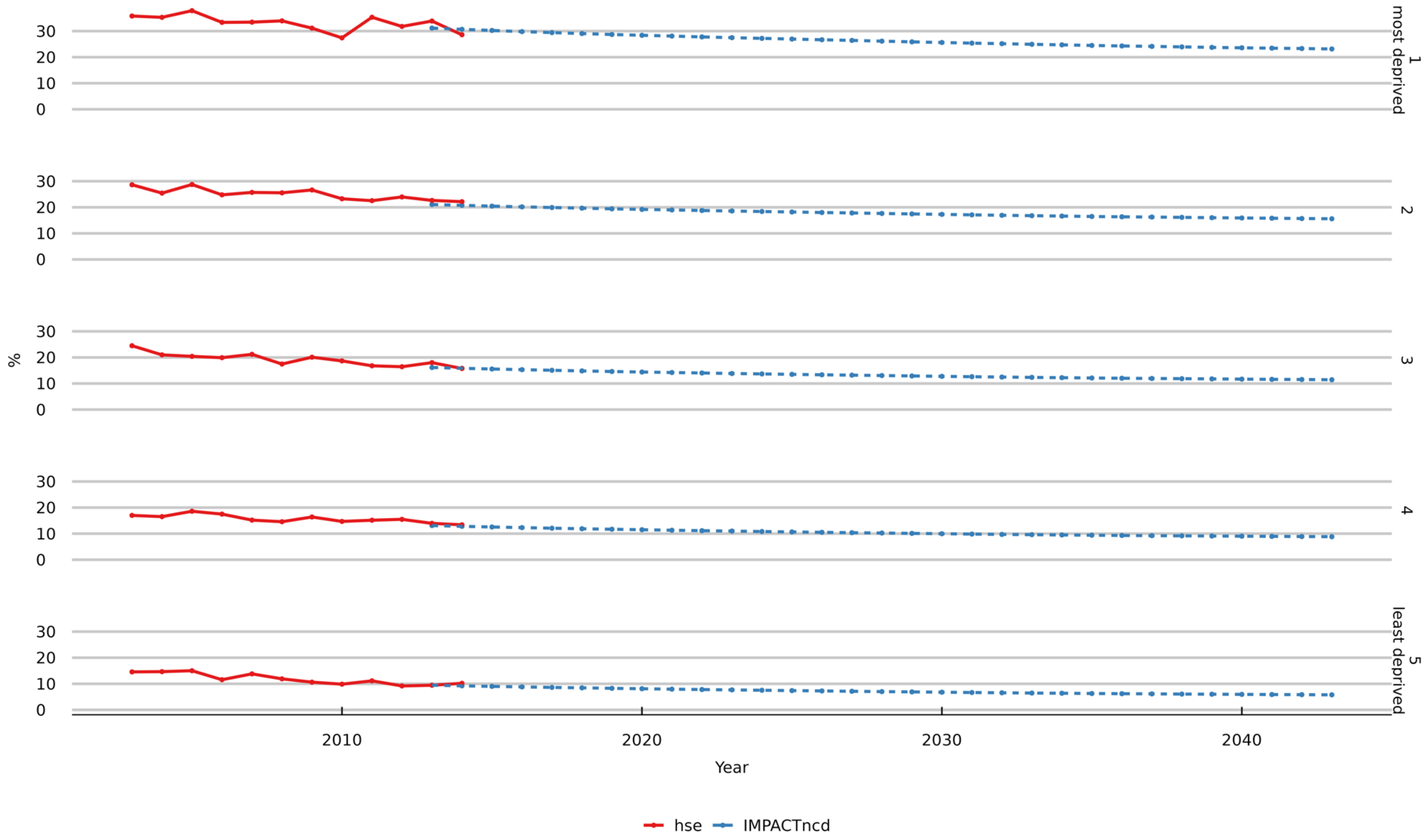

Figure B-28 - Validation: Smoking status by year and age group

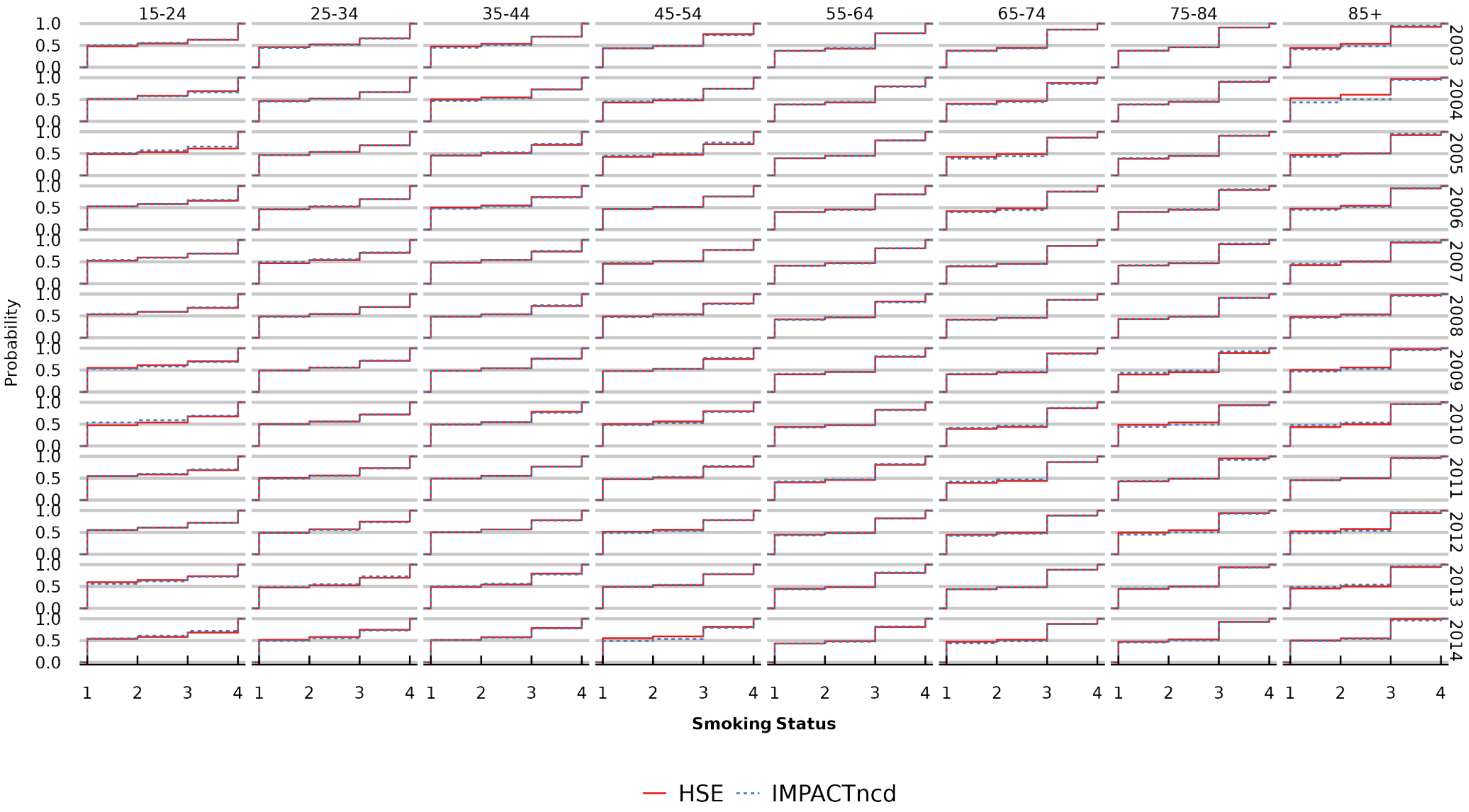

Figure B-29 - Validation: Smoking status by quintiles of IMD and age group

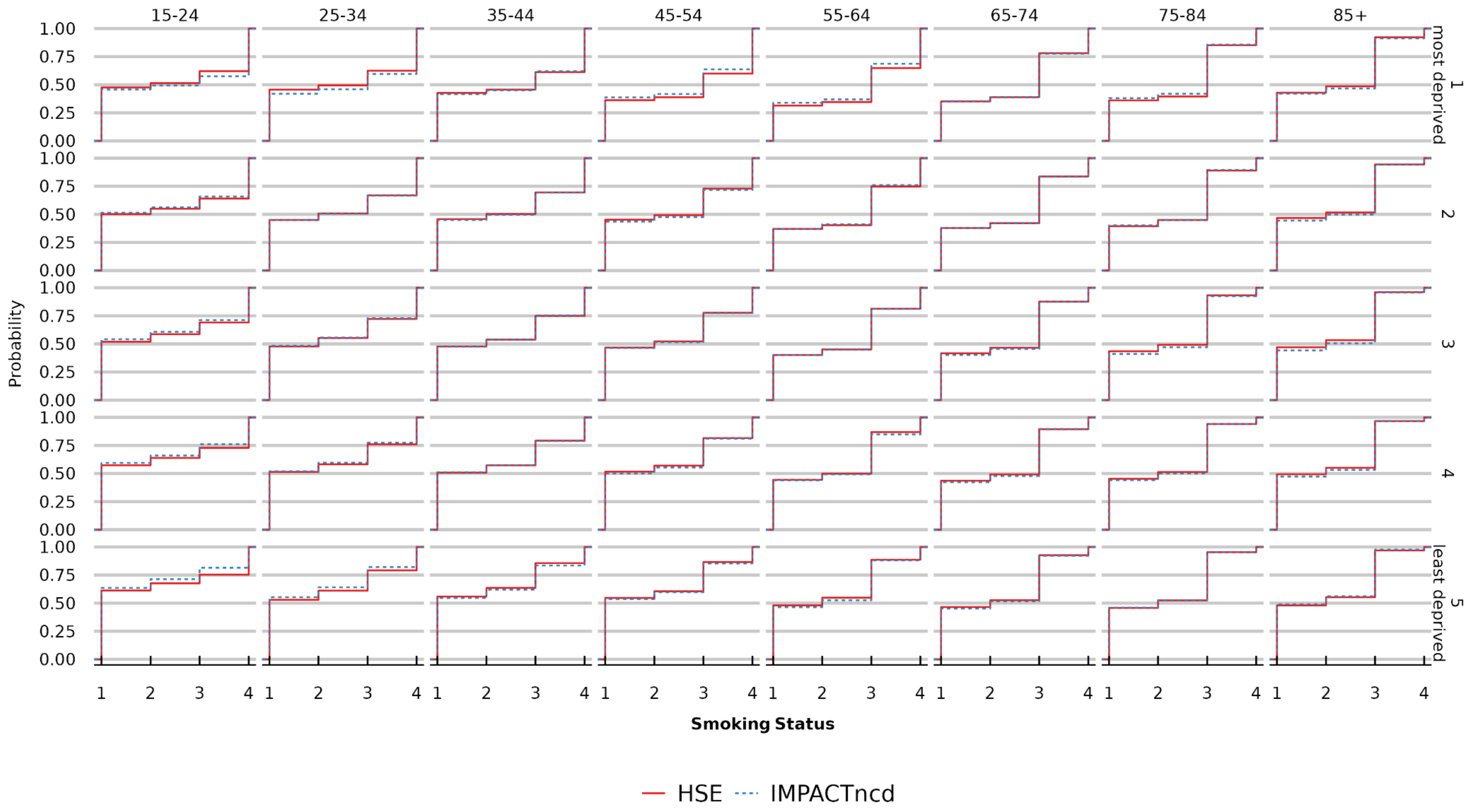

Years of smoking (ex-smokers)

Figure B-30 - Validation: years of smoking (ex-smokers) by year and age

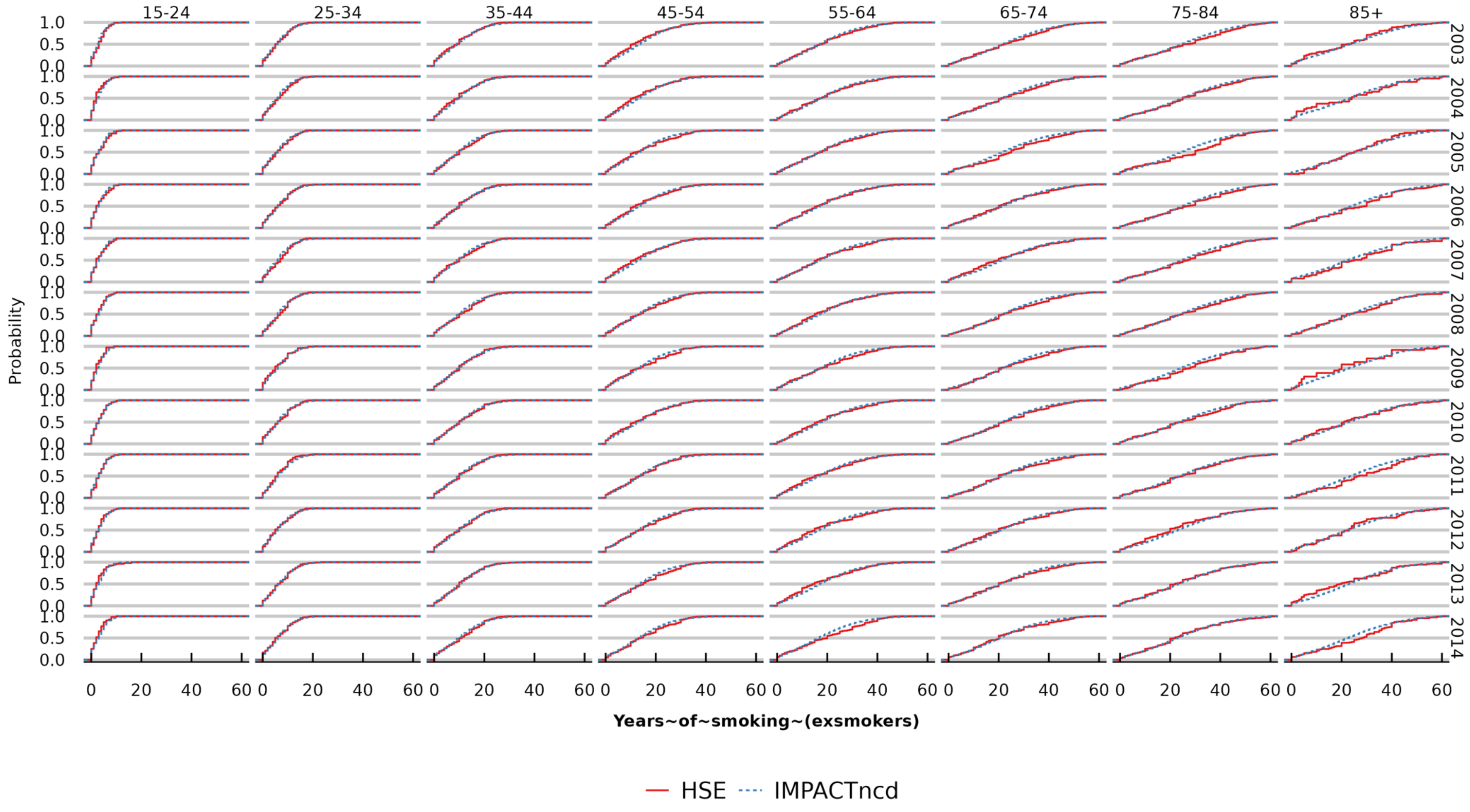

Figure B-31 - Validation: years of smoking (ex-smokers) by quintiles of IMD and age

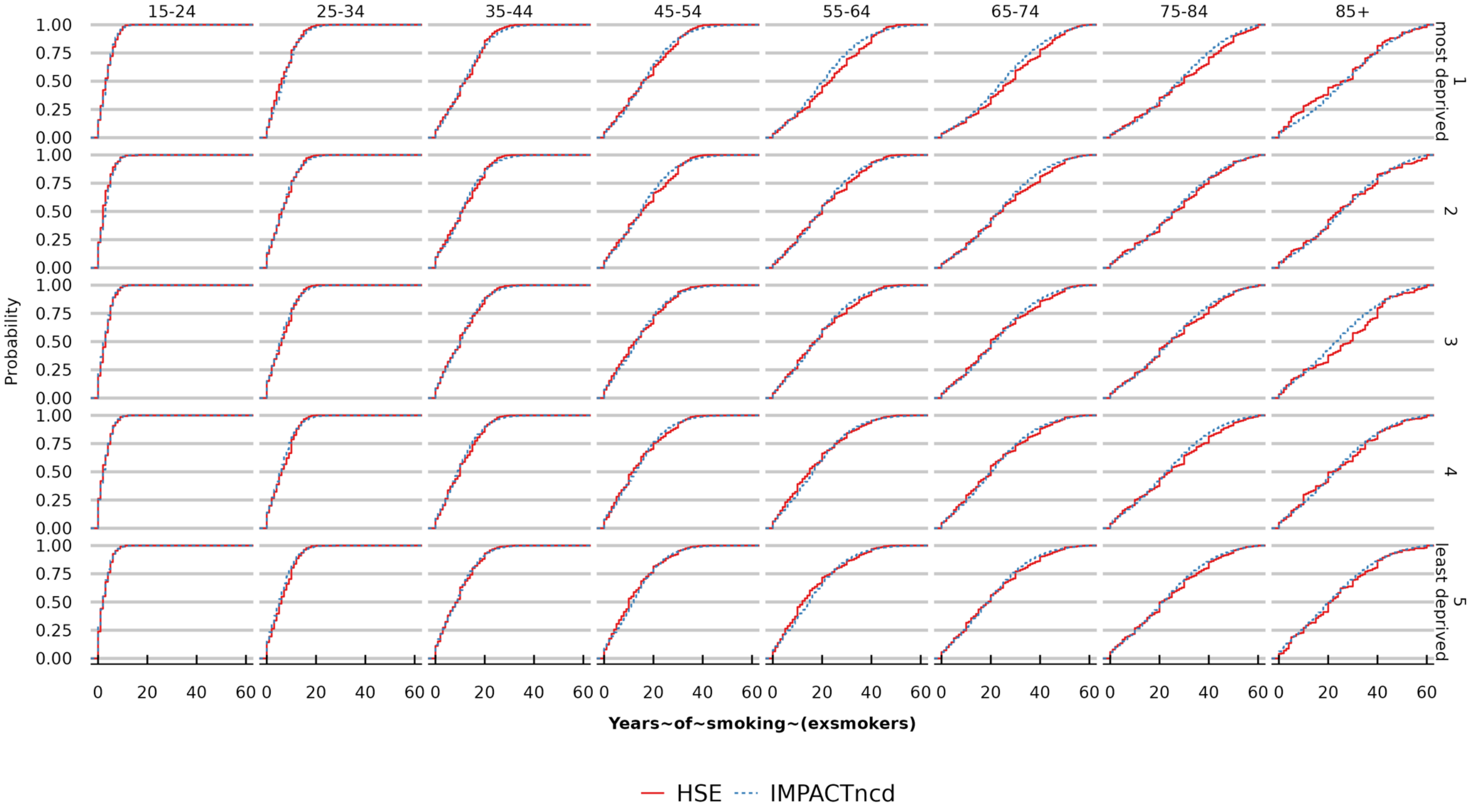

Years of smoking (current smokers)

Figure B-32 - Validation: years of smoking (current smokers) by year and age

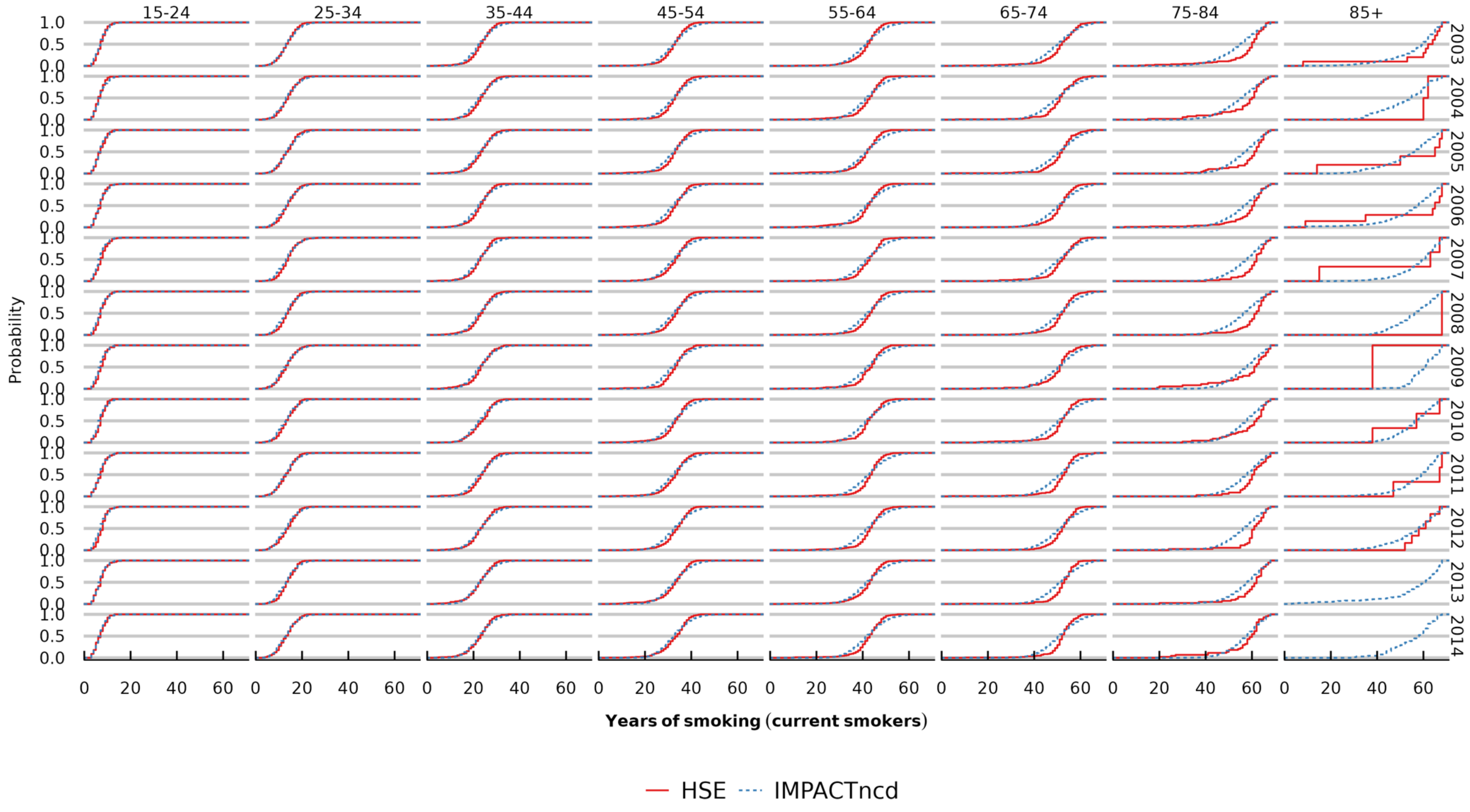

Figure B-33 - Validation: years of smoking (current smokers) by quintiles of IMD and age

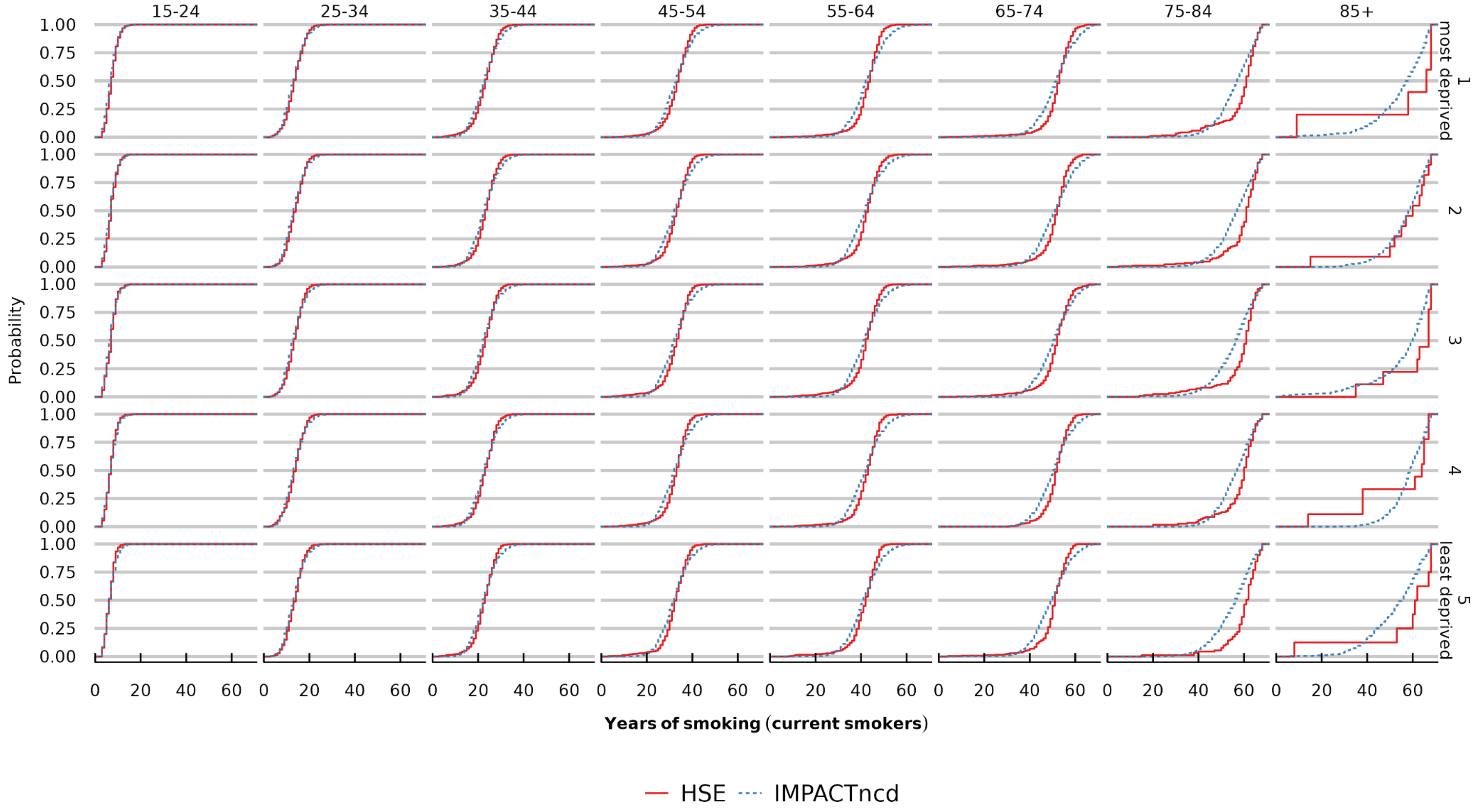

Years since smoking cessation

Figure B-34 - Validation: years since smoking cessation by year and age

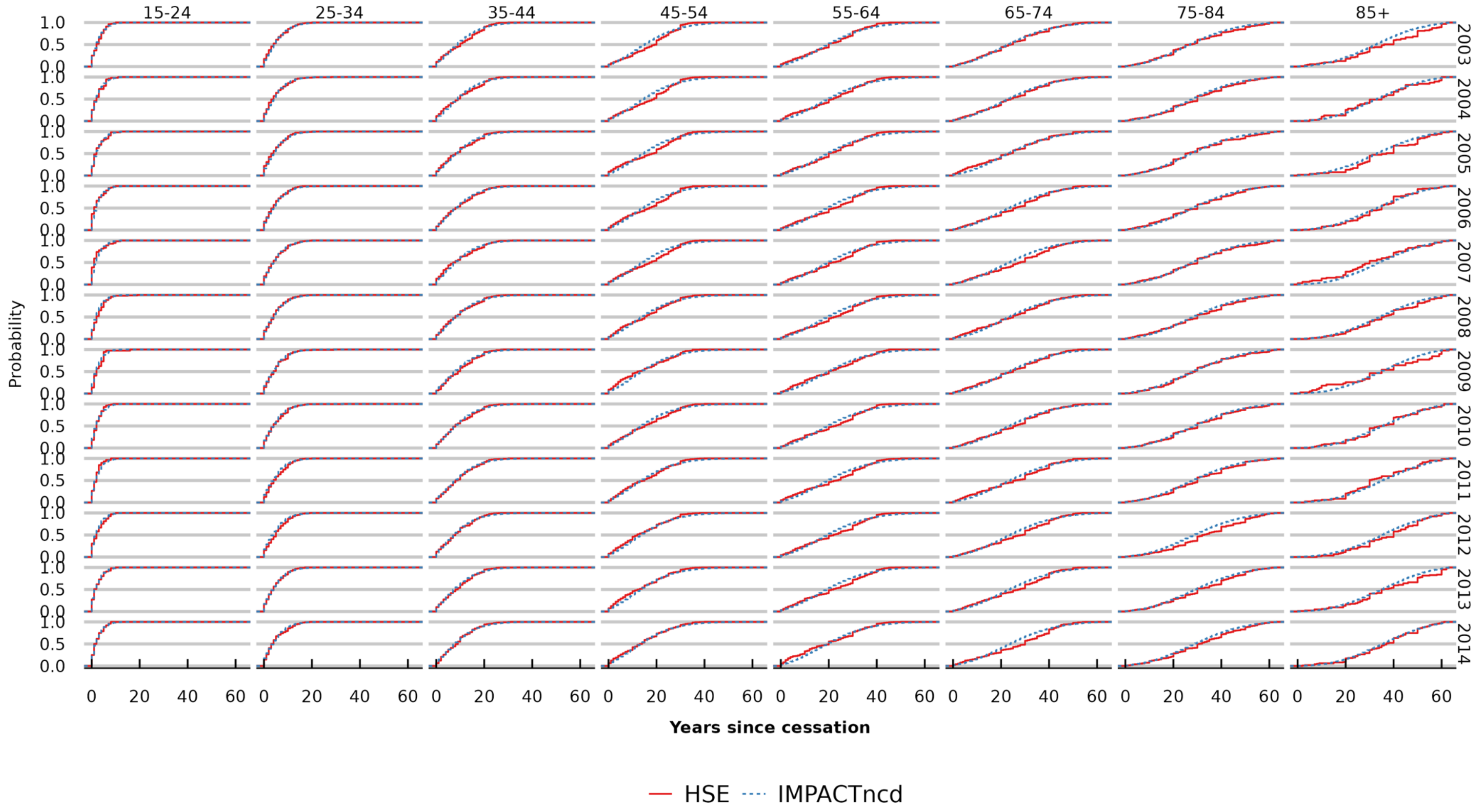

Figure B-35 - Validation: years since smoking cessation by quintiles of IMD and age

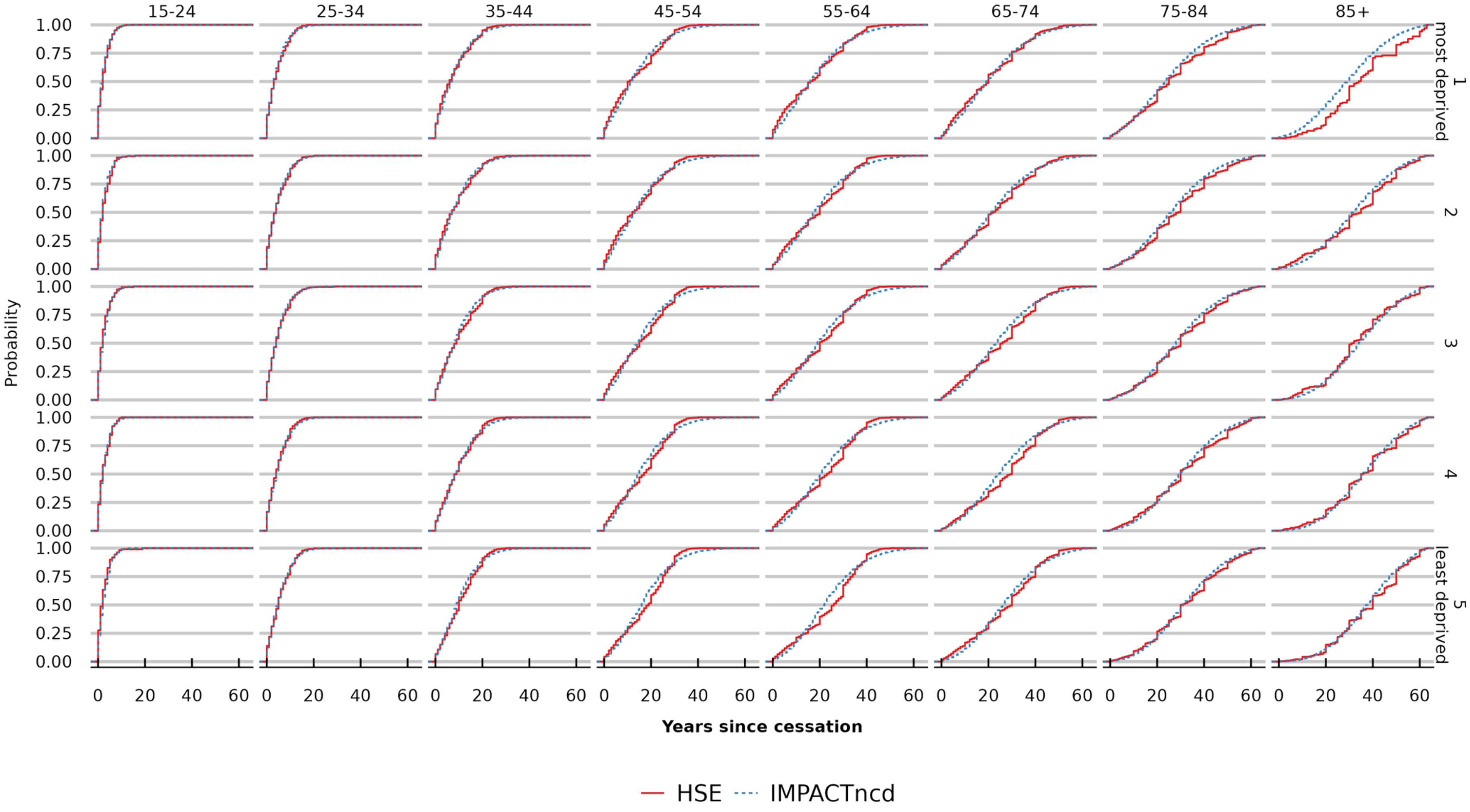

Exposure to environmental smoking

Figure B-36 - Validation: Exposure to environmental smoking – HSE and projected IMPACT$_{NCD}$ trends by quintile of IMD (unstandardised)

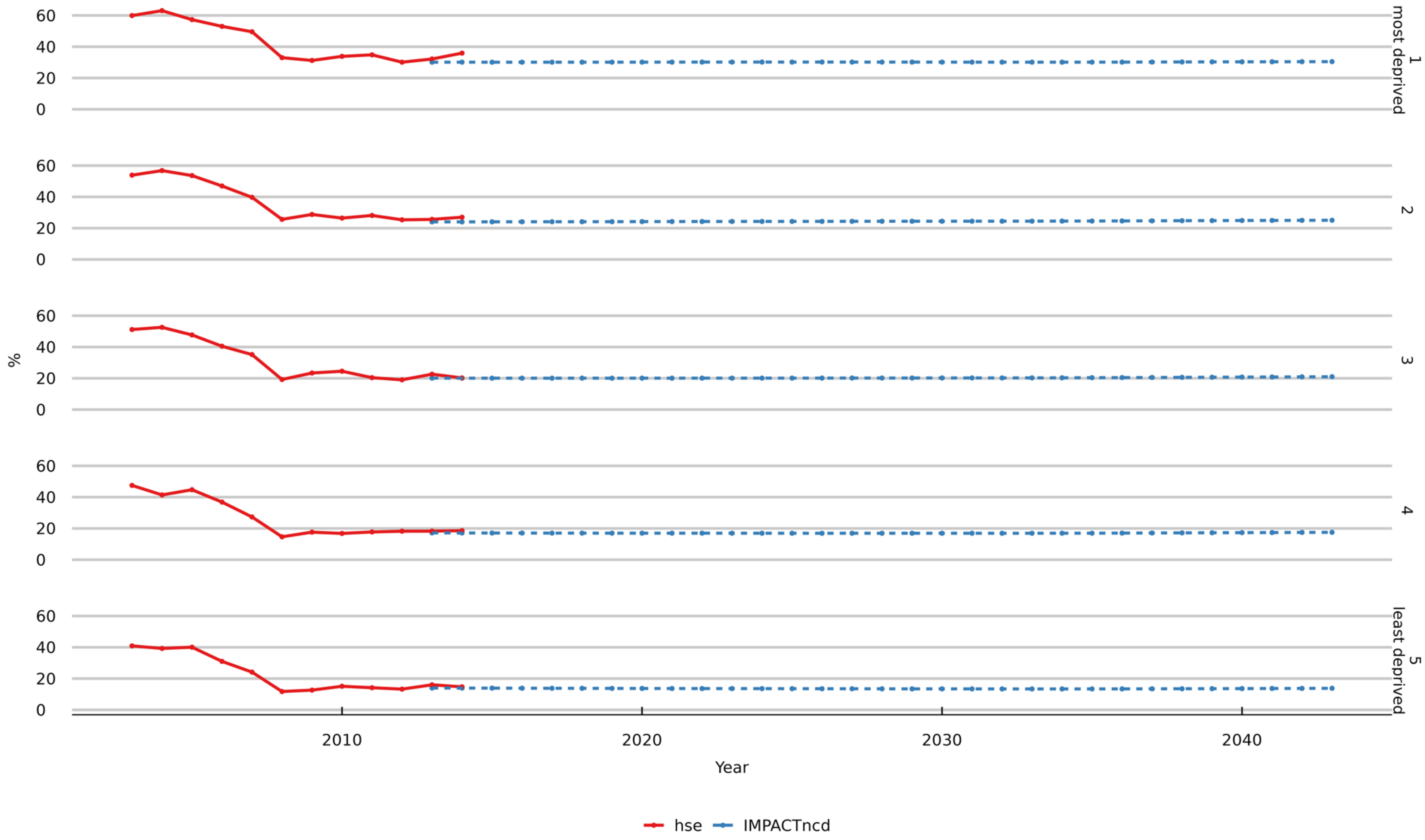

Figure B-37 - Validation: Exposure to environmental smoking by year and age group

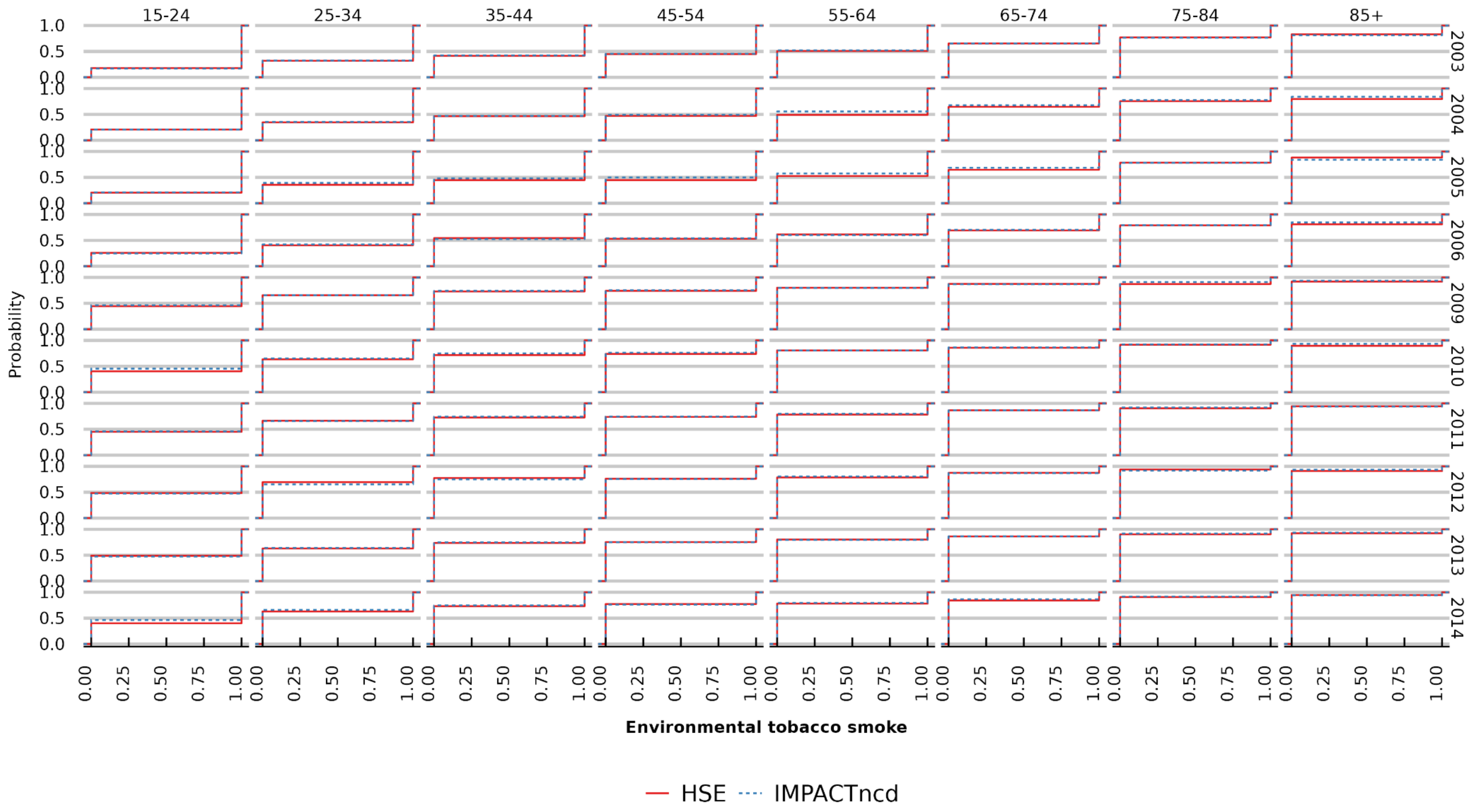

Figure B-38 - Validation: Exposure to environmental smoking by quintile groups of IMD and age group

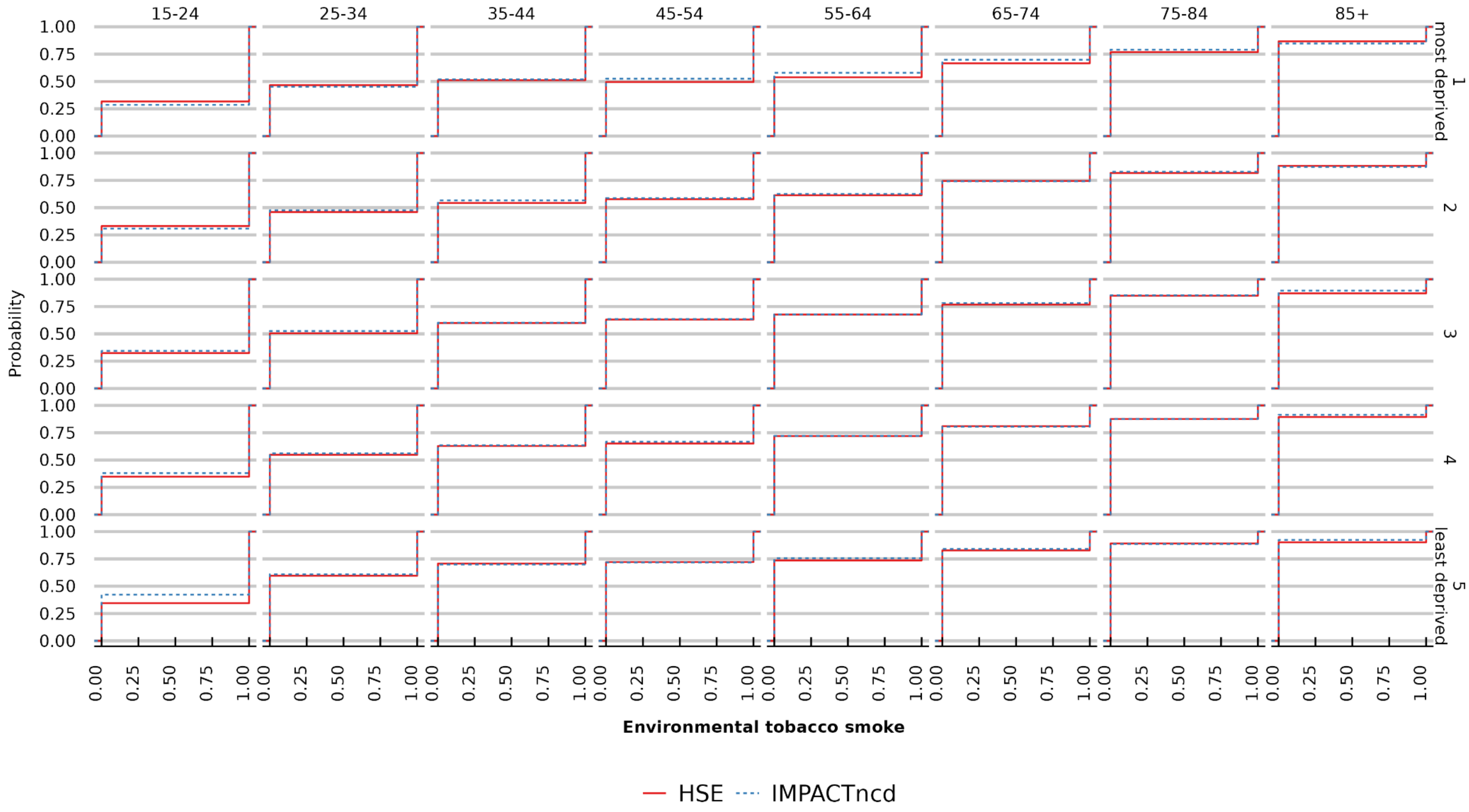

Supplementary results

Table of contents

1. Risk factor exposure improvement scenarios 1

2. Projected population sizes (base-case scenario) 3

3. Summary table of results 4

4. Absolute differences compared to base-case of continuing trends– tables 5

5. Absolute differences compared to base-case scenario– results by age-group 15

6. Absolute differences compared to base-case scenario– results by birth cohort 16

7. Relative change compared to base-case scenario 17

10% improvement in risk factor exposure levels 17

Theoretical minimum risk exposure levels 18

Relative change compared to base-case scenario - tables 19

8. Sensitivity analyses 25

No effect of improved risk factor exposures on mortality 25

Alternative major illness thresholds 26

Indirect effect of BMI through SBP and total cholesterol 27

1. Risk factor exposure improvement scenarios

Figure 1-1 – Mean exposure levels (unstandardised) under continuing trends (base-case) and 10% improvement scenarios, by quintile group of IMD

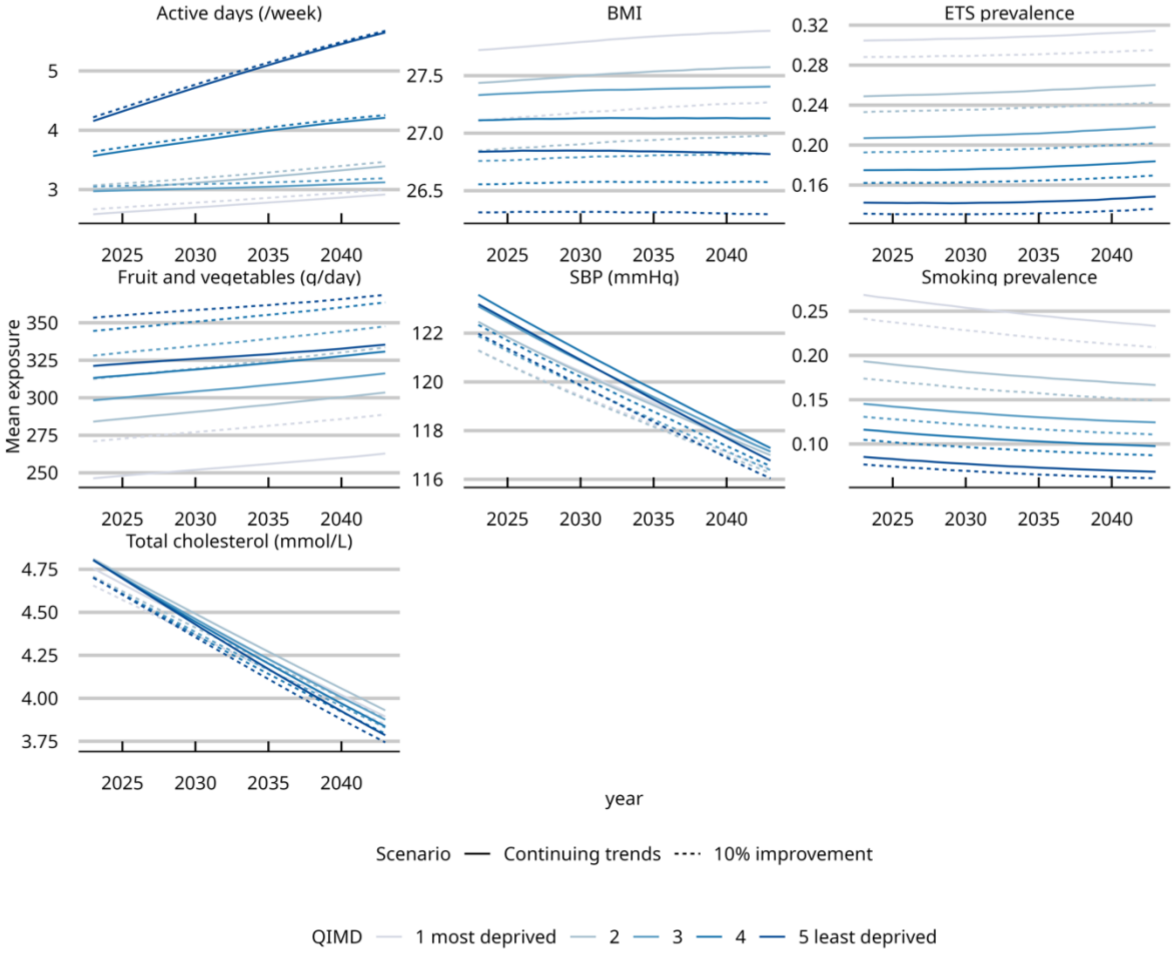

BMI = Body mass index; ETS = Environmental tobacco smoke; SBP = Systolic blood pressure

Figure 1-2 – Mean exposure levels (unstandardised) under continuing trends (base-case) and 10% improvement scenarios, by 20-year age-group

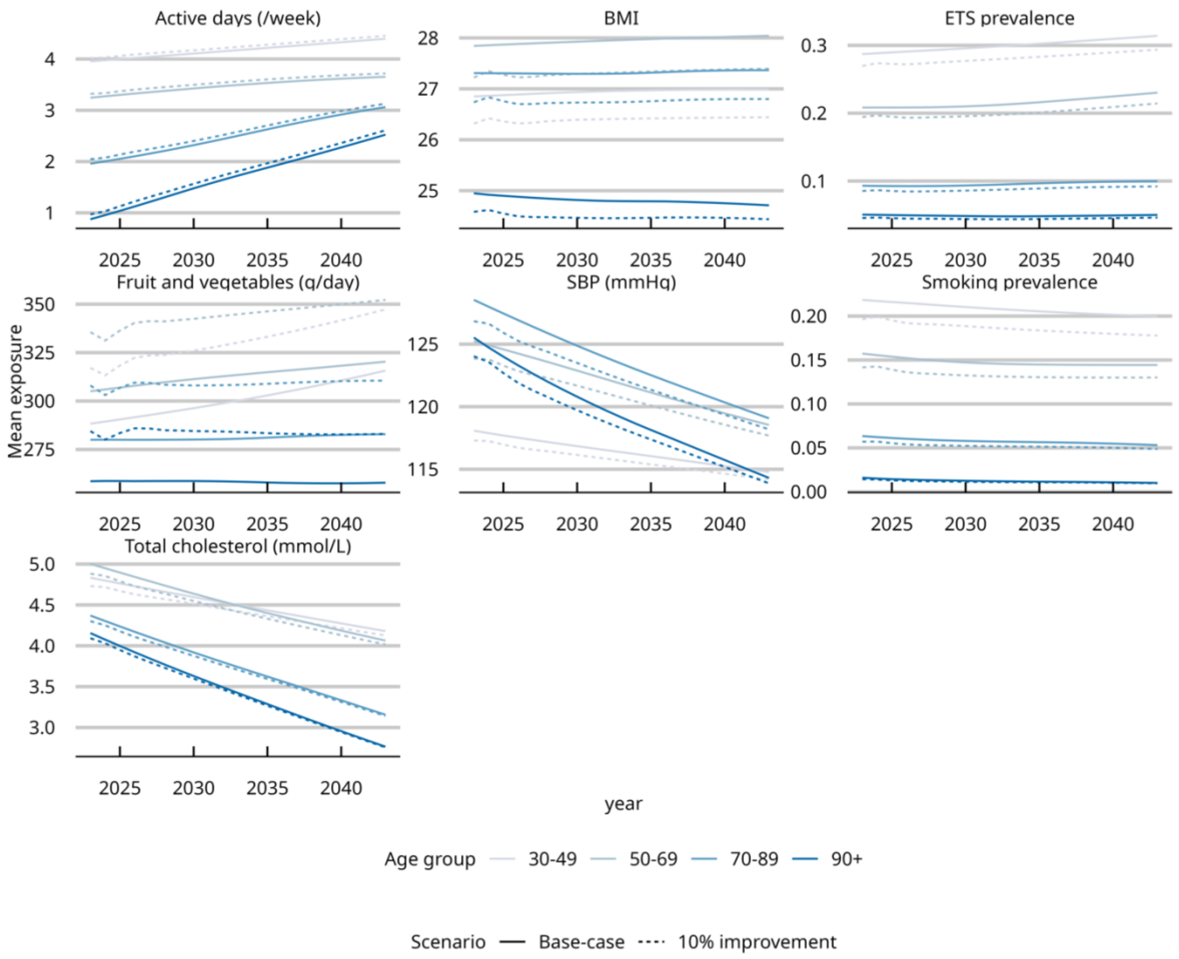

All curves are smoothed; BMI = Body mass index; ETS = Environmental tobacco smoke; SBP = Systolic blood pressure

2. Projected population sizes (base-case scenario)

Figure 2-1 - Projected population size (in millions) by 10-year age groups in selected years – base-case scenario

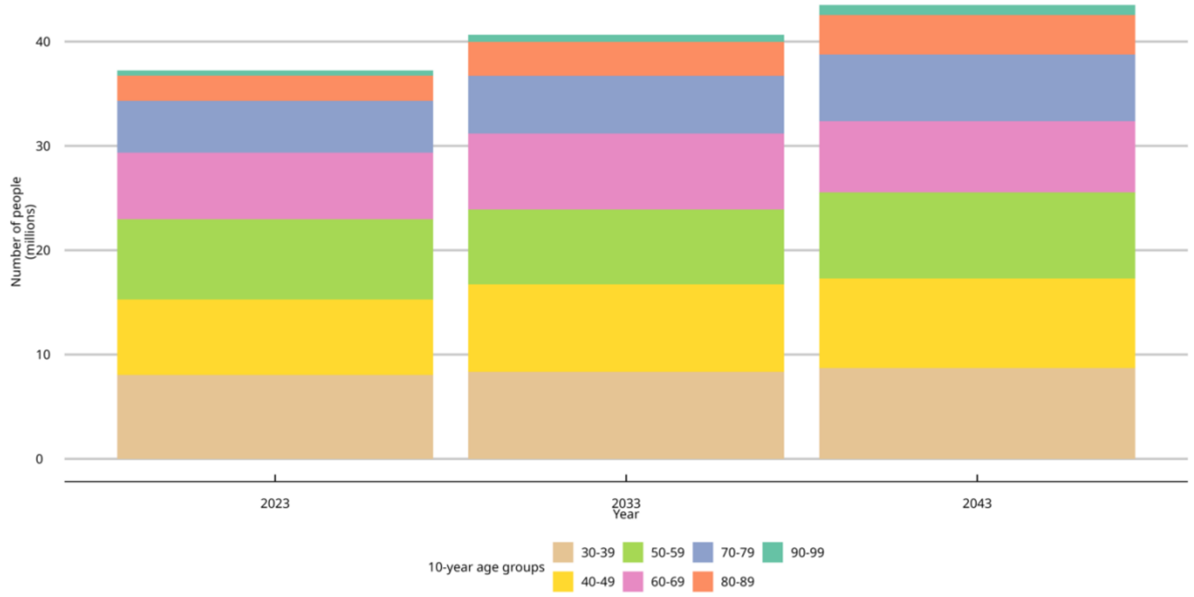

Source data are provided as a Source Data file.

Figure 2-2 - Proportion of population by 10-year age groups in selected years – base-case scenario*

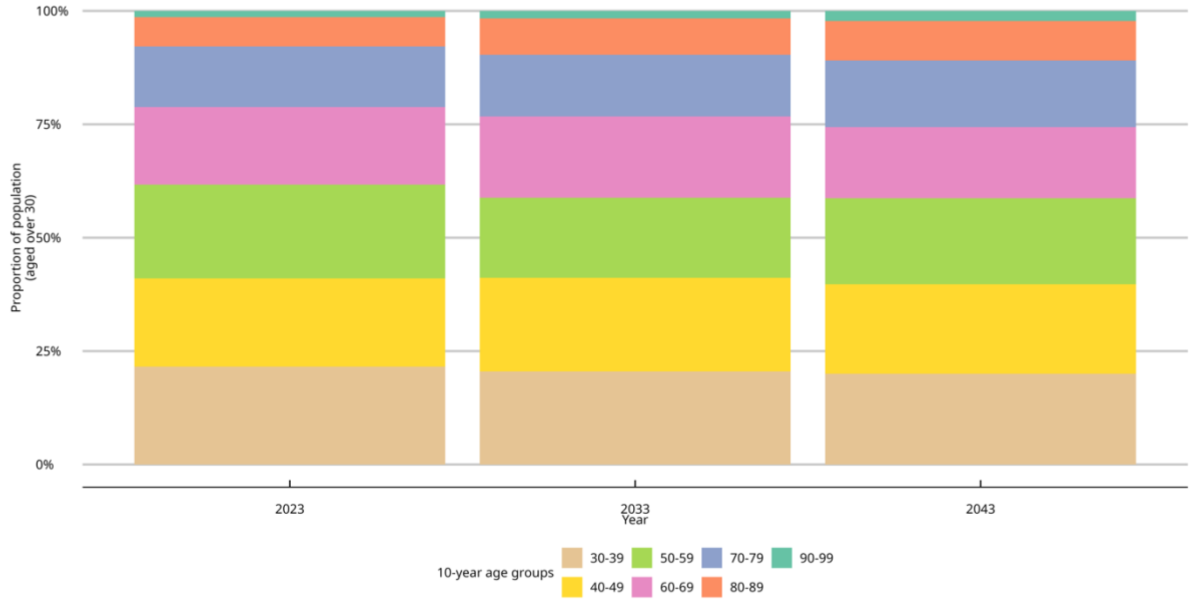

*30 years old and over. Source data are the same as for Figure 2-1, provided in the Source Data file.

3. Summary table of results

Table 3-1 - Summary table of 2043 major illness prevalence rates and total case years lived with major illness prevented or postponed by 2043 by scenario

Reduction level	Scenario	Prevalence in 2043 (%)	Absolute difference in 2043 prevalence rate compared to base-case (percentage points)	Relative change in 2043 prevalence rate compared to base-case (%)	Case years prevented or postponed by 2043 compared to base-case+ (millions)
**Continuing trends**	Base-case	29.8 (29.2, 30.4)	/	/	/
**Theoretical minimum risk**	BMI	28.1 (27.4,28.7)	-1.7 (-1.1,-2.5)	-5.6 (-3.9,-8.1)	6.7 (9.5,4.4)
	Fruit & veg	29.7 (29.1,30.3)	-0.1 (-0.1,-0.2)	-0.3 (-0.2,-0.5)	0.3 (0.5,0.1)
	Physical activity	29.7 (29.1,30.4)	0 (0.2,-0.2)	0 (0.5,-0.6)	-1.1 (0.1,-2.6)
	SBP	29.3 (28.7,30.0)	-0.5 (-0.2,-0.9)	-1.6 (-0.5,-3)	1.7 (3.3,0.4)
	Smoking	29.7 (29.2,30.4)	0 (0,-0.1)	-0.2 (0.1,-0.4)	-0.9 (-0.3,-1.3)
	Total cholesterol	29.7 (29.1,30.3)	-0.1 (0,-0.2)	-0.2 (-0.1,-0.5)	0.2 (0.5,0.1)
	All	27.8 (27.3,28.4)	-2 (-1.3,-2.7)	-6.6 (-4.5,-9)	5.5 (8.6,2.5)
**10%**	BMI	29.5 (29.0,30.0)	-0.3 (-0.2,-0.4)	-0.9 (-0.5,-1.4)	1.1 (1.7,0.7)
	Fruit & veg	29.8 (29.2,30.4)	0 (0,0)	0 (0,0)	0 (0,0)
	Physical activity	29.8 (29.2,30.4)	0 (0,0)	0 (0,0)	0 (0,-0.1)
	SBP	29.8 (29.1,30.3)	0 (0,-0.1)	-0.1 (0,-0.3)	0.1 (0.3,0)
	Smoking	29.8 (29.2,30.4)	0 (0,0)	0.1 (0.1,0)	-0.2 (-0.1,-0.3)
	Total cholesterol	29.8 (29.2,30.4)	0 (0,0)	0 (0,-0.1)	0 (0.1,0)
	All	29.5 (29.0,30.1)	-0.3 (-0.2,-0.4)	-0.9 (-0.5,-1.3)	0.9 (1.4,0.4)

For all scenarios, prevalence rate of major illness in 2023 was 25.7% (25.2%,26.1%) + Total case years lived with major illness by 2043 under the continuing trends (base-case) scenario: 238.6 million (233.8,242.5); BMI = Body mass index; SBP = Systolic blood pressure

4. Absolute differences compared to base-case of continuing trends– tables

Table 4-1 - Absolute difference (percentage points) in major illness prevalence by scenario and year under a 10% improvement in risk factors compared to a base-case scenario of continuing trends

Year	All (pp)	BMI (pp)	BMI sensitivity (pp)	Fruit & veg (pp)	Physical activity (pp)	SBP (pp)	Smoking (pp)	Total cholesterol (pp)
**2023**	0 (0,0)	0 (0,0)	0 (0,0)	0 (0,0)	0 (0,0)	0 (0,0)	0 (0,0)	0 (0,0)
**2024**	0 (0,0)	0 (0,0)	0 (0,0)	0 (0,0)	0 (0,0)	0 (0,0)	0 (0,0)	0 (0,0)
**2025**	0 (0,0)	0 (0,0)	0 (0,0)	0 (0,0)	0 (0,0)	0 (0,0)	0 (0,0)	0 (0,0)
**2026**	0 (-0.1,0)	0 (-0.1,0)	0 (0,0)	0 (0,0)	0 (0,0)	0 (0,0)	0 (0,0)	0 (0,0)
**2027**	0 (-0.1,0)	0 (-0.1,0)	0 (-0.1,0)	0 (0,0)	0 (0,0)	0 (0,0)	0 (0,0)	0 (0,0)
**2028**	-0.1 (-0.1,0)	-0.1 (-0.1,0)	0 (-0.1,0)	0 (0,0)	0 (0,0)	0 (0,0)	0 (0,0)	0 (0,0)
**2029**	-0.1 (-0.2,-0.1)	-0.1 (-0.1,0)	-0.1 (-0.1,0)	0 (0,0)	0 (0,0)	0 (-0.1,0)	0 (0,0)	0 (0,0)
**2030**	-0.1 (-0.2,-0.1)	-0.1 (-0.2,-0.1)	-0.1 (-0.1,0)	0 (0,0)	0 (0,0)	0 (-0.1,0)	0 (0,0)	0 (0,0)
**2031**	-0.2 (-0.2,-0.1)	-0.1 (-0.2,-0.1)	-0.1 (-0.2,-0.1)	0 (0,0)	0 (0,0)	0 (-0.1,0)	0 (0,0)	0 (0,0)
**2032**	-0.2 (-0.3,-0.1)	-0.2 (-0.2,-0.1)	-0.1 (-0.2,-0.1)	0 (0,0)	0 (0,0)	0 (-0.1,0)	0 (0,0)	0 (0,0)
**2033**	-0.2 (-0.3,-0.1)	-0.2 (-0.3,-0.1)	-0.1 (-0.2,-0.1)	0 (0,0)	0 (0,0)	0 (-0.1,0)	0 (0,0)	0 (0,0)
**2034**	-0.2 (-0.3,-0.1)	-0.2 (-0.3,-0.1)	-0.2 (-0.2,-0.1)	0 (0,0)	0 (0,0)	0 (-0.1,0)	0 (0,0)	0 (0,0)
**2035**	-0.2 (-0.3,-0.1)	-0.2 (-0.3,-0.1)	-0.2 (-0.2,-0.1)	0 (0,0)	0 (0,0)	0 (-0.1,0)	0 (0,0)	0 (0,0)
**2036**	-0.2 (-0.3,-0.2)	-0.2 (-0.3,-0.1)	-0.2 (-0.3,-0.1)	0 (0,0)	0 (0,0)	0 (-0.1,0)	0 (0,0)	0 (0,0)
**2037**	-0.2 (-0.3,-0.2)	-0.2 (-0.4,-0.1)	-0.2 (-0.3,-0.1)	0 (0,0)	0 (0,0)	0 (-0.1,0)	0 (0,0)	0 (0,0)
**2038**	-0.3 (-0.4,-0.2)	-0.2 (-0.4,-0.2)	-0.2 (-0.3,-0.1)	0 (0,0)	0 (0,0)	0 (-0.1,0)	0 (0,0)	0 (0,0)
**2039**	-0.3 (-0.4,-0.2)	-0.3 (-0.4,-0.2)	-0.2 (-0.3,-0.1)	0 (0,0)	0 (0,0)	0 (-0.1,0)	0 (0,0)	0 (0,0)
**2040**	-0.3 (-0.4,-0.2)	-0.3 (-0.4,-0.2)	-0.2 (-0.3,-0.1)	0 (0,0)	0 (0,0)	0 (-0.1,0)	0 (0,0)	0 (0,0)
**2041**	-0.3 (-0.4,-0.2)	-0.3 (-0.4,-0.2)	-0.2 (-0.3,-0.1)	0 (0,0)	0 (0,0)	0 (-0.1,0)	0 (0,0)	0 (0,0)
**2042**	-0.3 (-0.4,-0.2)	-0.3 (-0.4,-0.2)	-0.2 (-0.3,-0.2)	0 (0,0)	0 (0,0)	0 (-0.1,0)	0 (0,0)	0 (0,0)
**2043**	-0.3 (-0.4,-0.2)	-0.3 (-0.4,-0.2)	-0.2 (-0.3,-0.2)	0 (0,0)	0 (0,0)	0 (-0.1,0)	0 (0,0)	0 (0,0)

BMI = Body mass index; SBP = Systolic blood pressure

Table 4-2- Absolute difference (percentage points) in major illness prevalence by scenario and year under theoretical minimum risk factor exposure levels compared to a base-case scenario of continuing trends

Year	All (pp)	BMI (pp)	Fruit & veg (pp)	Physical activity (pp)	SBP (pp)	Smoking (pp)	Total cholesterol (pp)
**2023**	0 (0,0)	0 (0,0)	0 (0,0)	0 (0,0)	0 (0,0)	0 (0,0)	0 (0,0)
**2024**	0 (-0.1,0)	0 (-0.1,0)	0 (0,0)	0 (0,0)	0 (-0.1,0)	0 (0,0)	0 (0,0)
**2025**	-0.1 (-0.2,0)	0 (-0.1,0)	0 (0,0)	0 (0,0)	0 (-0.1,0)	0 (0,0)	0 (0,0)
**2026**	-0.2 (-0.4,-0.1)	0 (-0.2,0)	0 (0,0)	0 (-0.1,0)	-0.1 (-0.2,0)	0 (-0.1,0)	0 (0,0)
**2027**	-0.3 (-0.6,-0.1)	-0.1 (-0.3,0)	0 (0,0)	0 (-0.1,0.1)	-0.1 (-0.3,0)	-0.1 (-0.1,0)	0 (0,0)
**2028**	-0.5 (-0.8,-0.3)	-0.3 (-0.5,-0.1)	0 (-0.1,0)	0 (-0.1,0.1)	-0.2 (-0.4,-0.1)	-0.1 (-0.1,0)	0 (0,0)
**2029**	-0.7 (-1,-0.4)	-0.4 (-0.7,-0.1)	0 (-0.1,0)	0 (-0.2,0.1)	-0.2 (-0.4,-0.1)	-0.1 (-0.1,0)	0 (-0.1,0)
**2030**	-0.8 (-1.1,-0.5)	-0.6 (-0.8,-0.3)	0 (-0.1,0)	0 (-0.2,0.1)	-0.3 (-0.5,-0.1)	-0.1 (-0.1,0)	0 (-0.1,0)
**2031**	-0.9 (-1.3,-0.6)	-0.7 (-1,-0.4)	-0.1 (-0.1,0)	0 (-0.1,0.2)	-0.3 (-0.5,-0.1)	0 (-0.1,0)	0 (-0.1,0)
**2032**	-1.1 (-1.5,-0.7)	-0.8 (-1.2,-0.5)	-0.1 (-0.1,0)	0 (-0.1,0.2)	-0.3 (-0.6,-0.2)	-0.1 (-0.1,0)	0 (-0.1,0)
**2033**	-1.2 (-1.6,-0.8)	-1 (-1.3,-0.6)	-0.1 (-0.1,0)	0 (-0.1,0.2)	-0.4 (-0.6,-0.2)	-0.1 (-0.1,0)	0 (-0.1,0)
**2034**	-1.3 (-1.8,-0.9)	-1.1 (-1.5,-0.7)	-0.1 (-0.1,0)	0 (-0.2,0.1)	-0.4 (-0.7,-0.2)	-0.1 (-0.1,0)	-0.1 (-0.1,0)
**2035**	-1.5 (-1.9,-1)	-1.2 (-1.6,-0.8)	-0.1 (-0.1,-0.1)	0 (-0.1,0.2)	-0.4 (-0.7,-0.2)	-0.1 (-0.1,0)	-0.1 (-0.1,0)
**2036**	-1.5 (-2,-1)	-1.2 (-1.7,-0.8)	-0.1 (-0.1,-0.1)	0 (-0.2,0.2)	-0.4 (-0.8,-0.2)	-0.1 (-0.1,0)	-0.1 (-0.1,0)
**2037**	-1.6 (-2.1,-1.1)	-1.3 (-1.8,-0.9)	-0.1 (-0.1,-0.1)	0 (-0.1,0.2)	-0.4 (-0.8,-0.2)	-0.1 (-0.1,0)	-0.1 (-0.1,0)
**2038**	-1.7 (-2.2,-1.2)	-1.4 (-1.9,-1)	-0.1 (-0.1,-0.1)	0 (-0.2,0.2)	-0.4 (-0.8,-0.2)	-0.1 (-0.1,0)	-0.1 (-0.1,0)
**2039**	-1.8 (-2.3,-1.2)	-1.5 (-2.1,-1)	-0.1 (-0.1,-0.1)	0 (-0.2,0.2)	-0.5 (-0.8,-0.2)	-0.1 (-0.1,0)	-0.1 (-0.1,0)
**2040**	-1.8 (-2.4,-1.3)	-1.5 (-2.2,-1)	-0.1 (-0.1,-0.1)	0 (-0.2,0.2)	-0.5 (-0.9,-0.2)	-0.1 (-0.1,0)	-0.1 (-0.1,0)
**2041**	-1.9 (-2.5,-1.3)	-1.6 (-2.3,-1.1)	-0.1 (-0.1,-0.1)	0 (-0.2,0.2)	-0.5 (-0.9,-0.1)	-0.1 (-0.1,0)	-0.1 (-0.1,0)
**2042**	-2 (-2.6,-1.4)	-1.6 (-2.4,-1.1)	-0.1 (-0.1,-0.1)	0 (-0.2,0.2)	-0.5 (-0.9,-0.2)	-0.1 (-0.1,0)	-0.1 (-0.1,0)
**2043**	-2 (-2.7,-1.3)	-1.7 (-2.5,-1.1)	-0.1 (-0.2,-0.1)	0 (-0.2,0.2)	-0.5 (-0.9,-0.2)	0 (-0.1,0)	-0.1 (-0.2,0)

BMI = Body mass index; SBP = Systolic blood pressure

Table 4-3 - Absolute difference (cases per 10,000 persons) in major illness incidence rates by scenario and year under a 10% improvement in risk factors compared to a base-case scenario of continuing trends

Year	All	BMI	BMI sensitivity	Fruit & veg	Physical activity	SBP	Smoking	Total cholesterol
**2023**	0 (-0.2,0)	0 (-0.1,0.2)	0 (0,0)	0 (0,0)	0 (0,0)	0 (-0.1,0)	0 (-0.1,0)	0 (0,0)
**2024**	-0.2 (-1.6,0.2)	0 (-1.3,0.2)	0 (-1,0)	0 (0,0)	0 (-0.1,0)	0 (-0.9,0)	0 (-0.5,0.3)	0 (0,0)
**2025**	-0.6 (-2.5,0)	-0.3 (-2.2,0.2)	0 (-1.6,0)	0 (-0.1,0)	0 (-0.1,0)	-0.2 (-1.4,0)	-0.1 (-0.6,0.3)	0 (-0.3,0)
**2026**	-2 (-4.3,-0.5)	-1.3 (-3.9,-0.1)	-0.7 (-2.9,0)	0 (-0.2,0)	0 (-0.2,0)	-0.8 (-1.9,0)	-0.2 (-0.9,0.1)	0 (-0.5,0)
**2027**	-3.7 (-6.6,-1.5)	-2.7 (-5,-0.7)	-1.4 (-3.8,0)	-0.1 (-0.3,0)	-0.1 (-0.3,0)	-1.2 (-3.2,-0.3)	-0.5 (-1.3,0.1)	-0.1 (-0.5,0)
**2028**	-5.1 (-8.3,-2.6)	-3.6 (-7.6,-1.4)	-2.4 (-5.1,-0.4)	-0.1 (-0.4,0)	-0.1 (-0.3,0.1)	-1.6 (-3,-0.7)	-0.7 (-1.5,0)	-0.1 (-0.6,0)
**2029**	-5.9 (-8.5,-3.3)	-4.4 (-7.4,-2.1)	-3.1 (-5.1,-1.1)	-0.1 (-0.4,0)	-0.1 (-0.3,0.1)	-1.6 (-3.3,-0.6)	-0.8 (-1.5,-0.1)	-0.2 (-0.7,0.1)
**2030**	-6.5 (-9,-4.3)	-4.7 (-7.5,-2.6)	-3.6 (-5.6,-1.9)	-0.1 (-0.5,0.1)	-0.1 (-0.4,0.1)	-1.7 (-3.1,-0.5)	-0.9 (-1.7,-0.4)	-0.2 (-0.7,0.1)
**2031**	-6.9 (-9.5,-4.8)	-4.9 (-7.8,-2.6)	-3.7 (-5.7,-2.1)	-0.2 (-0.4,0.1)	-0.1 (-0.3,0.1)	-1.6 (-2.7,-0.4)	-1 (-1.8,-0.3)	-0.2 (-0.6,0.1)
**2032**	-6.9 (-9.9,-4.4)	-5.1 (-8.2,-2.7)	-4 (-5.9,-2)	-0.1 (-0.5,0.2)	-0.1 (-0.4,0.1)	-1.4 (-2.8,-0.3)	-1.1 (-1.9,-0.1)	-0.2 (-0.6,0.2)
**2033**	-7 (-10.2,-4.3)	-5.4 (-8.5,-2.4)	-4.2 (-7,-2)	-0.2 (-0.5,0.1)	-0.1 (-0.4,0.2)	-1.3 (-2.9,-0.2)	-1.1 (-1.9,-0.4)	-0.2 (-0.6,0.1)
**2034**	-6.7 (-9.5,-4.5)	-5.2 (-8.1,-2.4)	-4.2 (-6.3,-2)	-0.1 (-0.4,0.1)	-0.1 (-0.3,0.2)	-1.1 (-2.4,-0.2)	-1 (-1.9,-0.2)	-0.2 (-0.7,0.2)
**2035**	-6.5 (-9,-4.2)	-4.9 (-7.7,-2.7)	-4 (-6.4,-2.6)	-0.2 (-0.5,0.1)	-0.1 (-0.4,0.2)	-1.1 (-2.1,0.2)	-1.1 (-2,-0.2)	-0.2 (-0.7,0.1)
**2036**	-6.4 (-8.8,-3.4)	-4.7 (-7.9,-2.2)	-3.8 (-6.4,-2.1)	-0.1 (-0.5,0.2)	-0.1 (-0.4,0.3)	-0.9 (-2.3,0.1)	-0.9 (-1.8,0.1)	-0.2 (-0.6,0.3)
**2037**	-5.9 (-8.3,-3.3)	-4.8 (-7.7,-2.4)	-3.9 (-6.2,-2.2)	-0.1 (-0.4,0.2)	0 (-0.3,0.2)	-0.7 (-2.2,0.4)	-0.8 (-1.7,0.2)	-0.1 (-0.6,0.3)
**2038**	-5.9 (-8.7,-3.3)	-4.6 (-7.8,-2.2)	-3.9 (-6.1,-1.9)	-0.2 (-0.5,0.2)	-0.1 (-0.3,0.3)	-0.8 (-2.2,0.3)	-0.7 (-1.6,0)	-0.1 (-0.6,0.1)
**2039**	-5.5 (-8.4,-2.8)	-4.3 (-7.2,-1.7)	-3.7 (-6.4,-1.8)	-0.1 (-0.5,0.3)	-0.1 (-0.3,0.2)	-0.6 (-2.1,0.6)	-0.7 (-1.6,0.3)	-0.1 (-0.7,0.1)
**2040**	-5.2 (-7.9,-2)	-4.3 (-7.2,-2.1)	-3.6 (-6.3,-1.8)	-0.1 (-0.4,0.2)	0 (-0.3,0.3)	-0.5 (-1.8,0.5)	-0.6 (-1.4,0.5)	-0.1 (-0.7,0.3)
**2041**	-4.9 (-8,-2.6)	-4.2 (-7.2,-1.8)	-3.7 (-6.3,-1.9)	-0.1 (-0.6,0.2)	0 (-0.4,0.4)	-0.4 (-2.1,0.5)	-0.6 (-1.4,0.4)	-0.1 (-0.6,0.3)
**2042**	-4.9 (-7.9,-2)	-4.2 (-7.1,-1.2)	-3.7 (-6.4,-1.1)	-0.1 (-0.5,0.2)	0 (-0.3,0.4)	-0.5 (-1.9,0.5)	-0.4 (-1.3,0.5)	-0.1 (-0.6,0.2)
**2043**	-4.4 (-7.9,-2)	-3.5 (-6.8,-1.1)	-3.1 (-6.6,-1.3)	-0.1 (-0.5,0.2)	-0.1 (-0.4,0.4)	-0.2 (-1.5,0.5)	-0.4 (-1.6,0.8)	-0.1 (-0.7,0.3)

BMI = Body mass index; SBP = Systolic blood pressure

Table 4-4- Absolute difference (cases per 10,000 persons) in major illness incidence rates by scenario and year under theoretical minimum risk factor exposure levels compared to a base-case scenario of continuing trends

Year	All	BMI	Fruit & veg	Physical activity	SBP	Smoking	Total cholesterol
**2023**	-0.4 (-0.8,-0.1)	0 (0,0)	0 (0,0)	0 (0,0)	-0.3 (-0.6,0)	-0.1 (-0.4,0)	0 (0,0)
**2024**	-3.3 (-11.8,-1.9)	0 (-7,0)	0 (-0.5,0)	0 (-3.2,0)	-0.3 (-6.1,0)	-2.6 (-3.4,-1.7)	0 (-0.7,0)
**2025**	-7 (-18.1,-2.6)	0 (-9.2,0)	-0.2 (-1.2,0)	0 (-4.3,0)	-1.4 (-9.1,0)	-2.5 (-3.7,-1.5)	0 (-1.6,0)
**2026**	-16.5 (-27.7,-6.4)	-5.2 (-16.4,0)	-0.9 (-2.3,0)	-1.2 (-5.5,0)	-5.5 (-13.4,0)	-2.7 (-3.8,-1.6)	-0.4 (-2.5,0)
**2027**	-26.1 (-35.7,-10.5)	-10.7 (-21.3,-0.2)	-1.7 (-3.5,-0.5)	-3.9 (-5.6,0)	-8.9 (-19.7,-2.4)	-2.8 (-4.3,-1.6)	-1 (-3.1,0)
**2028**	-33.1 (-46.4,-22.1)	-17.2 (-30.9,-3.9)	-2.2 (-3.9,-1)	-4.6 (-6.4,-0.4)	-12 (-21.1,-5.8)	-3 (-4.8,-2)	-1.3 (-3.2,-0.1)
**2029**	-38.2 (-47.5,-27.6)	-21.6 (-33.5,-11.2)	-2.5 (-4,-1.3)	-4.7 (-6.7,-1.5)	-13.2 (-20.3,-6.7)	-3.4 (-5.1,-1.7)	-1.5 (-3.5,-0.2)
**2030**	-40.3 (-50,-30.8)	-24 (-34.3,-15.3)	-2.5 (-4.1,-1.3)	-4.9 (-6.7,-3.3)	-12.6 (-18.5,-5.8)	-3.9 (-5.9,-2)	-1.5 (-3.3,-0.2)
**2031**	-41.2 (-53.2,-31.9)	-26.3 (-36.8,-18.5)	-2.7 (-4.6,-1.1)	-5.1 (-8.3,-2.6)	-11.2 (-17.9,-6.1)	-4.5 (-6.1,-3.2)	-1.4 (-2.9,-0.3)
**2032**	-44.1 (-53.8,-34.2)	-28.1 (-39.7,-19)	-2.6 (-4.1,-0.9)	-5.5 (-8.3,-3.1)	-10.8 (-18.1,-5.4)	-4.7 (-6.6,-2.9)	-1.5 (-3.2,-0.1)
**2033**	-46 (-57.7,-35.6)	-28.9 (-41.6,-19.7)	-2.6 (-4.2,-1.2)	-6.1 (-9,-4.5)	-9.5 (-17,-5.4)	-5.3 (-7.5,-3.1)	-1.5 (-3.3,-0.3)
**2034**	-46.4 (-55.8,-34.5)	-30.3 (-39.1,-19.2)	-2.5 (-4.4,-0.7)	-6 (-8.1,-4.2)	-9.4 (-15.9,-3.8)	-5.8 (-7.7,-3.6)	-1.2 (-3.4,-0.2)
**2035**	-45.5 (-54.1,-34.4)	-29.9 (-38.2,-19.2)	-2.6 (-4.2,-1.2)	-5.8 (-7.9,-3.5)	-8.4 (-14.5,-3.2)	-6 (-8.1,-4)	-1.3 (-3.3,0)
**2036**	-44.1 (-54.2,-34)	-27.9 (-39,-19.5)	-2.1 (-4.1,-0.5)	-5.6 (-7.4,-3.4)	-7.7 (-13.7,-2.7)	-6.7 (-8.8,-4.4)	-1.1 (-2.9,0.1)
**2037**	-43.5 (-52.4,-34.5)	-29.4 (-38.3,-19.8)	-2.3 (-4,-0.8)	-5.2 (-7.5,-3.4)	-7 (-12.4,-2.7)	-6.6 (-8.7,-4.6)	-1.1 (-2.5,0.1)
**2038**	-43.1 (-52.3,-33.1)	-28.9 (-38.9,-19.7)	-2.3 (-3.9,-0.7)	-5.3 (-7.6,-2.6)	-6.3 (-12.6,-1.7)	-6.7 (-9.3,-4.2)	-1.1 (-3,-0.1)
**2039**	-42 (-52.3,-31.1)	-28.6 (-38.7,-18.3)	-2.2 (-3.7,-0.4)	-4.5 (-7.1,-2.7)	-6 (-11.4,-0.6)	-6.4 (-8.9,-4.3)	-0.9 (-3.3,0.1)
**2040**	-41 (-50.1,-29.6)	-28.2 (-38.4,-17.8)	-1.9 (-3.5,-0.6)	-4.6 (-6.9,-2.1)	-4.9 (-12,-0.3)	-6.7 (-8.6,-4.3)	-0.8 (-2.6,0.3)
**2041**	-40.4 (-51.4,-27.5)	-27.8 (-38.5,-18.6)	-2 (-3.7,-0.6)	-4.4 (-7.4,-1.9)	-4.6 (-10,-0.2)	-6.7 (-8.6,-4)	-0.9 (-3.1,0.2)
**2042**	-38.2 (-48.6,-28.4)	-27.3 (-36.9,-17.4)	-1.9 (-3.2,-0.5)	-4.2 (-6,-1.5)	-4.1 (-8.4,-0.3)	-6.1 (-8.6,-3.3)	-0.6 (-2.3,0.3)
**2043**	-36.9 (-45.2,-25.6)	-26.7 (-35.4,-16.7)	-1.7 (-3.7,-0.3)	-3.9 (-6.5,-1.4)	-3.5 (-8.5,0.7)	-6.4 (-8.7,-3.9)	-0.8 (-3,0.3)

BMI = Body mass index; SBP = Systolic blood pressure

Table 4-5 - Absolute difference (deaths per 10,000 persons) in all-cause mortality rates by scenario and year under a 10% improvement in risk factors compared to a base-case scenario of continuing trends

Year	All	BMI	BMI sensitivity	Fruit & veg	Physical activity	SBP	Smoking	Total cholesterol
**2023**	0 (-0.1,0)	0 (0,0)	0 (0,0)	0 (0,0)	0 (0,0)	0 (0,0)	0 (-0.1,0)	0 (0,0)
**2024**	-0.1 (-0.4,0.1)	0 (-0.1,0)	0 (-0.1,0)	0 (0,0)	0 (0,0)	0 (-0.2,0)	0 (-0.2,0.1)	0 (0,0)
**2025**	-0.2 (-1.1,0.1)	-0.1 (-0.6,0)	0 (-0.1,0)	0 (-0.1,0)	0 (-0.1,0)	0 (-1.1,0)	0 (-0.4,0.2)	0 (-0.2,0)
**2026**	-0.6 (-1.6,0)	-0.2 (-0.7,0)	0 (-0.2,0)	0 (-0.1,0)	0 (-0.3,0)	-0.2 (-1.2,0)	-0.1 (-0.9,0.1)	0 (-0.3,0)
**2027**	-1.1 (-2.6,-0.4)	-0.4 (-1.2,-0.1)	-0.1 (-0.3,0)	0 (-0.2,0)	-0.1 (-0.4,0)	-0.5 (-1.6,0)	-0.3 (-1,0.1)	0 (-0.3,0)
**2028**	-1.9 (-3.6,-0.8)	-0.7 (-1.6,-0.3)	-0.2 (-0.4,0)	-0.1 (-0.2,0)	-0.2 (-0.4,0)	-0.9 (-1.9,-0.2)	-0.6 (-1.1,0.1)	-0.1 (-0.4,0)
**2029**	-2.2 (-3.4,-1.3)	-0.8 (-1.7,-0.3)	-0.2 (-0.5,-0.1)	-0.1 (-0.2,0)	-0.2 (-0.4,0)	-1 (-1.9,-0.3)	-0.7 (-1.3,-0.2)	-0.1 (-0.4,0)
**2030**	-2.4 (-3.7,-1.2)	-0.9 (-2,-0.4)	-0.3 (-1.1,-0.1)	0 (-0.2,0)	-0.2 (-0.4,0)	-1 (-1.7,-0.3)	-0.7 (-1.6,-0.2)	-0.1 (-0.3,0)
**2031**	-2.6 (-4,-1.4)	-1 (-2.1,-0.3)	-0.3 (-1.2,0)	-0.1 (-0.3,0.1)	-0.2 (-0.5,0)	-1 (-1.7,-0.3)	-0.8 (-1.5,-0.4)	-0.1 (-0.3,0)
**2032**	-3.2 (-4.4,-1.8)	-1.3 (-2.3,-0.4)	-0.7 (-1.6,-0.1)	0 (-0.2,0.1)	-0.2 (-0.4,0)	-0.9 (-1.5,-0.2)	-1.1 (-1.7,-0.5)	-0.1 (-0.3,0.1)
**2033**	-3.2 (-4.4,-2.4)	-1.3 (-2.6,-0.7)	-0.9 (-1.9,-0.4)	-0.1 (-0.2,0.1)	-0.2 (-0.4,0.1)	-0.8 (-1.5,-0.1)	-1.3 (-2,-0.6)	-0.1 (-0.3,0.1)
**2034**	-2.9 (-4.5,-1.8)	-1.3 (-2.3,-0.5)	-0.8 (-1.7,-0.3)	0 (-0.2,0.1)	-0.2 (-0.4,0.1)	-0.7 (-1.4,-0.1)	-1.2 (-1.8,-0.5)	-0.1 (-0.3,0.1)
**2035**	-2.8 (-4.1,-1.6)	-1.3 (-2.4,-0.3)	-0.8 (-1.6,-0.1)	-0.1 (-0.2,0.1)	-0.1 (-0.4,0)	-0.7 (-1.4,0.1)	-1.2 (-1.7,-0.4)	0 (-0.3,0.1)
**2036**	-2.7 (-4,-1.3)	-1.1 (-2.1,-0.3)	-0.8 (-1.7,-0.1)	0 (-0.2,0.1)	-0.1 (-0.4,0.2)	-0.5 (-1.3,0.1)	-1 (-1.8,-0.3)	0 (-0.2,0.1)
**2037**	-2.3 (-3.6,-1)	-1.1 (-2,-0.2)	-0.7 (-1.4,-0.1)	0 (-0.2,0.1)	-0.1 (-0.3,0.2)	-0.5 (-1.1,0.3)	-0.9 (-1.7,-0.1)	0 (-0.2,0.1)
**2038**	-2.1 (-3.5,-0.8)	-0.9 (-2,0)	-0.6 (-1.4,0)	0 (-0.2,0.2)	-0.1 (-0.4,0.2)	-0.3 (-1,0.2)	-0.9 (-1.8,0)	0 (-0.2,0.1)
**2039**	-1.9 (-3.3,-0.5)	-0.8 (-1.9,-0.1)	-0.6 (-1.3,0)	0 (-0.2,0.1)	-0.1 (-0.3,0.2)	-0.3 (-1,0.4)	-0.7 (-1.8,0)	0 (-0.2,0.1)
**2040**	-1.7 (-3.4,-0.6)	-0.9 (-1.9,-0.1)	-0.6 (-1.5,0.1)	0 (-0.3,0.2)	-0.1 (-0.3,0.1)	-0.2 (-0.9,0.4)	-0.7 (-1.3,0.2)	0 (-0.2,0.1)
**2041**	-1.6 (-2.6,-0.1)	-0.9 (-2,0.1)	-0.7 (-1.7,0)	0 (-0.2,0.1)	0 (-0.3,0.2)	-0.2 (-1,0.4)	-0.7 (-1.5,0.3)	0 (-0.2,0.2)
**2042**	-1.4 (-3.2,0)	-0.7 (-2,0.2)	-0.6 (-1.7,0.2)	0 (-0.2,0.2)	-0.1 (-0.3,0.2)	-0.2 (-0.8,0.6)	-0.5 (-1.3,0.3)	0 (-0.2,0.2)
**2043**	-1.3 (-3,0)	-0.7 (-1.8,0.3)	-0.6 (-1.4,0.2)	0 (-0.3,0.2)	-0.1 (-0.4,0.3)	-0.1 (-0.8,0.5)	-0.5 (-1.5,0.3)	0 (-0.2,0.1)

BMI = Body mass index; SBP = Systolic blood pressure

Table 4-6- Absolute difference (deaths per 10,000 persons) in all-cause mortality rates by scenario and year under theoretical minimum risk factor exposure levels compared to a base-case scenario of continuing trends

Year	All	BMI	Fruit & veg	Physical activity	SBP	Smoking	Total cholesterol
**2023**	-0.1 (-0.2,0)	0 (0,0)	0 (0,0)	0 (0,0)	0 (0,0)	-0.1 (-0.2,0)	0 (0,0)
**2024**	-1.1 (-2.4,-0.6)	0 (-0.5,0)	0 (-0.4,0)	0 (-0.9,0)	0 (-1.4,0)	-1 (-1.4,-0.6)	0 (-0.1,0)
**2025**	-2.2 (-7.3,-0.7)	0 (-0.8,0)	-0.1 (-0.8,0)	0 (-1.4,0)	0 (-5.5,0)	-0.9 (-5.4,-0.5)	0 (-1.1,0)
**2026**	-4.8 (-16.6,-2.1)	-0.4 (-1.3,0)	-0.5 (-1.5,0)	-0.7 (-10.9,0)	-1.6 (-6.8,0)	-1.1 (-6.9,-0.5)	0 (-1.6,0)
**2027**	-10.4 (-20.2,-3.9)	-0.7 (-1.9,0)	-0.9 (-2.1,-0.1)	-1.3 (-11.5,-0.1)	-3.1 (-7.9,-0.5)	-1.2 (-6.7,-0.5)	-0.4 (-1.4,0)
**2028**	-16.1 (-23.9,-6.1)	-1.3 (-2.3,-0.3)	-1.1 (-2.1,-0.5)	-7.6 (-12,-0.4)	-5.5 (-8.9,-1.5)	-5.2 (-7.2,-0.7)	-0.6 (-2.1,0)
**2029**	-19.6 (-24.5,-9.8)	-1.5 (-2.7,-0.6)	-1.1 (-2,-0.5)	-7.6 (-11.6,-0.7)	-5.9 (-9.2,-2.7)	-5.7 (-7.7,-0.6)	-0.7 (-1.6,0)
**2030**	-19.9 (-27.6,-13.2)	-1.7 (-8.9,-0.9)	-1.1 (-2,-0.4)	-7.4 (-10.9,-1.1)	-5.8 (-8.9,-3.1)	-5.9 (-7.8,-1.4)	-0.6 (-1.4,-0.1)
**2031**	-19.8 (-27.3,-15)	-2.2 (-8.9,-1)	-1.1 (-2.3,-0.4)	-6.8 (-10.7,-3.6)	-5.4 (-8,-3.1)	-5.9 (-7.8,-4.2)	-0.5 (-1.4,-0.1)
**2032**	-22.3 (-28.8,-16.2)	-5 (-11.5,-1.4)	-1.2 (-1.9,-0.3)	-6.7 (-10.1,-4.1)	-5.1 (-7.2,-2.8)	-6.2 (-8.1,-4.4)	-0.4 (-1.4,0)
**2033**	-24.1 (-30.3,-18.9)	-7.3 (-12.6,-3.7)	-1.1 (-2.2,-0.4)	-6 (-9.6,-3.6)	-4.2 (-6.6,-2.2)	-6.2 (-8,-4.9)	-0.4 (-1.3,0.1)
**2034**	-22.6 (-28.7,-18.3)	-6.5 (-12.5,-3.5)	-1.1 (-2.1,-0.4)	-5.5 (-8.3,-3.5)	-3.7 (-6.4,-2)	-6.4 (-8.2,-4.8)	-0.3 (-1.3,0)
**2035**	-21.8 (-27.4,-16.8)	-6.6 (-10.7,-2.8)	-1 (-1.9,-0.3)	-4.9 (-7.6,-2.9)	-3.5 (-5.4,-1.4)	-6.6 (-8.7,-5.2)	-0.3 (-1.2,0.1)
**2036**	-21.1 (-26.9,-16.4)	-6.4 (-11.4,-3.3)	-0.9 (-2.1,-0.2)	-4.4 (-6.9,-2.6)	-3 (-5.3,-1.1)	-6.9 (-9.1,-5.2)	-0.3 (-1.1,0)
**2037**	-19.8 (-25.4,-15.9)	-6 (-10.1,-3.3)	-0.8 (-1.8,0)	-3.8 (-7,-2.2)	-2.4 (-4.7,-0.7)	-7 (-9.4,-5)	-0.2 (-0.9,0.2)
**2038**	-19.5 (-23.8,-14.7)	-5.8 (-10.3,-2.5)	-0.7 (-1.8,0.2)	-3.5 (-6,-1.6)	-2.1 (-5,-0.3)	-7.3 (-8.9,-5.6)	-0.2 (-0.8,0.1)
**2039**	-18.9 (-23.9,-13.9)	-5.7 (-10.4,-3.1)	-0.6 (-1.6,0.2)	-3.1 (-5.9,-1.1)	-1.8 (-4.2,0)	-7.4 (-9,-5.6)	-0.1 (-0.7,0.2)
**2040**	-17.9 (-23,-13.4)	-5.3 (-9.4,-2.4)	-0.6 (-1.5,0.2)	-3.2 (-5.2,-0.7)	-1.5 (-3.3,-0.1)	-7.5 (-9.1,-5.7)	-0.2 (-0.8,0.2)
**2041**	-17.1 (-22.2,-13.2)	-4.8 (-9.4,-2.3)	-0.4 (-1.5,0.3)	-2.8 (-4.5,-1.1)	-1.2 (-3.4,0.8)	-7.4 (-9.2,-5.2)	-0.2 (-0.7,0.2)
**2042**	-16.6 (-22.3,-12.7)	-5.4 (-9.2,-1.7)	-0.5 (-1.6,0.4)	-2.8 (-4.9,-1.2)	-1.1 (-3.1,0.2)	-7.2 (-9.3,-5.7)	-0.2 (-0.7,0.4)
**2043**	-16.3 (-20.2,-12.1)	-5.2 (-8.1,-2)	-0.5 (-1.6,0.5)	-2.6 (-4.4,-0.6)	-0.8 (-2.3,1)	-7.1 (-9.2,-5.1)	-0.1 (-0.6,0.2)

BMI = Body mass index; SBP = Systolic blood pressure

Table 4-7- Case-years prevented or postponed (million case-years) by scenario and year under a 10% improvement in risk factors compared to a base-case scenario of continuing trends

Year	All	BMI	BMI sensitivity	Fruit & veg	Physical activity	SBP	Smoking	Total cholesterol
**2023**	0 (0,0)	0 (0,0)	0 (0,0)	0 (0,0)	0 (0,0)	0 (0,0)	0 (0,0)	0 (0,0)
**2024**	0 (0,0)	0 (0,0)	0 (0,0)	0 (0,0)	0 (0,0)	0 (0,0)	0 (0,0)	0 (0,0)
**2025**	0 (0,0)	0 (0,0)	0 (0,0)	0 (0,0)	0 (0,0)	0 (0,0)	0 (0,0)	0 (0,0)
**2026**	0 (0,0)	0 (0,0)	0 (0,0)	0 (0,0)	0 (0,0)	0 (0,0)	0 (0,0)	0 (0,0)
**2027**	0 (0,0.1)	0 (0,0)	0 (0,0)	0 (0,0)	0 (0,0)	0 (0,0)	0 (0,0)	0 (0,0)
**2028**	0.1 (0,0.1)	0 (0,0)	0 (0,0.1)	0 (0,0)	0 (0,0)	0 (0,0)	0 (0,0)	0 (0,0)
**2029**	0.1 (0,0.2)	0 (0,0)	0 (0,0.1)	0 (0,0)	0 (0,0)	0 (0,0.1)	0 (0,0)	0 (0,0)
**2030**	0.1 (0.1,0.2)	0 (0,0)	0.1 (0,0.1)	0 (0,0)	0 (0,0)	0 (0,0.1)	0 (0,0)	0 (0,0)
**2031**	0.2 (0.1,0.3)	0 (0,0.1)	0.1 (0.1,0.2)	0 (0,0)	0 (0,0)	0 (0,0.1)	0 (0,0)	0 (0,0)
**2032**	0.3 (0.1,0.4)	0 (0,0.1)	0.2 (0.1,0.3)	0 (0,0)	0 (0,0)	0.1 (0,0.1)	0 (0,0)	0 (0,0)
**2033**	0.3 (0.2,0.5)	0.1 (0,0.1)	0.2 (0.1,0.4)	0 (0,0)	0 (0,0)	0.1 (0,0.1)	0 (0,0)	0 (0,0)
**2034**	0.4 (0.2,0.6)	0.1 (0.1,0.2)	0.3 (0.1,0.4)	0 (0,0)	0 (0,0)	0.1 (0,0.2)	0 (0,0)	0 (0,0)
**2035**	0.4 (0.2,0.6)	0.2 (0.1,0.3)	0.3 (0.2,0.5)	0 (0,0)	0 (0,0)	0.1 (0,0.2)	0 (0,0)	0 (0,0)
**2036**	0.5 (0.3,0.7)	0.2 (0.1,0.4)	0.4 (0.2,0.6)	0 (0,0)	0 (0,0)	0.1 (0,0.2)	0 (-0.1,0)	0 (0,0)
**2037**	0.6 (0.3,0.8)	0.3 (0.1,0.5)	0.5 (0.3,0.7)	0 (0,0)	0 (0,0)	0.1 (0,0.2)	0 (-0.1,0)	0 (0,0)
**2038**	0.6 (0.3,1)	0.4 (0.2,0.6)	0.6 (0.3,0.8)	0 (0,0)	0 (-0.1,0)	0.1 (0,0.2)	0 (-0.1,0)	0 (0,0.1)
**2039**	0.7 (0.3,1)	0.4 (0.2,0.7)	0.6 (0.4,0.9)	0 (0,0)	0 (-0.1,0)	0.1 (0,0.2)	-0.1 (-0.1,0)	0 (0,0.1)
**2040**	0.7 (0.4,1.1)	0.5 (0.3,0.8)	0.7 (0.4,1.1)	0 (0,0)	0 (-0.1,0)	0.1 (0,0.3)	-0.1 (-0.2,0)	0 (0,0.1)
**2041**	0.8 (0.4,1.2)	0.6 (0.3,0.9)	0.8 (0.5,1.2)	0 (0,0)	0 (-0.1,0)	0.1 (0,0.3)	-0.1 (-0.2,0)	0 (0,0.1)
**2042**	0.8 (0.4,1.3)	0.7 (0.4,1.1)	0.9 (0.5,1.3)	0 (0,0)	0 (-0.1,0)	0.1 (0,0.3)	-0.1 (-0.2,-0.1)	0 (0,0.1)
**2043**	0.9 (0.4,1.4)	0.8 (0.4,1.2)	0.9 (0.6,1.4)	0 (0,0)	0 (-0.1,0)	0.1 (0,0.3)	-0.2 (-0.3,-0.1)	0 (0,0.1)

BMI = Body mass index; SBP = Systolic blood pressure

Table 4-8 - Case-years prevented or postponed (million case-years) by scenario and year under theoretical minimum risk factor exposure levels compared to a base-case scenario of continuing trends

Year	All	BMI	Fruit & veg	Physical activity	SBP	Smoking	Total cholesterol
**2023**	0 (0,0)	0 (0,0)	0 (0,0)	0 (0,0)	0 (0,0)	0 (0,0)	0 (0,0)
**2024**	0 (0,0)	0 (0,0)	0 (0,0)	0 (0,0)	0 (0,0)	0 (0,0)	0 (0,0)
**2025**	0 (0,0.1)	0 (0,0.1)	0 (0,0)	0 (0,0)	0 (0,0.1)	0 (0,0)	0 (0,0)
**2026**	0.1 (0.1,0.3)	0 (0,0.1)	0 (0,0)	0 (0,0.1)	0 (0,0.1)	0 (0,0)	0 (0,0)
**2027**	0.2 (0.1,0.5)	0.1 (0,0.2)	0 (0,0)	0 (0,0.1)	0.1 (0,0.2)	0 (0,0.1)	0 (0,0)
**2028**	0.4 (0.2,0.8)	0.2 (0,0.4)	0 (0,0)	0 (-0.1,0.1)	0.1 (0,0.3)	0.1 (0,0.1)	0 (0,0)
**2029**	0.6 (0.3,1.1)	0.3 (0.1,0.6)	0 (0,0.1)	0 (-0.2,0.2)	0.2 (0,0.5)	0.1 (0,0.1)	0 (0,0.1)
**2030**	0.9 (0.4,1.5)	0.6 (0.2,0.9)	0 (0,0.1)	0 (-0.3,0.2)	0.3 (0,0.6)	0.1 (-0.1,0.1)	0 (0,0.1)
**2031**	1.2 (0.6,1.9)	0.8 (0.4,1.3)	0.1 (0,0.1)	0 (-0.4,0.3)	0.4 (0.1,0.8)	0.1 (-0.1,0.2)	0 (0,0.1)
**2032**	1.5 (0.7,2.3)	1.1 (0.6,1.8)	0.1 (0,0.1)	-0.1 (-0.6,0.3)	0.5 (0.1,1)	0 (-0.2,0.2)	0 (0,0.1)
**2033**	1.8 (0.9,2.8)	1.5 (0.8,2.3)	0.1 (0,0.2)	-0.1 (-0.8,0.3)	0.7 (0.1,1.2)	0 (-0.2,0.2)	0.1 (0,0.1)
**2034**	2.2 (1,3.3)	1.9 (1,2.8)	0.1 (0.1,0.2)	-0.2 (-0.9,0.3)	0.8 (0.2,1.3)	0 (-0.3,0.2)	0.1 (0,0.2)
**2035**	2.5 (1.2,3.7)	2.4 (1.2,3.4)	0.1 (0.1,0.2)	-0.3 (-1.1,0.3)	0.8 (0.2,1.5)	-0.1 (-0.3,0.2)	0.1 (0,0.2)
**2036**	2.9 (1.4,4.2)	2.8 (1.5,4.1)	0.1 (0.1,0.3)	-0.4 (-1.3,0.3)	1 (0.2,1.7)	-0.1 (-0.4,0.2)	0.1 (0,0.2)
**2037**	3.2 (1.5,4.7)	3.3 (1.9,4.8)	0.2 (0.1,0.3)	-0.5 (-1.5,0.3)	1.1 (0.2,1.9)	-0.2 (-0.5,0.2)	0.1 (0,0.3)
**2038**	3.7 (1.7,5.3)	3.8 (2.2,5.4)	0.2 (0.1,0.3)	-0.6 (-1.6,0.3)	1.2 (0.3,2.1)	-0.3 (-0.6,0.1)	0.1 (0,0.3)
**2039**	4 (1.9,6)	4.4 (2.6,6.2)	0.2 (0.1,0.3)	-0.7 (-1.8,0.3)	1.3 (0.3,2.3)	-0.3 (-0.7,0.1)	0.2 (0.1,0.4)
**2040**	4.3 (2.1,6.6)	4.9 (3,7)	0.2 (0.1,0.4)	-0.8 (-2,0.3)	1.4 (0.3,2.5)	-0.5 (-0.8,0)	0.2 (0.1,0.4)
**2041**	4.7 (2.3,7.2)	5.5 (3.5,7.8)	0.2 (0.1,0.4)	-0.9 (-2.2,0.3)	1.5 (0.4,2.8)	-0.6 (-1,-0.1)	0.2 (0.1,0.4)
**2042**	5.2 (2.4,7.9)	6.1 (3.9,8.6)	0.3 (0.1,0.4)	-1 (-2.4,0.2)	1.6 (0.4,3)	-0.7 (-1.1,-0.2)	0.2 (0.1,0.5)
**2043**	5.5 (2.5,8.6)	6.7 (4.4,9.5)	0.3 (0.1,0.5)	-1.1 (-2.6,0.1)	1.7 (0.4,3.3)	-0.9 (-1.3,-0.3)	0.2 (0.1,0.5)

BMI = Body mass index; SBP = Systolic blood pressure

Table 4-9 - Absolute difference in numbers living without major illness (millions) by scenario and year under a 10% improvement in risk factors compared to a base-case scenario of continuing trends

Year	All	BMI	BMI sensitivity	Fruit & veg	Physical activity	SBP	Smoking	Total cholesterol
**2023**	0 (0,0)	0 (0,0)	0 (0,0)	0 (0,0)	0 (0,0)	0 (0,0)	0 (0,0)	0 (0,0)
**2024**	0 (0,0)	0 (0,0)	0 (0,0)	0 (0,0)	0 (0,0)	0 (0,0)	0 (0,0)	0 (0,0)
**2025**	0 (0,0)	0 (0,0)	0 (0,0)	0 (0,0)	0 (0,0)	0 (0,0)	0 (0,0)	0 (0,0)
**2026**	0 (0,0)	0 (0,0)	0 (0,0)	0 (0,0)	0 (0,0)	0 (0,0)	0 (0,0)	0 (0,0)
**2027**	0 (0,0)	0 (0,0)	0 (0,0)	0 (0,0)	0 (0,0)	0 (0,0)	0 (0,0)	0 (0,0)
**2028**	0 (0,0.1)	0 (0,0)	0 (0,0)	0 (0,0)	0 (0,0)	0 (0,0)	0 (0,0)	0 (0,0)
**2029**	0.1 (0,0.1)	0 (0,0.1)	0 (0,0)	0 (0,0)	0 (0,0)	0 (0,0)	0 (0,0)	0 (0,0)
**2030**	0.1 (0.1,0.1)	0.1 (0,0.1)	0 (0,0.1)	0 (0,0)	0 (0,0)	0 (0,0)	0 (0,0)	0 (0,0)
**2031**	0.1 (0.1,0.1)	0.1 (0,0.1)	0 (0,0.1)	0 (0,0)	0 (0,0)	0 (0,0.1)	0 (0,0)	0 (0,0)
**2032**	0.1 (0.1,0.2)	0.1 (0.1,0.1)	0.1 (0,0.1)	0 (0,0)	0 (0,0)	0 (0,0.1)	0 (0,0)	0 (0,0)
**2033**	0.1 (0.1,0.2)	0.1 (0.1,0.1)	0.1 (0,0.1)	0 (0,0)	0 (0,0)	0 (0,0.1)	0 (0,0)	0 (0,0)
**2034**	0.2 (0.1,0.2)	0.1 (0.1,0.2)	0.1 (0,0.1)	0 (0,0)	0 (0,0)	0 (0,0.1)	0 (0,0)	0 (0,0)
**2035**	0.2 (0.1,0.2)	0.1 (0.1,0.2)	0.1 (0.1,0.1)	0 (0,0)	0 (0,0)	0 (0,0.1)	0 (0,0)	0 (0,0)
**2036**	0.2 (0.1,0.2)	0.1 (0.1,0.2)	0.1 (0.1,0.1)	0 (0,0)	0 (0,0)	0 (0,0.1)	0 (0,0)	0 (0,0)
**2037**	0.2 (0.2,0.3)	0.1 (0.1,0.2)	0.1 (0.1,0.1)	0 (0,0)	0 (0,0)	0 (0,0.1)	0 (0,0)	0 (0,0)
**2038**	0.2 (0.2,0.3)	0.2 (0.1,0.2)	0.1 (0.1,0.2)	0 (0,0)	0 (0,0)	0.1 (0,0.1)	0 (0,0)	0 (0,0)
**2039**	0.2 (0.2,0.3)	0.2 (0.1,0.2)	0.1 (0.1,0.2)	0 (0,0)	0 (0,0)	0.1 (0,0.1)	0 (0,0.1)	0 (0,0)
**2040**	0.2 (0.2,0.3)	0.2 (0.1,0.2)	0.1 (0.1,0.2)	0 (0,0)	0 (0,0)	0.1 (0,0.1)	0 (0,0.1)	0 (0,0)
**2041**	0.3 (0.2,0.3)	0.2 (0.1,0.3)	0.1 (0.1,0.2)	0 (0,0)	0 (0,0)	0.1 (0,0.1)	0 (0,0.1)	0 (0,0)
**2042**	0.3 (0.2,0.3)	0.2 (0.1,0.3)	0.1 (0.1,0.2)	0 (0,0)	0 (0,0)	0.1 (0,0.1)	0 (0,0.1)	0 (0,0)
**2043**	0.3 (0.2,0.3)	0.2 (0.1,0.3)	0.1 (0.1,0.2)	0 (0,0)	0 (0,0)	0.1 (0,0.1)	0.1 (0,0.1)	0 (0,0)

BMI = Body mass index; SBP = Systolic blood pressure

Table 4-10 - Absolute difference in numbers living without major illness (millions) by scenario and year under theoretical minimum risk factor exposure levels compared to a base-case scenario of continuing trends

Year	All	BMI	Fruit & veg	Physical activity	SBP	Smoking	Total cholesterol
**2023**	0 (0,0)	0 (0,0)	0 (0,0)	0 (0,0)	0 (0,0)	0 (0,0)	0 (0,0)
**2024**	0 (0,0)	0 (0,0)	0 (0,0)	0 (0,0)	0 (0,0)	0 (0,0)	0 (0,0)
**2025**	0 (0,0.1)	0 (0,0)	0 (0,0)	0 (0,0)	0 (0,0)	0 (0,0)	0 (0,0)
**2026**	0.1 (0,0.2)	0 (0,0.1)	0 (0,0)	0 (0,0)	0 (0,0.1)	0 (0,0)	0 (0,0)
**2027**	0.2 (0.1,0.3)	0.1 (0,0.1)	0 (0,0)	0 (0,0)	0.1 (0,0.1)	0 (0,0.1)	0 (0,0)
**2028**	0.3 (0.2,0.4)	0.1 (0,0.2)	0 (0,0)	0 (0,0.1)	0.1 (0,0.2)	0 (0,0.1)	0 (0,0)
**2029**	0.4 (0.3,0.5)	0.2 (0.1,0.3)	0 (0,0)	0.1 (0,0.1)	0.1 (0.1,0.2)	0.1 (0,0.1)	0 (0,0)
**2030**	0.5 (0.4,0.7)	0.2 (0.1,0.4)	0 (0,0.1)	0.1 (0,0.1)	0.2 (0.1,0.3)	0.1 (0.1,0.1)	0 (0,0)
**2031**	0.7 (0.5,0.8)	0.3 (0.2,0.4)	0 (0,0.1)	0.1 (0.1,0.1)	0.2 (0.1,0.3)	0.1 (0.1,0.1)	0 (0,0.1)
**2032**	0.8 (0.7,1)	0.4 (0.2,0.5)	0 (0,0.1)	0.1 (0.1,0.2)	0.2 (0.2,0.4)	0.1 (0.1,0.2)	0 (0,0.1)
**2033**	1 (0.8,1.1)	0.4 (0.3,0.6)	0.1 (0,0.1)	0.1 (0.1,0.2)	0.3 (0.2,0.4)	0.2 (0.1,0.2)	0 (0,0.1)
**2034**	1.1 (0.9,1.3)	0.5 (0.3,0.7)	0.1 (0,0.1)	0.2 (0.1,0.2)	0.3 (0.2,0.5)	0.2 (0.1,0.2)	0 (0,0.1)
**2035**	1.2 (1,1.5)	0.6 (0.4,0.8)	0.1 (0,0.1)	0.2 (0.2,0.2)	0.3 (0.2,0.5)	0.2 (0.2,0.2)	0 (0,0.1)
**2036**	1.4 (1.1,1.6)	0.7 (0.4,0.9)	0.1 (0.1,0.1)	0.2 (0.2,0.3)	0.3 (0.2,0.6)	0.2 (0.2,0.3)	0 (0,0.1)
**2037**	1.5 (1.2,1.7)	0.7 (0.5,0.9)	0.1 (0.1,0.1)	0.2 (0.2,0.3)	0.4 (0.2,0.6)	0.3 (0.2,0.3)	0 (0,0.1)
**2038**	1.6 (1.3,1.9)	0.8 (0.5,1)	0.1 (0.1,0.1)	0.3 (0.2,0.3)	0.4 (0.2,0.6)	0.3 (0.2,0.3)	0 (0,0.1)
**2039**	1.7 (1.4,2)	0.8 (0.6,1.1)	0.1 (0.1,0.1)	0.3 (0.2,0.3)	0.4 (0.2,0.6)	0.3 (0.3,0.3)	0 (0,0.1)
**2040**	1.8 (1.5,2.1)	0.9 (0.6,1.2)	0.1 (0.1,0.1)	0.3 (0.2,0.3)	0.4 (0.2,0.7)	0.3 (0.3,0.4)	0 (0,0.1)
**2041**	1.9 (1.6,2.3)	1 (0.6,1.3)	0.1 (0.1,0.1)	0.3 (0.3,0.4)	0.4 (0.2,0.7)	0.4 (0.3,0.4)	0 (0,0.1)
**2042**	2 (1.7,2.4)	1 (0.7,1.3)	0.1 (0.1,0.2)	0.3 (0.3,0.4)	0.4 (0.2,0.7)	0.4 (0.3,0.4)	0 (0,0.1)
**2043**	2.1 (1.8,2.5)	1.1 (0.7,1.4)	0.1 (0.1,0.2)	0.3 (0.3,0.4)	0.4 (0.2,0.7)	0.4 (0.4,0.5)	0.1 (0,0.1)

BMI = Body mass index; SBP = Systolic blood pressure

5. Absolute differences compared to base-case scenario– results by age-group

Figure 5-1 – Absolute change in projected prevalence of major illness (Cambridge multimorbidity score > 1.5) under a scenario of 10% improvement in risk factor exposures by age-group compared to a base-case scenario of continuing trends

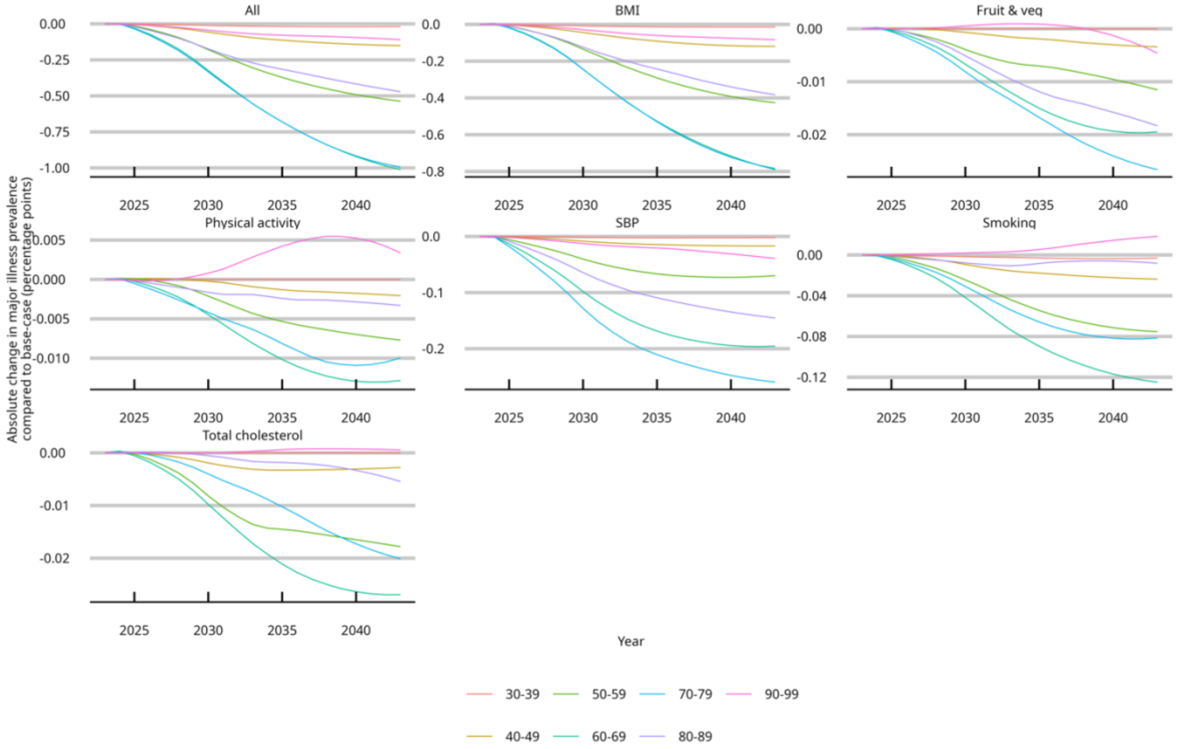

All curves are smoothed; *Major illness is defined as a Cambridge multimorbidity score > 1.5; BMI = Body mass index; SBP = Systolic blood pressure

6. Absolute differences compared to base-case scenario– results by birth cohort

Figure 6-1 - Absolute change in the projected prevalence rate of major illness for three cohorts under a scenario of 10% improvement in risk factor exposures

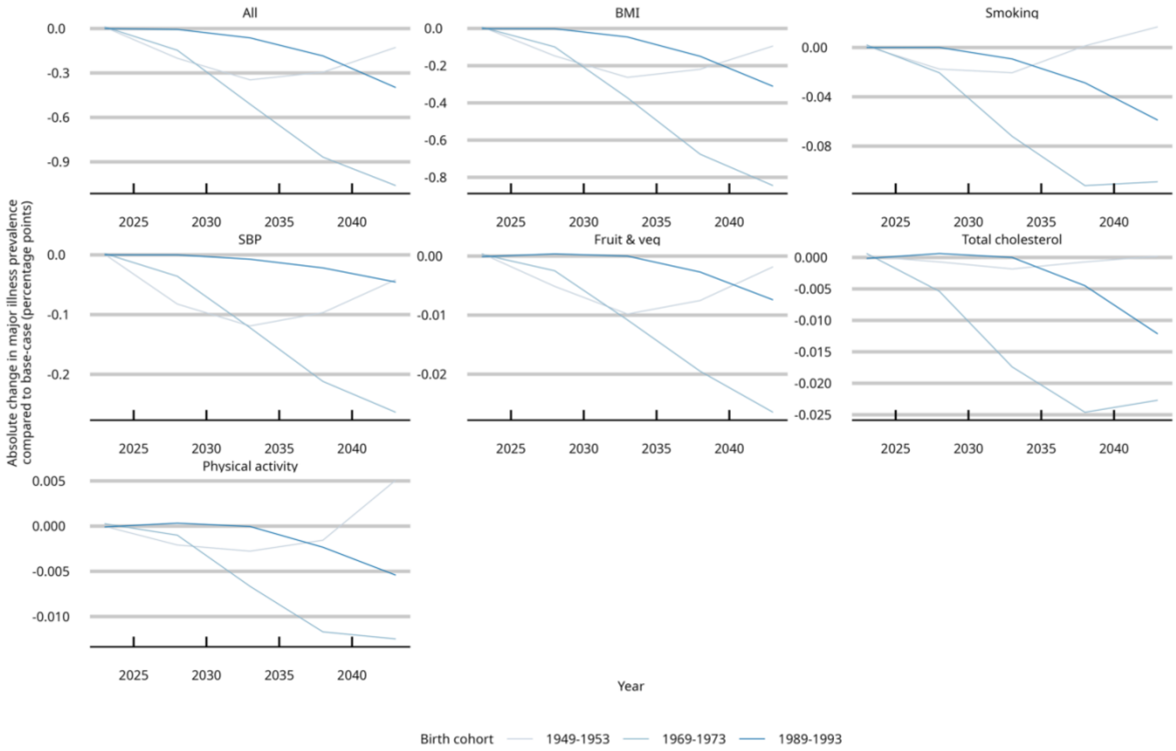

All curves are smoothed; Major illness is defined as a Cambridge Multimorbidity Score > 1.5; SBP = systolic blood pressure; BMI = body mass index

7. Relative change compared to base-case scenario

10% improvement in risk factor exposure levels

Figure 7-1 – Relative change in projected incidence of major illness, prevalence of major illness, and all-cause mortality under a scenario of 10% improvement in risk factor exposure levels compared to a base-case scenario of continuing trends*

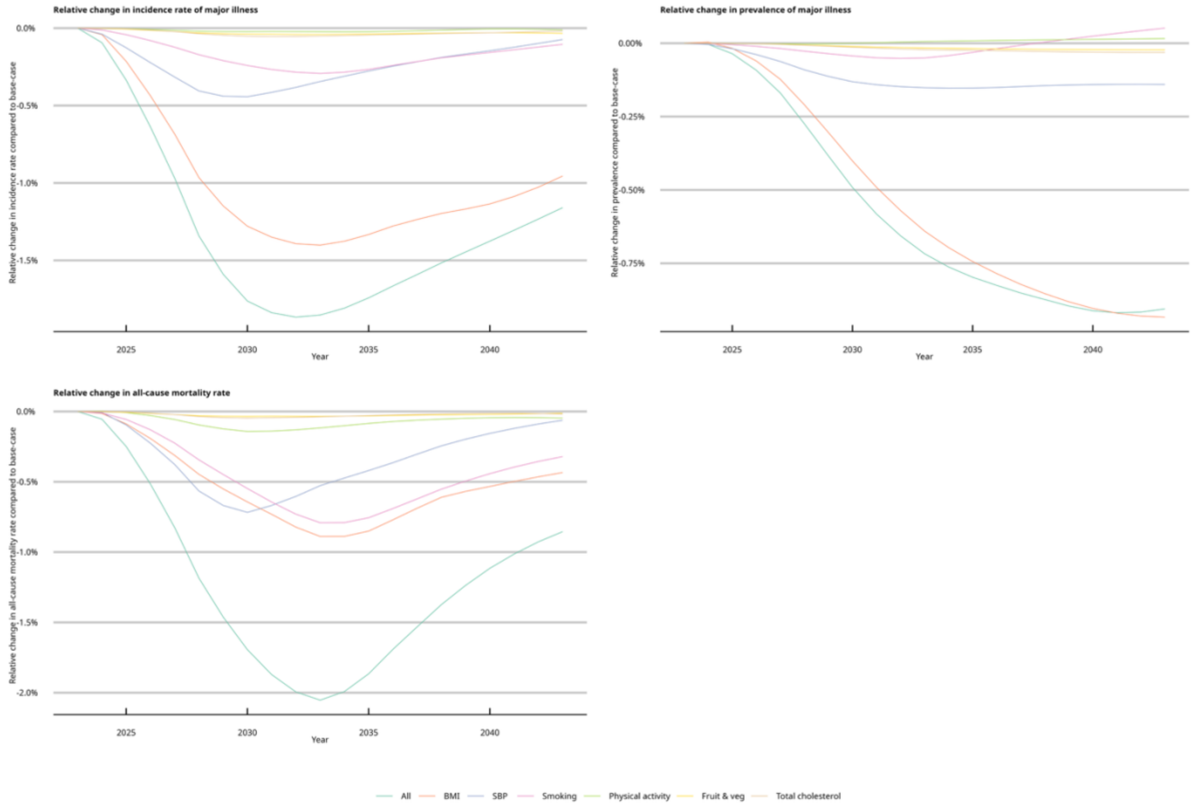

All curves are smoothed; *Major illness is defined as a Cambridge multimorbidity score > 1.5; BMI = Body mass index; SBP = Systolic blood pressure

Theoretical minimum risk exposure levels

Figure 7-2 – Relative change in projected incidence of major illness, prevalence of major illness, and all-cause mortality under a scenario of theoretical minimum risk exposure levels compared to a base-case scenario of continuing trends*

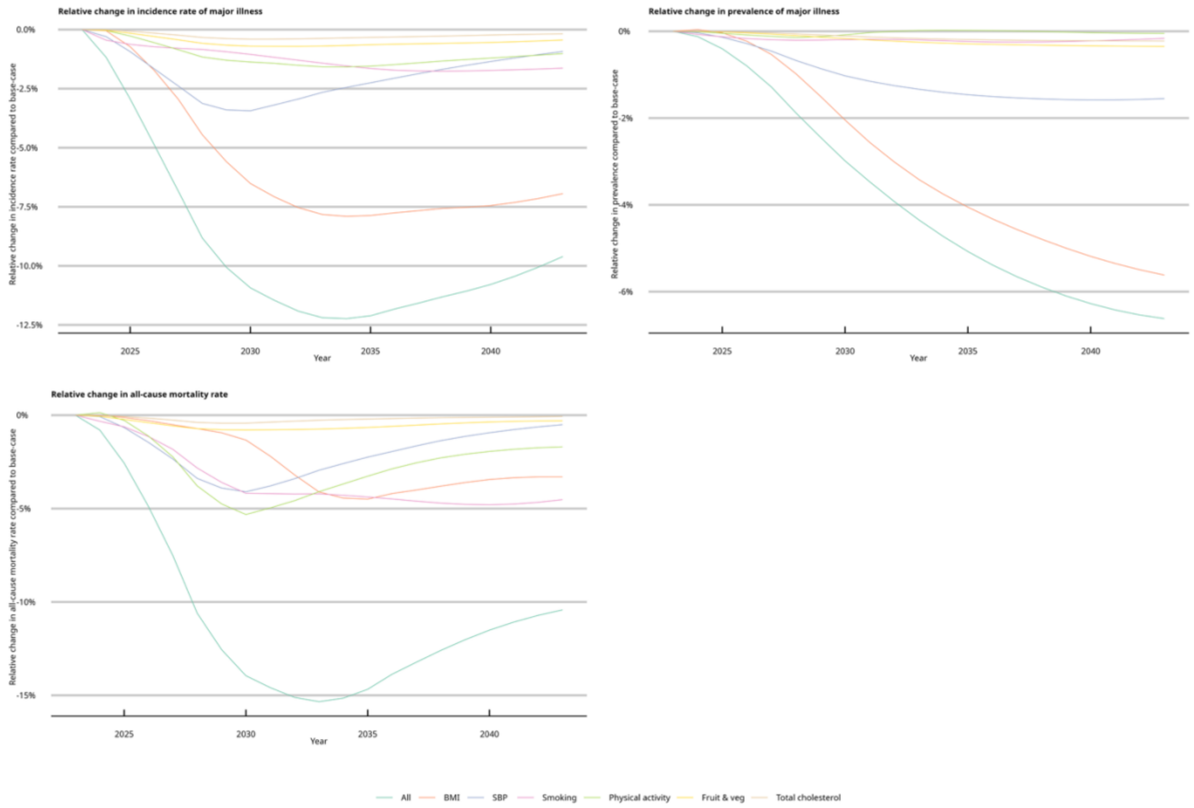

All curves are smoothed; *Major illness is defined as a Cambridge multimorbidity score > 1.5; BMI = Body mass index; SBP = Systolic blood pressure

Relative change compared to base-case scenario - tables

Table 7-1- Relative change (%) in major illness prevalence by scenario and year under a 10% improvement in risk factors compared to a base-case scenario of continuing trends

Year	All (%)	BMI (%)	BMI sensitivity (%)	Fruit & veg (%)	Physical activity (%)	SBP (%)	Smoking (%)	Total cholesterol (%)
**2023**	0 (0,0)	0 (0,0)	0 (0,0)	0 (0,0)	0 (0,0)	0 (0,0)	0 (0,0)	0 (0,0)
**2024**	0 (0,0)	0 (0,0)	0 (0,0)	0 (0,0)	0 (0,0)	0 (0,0)	0 (0,0)	0 (0,0)
**2025**	0 (-0.1,0)	0 (-0.1,0)	0 (-0.1,0)	0 (0,0)	0 (0,0)	0 (-0.1,0)	0 (0,0)	0 (0,0)
**2026**	-0.1 (-0.2,0)	0 (-0.2,0)	0 (-0.1,0)	0 (0,0)	0 (0,0)	0 (-0.1,0)	0 (0,0)	0 (0,0)
**2027**	-0.2 (-0.3,-0.1)	-0.1 (-0.3,0)	-0.1 (-0.2,0)	0 (0,0)	0 (0,0)	-0.1 (-0.1,0)	0 (-0.1,0)	0 (0,0)
**2028**	-0.3 (-0.5,-0.1)	-0.2 (-0.4,-0.1)	-0.1 (-0.3,0)	0 (0,0)	0 (0,0)	-0.1 (-0.2,0)	0 (-0.1,0)	0 (0,0)
**2029**	-0.4 (-0.6,-0.2)	-0.3 (-0.5,-0.1)	-0.2 (-0.4,-0.1)	0 (0,0)	0 (0,0)	-0.1 (-0.2,0)	0 (-0.1,0)	0 (0,0)
**2030**	-0.5 (-0.7,-0.3)	-0.4 (-0.7,-0.2)	-0.3 (-0.5,-0.1)	0 (0,0)	0 (0,0)	-0.1 (-0.2,0)	0 (-0.1,0)	0 (0,0)
**2031**	-0.6 (-0.8,-0.4)	-0.5 (-0.8,-0.3)	-0.4 (-0.6,-0.2)	0 (0,0)	0 (0,0)	-0.1 (-0.2,0)	0 (-0.1,0)	0 (-0.1,0)
**2032**	-0.7 (-0.9,-0.4)	-0.6 (-0.9,-0.4)	-0.4 (-0.7,-0.2)	0 (0,0)	0 (0,0)	-0.1 (-0.3,-0.1)	-0.1 (-0.1,0)	0 (-0.1,0)
**2033**	-0.7 (-1,-0.5)	-0.6 (-0.9,-0.4)	-0.5 (-0.7,-0.3)	0 (0,0)	0 (0,0)	-0.2 (-0.3,-0.1)	-0.1 (-0.1,0)	0 (-0.1,0)
**2034**	-0.8 (-1.1,-0.5)	-0.7 (-1,-0.4)	-0.5 (-0.8,-0.3)	0 (0,0)	0 (0,0)	-0.2 (-0.3,-0.1)	0 (-0.1,0)	0 (-0.1,0)
**2035**	-0.8 (-1.1,-0.5)	-0.7 (-1.1,-0.5)	-0.6 (-0.8,-0.4)	0 (0,0)	0 (0,0)	-0.2 (-0.3,-0.1)	0 (-0.1,0)	0 (-0.1,0)
**2036**	-0.8 (-1.1,-0.6)	-0.8 (-1.2,-0.5)	-0.6 (-0.9,-0.4)	0 (0,0)	0 (0,0)	-0.1 (-0.3,-0.1)	0 (-0.1,0)	0 (-0.1,0)
**2037**	-0.8 (-1.2,-0.6)	-0.8 (-1.2,-0.5)	-0.7 (-0.9,-0.4)	0 (0,0)	0 (0,0)	-0.1 (-0.3,-0.1)	0 (-0.1,0.1)	0 (-0.1,0)
**2038**	-0.9 (-1.2,-0.5)	-0.9 (-1.2,-0.5)	-0.7 (-1,-0.5)	0 (0,0)	0 (0,0)	-0.1 (-0.3,-0.1)	0 (-0.1,0.1)	0 (-0.1,0)
**2039**	-0.9 (-1.2,-0.6)	-0.9 (-1.3,-0.5)	-0.7 (-1,-0.5)	0 (0,0)	0 (0,0)	-0.1 (-0.3,-0.1)	0 (0,0.1)	0 (-0.1,0)
**2040**	-0.9 (-1.2,-0.6)	-0.9 (-1.3,-0.6)	-0.7 (-1.1,-0.5)	0 (0,0)	0 (0,0)	-0.1 (-0.3,0)	0 (0,0.1)	0 (-0.1,0)
**2041**	-0.9 (-1.2,-0.6)	-0.9 (-1.3,-0.6)	-0.7 (-1.1,-0.5)	0 (0,0)	0 (0,0)	-0.1 (-0.3,-0.1)	0 (0,0.1)	0 (-0.1,0)
**2042**	-0.9 (-1.3,-0.5)	-0.9 (-1.3,-0.6)	-0.8 (-1.1,-0.5)	0 (0,0)	0 (0,0)	-0.1 (-0.3,0)	0 (0,0.1)	0 (-0.1,0)
**2043**	-0.9 (-1.3,-0.5)	-0.9 (-1.4,-0.5)	-0.8 (-1.2,-0.5)	0 (0,0)	0 (0,0)	-0.1 (-0.3,0)	0.1 (0,0.1)	0 (-0.1,0)

BMI = Body mass index; SBP = Systolic blood pressure

Table 7-2 - Relative change (%) in major illness prevalence by scenario and year under theoretical minimum risk factor exposure levels compared to a base-case scenario of continuing trends

Year	All (%)	BMI (%)	Fruit & veg (%)	Physical activity (%)	SBP (%)	Smoking (%)	Total cholesterol (%)
**2023**	0 (0,0)	0 (0,0)	0 (0,0)	0 (0,0)	0 (0,0)	0 (0,0)	0 (0,0)
**2024**	-0.1 (-0.4,-0.1)	0 (-0.2,0)	0 (0,0)	0 (-0.1,0)	0 (-0.2,0)	-0.1 (-0.1,-0.1)	0 (0,0)
**2025**	-0.3 (-0.8,-0.1)	0 (-0.4,0)	0 (0,0)	0 (-0.2,0)	-0.1 (-0.4,0)	-0.1 (-0.2,-0.1)	0 (0,0)
**2026**	-0.7 (-1.5,-0.4)	-0.2 (-0.8,0)	0 (-0.1,0)	-0.1 (-0.3,0)	-0.3 (-0.7,0)	-0.2 (-0.2,-0.1)	0 (-0.1,0)
**2027**	-1.3 (-2.2,-0.5)	-0.5 (-1.2,0)	-0.1 (-0.1,0)	-0.1 (-0.4,0.2)	-0.4 (-0.9,0)	-0.2 (-0.3,-0.1)	0 (-0.1,0)
**2028**	-1.9 (-2.9,-1.1)	-1 (-1.7,-0.2)	-0.1 (-0.2,0)	-0.2 (-0.5,0.3)	-0.7 (-1.3,-0.2)	-0.2 (-0.3,0)	-0.1 (-0.2,0)
**2029**	-2.5 (-3.6,-1.5)	-1.5 (-2.4,-0.5)	-0.1 (-0.2,-0.1)	-0.1 (-0.6,0.4)	-0.9 (-1.6,-0.3)	-0.2 (-0.4,0)	-0.1 (-0.2,0)
**2030**	-3 (-4,-1.9)	-2.1 (-3,-1.1)	-0.2 (-0.3,-0.1)	-0.1 (-0.5,0.5)	-1 (-1.8,-0.4)	-0.2 (-0.4,0)	-0.1 (-0.2,0)
**2031**	-3.4 (-4.7,-2.3)	-2.6 (-3.7,-1.3)	-0.2 (-0.3,-0.1)	0 (-0.5,0.6)	-1.2 (-1.9,-0.5)	-0.2 (-0.5,0)	-0.1 (-0.3,0)
**2032**	-3.9 (-5.2,-2.7)	-3 (-4.3,-1.7)	-0.2 (-0.4,-0.1)	0 (-0.5,0.6)	-1.2 (-2.1,-0.6)	-0.2 (-0.5,0)	-0.2 (-0.3,0)
**2033**	-4.4 (-5.7,-3)	-3.4 (-4.7,-2)	-0.2 (-0.4,-0.2)	0 (-0.5,0.6)	-1.3 (-2.2,-0.6)	-0.2 (-0.5,-0.1)	-0.2 (-0.4,-0.1)
**2034**	-4.7 (-6.2,-3.3)	-3.7 (-5.1,-2.4)	-0.3 (-0.4,-0.2)	0 (-0.6,0.5)	-1.4 (-2.4,-0.6)	-0.2 (-0.4,-0.1)	-0.2 (-0.4,-0.1)
**2035**	-5.1 (-6.6,-3.4)	-4.1 (-5.5,-2.7)	-0.3 (-0.4,-0.2)	0 (-0.5,0.6)	-1.5 (-2.5,-0.6)	-0.2 (-0.4,-0.1)	-0.2 (-0.4,-0.1)
**2036**	-5.4 (-6.9,-3.7)	-4.3 (-5.9,-2.9)	-0.3 (-0.4,-0.2)	0 (-0.5,0.5)	-1.5 (-2.6,-0.6)	-0.3 (-0.5,0)	-0.2 (-0.4,-0.1)
**2037**	-5.7 (-7.3,-3.9)	-4.6 (-6.2,-3.1)	-0.3 (-0.4,-0.2)	0 (-0.5,0.5)	-1.5 (-2.7,-0.6)	-0.3 (-0.4,0)	-0.2 (-0.5,-0.1)
**2038**	-5.9 (-7.6,-4.1)	-4.8 (-6.6,-3.3)	-0.3 (-0.5,-0.2)	0 (-0.5,0.5)	-1.6 (-2.8,-0.6)	-0.2 (-0.4,0)	-0.2 (-0.5,-0.1)
**2039**	-6.1 (-7.9,-4.2)	-5 (-7,-3.4)	-0.3 (-0.5,-0.2)	0 (-0.5,0.6)	-1.6 (-2.8,-0.6)	-0.2 (-0.5,-0.1)	-0.2 (-0.5,-0.1)
**2040**	-6.3 (-8.2,-4.4)	-5.2 (-7.3,-3.5)	-0.3 (-0.5,-0.2)	0 (-0.6,0.5)	-1.6 (-2.9,-0.5)	-0.2 (-0.5,0)	-0.2 (-0.5,-0.1)
**2041**	-6.4 (-8.5,-4.5)	-5.4 (-7.6,-3.6)	-0.3 (-0.5,-0.2)	0 (-0.7,0.5)	-1.6 (-2.9,-0.5)	-0.2 (-0.4,0)	-0.2 (-0.5,-0.1)
**2042**	-6.6 (-8.8,-4.6)	-5.5 (-7.9,-3.8)	-0.3 (-0.5,-0.2)	-0.1 (-0.6,0.5)	-1.6 (-2.9,-0.5)	-0.2 (-0.4,0)	-0.2 (-0.5,-0.1)
**2043**	-6.6 (-9,-4.5)	-5.6 (-8.1,-3.9)	-0.3 (-0.5,-0.2)	0 (-0.6,0.5)	-1.6 (-3,-0.5)	-0.2 (-0.4,0.1)	-0.2 (-0.5,-0.1)

BMI = Body mass index; SBP = Systolic blood pressure

Table 7-3- Relative change (%) in major illness incidence rates by scenario and year under a 10% improvement in risk factors compared to a base-case scenario of continuing trends

Year	All	BMI	BMI sensitivity	Fruit & veg	Physical activity	SBP	Smoking	Total cholesterol
**2023**	0 (0,0)	0 (0,0)	0 (0,0)	0 (0,0)	0 (0,0)	0 (0,0)	0 (0,0)	0 (0,0)
**2024**	0 (-0.4,0)	0 (-0.4,0.1)	0 (-0.3,0)	0 (0,0)	0 (0,0)	0 (-0.2,0)	0 (-0.1,0.1)	0 (0,0)
**2025**	-0.2 (-0.7,0)	-0.1 (-0.6,0)	0 (-0.5,0)	0 (0,0)	0 (0,0)	-0.1 (-0.4,0)	0 (-0.1,0.1)	0 (-0.1,0)
**2026**	-0.5 (-1.2,-0.1)	-0.4 (-1.1,0)	-0.2 (-0.8,0)	0 (-0.1,0)	0 (-0.1,0)	-0.2 (-0.5,0)	-0.1 (-0.3,0)	0 (-0.1,0)
**2027**	-1 (-1.8,-0.4)	-0.7 (-1.3,-0.2)	-0.4 (-1,0)	0 (-0.1,0)	0 (-0.1,0)	-0.3 (-0.9,-0.1)	-0.1 (-0.4,0)	0 (-0.1,0)
**2028**	-1.4 (-2.2,-0.7)	-1 (-2,-0.4)	-0.7 (-1.4,-0.1)	0 (-0.1,0)	0 (-0.1,0)	-0.4 (-0.8,-0.2)	-0.2 (-0.4,0)	0 (-0.2,0)
**2029**	-1.6 (-2.3,-0.9)	-1.2 (-2,-0.6)	-0.8 (-1.4,-0.3)	0 (-0.1,0)	0 (-0.1,0)	-0.4 (-0.9,-0.2)	-0.2 (-0.4,0)	0 (-0.2,0)
**2030**	-1.8 (-2.4,-1.2)	-1.3 (-2,-0.7)	-1 (-1.5,-0.5)	0 (-0.1,0)	0 (-0.1,0)	-0.5 (-0.9,-0.1)	-0.2 (-0.5,-0.1)	-0.1 (-0.2,0)
**2031**	-1.8 (-2.6,-1.3)	-1.3 (-2.1,-0.7)	-1 (-1.5,-0.6)	0 (-0.1,0)	0 (-0.1,0)	-0.4 (-0.7,-0.1)	-0.3 (-0.5,-0.1)	0 (-0.2,0)
**2032**	-1.8 (-2.6,-1.2)	-1.4 (-2.2,-0.7)	-1.1 (-1.6,-0.5)	0 (-0.1,0)	0 (-0.1,0)	-0.4 (-0.7,-0.1)	-0.3 (-0.5,0)	-0.1 (-0.2,0)
**2033**	-1.9 (-2.6,-1.1)	-1.5 (-2.2,-0.6)	-1.1 (-1.8,-0.5)	0 (-0.1,0)	0 (-0.1,0)	-0.3 (-0.8,-0.1)	-0.3 (-0.5,-0.1)	-0.1 (-0.2,0)
**2034**	-1.8 (-2.5,-1.2)	-1.4 (-2.1,-0.6)	-1.1 (-1.7,-0.6)	0 (-0.1,0)	0 (-0.1,0)	-0.3 (-0.7,-0.1)	-0.3 (-0.5,-0.1)	0 (-0.2,0.1)
**2035**	-1.7 (-2.4,-1.1)	-1.3 (-2,-0.7)	-1.1 (-1.7,-0.7)	0 (-0.1,0)	0 (-0.1,0.1)	-0.3 (-0.6,0)	-0.3 (-0.5,-0.1)	0 (-0.2,0)
**2036**	-1.7 (-2.3,-0.9)	-1.2 (-2.1,-0.6)	-1 (-1.7,-0.6)	0 (-0.1,0)	0 (-0.1,0.1)	-0.2 (-0.6,0)	-0.2 (-0.5,0)	0 (-0.2,0.1)
**2037**	-1.6 (-2.2,-0.9)	-1.3 (-2,-0.6)	-1 (-1.6,-0.6)	0 (-0.1,0)	0 (-0.1,0.1)	-0.2 (-0.6,0.1)	-0.2 (-0.5,0.1)	0 (-0.1,0.1)
**2038**	-1.5 (-2.3,-0.9)	-1.2 (-2,-0.6)	-1 (-1.6,-0.5)	0 (-0.1,0)	0 (-0.1,0.1)	-0.2 (-0.6,0.1)	-0.2 (-0.4,0)	0 (-0.2,0)
**2039**	-1.4 (-2.2,-0.7)	-1.1 (-1.9,-0.5)	-1 (-1.7,-0.5)	0 (-0.1,0.1)	0 (-0.1,0.1)	-0.2 (-0.5,0.1)	-0.2 (-0.4,0.1)	0 (-0.2,0)
**2040**	-1.4 (-2.1,-0.5)	-1.1 (-1.9,-0.5)	-1 (-1.7,-0.5)	0 (-0.1,0.1)	0 (-0.1,0.1)	-0.1 (-0.5,0.1)	-0.2 (-0.4,0.1)	0 (-0.2,0.1)
**2041**	-1.3 (-2.1,-0.7)	-1.1 (-1.9,-0.5)	-1 (-1.6,-0.5)	0 (-0.1,0.1)	0 (-0.1,0.1)	-0.1 (-0.5,0.1)	-0.2 (-0.4,0.1)	0 (-0.1,0.1)
**2042**	-1.3 (-2.1,-0.5)	-1.1 (-1.8,-0.3)	-1 (-1.7,-0.3)	0 (-0.1,0.1)	0 (-0.1,0.1)	-0.1 (-0.5,0.1)	-0.1 (-0.4,0.1)	0 (-0.2,0.1)
**2043**	-1.1 (-2,-0.5)	-0.9 (-1.7,-0.3)	-0.8 (-1.7,-0.3)	0 (-0.1,0)	0 (-0.1,0.1)	-0.1 (-0.4,0.1)	-0.1 (-0.4,0.2)	0 (-0.2,0.1)

BMI = Body mass index; SBP = Systolic blood pressure

Table 7-4 - Relative change (%) in major illness incidence rates by scenario and year under theoretical minimum risk factor exposure levels compared to a base-case scenario of continuing trends

Year	All	BMI	Fruit & veg	Physical activity	SBP	Smoking	Total cholesterol
**2023**	-0.1 (-0.2,0)	0 (0,0)	0 (0,0)	0 (0,0)	-0.1 (-0.2,0)	0 (-0.1,0)	0 (0,0)
**2024**	-0.9 (-3.2,-0.5)	0 (-1.9,0)	0 (-0.1,0)	0 (-0.9,0)	-0.1 (-1.7,0)	-0.7 (-0.9,-0.5)	0 (-0.2,0)
**2025**	-1.9 (-4.9,-0.7)	0 (-2.5,0)	-0.1 (-0.3,0)	0 (-1.2,0)	-0.4 (-2.5,0)	-0.7 (-1,-0.4)	0 (-0.4,0)
**2026**	-4.5 (-7.6,-1.8)	-1.4 (-4.5,0)	-0.2 (-0.6,0)	-0.3 (-1.5,0)	-1.5 (-3.7,0)	-0.7 (-1,-0.4)	-0.1 (-0.7,0)
**2027**	-7.1 (-9.7,-2.9)	-3 (-5.8,-0.1)	-0.5 (-0.9,-0.1)	-1.1 (-1.5,0)	-2.4 (-5.4,-0.7)	-0.8 (-1.2,-0.4)	-0.3 (-0.9,0)
**2028**	-9.1 (-12.4,-6)	-4.7 (-8.4,-1.1)	-0.6 (-1,-0.3)	-1.3 (-1.8,-0.1)	-3.3 (-5.7,-1.6)	-0.8 (-1.3,-0.5)	-0.3 (-0.9,0)
**2029**	-10.3 (-12.8,-7.6)	-5.9 (-9,-3)	-0.7 (-1.1,-0.4)	-1.3 (-1.8,-0.4)	-3.6 (-5.5,-1.8)	-0.9 (-1.4,-0.5)	-0.4 (-0.9,-0.1)
**2030**	-10.9 (-13.3,-8.3)	-6.4 (-9.1,-4.1)	-0.7 (-1.1,-0.3)	-1.3 (-1.8,-0.9)	-3.5 (-5,-1.6)	-1.1 (-1.6,-0.5)	-0.4 (-0.9,-0.1)
**2031**	-11.1 (-14.1,-8.8)	-7 (-9.8,-5)	-0.7 (-1.2,-0.3)	-1.4 (-2.2,-0.7)	-3 (-4.7,-1.6)	-1.2 (-1.7,-0.9)	-0.4 (-0.8,-0.1)
**2032**	-12 (-14.4,-9.2)	-7.5 (-10.5,-5.1)	-0.7 (-1.1,-0.2)	-1.5 (-2.2,-0.8)	-2.9 (-4.8,-1.5)	-1.3 (-1.8,-0.8)	-0.4 (-0.9,0)
**2033**	-12.3 (-15.2,-9.7)	-7.8 (-11,-5.3)	-0.7 (-1.1,-0.3)	-1.6 (-2.4,-1.2)	-2.6 (-4.5,-1.5)	-1.4 (-2,-0.8)	-0.4 (-0.9,-0.1)
**2034**	-12.3 (-14.8,-9.3)	-8.1 (-10.4,-5.2)	-0.7 (-1.2,-0.2)	-1.6 (-2.2,-1.2)	-2.5 (-4.2,-1)	-1.6 (-2,-1)	-0.3 (-0.9,-0.1)
**2035**	-12.1 (-14.4,-9.2)	-8 (-10.1,-5.1)	-0.7 (-1.1,-0.3)	-1.5 (-2.1,-0.9)	-2.3 (-3.8,-0.9)	-1.6 (-2.2,-1.1)	-0.3 (-0.9,0)
**2036**	-11.7 (-14.3,-9)	-7.4 (-10.3,-5.1)	-0.6 (-1.1,-0.1)	-1.5 (-2,-0.9)	-2 (-3.6,-0.7)	-1.8 (-2.3,-1.2)	-0.3 (-0.8,0)
**2037**	-11.5 (-13.9,-9.1)	-7.8 (-10.2,-5.3)	-0.6 (-1.1,-0.2)	-1.4 (-2,-0.9)	-1.9 (-3.3,-0.7)	-1.7 (-2.3,-1.2)	-0.3 (-0.7,0)
**2038**	-11.4 (-13.5,-8.7)	-7.6 (-10,-5.2)	-0.6 (-1,-0.2)	-1.4 (-2,-0.7)	-1.7 (-3.3,-0.5)	-1.8 (-2.5,-1.1)	-0.3 (-0.8,0)
**2039**	-11.1 (-13.6,-8.3)	-7.5 (-10,-4.9)	-0.6 (-1,-0.1)	-1.2 (-1.9,-0.7)	-1.6 (-3,-0.2)	-1.7 (-2.3,-1.1)	-0.2 (-0.9,0)
**2040**	-10.8 (-13,-7.9)	-7.4 (-9.9,-4.7)	-0.5 (-0.9,-0.1)	-1.2 (-1.8,-0.6)	-1.3 (-3.1,-0.1)	-1.7 (-2.3,-1.1)	-0.2 (-0.7,0.1)
**2041**	-10.6 (-13.5,-7.4)	-7.4 (-10,-4.9)	-0.5 (-1,-0.1)	-1.2 (-1.9,-0.5)	-1.2 (-2.6,-0.1)	-1.7 (-2.3,-1.1)	-0.2 (-0.8,0.1)
**2042**	-9.9 (-12.6,-7.4)	-7.2 (-9.7,-4.5)	-0.5 (-0.9,-0.1)	-1.1 (-1.6,-0.4)	-1.1 (-2.2,-0.1)	-1.6 (-2.2,-0.9)	-0.1 (-0.6,0.1)
**2043**	-9.6 (-11.8,-6.8)	-6.9 (-9.2,-4.3)	-0.4 (-0.9,-0.1)	-1 (-1.7,-0.4)	-0.9 (-2.2,0.2)	-1.7 (-2.3,-1)	-0.2 (-0.8,0.1)

BMI = Body mass index; SBP = Systolic blood pressure

Table 7-5 - Relative change (%) in all-cause mortality rates by scenario and year under a 10% improvement in risk factors compared to a base-case scenario of continuing trends

Year	All	BMI	BMI sensitivity	Fruit & veg	Physical activity	SBP	Smoking	Total cholesterol
**2023**	0 (-0.1,0)	0 (0,0)	0 (0,0)	0 (0,0)	0 (0,0)	0 (0,0)	0 (0,0)	0 (0,0)
**2024**	0 (-0.2,0.1)	0 (-0.1,0)	0 (0,0)	0 (0,0)	0 (0,0)	0 (-0.1,0)	0 (-0.1,0.1)	0 (0,0)
**2025**	-0.1 (-0.8,0.1)	0 (-0.4,0)	0 (-0.1,0)	0 (-0.1,0)	0 (-0.1,0)	0 (-0.8,0)	0 (-0.3,0.1)	0 (-0.1,0)
**2026**	-0.4 (-1.1,0)	-0.1 (-0.5,0)	0 (-0.2,0)	0 (-0.1,0)	0 (-0.2,0)	-0.2 (-0.8,0)	-0.1 (-0.6,0.1)	0 (-0.2,0)
**2027**	-0.8 (-1.8,-0.3)	-0.3 (-0.8,0)	-0.1 (-0.2,0)	0 (-0.1,0)	0 (-0.2,0)	-0.3 (-1.1,0)	-0.2 (-0.7,0.1)	0 (-0.2,0)
**2028**	-1.3 (-2.5,-0.6)	-0.5 (-1.1,-0.2)	-0.1 (-0.3,0)	0 (-0.1,0)	-0.1 (-0.3,0)	-0.6 (-1.3,-0.1)	-0.4 (-0.8,0)	0 (-0.3,0)
**2029**	-1.5 (-2.3,-0.9)	-0.6 (-1.2,-0.2)	-0.1 (-0.3,0)	0 (-0.1,0)	-0.1 (-0.3,0)	-0.7 (-1.3,-0.2)	-0.5 (-0.9,-0.1)	-0.1 (-0.3,0)
**2030**	-1.6 (-2.5,-0.8)	-0.6 (-1.4,-0.3)	-0.2 (-0.7,0)	0 (-0.1,0)	-0.1 (-0.3,0)	-0.7 (-1.2,-0.2)	-0.5 (-1.1,-0.1)	0 (-0.2,0)
**2031**	-1.7 (-2.6,-0.9)	-0.7 (-1.4,-0.2)	-0.2 (-0.8,0)	0 (-0.2,0)	-0.1 (-0.3,0)	-0.7 (-1.2,-0.2)	-0.6 (-1,-0.2)	0 (-0.2,0)
**2032**	-2.1 (-3,-1.2)	-0.9 (-1.6,-0.3)	-0.5 (-1.1,0)	0 (-0.1,0)	-0.1 (-0.3,0)	-0.6 (-1,-0.2)	-0.8 (-1.1,-0.3)	0 (-0.2,0)
**2033**	-2.2 (-2.9,-1.6)	-0.9 (-1.7,-0.5)	-0.6 (-1.2,-0.2)	0 (-0.1,0.1)	-0.1 (-0.3,0)	-0.5 (-1,-0.1)	-0.8 (-1.3,-0.4)	0 (-0.2,0)
**2034**	-1.9 (-3,-1.2)	-0.9 (-1.6,-0.4)	-0.5 (-1.1,-0.2)	0 (-0.1,0.1)	-0.1 (-0.2,0.1)	-0.5 (-0.9,-0.1)	-0.8 (-1.2,-0.3)	0 (-0.2,0.1)
**2035**	-1.9 (-2.6,-1)	-0.9 (-1.6,-0.2)	-0.5 (-1.1,-0.1)	0 (-0.1,0.1)	-0.1 (-0.3,0)	-0.4 (-0.9,0.1)	-0.7 (-1.1,-0.3)	0 (-0.2,0.1)
**2036**	-1.8 (-2.6,-0.8)	-0.7 (-1.4,-0.2)	-0.5 (-1.1,-0.1)	0 (-0.2,0)	-0.1 (-0.3,0.1)	-0.4 (-0.9,0.1)	-0.7 (-1.2,-0.2)	0 (-0.1,0)
**2037**	-1.5 (-2.4,-0.7)	-0.7 (-1.3,-0.1)	-0.5 (-0.9,-0.1)	0 (-0.2,0.1)	-0.1 (-0.2,0.1)	-0.3 (-0.7,0.2)	-0.6 (-1.1,-0.1)	0 (-0.1,0.1)
**2038**	-1.4 (-2.2,-0.5)	-0.6 (-1.3,0)	-0.4 (-0.9,0)	0 (-0.1,0.1)	-0.1 (-0.2,0.1)	-0.2 (-0.7,0.1)	-0.6 (-1.2,0)	0 (-0.1,0.1)
**2039**	-1.3 (-2.1,-0.3)	-0.5 (-1.2,-0.1)	-0.4 (-0.8,0)	0 (-0.1,0.1)	0 (-0.2,0.1)	-0.2 (-0.6,0.3)	-0.4 (-1.1,0)	0 (-0.1,0.1)
**2040**	-1.1 (-2.3,-0.4)	-0.6 (-1.2,0)	-0.4 (-1,0)	0 (-0.2,0.1)	-0.1 (-0.2,0.1)	-0.2 (-0.6,0.3)	-0.4 (-0.9,0.1)	0 (-0.1,0.1)
**2041**	-1 (-1.7,-0.1)	-0.6 (-1.3,0.1)	-0.4 (-1.1,0)	0 (-0.1,0.1)	0 (-0.2,0.1)	-0.1 (-0.6,0.3)	-0.4 (-0.9,0.2)	0 (-0.1,0.1)
**2042**	-0.9 (-2.1,0)	-0.4 (-1.3,0.1)	-0.4 (-1.1,0.1)	0 (-0.1,0.1)	0 (-0.2,0.1)	-0.1 (-0.5,0.4)	-0.3 (-0.8,0.2)	0 (-0.1,0.1)
**2043**	-0.8 (-1.9,0)	-0.4 (-1.1,0.2)	-0.4 (-0.9,0.1)	0 (-0.2,0.1)	0 (-0.2,0.2)	0 (-0.5,0.3)	-0.3 (-0.9,0.2)	0 (-0.1,0.1)

BMI = Body mass index; SBP = Systolic blood pressure

Table 7-6 - Relative change (%) in all-cause mortality rates by scenario and year under theoretical minimum risk factor exposure levels compared to a base-case scenario of continuing trends

Year	All	BMI	Fruit & veg	Physical activity	SBP	Smoking	Total cholesterol
**2023**	-0.1 (-0.2,0)	0 (0,0)	0 (0,0)	0 (0,0)	0 (0,0)	-0.1 (-0.2,0)	0 (0,0)
**2024**	-0.8 (-1.7,-0.4)	0 (-0.4,0)	0 (-0.3,0)	0 (-0.7,0)	0 (-1,0)	-0.7 (-1,-0.4)	0 (-0.1,0)
**2025**	-1.6 (-5.2,-0.5)	0 (-0.5,0)	-0.1 (-0.6,0)	0 (-1,0)	0 (-3.8,0)	-0.7 (-3.9,-0.3)	0 (-0.8,0)
**2026**	-3.4 (-11.8,-1.5)	-0.3 (-0.9,0)	-0.4 (-1.1,0)	-0.5 (-7.7,0)	-1.1 (-4.7,0)	-0.8 (-4.8,-0.3)	0 (-1.2,0)
**2027**	-7.5 (-14.1,-2.7)	-0.5 (-1.3,0)	-0.6 (-1.5,-0.1)	-0.9 (-8,0)	-2.2 (-5.5,-0.3)	-0.8 (-4.7,-0.4)	-0.3 (-1,0)
**2028**	-11.3 (-16.5,-4.3)	-0.9 (-1.6,-0.2)	-0.7 (-1.5,-0.3)	-5.3 (-8.4,-0.3)	-3.9 (-6.1,-1)	-3.7 (-5.1,-0.5)	-0.4 (-1.5,0)
**2029**	-13.6 (-16.2,-7.1)	-1 (-1.9,-0.4)	-0.8 (-1.4,-0.4)	-5.3 (-7.9,-0.5)	-4.1 (-6.3,-1.9)	-4 (-5.3,-0.4)	-0.5 (-1.1,0)
**2030**	-13.7 (-19,-9.2)	-1.2 (-6,-0.6)	-0.8 (-1.4,-0.3)	-5.1 (-7.5,-0.8)	-4 (-6.1,-2.1)	-4.1 (-5.4,-0.9)	-0.4 (-1,-0.1)
**2031**	-13.5 (-18.2,-10.4)	-1.5 (-6.1,-0.7)	-0.7 (-1.6,-0.3)	-4.7 (-7.5,-2.4)	-3.7 (-5.4,-2.1)	-4.1 (-5.2,-2.9)	-0.4 (-0.9,0)
**2032**	-15.3 (-19.4,-10.9)	-3.4 (-7.8,-1)	-0.8 (-1.3,-0.2)	-4.6 (-6.9,-2.8)	-3.4 (-4.9,-1.9)	-4.1 (-5.4,-2.9)	-0.3 (-0.9,0)
**2033**	-16 (-20,-12.6)	-4.9 (-8.4,-2.5)	-0.7 (-1.5,-0.3)	-4.1 (-6.6,-2.4)	-2.8 (-4.5,-1.5)	-4.2 (-5.3,-3.3)	-0.3 (-0.8,0)
**2034**	-15.1 (-19.3,-12.4)	-4.3 (-8.1,-2.3)	-0.7 (-1.4,-0.3)	-3.7 (-5.4,-2.4)	-2.5 (-4.2,-1.4)	-4.3 (-5.4,-3.3)	-0.2 (-0.8,0)
**2035**	-14.5 (-18,-11.2)	-4.4 (-7.1,-1.9)	-0.7 (-1.2,-0.2)	-3.2 (-5,-1.9)	-2.4 (-3.5,-0.9)	-4.4 (-5.6,-3.4)	-0.2 (-0.8,0.1)
**2036**	-14 (-17.6,-11.2)	-4.2 (-7.3,-2.1)	-0.6 (-1.4,-0.2)	-2.9 (-4.5,-1.7)	-2 (-3.4,-0.7)	-4.5 (-6,-3.4)	-0.2 (-0.7,0)
**2037**	-13 (-16.5,-10.3)	-3.9 (-6.6,-2.2)	-0.5 (-1.1,0)	-2.5 (-4.6,-1.4)	-1.6 (-3.1,-0.5)	-4.6 (-6.1,-3.3)	-0.1 (-0.6,0.1)
**2038**	-12.6 (-15.4,-9.5)	-3.8 (-6.7,-1.6)	-0.5 (-1.1,0.1)	-2.3 (-3.9,-1.1)	-1.4 (-3.2,-0.2)	-4.7 (-5.8,-3.6)	-0.1 (-0.5,0.1)
**2039**	-12.2 (-15.7,-9.2)	-3.7 (-6.7,-2)	-0.4 (-1,0.1)	-2.1 (-3.8,-0.7)	-1.1 (-2.7,0)	-4.8 (-5.7,-3.7)	-0.1 (-0.4,0.1)
**2040**	-11.5 (-14.8,-8.6)	-3.4 (-5.9,-1.6)	-0.4 (-0.9,0.1)	-2 (-3.4,-0.4)	-1 (-2.1,0)	-4.8 (-6.1,-3.5)	-0.1 (-0.5,0.1)
**2041**	-10.9 (-14.4,-8.5)	-3.1 (-6.1,-1.5)	-0.3 (-0.9,0.2)	-1.8 (-2.9,-0.7)	-0.8 (-2.2,0.5)	-4.8 (-5.9,-3.4)	-0.1 (-0.4,0.1)
**2042**	-10.7 (-14.2,-8.1)	-3.4 (-5.9,-1.1)	-0.3 (-1,0.2)	-1.8 (-3.1,-0.8)	-0.7 (-2,0.1)	-4.6 (-5.8,-3.6)	-0.1 (-0.5,0.2)
**2043**	-10.5 (-12.6,-7.7)	-3.3 (-5.1,-1.3)	-0.3 (-1,0.3)	-1.7 (-2.9,-0.4)	-0.5 (-1.5,0.6)	-4.5 (-5.9,-3.3)	-0.1 (-0.4,0.1)

BMI = Body mass index; SBP = Systolic blood pressure

8. Sensitivity analyses

No effect of improved risk factor exposures on mortality

The prevalence rates of major illness (CMS > 1.5), minor illness (0 < CMS <= 1.5), and no illness (CMS = 0) change very little between modelling and not modelling an association between improved risk factor exposures and survival, for both the 10% improvement and theoretical minimum risk exposure level scenarios (Figure 7-1). If no association is modelled (sensitivity analysis), then the prevalence rate of major illness is slightly lower than in the main results, and a slightly greater proportion of people have no CMS conditions. The total projected population size, however, is greater when an association with survival is modelled, as simulants at all levels of CMS score have improved mortality rates and therefore live longer at a given level of health (Figure 7-2)

Figure 8-1 – Absolute change in prevalence of three levels of CMS score under two assumptions of the effect of risk factor improvements on mortality

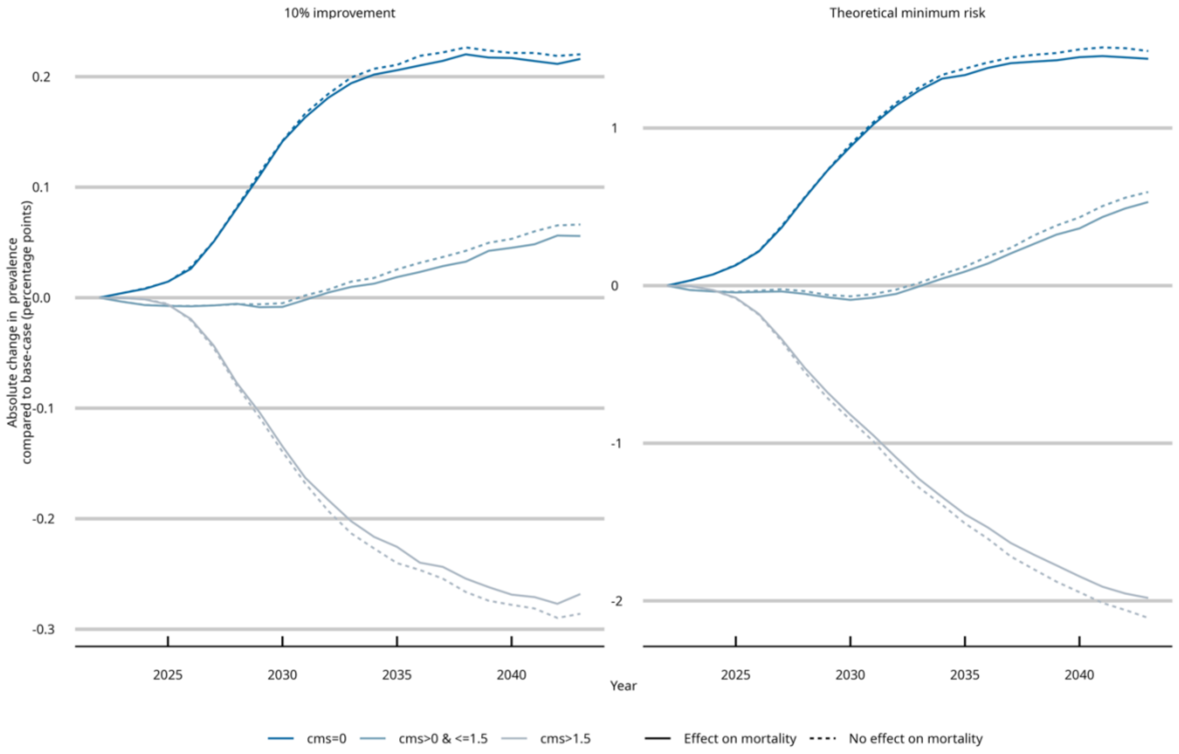

CMS = Cambridge multimorbidity score

Figure 8-2 – Absolute change in projected population size ('000s) under two assumptions of the effect of risk factor improvements on mortality

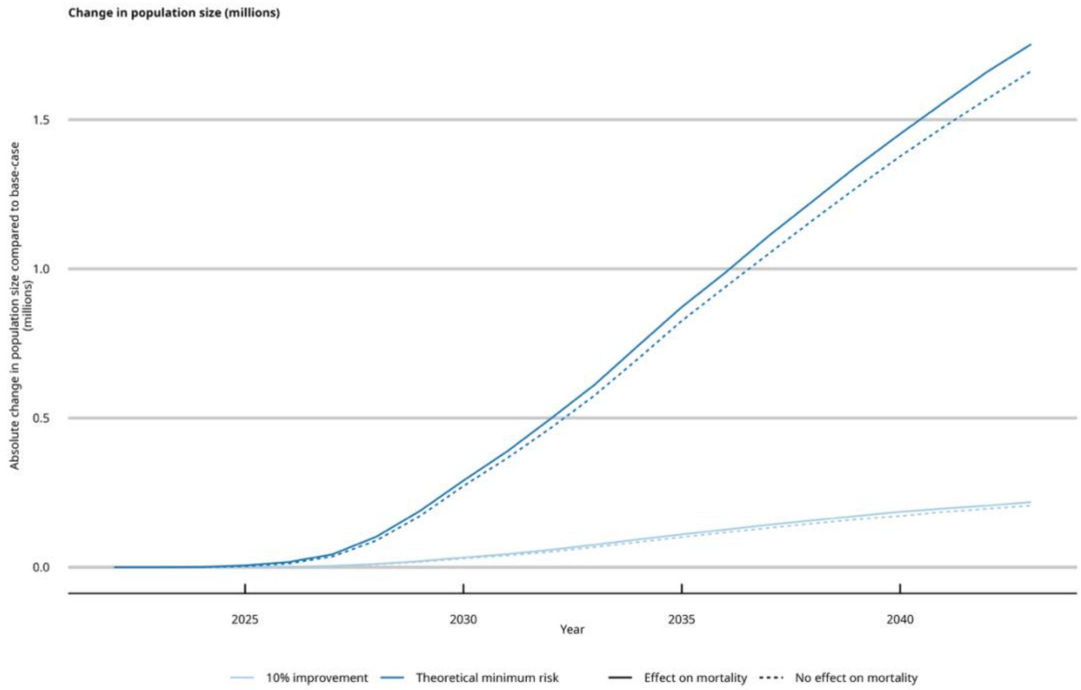

Alternative major illness thresholds

Figure 8-3 – Absolute change in prevalence of major illness as defined by three Cambridge Multimorbidity Score (CMS) thresholds: CMS > 1, CMS > 1.5 (main analysis), and CMS > 2

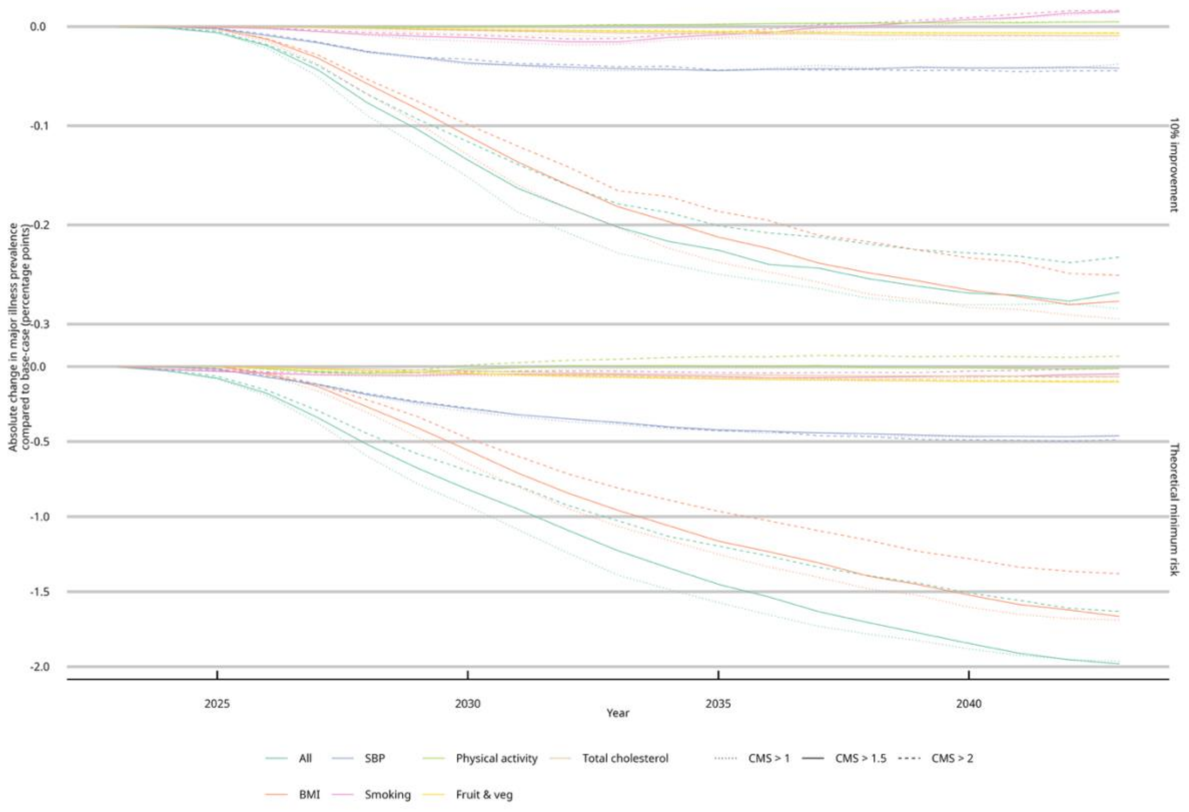

All curves are smoothed; BMI = Body mass index; SBP = Systolic blood pressure

In sensitivity analyses adjusting our threshold CMS score for major illness (Alternative major illness thresholds (Figure 7-3), for the BMI and all risk factor scenarios, there were slightly greater absolute reductions in prevalence for a lower threshold of CMS > 1, and smaller reductions for a threshold of CMS > 2. For all other scenarios, reductions in major illness prevalence were similar regardless of CMS threshold used. Patterns over time and between risk factors were similar to our main analysis threshold (CMS > 1.5).

Indirect effect of BMI through SBP and total cholesterol

Our final sensitivity analysis modelled the direct effect only of a 10% improvement in BMI, excluding the indirect effect through improved SBP and total cholesterol. This would suggest a slightly lesser reduction in prevalence of major illness in 2043: a 0.2pp reduction (0.2,0.3) compared to 0.3pp (0.2,0.4) if the indirect effect is also modelled (Figure 7-4).

Figure 8-4 – Absolute change in prevalence of major illness under a 10% improvement in BMI with ("BMI") and without ("BMI direct effect only") modelling the indirect effect of BMI on SBP and total cholesterol

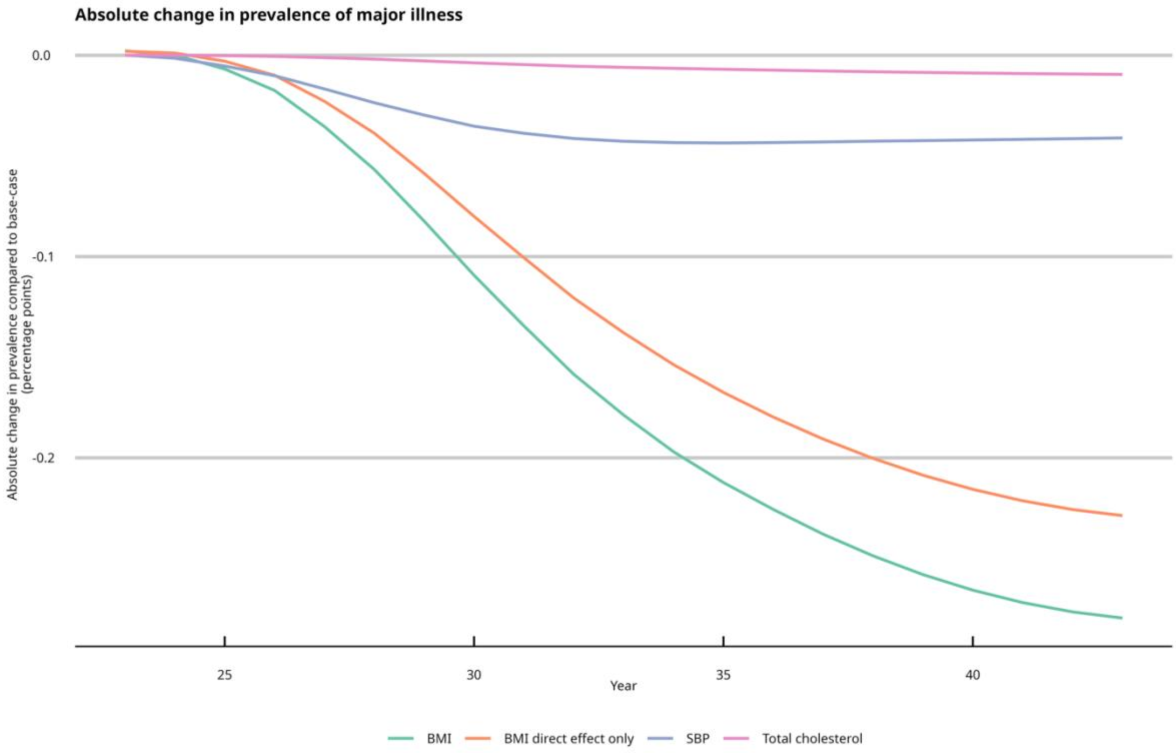

All curves are smoothed; *Major illness is defined as a Cambridge Multimorbidity Score > 1.5; 10% improvement in SBP and total cholesterol scenarios included for comparison; BMI = Body mass index; SBP = Systolic Blood Pressure

RECORD Statement

The RECORD statement – checklist of items, extended from the STROBE statement, that should be reported in observational studies using routinely collected health data

	Item No.	STROBE items	Location in manuscript where items are reported	RECORD items	Location in manuscript where items are reported
Title and abstract					
	1	(a) Indicate the study's design with a commonly used term in the title or the abstract<br>(b) Provide in the abstract an informative and balanced summary of what was done and what was found	a) title<br><br>b) abstract	RECORD 1.1: The type of data used should be specified in the title or abstract. When possible, the name of the databases used should be included.<br><br>RECORD 1.2: If applicable, the geographic region and timeframe within which the study took place should be reported in the title or abstract.<br><br>RECORD 1.3: If linkage between databases was conducted for the study, this should be clearly stated in the title or abstract.	1.1 Introduction (abstract only 150 words)<br><br>1.2 title and abstract<br><br>1.3 Introduction (abstract only 150 words)
Introduction					
Background rationale	2	Explain the scientific background and rationale for the investigation being reported	Introduction p3-4		
Objectives	3	State specific objectives, including any prespecified hypotheses	Introduction p4,		
Methods					
Study Design	4	Present key elements of study design early in the paper	Brief summary in Introduction p4, more detail in methods p18 & supplementary methods		
Setting	5	Describe the setting, locations, and relevant dates, including periods of recruitment, exposure, follow-up, and data collection	Introduction p4, *Data sources* section of methods p13-14		
Participants	6	*(a) Cohort study* - Give the eligibility criteria, and the sources and methods of selection of participants. Describe methods of follow-up<br>*Case-control study* - Give the eligibility criteria, and the sources and methods of case ascertainment and control selection. Give the rationale for the choice of cases and controls<br>*Cross-sectional study* - Give the eligibility criteria, and the sources and methods of selection of participants<br><br>*(b) Cohort study* - For matched studies, give matching criteria and number of exposed and unexposed<br>*Case-control study* - For matched studies, give matching criteria and the number of controls per case	a) *Data sources* section of methods p13-14 & Supplementary methods<br>b) N/A	RECORD 6.1: The methods of study population selection (such as codes or algorithms used to identify subjects) should be listed in detail. If this is not possible, an explanation should be provided.<br><br>RECORD 6.2: Any validation studies of the codes or algorithms used to select the population should be referenced. If validation was conducted for this study and not published elsewhere, detailed methods and results should be provided.<br><br>RECORD 6.3: If the study involved linkage of databases, consider use of a flow diagram or other graphical display to demonstrate the data linkage process, including the number of individuals with linked data at each stage.	6.1 summary in *data sources (p13-14)*, more detail in supplementary methods and link to online, open-access information<br><br>6.2 link provided to original code lists and implementation code<br><br>6.3 not included specifically for linkage. Data flowchart included in Supplementary methods, p24
Variables	7	Clearly define all outcomes, exposures, predictors, potential confounders, and effect modifiers. Give diagnostic criteria, if applicable.	methods sections: *risk factors and disease incidence, health outcomes pp14-15*	RECORD 7.1: A complete list of codes and algorithms used to classify exposures, outcomes, confounders, and effect modifiers	7.1 provided link to online, open-access information

				should be provided. If these cannot be reported, an explanation should be provided.	
Data sources/ measurement	8	For each variable of interest, give sources of data and details of methods of assessment (measurement). Describe comparability of assessment methods if there is more than one group	Full details available in the Supplementary methods		
Bias	9	Describe any efforts to address potential sources of bias	Discussion pp8-12		
Study size	10	Explain how the study size was arrived at	Supplementary methods		
Quantitative variables	11	Explain how quantitative variables were handled in the analyses. If applicable, describe which groupings were chosen, and why	Supplementary methods		
Statistical methods	12	(a) Describe all statistical methods, including those used to control for confounding<br>(b) Describe any methods used to examine subgroups and interactions<br>(c) Explain how missing data were addressed<br>(d) *Cohort study* - If applicable, explain how loss to follow-up was addressed<br>*Case-control study* - If applicable, explain how matching of cases and controls was addressed<br>*Cross-sectional study* - If applicable, describe analytical methods taking account of sampling strategy<br>(e) Describe any sensitivity analyses	a) Supplementary methods – Epidemiological engine<br>b) Supplementary methods – Epidemiological engine<br>c) complete-case analysis.<br>d) N/A<br>e) N/A		
Data access and cleaning methods		..		RECORD 12.1: Authors should describe the extent to which the investigators had access to the database population used to create the study population.<br><br>RECORD 12.2: Authors should provide information on the data cleaning methods used in the study.	12.1 Not included in the manuscript. We used a simple random sample of participants meeting our eligibility criteria.<br><br>12.2 Supplementary methods
Linkage		..		RECORD 12.3: State whether the study included person-level, institutional-level, or other data linkage across two or more databases. The methods of linkage and methods of linkage quality evaluation should be provided.	12.3 *Data sources.* Methods & quality evaluation not discussed.
Results					
Participants	13	(a) Report the numbers of individuals at each stage of the study (*e.g.*, numbers potentially eligible, examined for eligibility, confirmed eligible, included in the study, completing follow-up, and analysed)<br>(b) Give reasons for non-participation at each stage.<br>(c) Consider use of a flow diagram	a-c) Supplementary methods p 24	RECORD 13.1: Describe in detail the selection of the persons included in the study (*i.e.,* study population selection) including filtering based on data quality, data availability and linkage. The selection of included persons can be described in the text and/or by means of the study flow diagram.	13.1 Supplementary methods p 24
Descriptive data	14	(a) Give characteristics of study participants (*e.g.*, demographic, clinical, social) and information on exposures and potential confounders<br>(b) Indicate the number of participants with missing data for each variable of interest<br>(c) *Cohort study* - summarise follow-up time (*e.g.*, average and total amount)	a) Not included<br>b) Not included<br>c) Not included		

Outcome data	15	*Cohort study* - Report numbers of outcome events or summary measures over time<br>*Case-control study* - Report numbers in each exposure category, or summary measures of exposure<br>*Cross-sectional study* - Report numbers of outcome events or summary measures	Results and Supplementary Results		
Main results	16	(a) Give unadjusted estimates and, if applicable, confounder-adjusted estimates and their precision (e.g., 95% confidence interval). Make clear which confounders were adjusted for and why they were included<br>(b) Report category boundaries when continuous variables were categorized<br>(c) If relevant, consider translating estimates of relative risk into absolute risk for a meaningful time period	Not applicable		
Other analyses	17	Report other analyses done—e.g., analyses of subgroups and interactions, and sensitivity analyses	Validation reported in Supplementary methods.<br>Sensitivity analyses results in Supplementary results		
Discussion					
Key results	18	Summarise key results with reference to study objectives	Discussion 1st paragraph		
Limitations	19	Discuss limitations of the study, taking into account sources of potential bias or imprecision. Discuss both direction and magnitude of any potential bias	Limitations reported in discussion<br>Potential direction/magnitude not discussed as unknown	RECORD 19.1: Discuss the implications of using data that were not created or collected to answer the specific research question(s). Include discussion of misclassification bias, unmeasured confounding, missing data, and changing eligibility over time, as they pertain to the study being reported.	Relevant points reported in discussion and in the supplementary methods
Interpretation	20	Give a cautious overall interpretation of results considering objectives, limitations, multiplicity of analyses, results from similar studies, and other relevant evidence	Discussion		
Generalisability	21	Discuss the generalisability (external validity) of the study results	Discussion (in relation to other studies)		
Other Information					
Funding	22	Give the source of funding and the role of the funders for the present study and, if applicable, for the original study on which the present article is based	Funding statement		
Accessibility of protocol, raw data, and programming code		..		RECORD 22.1: Authors should provide information on how to access any supplemental information such as the study protocol, raw data, or programming code.	Data sharing statement; all code available online

*Reference: Benchimol EI, Smeeth L, Guttmann A, Harron K, Moher D, Petersen I, Sørensen HT, von Elm E, Langan SM, the RECORD Working Committee. The REporting of studies Conducted using Observational Routinely-collected health Data (RECORD) Statement. *PLoS Medicine* 2015; in press.

*Checklist is protected under Creative Commons Attribution (CC BY) license.
