# Supplementary material for: Exploring the contribution of risk factors on major illness: a microsimulation study in England, 2023-2043
Source: Nat Commun. 2025 Nov 4;16:9402. doi: 10.1038/s41467-025-64820-1 (PMC12586531; doi:10.1038/s41467-025-64820-1)
Supplement: Supplementary file 2 — Reporting Summary [file 41467_2025_64820_MOESM2_ESM.pdf]

nature portfolio

Corresponding author(s): Anna Head

Last updated by author(s): 2025/07/28

Reporting Summary

Nature Portfolio wishes to improve the reproducibility of the work that we publish. This form provides structure for consistency and transparency in reporting. For further information on Nature Portfolio policies, see our Editorial Policies and the Editorial Policy Checklist.

Statistics

For all statistical analyses, confirm that the following items are present in the figure legend, table legend, main text, or Methods section.

n/a	Confirmed	
☐	☒	The exact sample size (*n*) for each experimental group/condition, given as a discrete number and unit of measurement
☒	☐	A statement on whether measurements were taken from distinct samples or whether the same sample was measured repeatedly
☒	☐	The statistical test(s) used AND whether they are one- or two-sided<br>*Only common tests should be described solely by name; describe more complex techniques in the Methods section.*
☐	☒	A description of all covariates tested
☐	☒	A description of any assumptions or corrections, such as tests of normality and adjustment for multiple comparisons
☐	☒	A full description of the statistical parameters including central tendency (e.g. means) or other basic estimates (e.g. regression coefficient) AND variation (e.g. standard deviation) or associated estimates of uncertainty (e.g. confidence intervals)
☒	☐	For null hypothesis testing, the test statistic (e.g. *F*, *t*, *r*) with confidence intervals, effect sizes, degrees of freedom and *P* value noted<br>*Give P values as exact values whenever suitable.*
☒	☐	For Bayesian analysis, information on the choice of priors and Markov chain Monte Carlo settings
☒	☐	For hierarchical and complex designs, identification of the appropriate level for tests and full reporting of outcomes
☒	☐	Estimates of effect sizes (e.g. Cohen's *d*, Pearson's *r*), indicating how they were calculated

Our web collection on statistics for biologists contains articles on many of the points above.

Software and code

Policy information about availability of computer code

Data collection	This project used only secondary data. No primary data was collected.
Data analysis	The IMPACTNCD_Engl model is written primarily in the R programming language, for which version 4.2.3 was the latest release at the time of submission. C++ code was also used.<br>Code for the model is available here: https://doi.org/10.5281/zenodo.16314082

For manuscripts utilizing custom algorithms or software that are central to the research but not yet described in published literature, software must be made available to editors and reviewers. We strongly encourage code deposition in a community repository (e.g. GitHub). See the Nature Portfolio guidelines for submitting code & software for further information.

Data

Policy information about availability of data

All manuscripts must include a data availability statement. This statement should provide the following information, where applicable:

- Accession codes, unique identifiers, or web links for publicly available datasets
- A description of any restrictions on data availability
- For clinical datasets or third party data, please ensure that the statement adheres to our policy

This study did not generate any original raw data. All data used were from secondary data sources, each of which are detailed below.

Linked CPRD-HES-ONS data: The individual-level health data used in this study were obtained from the Clinical Practice Research Datalink (CPRD) Aurum database after approval by the CPRD independent scientific advisory panel (protocol ISAAC 20_000096). A detailed protocol for this study can be provided upon request. Access to anonymised data from CPRD is subject to a full licence agreement containing detailed terms and conditions of use. Anonymised patient datasets can be extracted for researchers against specific study specifications, following protocol approval. Further information is available at www.cprd.com/data-access.
Data citation: Clinical Practice Research Datalink. (2021). CPRD Aurum June 2021 (Version 2021.06.001) [Data set]. Clinical Practice Research Datalink. https://doi.org/10.48329/pyc2-we97

HSE data: Data on risk factor exposures were obtained from the Health Survey for England, made available via the UK Data Service (UKDS). Access to UKDS data is subject to their end user licence agreement.
Data citation: NatCen Social Research, University College London, Department of Epidemiology and Public Health. (2024). Health Survey for England. [data series]. 8th Release. UK Data Service. SN: 2000021, DOI: http://doi.org/10.5255/UKDA-Series-2000021

ONS data: Data on population estimates, population projections, and mortality are available directly from the Office for National Statistics website.
Data citation: Office for National Statistics (ONS), released 30 January 2024, ONS website, statistical bulletin, National population projections: 2021-based interim. Available from: https://www.ons.gov.uk/peoplepopulationandcommunity/populationandmigration/populationprojections/bulletins/nationalpopulationprojections/2021basedinterim
Number of deaths and populations in deprivation decile areas by sex and single year of age, England and Wales, registered years 2001 to 2018 - Office for National Statistics [Internet]. [cited 2022 Feb 18]. Available from: https://www.ons.gov.uk/peoplepopulationandcommunity/birthsdeathsandmarriages/deaths/adhocs/11169deathregistrationsandpopulationsbyindexofmultipledeprivationengland2001to2018

The aggregated derived data underlying the microsimulation model is available here: https://doi.org/10.5281/zenodo.16314082

Research involving human participants, their data, or biological material

Policy information about studies with human participants or human data. See also policy information about sex, gender (identity/presentation), and sexual orientation and race, ethnicity and racism.

Reporting on sex and gender	The variable of gender within CPRD (our main data source) is self-reported sex; we assumed that male and female refer to biological sex.
Reporting on race, ethnicity, or other socially relevant groupings	Within the model, we have used the nine ethnicity groupings from the 2011 UK Census (white, Indian, Pakistani, Bangladeshi, other Asian, Black Caribbean, Black African, Chinese, and others). Results are not disaggregated by ethnicity.<br><br>We have used the deciles of the 2015 English Index of Multiple Deprivation as a measure of socioeconomic deprivation.
Population characteristics	Adults aged 30 and over.
Recruitment	N/A Our study did not involve primary collection of data.
Ethics oversight	The CPRD Independent Scientific Advisory Panel approved the study protocol [ISAAC 20_000096]. CPRD has ethics approval from the Health Research Authority to support research using anonymised patient data.

Note that full information on the approval of the study protocol must also be provided in the manuscript.

Field-specific reporting

Please select the one below that is the best fit for your research. If you are not sure, read the appropriate sections before making your selection.

☐ Life sciences ☒ Behavioural & social sciences ☐ Ecological, evolutionary & environmental sciences

For a reference copy of the document with all sections, see nature.com/documents/nr-reporting-summary-flat.pdf

Behavioural & social sciences study design

All studies must disclose on these points even when the disclosure is negative.

Study description	A dynamic discrete-time microsimulation model. The model combines quantitative data from linked primary care records (Clinical Practice Research Datalink Aurum (CPRD), Hospital Episodes Statistics (HES), and Office for National Statistics (ONS) Mortality data), Health Survey for England (HSE), ONS population estimates, systematic reviews and meta-analyses.
Research sample	Linked CPRD-HES-ONS data: secondary use of routinely collected healthcare data: linked primary care records (Clinical Practice Research Datalink (CPRD) Aurum linked to Hospital Episodes Statistics (HES) inpatient, HES outpatient, and Office for National Statistics (ONS) mortality records). We used a random sample of 2m patients registered between 2008-2019 at GP practices in England contributing to CPRD Aurum. The sample is considered representative of the GP-registered population in age and sex distribution. This model only uses data from adults aged 20 and over.<br>HSE survey data: an annual cross-sectional survey on health and wellbeing in England, from which we used data on risk factor exposures. The survey sampling is conducted to be representative of the community-dwelling population in England. Data are collected for all ages; we used data from adults aged 20 and over.

	ONS population estimates: estimates and projections of the population size and structure in England over time.<br>All of these datasets are widely considered the most representative for the population in England with regards to disease burden, exposure to risk factors and populations size and age/sex distribution.
Sampling strategy	Linked CPRD-HES-ONS data: random sampling. The size of the sample was chosen based on data minimisation guidelines from the data provider. A sample size calculation was not conducted; previous work using a 1million sample indicated sufficient size to allow multiple stratification levels required in the sample.<br>HSE survey data: stratified random probability sample of households. sampling methods for this survey are documented on the UK Data Service website: https://doi.org/10.5255/UKDA-Series-2000021 We did no further sampling<br>ONS population estimates: full population estimates - no sampling.
Data collection	Linked CPRD-HES-ONS data: secondary use of routine healthcare data recorded as part of patient care. Linked ONS mortality data is secondary use of cause of death data recorded as part of death registration. As research is a secondary use of this data, blinding is not applicable.<br>HSE survey data: questionnaire-based interview (predominantly in person) with physical measurements and a follow-up visit by a biomedical fieldworker for blood and urine samples.<br>ONS population estimates: based on census data, and registration data of vital events and migration.
Timing	Linked CPRD-HES-ONS data: routinely collected data from patient care. The latest date used for our analyses was 31 December 2019. Our sample was derived from patients registered at participating GP practices at any point between 2008-2019; past healthcare records (where available) were included in prevalence calculations, but excluded from incidence calculations.<br>HSE survey data: annual waves for 2003-2014
Data exclusions	Linked CPRD-HES-ONS data: random sampling. The size of the sample was chosen based on data minimisation guidelines from the data provider. A sample size calculation was not conducted; previous work using a 1million sample indicated sufficient size to allow multiple stratification levels required in the sample.<br>HSE survey data: stratified random probability sample of households. sampling methods for this survey are documented on the UK Data Service website: https://doi.org/10.5255/UKDA-Series-2000021 We did no further sampling
Non-participation	HSE survey data: information on non-participation rates are available within study documentation on the UK Data Service website: https://doi.org/10.5255/UKDA-Series-2000021
Randomization	N/A Our study does not allocate participants to different experimental groups. Statistical analyses and projections are based on sociodemographic characteristics.

Reporting for specific materials, systems and methods

We require information from authors about some types of materials, experimental systems and methods used in many studies. Here, indicate whether each material, system or method listed is relevant to your study. If you are not sure if a list item applies to your research, read the appropriate section before selecting a response.

Materials & experimental systems

n/a	Involved in the study
☒	☐ Antibodies
☒	☐ Eukaryotic cell lines
☒	☐ Palaeontology and archaeology
☒	☐ Animals and other organisms
☒	☐ Clinical data
☒	☐ Dual use research of concern
☒	☐ Plants

Methods

n/a	Involved in the study
☒	☐ ChIP-seq
☒	☐ Flow cytometry
☒	☐ MRI-based neuroimaging

Plants

Seed stocks	*Report on the source of all seed stocks or other plant material used. If applicable, state the seed stock centre and catalogue number. If plant specimens were collected from the field, describe the collection location, date and sampling procedures.*
Novel plant genotypes	*Describe the methods by which all novel plant genotypes were produced. This includes those generated by transgenic approaches, gene editing, chemical/radiation-based mutagenesis and hybridization. For transgenic lines, describe the transformation method, the number of independent lines analyzed and the generation upon which experiments were performed. For gene-edited lines, describe the editor used, the endogenous sequence targeted for editing, the targeting guide RNA sequence (if applicable) and how the editor was applied.*
Authentication	*Describe any authentication procedures for each seed stock used or novel genotype generated. Describe any experiments used to assess the effect of a mutation and, where applicable, how potential secondary effects (e.g. second site T-DNA insertions, mosiacism, off-target gene editing) were examined.*
